# Overlooked uneven progress across sustainable development goals at the global scale: Challenges and opportunities

**DOI:** 10.1016/j.xinn.2024.100573

**Published:** 2024-01-08

**Authors:** Yali Liu, Jianqing Du, Yanfen Wang, Xiaoyong Cui, Jichang Dong, Pan Gu, Yanbin Hao, Kai Xue, Hongbo Duan, Anquan Xia, Yi Hu, Zhi Dong, Bingfang Wu, Jürgen P. Kropp, Bojie Fu

**Affiliations:** 1 School of Grassland Science, Beijing Forestry University, Beijing 100083, China; 2 Beijing Yanshan Earth Critical Zone National Research Station, University of Chinese Academy of Sciences, Beijing 101408, China; 3 College of Resources and Environment, University of Chinese Academy of Sciences, Beijing 100049, China; 4 State Key Laboratory of Tibetan Plateau Earth System, Environment and Resources, Chinese Academy of Sciences, Beijing 100101, China; 5 College of Life Sciences, University of Chinese Academy of Sciences, Beijing 100049, China; 6 School of Economics and Management, University of Chinese Academy of Sciences, Beijing 100190, China; 7 School of Humanities, University of Chinese Academy of Sciences, Beijing 100049, China; 8 Key Laboratory of Adaptation and Evolution of Plateau Biota, Northwest Institute of Plateau Biology, Chinese Academy of Sciences, Xining 810001, China; 9 School of Innovation and Entrepreneurship, University of Chinese Academy of Sciences, Beijing 100190, China; 10 State Key Laboratory of Remote Sensing Science, Aerospace Information Research Institute, Chinese Academy of Sciences, Beijing 100101, China; 11 Potsdam Institute for Climate Impact Research, 14412 Potsdam, Germany; 12 Institute for Environmental Science and Geography, University of Potsdam, 14412 Potsdam, Germany; 13 State Key Laboratory of Urban and Regional Ecology, Research Center for Eco-Environmental Sciences, Chinese Academy of Sciences, Beijing 100085, China

## Abstract

Differences in progress across sustainable development goals (SDGs) are widespread globally; meanwhile, the rising call for prioritizing specific SDGs may exacerbate such gaps. Nevertheless, how these progress differences would influence global sustainable development has been long neglected. Here, we present the first quantitative assessment of SDGs’ progress differences globally by adopting the SDGs progress evenness index. Our results highlight that the uneven progress across SDGs has been a hindrance to sustainable development because (1) it is strongly associated with many public health risks (e.g., air pollution), social inequalities (e.g., gender inequality, modern slavery, wealth gap), and a reduction in life expectancy; (2) it is also associated with deforestation and habitat loss in terrestrial and marine ecosystems, increasing the challenges related to biodiversity conservation; (3) most countries with low average SDGs performance show lower progress evenness, which further hinders their fulfillment of SDGs; and (4) many countries with high average SDGs performance also showcase stagnation or even retrogression in progress evenness, which is partly ascribed to the antagonism between climate actions and other goals. These findings highlight that while setting SDGs priorities may be more realistic under the constraints of multiple global stressors, caution must be exercised to avoid new problems from intensifying uneven progress across goals. Moreover, our study reveals that the urgent needs regarding SDGs of different regions seem complementary, emphasizing that regional collaborations (e.g., demand-oriented carbon trading between SDGs poorly performed and well-performed countries) may promote sustainable development achievements at the global scale.

## Public summary

• Integrating progress evenness reveals hidden challenges and opportunities toward sustainable development goals (SDGs).
• Uneven SDGs progress is related to health risks, ecosystem damage, and social inequality.
• Uneven SDGs progress is common in the low-income and extremely arid countries.
• Many high-income countries face antagonism between climate actions and other goals.
• Complementary collaborations are crucial to achieve SDGs.

## Graphical abstract

**Figure:**
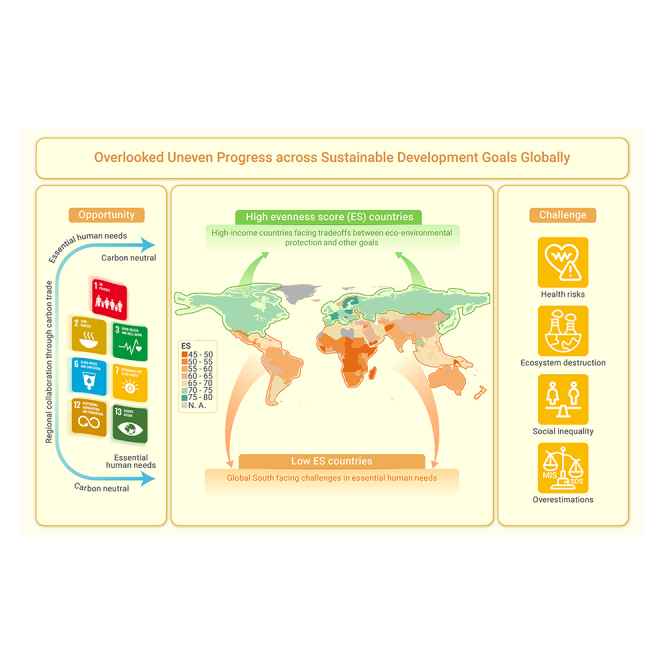

## Introduction

The 17 sustainable development goals (SDGs) were put forward at the 2015 United Nations Summit, and aim to provide a shared blueprint for peace and prosperity for all people and our planet.^1^ As we move toward 2030, multiple global stressors such as the coronavirus disease 2019 (COVID-19) pandemic and the Russo-Ukrainian conflict cast a shadow over the holistic achievements proposed by the SDGs.^2^^,^^3^ These multiple and simultaneous crises have directly threatened the SDGs regarding the goals no poverty, zero hunger, good health and well-being, and affordable and clean energy, and indirectly through its hindering of globalization.^2^^,^^3^^,^^4^ Moreover, they have diverted policy and public attention away from long-term planning and investment,^3^ leading to the rising call for a focus on more immediate and clearer SDG priorities instead of on the holistic achievement of the SDGs.^2^^,^^5^

These global stressors have also exposed, if not amplified, the conflicts over targets.^2^ Given that potential priorities such as food security, biodiversity conservation, and climate actions widely interact with other goals,^6^^,^^7^^,^^8^^,^^9^^,^^10^ the overemphasis on one or a few priorities could impede the achievement of the others. For instance, increasing food production could intensify emerging human infectious diseases globally.^6^ In addition, although the reduction in human activities during the COVID-19 pandemic could benefit environment-related targets,^4^^,^^11^^,^^12^^,^^13^^,^^14^ the strict regulations that emerged, such as travel restrictions, have largely slowed down globalization^15^ and may expose the global economy and food security to further threats.^2^ The Russo-Ukrainian conflict has also plunged Europe into a deep energy crisis, likely intensifying the tradeoffs between sustainable energy consumption and other SDGs.^3^ In general, these progress differences across SDGs, which already exist because of the socioeconomic conditions and/or the interactions among SDGs,^16^^,^^17^^,^^18^^,^^19^^,^^20^^,^^21^ may worsen because of these external disturbances.

It remains, nonetheless, that we currently lack a comprehensive understanding of the progress differences across SDGs at the global scale, whether and how they may influence SDGs achievement, and whether they may affect priority decision making in this context. The most recent assessment systems use the arithmetic mean of all 17 SDGs to quantify a given country’s or region’s SDG performance^13^^,^^22^^,^^23^ without considering the differences in performance across goals. An example of such methods is the widely used SDG index score proposed by the SDG Index and Dashboards 2016.^23^ Given their limitations, these methods could yield overoptimistic results; for example, they may yield a high average SDG performance because of a few well-achieved goals that have tradeoffs with others—such as a rapid economic development at the cost of environmental protection.^24^ Moreover, considering only this average score is very likely to lead to the neglect of the challenges caused by an uneven progress across goals. For instance, Liu et al. suggested that many top-income regions in China, whose performance toward SDGs was previously thought to be relatively good, were reaching a bottleneck because of their uneven development pathway.^24^ Such uneven progress could lead to challenges such as water depletion and air pollution.^25^^,^^26^^,^^27^^,^^28^ As such, it limits the policy-making capacity toward SDGs, particularly in choosing priorities without bringing up new problems.

Here, we innovatively adopted the concept of “progress evenness” from biodiversity measurements in ecology^29^ to quantify the global pattern of progress differences toward SDGs by country from 2017 to 2021 (hereafter referred to as SDGs progress evenness), and then investigated the associated challenges (explained further in the materials and methods section). A low SDGs progress evenness reflects a significant progress difference—that is, an uneven progress across SDGs.^24^ This score is supposed to supplement the existing major SDGs assessment indicator (i.e., the mean SDG index score [MIS]), which may then be combined to form a novel two-dimensional assessment system by integrating the average performance across all 17 SDGs and their progress differences (explained further in the materials and methods section).

Our results highlight that an uneven progress, rather than poor economic performance, is associated more with problems such as health risks, ecosystem destruction, and social inequality. Low-income countries and drylands exhibited the lowest SDGs progress evenness, which further broadens their gaps in approaching the SDGs. Meanwhile, many high-income countries (HICs) had setbacks in the holistic achievement of SDGs because of the stagnation or even retrogression in SDGs progress evenness. While considering SDGs progress evenness, we estimated that only approximately 30% of countries would be able to achieve SDGs by 2030—an underwhelming achievement that is far below the projections made based on previous SDGs assessment methods. These findings underscore that future policy making should consider SDGs progress evenness, especially in choosing priorities. Nevertheless, the uneven progress poses not only global challenges but also opportunities for international cooperation to restore and accelerate SDGs progress toward 2030 and beyond.

## Results

### Methods summary

In general, the MIS, the SDGs progress evenness score (ES), and their integration (i.e., the geometric mean of MIS and ES, hereafter referred to as sustainable development score [SDS]) all were considered to present a comprehensive understanding of global sustainable development. A radar chart method^24^ was adopted to quantify ES in each country, and we used a binary regression to study how ES relates to human health, social equality, and the environment, in addition to the economic condition as the major influencing factor. Then, to tap into the hidden challenges left behind by past assessments using exclusively the MIS, we reassessed the spatial and temporal progress toward SDGs by examining the ES global pattern from 2017 to 2021 and integrated it with the widely adopted MIS.^4^^,^^13^^,^^30^^,^^31^^,^^32^ This enabled us to investigate whether and how uneven progress across goals has impeded the holistic achievement of SDGs globally. Moreover, in response to the rising calls for reprioritizing SDGs under global stressors, we conducted analyses to explore the SDGs that require urgent prioritization by integrating each country’s poorly performed SDGs and development pathway.

### Social and environmental issues associated with uneven progress across SDGs

By controlling for economic development level across countries using gross domestic product (GDP) per capita, we revealed that uneven progress across SDGs was related to global challenges such as public health issues, environmental damage, and social inequality. Firstly, ES was positively related to life expectancy, strongly related to a high mortality rate for neonates and young children younger than 5 years, and elevated the age-standardized death rate owing to air pollution (Table 1). The binary regression further showed that these public health issues were related more to ES than to GDP per capita, because the former had much higher standardized coefficients (β) than the latter (from approximately 1.8 to 4.8 times; Table 1). To summarize, ES and GDP per capita could explain 70% of the variance in life expectancy. Some of these public health issues may be ascribed partly to environmental pollution and social inequality, which were also related to an uneven progress across SDGs. For instance, we observed an increase in the annual mean concentration of particulate matter of less than 2.5 μm in diameter (PM_2.5_), with a decrease in ES (Table 1). Further analyses suggested that an elevated PM_2.5_ could increase the aforementioned mortality rates and reduce life expectancy (Figure S1; Table S1).

**Table 1:** Effects of GDP per capita and ES on the indicators

Factor	Indicators related to health											
	Neonatal mortality rate, per 1,000 live births *R2* = 0.67, *F*_2,155_ = 161.23			Mortality rate under 5, per 1,000 live births *R2* = 0.63, *F*_2,155_ = 135.75			Age-standardized death rate owing to air pollution, per 100,000 population *R2* = 0.60, *F*_2,155_ = 118.88			Life expectancy at birth, years *R2* = 0.70, *F*_2,155_ = 186.30		
	β	t	p	β	t	p	β	t	p	β	t	p
GDP per capita	−0.23	−4.38	<0.001	−0.15	−2.69	0.008	−0.29	−4.99	<0.001	0.35	7.01	<0.001
ES	−0.69	−13.24	<0.001	−0.72	−13.03	<0.001	−0.60	−10.47	<0.001	0.62	12.50	<0.001

Factor	Indicators related to the environment											
	Annual mean concentration of PM 2.5, μg/m³ *R*^*2*^ = 0.17, *F*_2,155_ = 16.77			Mean area that is protected in marine sites important to biodiversity, % *R*^*2*^ = 0.16, *F*_2,109_ = 11.68			Mean area that is protected in terrestrial sites important to biodiversity, % *R*^*2*^ = 0.13, *F*_2,153_ = 12.23			Permanent deforestation, % of forest area, 5-year average *R*^*2*^ = 0.11, *F*_2,141_ = 9.80		
	β	t	P	β	t	p	β	t	p	β	t	p
GDP per capita	−0.20	−2.43	0.02	0.13	1.25	0.20	0.21	2.50	0.01	−0.12	−1.26	0.18
ES	−0.29	−3.47	0.001	0.34	3.39	0.001	0.22	2.56	0.01	−0.27	−3.01	0.003

Factor	Indicators related to equality											
	Ratio of female-to-male mean year of education received, % *R2* = 0.25, *F*_2,151_ = 26.74			Seats held by women in national parliament, % *R*^*2*^ = 0.06, *F*_2,155_ = 6.31			Victims of modern slavery, per 1,000 population *R2* = 0.22, *F*_2,137_ = 20.96			Gini coefficient adjusted for top income *R*^*2*^ = 0.27, *F*_2,140_ = 27.36		
	β	t	p	β	t	P	β	t	p	β	t	p
GDP per capita	0.13	1.64	0.10	0.22	2.5	0.01	−0.18	−2.07	0.04	−0.29	−3.45	0.001
ES	0.44	5.52	<0.001	0.09	1.06	0.30	−0.37	−4.37	<0.001	−0.33	−3.95	<0.001

All models are significant at p < 0.05. β, standardized coefficient; PM_2.5_, particulate matter of <2.5 μm in diameter.

In the path analysis, ES still exhibited a strong direct influence on all four indicators related to public health (Figure S1), indirectly affecting public health through PM_2.5_ and social inequality factors (i.e., ratio of female-to-male of education received, modern slavery, and the Gini coefficient). Unsurprisingly, although PM_2.5_ was an essential mediator affecting the age-standardized death rate owing to air pollution (Figure S1C), it did not contribute much to the mortality rate for neonates and young children younger than 5 years (Figures S1A and S1B) or to life expectancy (Figure S1D). Regarding social inequality factors, an elevated ES was associated with an increase in female rights, which could then strongly decrease the mortality rate for neonates and young children younger than 5 years (Figures S1E and S1F) while increasing life expectancy (Figure S1H).

ES was also crucial for biodiversity conservation, because a high ES was related to a larger protected area important for terrestrial and marine biodiversity, whereas a low ES was strongly related to deforestation (Table 1). Similar to the findings for public health issues, ES had a greater influence on biodiversity conservation indicators than did GDP per capita (Table 1). Moreover, both low ES and GDP per capita were related to social inequality (e.g., gender inequality, modern slavery, the rich–poor gap; Table 1). ES was also much more influential on female education level and modern slavery than GDP per capita (β = 0.44 versus 0.13, respectively, for female education level; β = −0.37 versus −0.18, respectively, for modern slavery).

### Spatial pattern of global sustainable development when incorporating SDGs progress evenness

**Figure 1:**
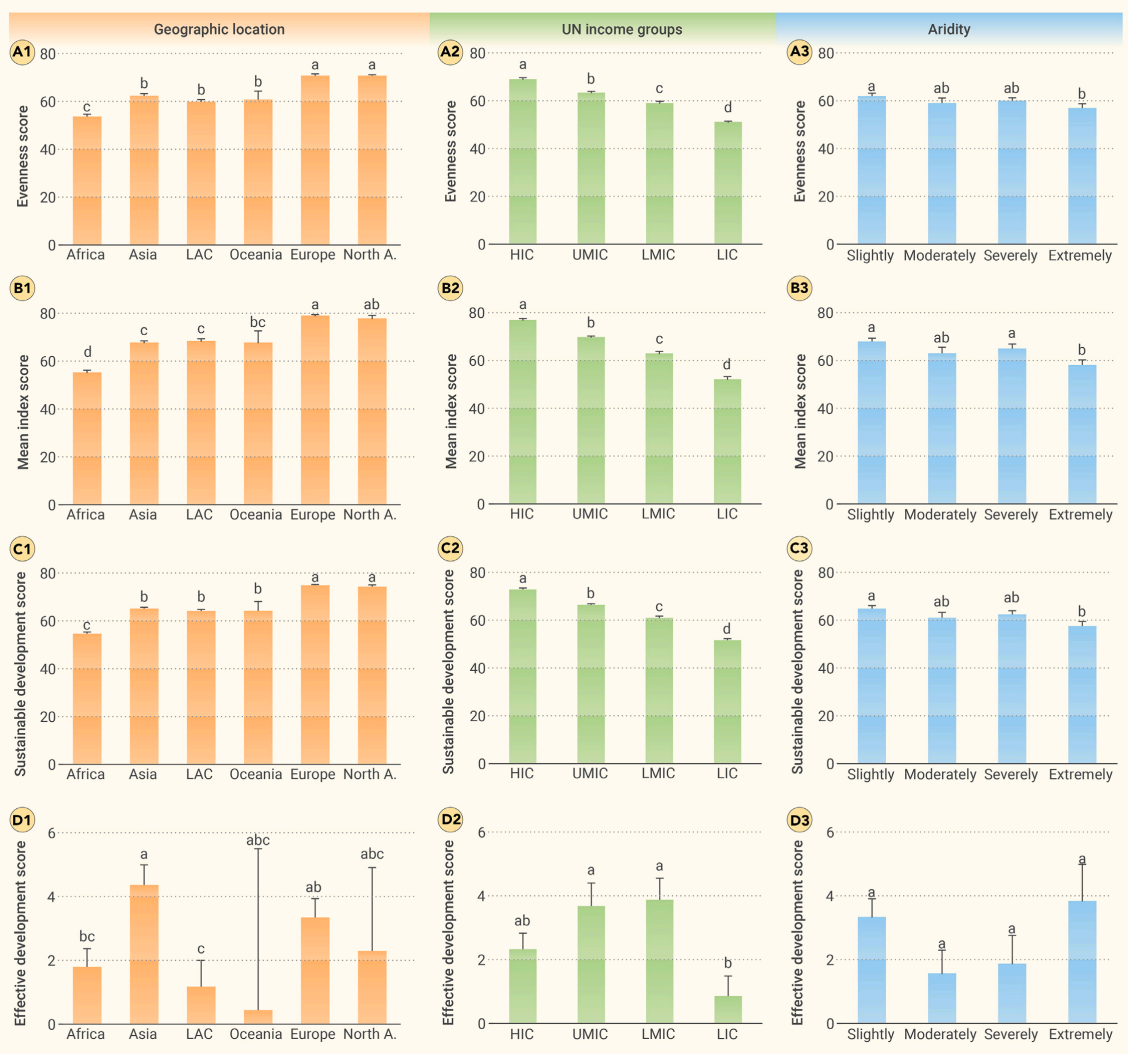
Differences in the ES (A1–A3), MIS (B1–B3), SDS (C1–C3), and EDS (D1–D3) across geographic locations, United Nations income groups, and arid levels in 2021 The histogram with error bars presents the mean value ±SE. Different lowercase letters visualize the significant differences at p < 0.05. EDS is based on data from 2017 to 2021. HIC, high-income countries; LAC, Latin America and the Caribbean; LIC, low-income countries; LMIC, lower-middle-income countries; North A., North America; UMIC, upper-middle-income countries.

While considering SDGs progress evenness (explained further in the materials and methods section), we reassessed the progress toward SDGs in 169 countries. Our results highlight that regions with a low MIS also had a low ES, whereas high-MIS regions had a high ES (Figures 1A and 1B); for instance, in 2021, HIC (ie, most countries in Europe and North America; Figure S2; Table S2) had the highest MIS and ES (76.70 for MIS, 68.74 for ES), whereas low-income countries (LICs; in general, in Africa; Figure S2) had the lowest MIS and ES (51.88 for MIS, 50.83 for ES; Table S3). In addition, slightly arid countries also had higher MIS and ES than extremely arid countries (67.89 versus 58.11, respectively, for MIS; 61.95 versus 56.97, respectively, for ES; Table S3). Consequently, countries facing the greatest challenges were LICs in drylands, namely Chad, Somalia, Niger, Yemen, Mali, Burkina Faso, and Gambia. These countries had a lower SDS (see the materials and methods section for the definition of SDS) than nonextremely arid LICs (49.49 versus 52.06, respectively).

**Figure 2:**
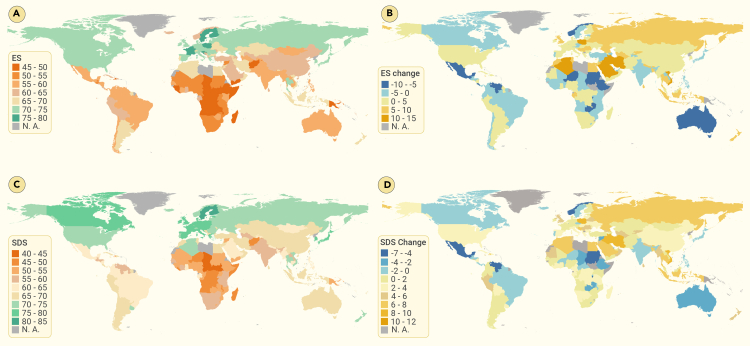
The spatial pattern of the ES and the sustainable development score (SDS) in 2021 and corresponding changes from 2017 to 2021 (A) ES; (B) change in ES; (C) SDS; (D) change in SDS. ES and SDS increase from red to green; notably, the change in ES and SDS increases from blue (negative) to orange (positive). N.A., not available.

The progress toward SDGs has varied mainly by regions of the globe, regardless of the index under consideration (ie, MIS, ES, and SDS; Figures 1A—1C and 2). Based on the SDS in 2021, Sub-Saharan Africa, the Caribbean, and West and South Asia were considered poorly performing regions, whereas high-income regions such as North America and Europe generally were considered well-performing regions (Figure 2C; Table S2). In general, the deficiencies in the poorly performing regions were related to SDGs 1 (no poverty), 2 (zero hunger), and 9 (industry, innovation and infrastructure), whereas the deficiencies in the well-performing regions were related to SDGs 12 (responsible consumption and production), 14 (life below water), and 17 (partnerships; Figure S3).

### Progress toward SDGs over time

**Figure 3:**
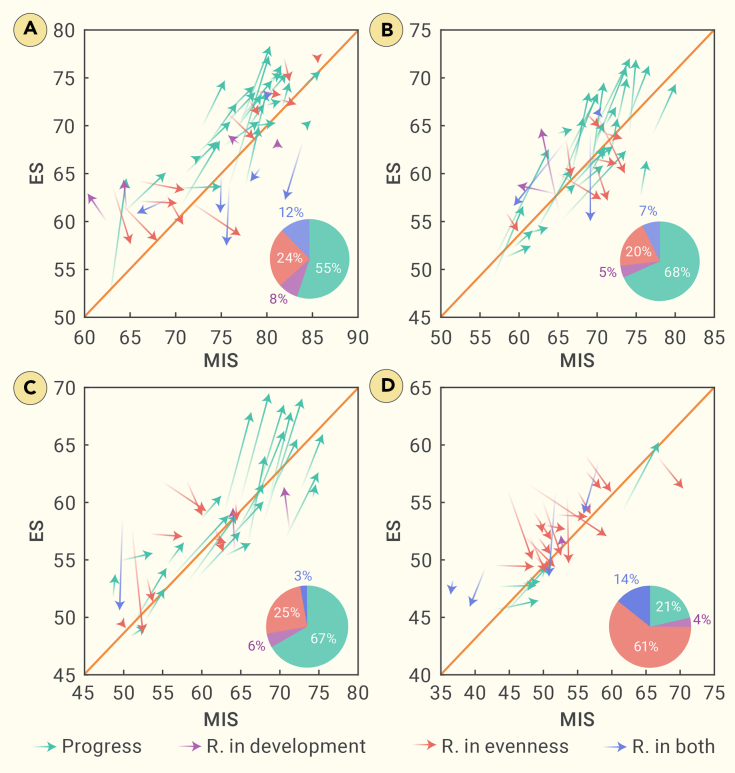
Development pathways for different United Nations income groups from 2017 to 2021 (A) HICs; (B) UMICs; (C) LMICs; (D) LICs. Different colors are used to represent the 4 types of development pathways: progress (green; progressing pathway), R. in development (retrogression in development, purple; underdeveloped pathway), R. in evenness (retrogression in evenness, red; uneven development pathway), and R. in both (retrogression in both, blue; retrogressive pathway). The orange diagonal stands for the ideal pathway (slope = 1). The pie charts at the bottom right corners show the proportion of different pathways.

The development pathway toward SDGs and the effective development score (EDS; i.e., the vector defined by a given country’s pairwise MIS and ES between 2017 and 2021) were used to evaluate the progress toward SDGs from 2017 to 2021 (explained further in the materials and methods section). The findings show that well-performing countries (mostly HICs) had their own challenges, because they generally made little or even negative SDS progress from 2017 to 2021. The number of countries experiencing a retrogression increased from 10 to 22 (44.9%) when considering ES (Figure 3A; Table S2), because some countries had setbacks only in ES. For example, the MIS of the Czech Republic increased from 63.10 to 65.06 from 2017 to 2021, whereas the ES decreased from 63.59 to 57.63; this can be ascribed largely to solid progress in SDG 9 but a setback in SDG 13 (climate action). As a result, the EDS of HIC was lower than that of middle-income countries (Figure 1D2). Nevertheless, the most undesirable pathway was still found in LICs, generally showing an uneven development pathway toward SDGs (decrease in ES; Figure 3D).

In 2020, HICs had the lowest EDS, even less than LICs (1.50 versus 1.51; Figure S4), albeit HICs also showed more resistance to the effects of the COVID-19 pandemic than LICs, because the latter had the lowest EDS in 2021 (decreased to 0.83; Figure 1D2). Moreover, more countries stepped into an uneven development pathway (ie, retrogression in ES plus retrogression in both ES and MIS) in 2021, with the proportion of uneven development pathways increasing from 19% to 27%, 25% to 28%, and 71% to 75% for upper-middle-income countries, lower-middle-income countries, and LICs, respectively (Figures 3 and S5). The only exception was found in HICs, which rarely showed changes in the proportion of uneven development pathways (39% in 2020 versus 36% in 2021).

The relationships among SDGs were also analyzed across income and aridity levels to study the potential causes of an uneven development pathway. Our results highlighted that the significant interactions among SDGs tended to be weaker in LICs (versus HICs) and extremely arid countries (versus slightly arid; Figures S6A and S6B). For example, a strong negative relationship between SDG 12 (responsible consumption and production) and other goals only existed in HICs (Figure S6A). The goals that generally correlated negatively with most other goals were SDGs 12 and 13 (Figure S6; Table S4). Compared with the prepandemic situation in 2020, the tradeoffs intensified in lower-middle-income countries and moderately arid countries, especially those between SDG 14 and other SDGs (Figures S6 and S7).

### Projection of SDG performance across countries in 2030

**Figure 4:**
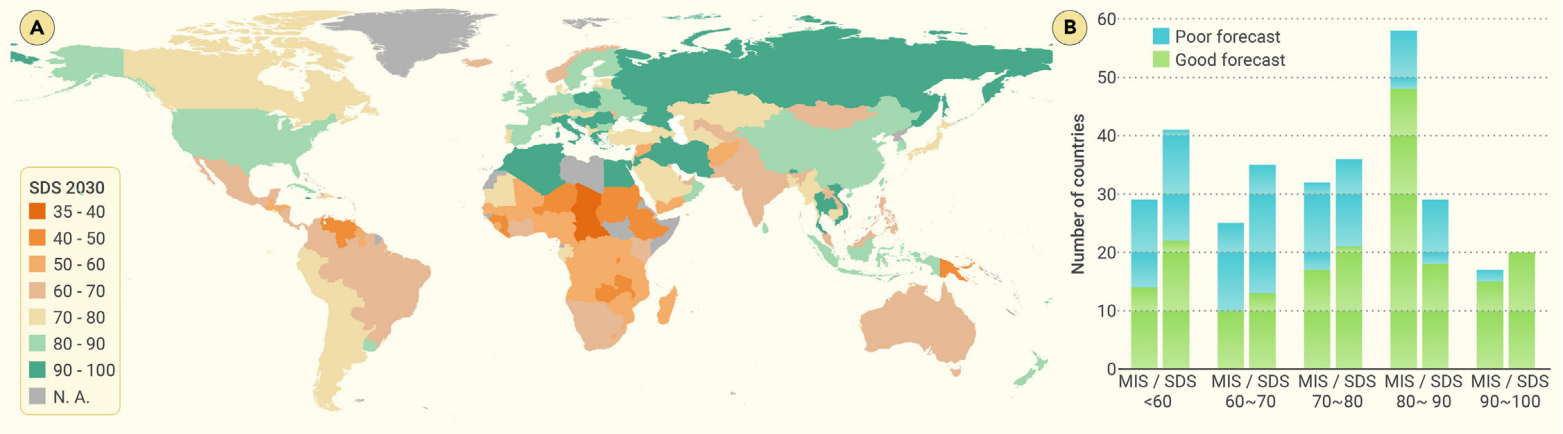
Projections of SDG performance across countries in 2030 based on trends from 2017 to 2021 (A) Global variation of the SDS; (B) the number of countries with different scores projected by the MIS and SDS. SDS increases from red (poorly performing) to green (well performing).

We projected the MIS and SDS in 2030 using curvilinear regression and a gray forecast model based on data from 2017 to 2021. Upon considering ES in the analyses, only 26.7% (by curvilinear regression) and 34.1% (by gray forecast model) of countries would be able to approach the SDGs (i.e., projected score higher than 80 based on data from 2017 to 2021) in 2030, which is much less than the projection using only MIS (41% and 47.2% based on curvilinear regression and gray forecast model, respectively; Figure 4B; Table S5). In addition, the number of countries with a projected SDS lower than 60 was much higher when considering ES than when using only MIS (27–28 using MIS versus 40–41 using SDS; Figure 4B).

These results align with the challenges mentioned above for the relatively well-performing and poorly performing countries. In addition, the situation could be even worse, because most countries with SDS projections above 80 in 2030 were middle-income countries (56% and 49% in curvilinear regression and gray forecast model, respectively; Figure 4A; Table S5). Furthermore, from 2017 to 2021, middle-income countries exhibited the most desirable development pathway toward SDGs (Figures 3B and 3C).

### Urgency and potential for priority development

In response to the rising calls to reprioritize SDGs under global stressors, we integrated each country’s poorly performing SDGs and development pathway toward the SDGs to identify goals that should be urgently prioritized (explained further in the materials and methods section).

In 2021, most countries were considered to have either a relatively sustainable performance (relatively high MIS and ES) or an underdeveloped and uneven performance (relatively low MIS and ES). Meanwhile, approximately 51.5% of countries (85 countries) showed an uneven performance across SDGs (relatively low ES; Table S6). There were 42 African countries (all LICs; out of 48 African countries) and 13 extremely arid countries (out of 20 extremely arid countries) that were classified as having an uneven performance, and only 36 countries with an uneven performance did not belong to any of these 3 categories (Figure S8). Fifty-one countries were classified as poorly performing on essential human needs and considered to have uneven performance (Table S6)—41 were in Africa, 46 were LICs or lower-middle-income countries, and 21 were extremely or severely arid countries.

**Figure 5:**
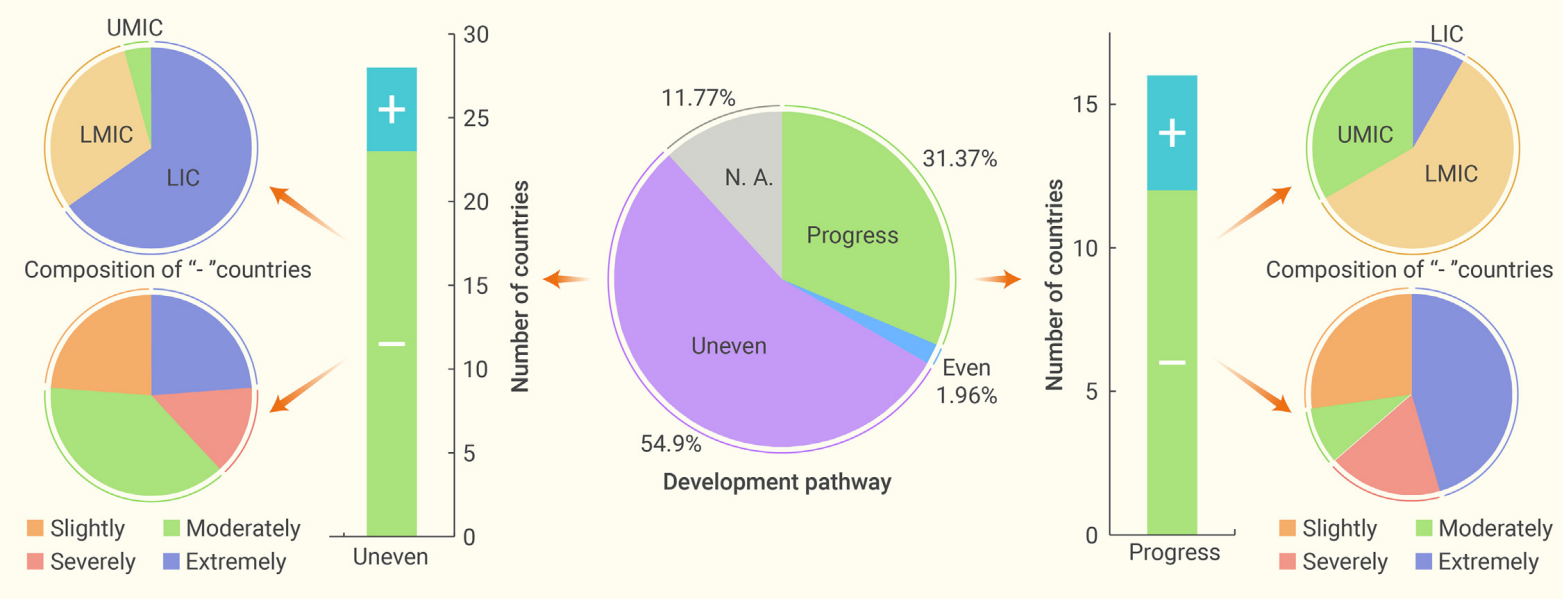
Composition of the 51 countries with a relatively poor performance on essential human needs All 51 countries were classified to have an uneven development pathway toward SDGs. The pie chart in the center represents the ratio for each development pathway. Progress stands for progress in both MIS and ES; Even refers to progress only in ES; Uneven refers to retrogression in ES. “+” and “-” stand for countries with increasing or decreasing scores in essential human needs, respectively. The composition of countries marked with “-” is shown in the small pie chart.

As for the development pathway toward the SDGs from 2017 to 2021, 28 countries with an uneven performance (out of 51) also showed an uneven development pathway (decrease in ES), and the other 17 showed an even pathway (increase in ES; data not available for 6 countries; Figure 5). Among the 16 countries with a progressing pathway (increase in both MIS and ES), 4 countries showed improved performance in essential human needs; the other 12 (10 from Africa and 2 from Asia) showed an average decline of 4.97 points in essential human needs and a general considerable progress in eco-environmental protection and economic development (average increase of 13.14 and 9.64, respectively). Therefore, these 12 countries may need to focus more on supporting essential human needs in the future. Five countries (out of the 28 countries with an uneven development pathway) with an uneven development pathway showed an increasing average score for essential human needs, albeit not as high as the increase in eco-environmental protection and economic development (Table S6). The other 23 countries (out of the 28 countries with an uneven development pathway) generally showed the worst performance in essential human needs (11/23) and had an average decrease of 4.37 points from 2017 to 2021 (Figure 5; Table S6); they were thus considered to be the countries that need to most urgently prioritize the development of essential human needs. These countries also had a distinct geographic feature, because most were in Africa (except Bolivarian Republic of Venezuela, India, and Lao People’s Democratic Republic [Laos]), suggesting that countries in Africa, particularly LICs and lower-middle-income African countries, need to reprioritize their development pathways regarding SDGs.

Meanwhile, 49 countries, generally HICs (43/49 countries; 24 in Europe, 12 in Asia, and 0 in Africa) with good performance on essential human needs and economic development (Table S6), had poor performance regarding eco-environmental protection. The challenge for these countries may be striking a balance between human development and eco-environmental protection. Furthermore, the number of countries with a relatively sustainable performance decreased by 10 from 2020 to 2021 (87 in 2020 versus 77 in 2021), and the proportion of countries with poor performance in eco-environmental protection among countries with a relatively sustainable performance increased from 37.93% to 51.95% during the same period (Figure S9).

## Discussion

### Major challenges associated with an uneven progress across SDGs

This study exposes various global issues related to public health, social inequality, and the environment by considering SDGs progress evenness. Our results suggest that SDGs progress evenness has a greater influence (versus GDP per capita) on public health issues (e.g., the mortality rate of neonates and young children younger than 5 years and the age-standardized death rate owing to air pollution) and a stronger association with life expectancy. These findings regarding SDGs progress evenness are partly ascribed to the environmental pollution (e.g., PM_2.5_, ozone, and nitrogen pollution) associated with an uneven progress across SDGs. For instance, elevated PM_2.5_ concentration—which was shown to be associated with an uneven progress across SDGs in this study—is a crucial risk factor for pulmonary diseases, cancers, diabetes, and cardiovascular health,^33^^,^^34^^,^^35^ and could increase the premature mortality rate.^12^ These findings of past studies are generally concordant with our results. Moreover, our results indicate that the uneven progress across SDGs could result in social inequalities such as gender inequality, which further reduce life expectancy.^36^^,^^37^ This study also demonstrates that that biodiversity conservation becomes more challenging when under the constraints of an uneven progress across SDGs, which was mainly associated with deforestation and habitat loss in terrestrial and marine ecosystems. Furthermore, the regions that we identified to have the lowest SDGs progress evenness—Sub-Saharan Africa, Latin America and the Caribbean, and South and Southeast Asia—were also identified in a past study to be global priority areas for ecosystem restoration to ensure the efficient promotion of biodiversity conservation.^38^ Growing evidence suggests that ecosystem destruction and fragmentation increases the risk of disease transmission to humans^5^^,^^39^^,^^40^^,^^41^; thus, these aforementioned regions may face greater challenges regarding public health in the near future. To summarize, the global issues associated with an uneven progress across SDGs could impede SDGs achievement, particularly if this uneven status intensifies because of multiple global stressors.

In the present study, low-income and extremely arid countries showed a low SDGs progress evenness and the worst average SDG performance. Given the aforementioned problems associated with an uneven progress across SDGs, we can infer that the situation of these regions is even worse than previously reported.^13^ A case study in China recently reported a similar conclusion, demonstrating that the country’s north and northwest arid regions are low-income regions with poor SDGs performance.^24^ These results imply that extreme droughts could be a crucial factor restricting sustainable development in the future. Such findings are of particular significance under the accelerated expansion of drylands under climate change.^42^ At the same time, our findings show that the progress of many HICs reached a bottleneck, because many of these countries (44.9%) showed a retrogression development pathway from 2017 to 2021, although they also generally had the best average performance toward SDGs. Such stagnant progress for economically developed regions was also reported in the aforementioned study in China,^24^ and is probably due to the tradeoffs between some SDGs.^16^^,^^17^^,^^19^ In our evidence, the SDGs that negatively correlated with most other SDGs were SDGs 12 and 13, and this is corroborated by previous studies.^16^^,^^19^^,^^20^ Our results also indicate that HICs and countries under economic or environmental stresses face divergent problems; although HICs need to achieve development in a responsible and sustainable way, the latter are struggling to fulfill the essential needs for human survival.^13^ Although the dilemmas vary by region, the challenges that seem to generally hinder the holistic achievement of SDGs remain to be securing responsible consumption and production and dealing with the associated climate issues,^43^ such as carbon emissions reduction, which is becoming more and more challenging.^44^

Among one of the most alarming and problematic findings of the present study, we observed that the incorporation of SDGs progress evenness into the analysis led to a projection of only approximately 30% of the countries analyzed being able to achieve the SDGs by 2030. This indicates that the ostensibly good average SDGs performance of many countries is primarily related to improvements in only a few of the goals. Moreover, many countries that were observed in this study to have the potential to achieve SDGs by 2030 are middle-income countries; considering their income characteristics, it may be hard for these countries to be able to sustain their noteworthy trends toward SDGs. Some of the reasons for this are as follows: (1) they may be caught in the middle-income trap, as many countries are^45^^,^^46^; (2) even if they manage to avoid the middle-income trap (e.g., by ramping domestic demand and/or finding new markets) and become HICs, they are very likely to step into the same bottlenecks as HICs; and (3) their progress could be affected by global stressors such as military clashes, energy crises, and global health risks.^47^ Therefore, prioritizing development in essential human needs rather than overoptimistically pursuing a holistic achievement of all SDGs seems more realistic under the multiple current global stressors.^2^

This study also identifies that LICs in Africa are the countries that most urgently need to reprioritize their development pathways toward SDGs, and that they should focus particularly on SDGs 1, 3 (good health and well-being), 7 (affordable and clean energy), and 9. Among these SDGs that may need to be prioritized, SDG 7 exhibited strong associations with the others. A recent study suggested that replacing traditional fuel with clean energy in rural pasture areas could improve the local livelihood by providing electricity, lowering health risks, and reducing indoor air pollution (e.g., from burning coal or cow dung), and lead to improvements regarding performance on SDGs 1 and 3.^48^ Moreover, there is an expected decrease in the cost of clean energy for the near future. For instance, solar power is predicted to become the cheapest energy source in most countries in 2027.^49^ With a low technical threshold and cost, distributed solar power could effectively accelerate the progress of SDG 7, which should be considered an effective pathway to promote sustainable development in these countries.

### Future opportunities enlighten by integrating SDGs progress evenness

Although the uneven progress across SDGs observed in this study brings many challenges for global sustainable development, it also presents opportunities to enhance regional cooperation. Our findings demonstrate that many HICs are facing a stagnant development of SDGs 12 and 13, which may be related to tradeoffs between climate action and other goals. To solve these problems, a sharp acceleration in clean energy innovation is essential, although this may be a very time-consuming endeavor.^50^ There is also the possibility of engaging in other natural-based actions such as natural climate solutions^51^; although crucial, these efforts are not sufficient to tackle all of the related problems.^52^ In this case, regional collaborations could effectively complement existing actions.^52^ Considering that LICs and middle-income countries generally performed well on SDGs 12 (responsible consumption and production) and 13 (climate action) but poorly on SDGs related to essential human needs or economic development, we argue that regional collaborations between LICs, middle-income countries, and HICs related to climate action (e.g., collaborations through carbon trade between Europe and Africa or North America and Latin America) could be mutually beneficial. Notably, LICs should aim at acquiring the infrastructure and technology conducive to development and sustainable livelihoods, rather than simply fulfilling basic needs (e.g., food, water, power).^1^^,^^53^ For example, LICs could focus on acquiring clean energy technology, as well as agricultural infrastructure and technology. If LICs can successfully cooperate with other countries to acquire such technologies, they may be able to more autonomously improve their performance regarding essential human needs and then sustain these betterments. These actions are particularly important for LICs because their progress toward SDGs has been primarily uneven.

Meanwhile, middle-income countries may need to focus on technical and industrial innovation, because this may enable them to undertake a more effective development pathway without incurring environmental costs, as well as help them step over the middle-income trap. In addition, regional collaborations could also promote the stability of suitable development across countries. For instance, Germany exported medical supplies and received critically ill patients from neighboring European countries, relieving the burden on the healthcare system of many of these countries and reducing their own pandemic-induced economic losses. On the contrary, the setbacks in SDGs performances in Latin America and the Caribbean may be related to the lack of or reduced regional collaborations caused by COVID-19. Therefore, revitalizing global partnerships is an urgent matter for supporting SDGs performance worldwide, and future research should focus on identifying various solutions for such revitalization.

## Conclusion

By incorporating SDGs progress evenness into the analyses, this study delivers a detailed evaluation of the development pathways and progress toward SDGs across regions worldwide (see Table S7 for comparisons with other assessments), making this the most comprehensive assessment of global sustainable development to date and to the best of our knowledge. In our findings, an uneven progress across SDGs was associated with problems such as public health risks, ecosystem destruction, and social inequality, which are also identified as major global challenges by the United Nations Environment Programme.^54^ These findings point to a possible dilemma related to priority development, because environmental pollution and other problems associated with an uneven progress across SDGs may, in turn, compromise human health and well-being. Nevertheless, the uneven development pathways may sometimes be unavoidable or even efficient for many countries because of their specific resource endowments or social backgrounds. Therefore, to guide future policy making toward SDGs, future studies should focus on exploring the tipping points, mechanisms, and pathways linking uneven progress across SDGs with economic development, environmental pollution, public health, and social equality. Overall, we argue that SDGs progress evenness represents a novel and far-reaching equality issue in human development. Its relationship with the widely used mean SDG index score seems to be similar to that between average income and the wealth gap—although a high average income value is good, a high average income value accompanied by low inequality is better. The integration of SDGs progress evenness and MIS into a single index holds potential to reduce the overestimation of SDGs performance when large progress differences exist across goals. Therefore, future studies and assessments of global sustainable development are suggested to consider both indices.

Our findings also emphasize that the major challenges of LICs and countries with extremely harsh climate conditions is to acquire and secure essential needs for human survival, such as food, water, and medicine. However, HICs should instead strive toward achieving a more even development pathway toward SDGs by paying more attention to biodiversity conservation and climate action. Given that LICs and countries with extremely harsh climate conditions generally performed better on SDGs 12 and 13 than did HICs, we argue that global collaborations over climate action may provide opportunities for solving the aforementioned problems in a mutually beneficial way. The integration of the suggestions provided in this study is expected to help narrow down the uneven progress across SDGs worldwide and support the holistic achievement of sustainable development. Thus, they should be implemented into the global roadmap toward SDGs.

## Lead contact website

Yanfen Wang https://people.ucas.edu.cn/∼Wang.Yanfen.

## Supplemental information

Document S1. Materials and methods, Figures S1–S11, and Tables S1–S7

Supplementary Data.xlsx

Document S2. Article plus supplemental information
