# Supplementary material for: Overlooked uneven progress across sustainable development goals at the global scale: Challenges and opportunities
Source: Innovation (Camb). 2024 Jan 8;5(2):100573. doi: 10.1016/j.xinn.2024.100573 (PMC10876912; doi:10.1016/j.xinn.2024.100573)
Supplement: Document S1. Materials and methods, Figures S1–S11, and Tables S1–S7 [file mmc1.pdf]

The Innovation, Volume *5*

Supplemental Information

Overlooked uneven progress across sustainable development goals at the global scale: Challenges and opportunities

Yali Liu, Jianqing Du, Yanfen Wang, Xiaoyong Cui, Jichang Dong, Pan Gu, Yanbin Hao, Kai Xue, Hongbo Duan, Anquan Xia, Yi Hu, Zhi Dong, Bingfang Wu, Jürgen P. Kropp, and Bojie Fu

MATERIALS AND METHODS

Data sources

All SDG scores (the score for each SDG) and SDG index scores (the average of all 17 SDG scores) over time and space were based on SDG Index and Dashboards Reports from 2017 to 2021.[1-5] Briefly, SDG Index and Dashboards Reports adopt around 100 indicators from a mix of official and non-official data sources, generally following the official SDG indicators endorsed by the UN Statistical Commission. The value for each indicator is normalized to 0-100 by range transformation with carefully established upper and lower bounds for each indicator. The score for each SDG is calculated using the arithmetic mean of all corresponding SDG indicators' normalized values. These scores across all 17 SDGs are later averaged to calculate the SDG Index score (the mean SDG index score in the present study, MIS). More details about indicator selection, data source, normalization, calculations, and sensitivity tests can be found in those reports (https://www.sdgindex.org/reports/).

In SDG Index and Dashboards Reports and other well-established SDGs assessments,[4,6] the quantification of progress toward SDGs across countries and regions was mainly based on the SDG index score, which might be biased and over-optimistic when a large difference exists across 17 SDGs.[7] Therefore, the present study further quantified the progress evenness across all 17 SDG scores by the SDGs progress evenness score (ES) to diminish the potential overestimation of sustainable development performance by adopting the SDG index score only, then computed the sustainable development score (SDS) integrating both ES and MIS to quantify the progress toward SDGs at the global scale comprehensively. We further compared the SDGs progress evenness before and after the outbreak of COVID-19 based on the SDG Reports 2020 and 2021, respectively. The raw data used in the SDG report was mainly before the year the report was published (mostly in June) due to the lagging update of statistical data. Therefore, the SDG Report 2021, published in June 2021, reflected the first evaluation of global sustainable development post-COVID-19.

SDGs progress evenness score and sustainable development score

The computation of the evenness score and sustainable development score generally follows Liu *et al.*[7] with an improved radar chart method.[8] More details are quoted as follows:

The score of each SDG for a given country or region in a specific year is used as the radius of each sector, forming a radar chart with 17 sectors corresponding to the 17 SDGs. The area (S) and perimeter (L) of the radar chart are expressed as follows (Fig. S10):

$$S_i = \sum_{j=1}^{n} S_j = \sum_{j=1}^{n} \pi f_j {r_j}^2 \quad j = 1,2,\cdots,n \tag{1}$$

$$L_i = \sum_{j=1}^{n} L_j = 2|r_{max} - r_{min}| + \sum_{j=1}^{n} 2\pi f_j r_j \quad j = 1,2,\cdots,n \tag{2}$$

where n represents the number of SDGs, which is 17; r_{max} and r_{min} represent the maximum and minimum among 17 SDG scores, respectively; r_j refers to the score of the jth SDG; and f_j stands for the weight of the jth SDG and is set as 1/17 for all SDGs because there is no reason to give one SDG greater weight than another in the SDGs assessment, which focuses on the current SDGs progress status rather than future policy-making.[4,6,9] The evenness score refers to the ratio between the total area of the radar chart formed by the 17 SDGs and the area of a circle with the same perimeter (the evenest distribution with the same perimeter), which is calculated by S_i and L_i following equation (3). It is re-scaled to 0-100 by multiplying 100 to be comparable with the SDG index score. As the area of the radar chart with a given perimeter reaches the largest (100) when it is a circle and decreases with increasing uneven progress across all 17 SDGs, the evenness score (ES) is the highest when each SDG shares the same score. ES depicts the progress differences across the 17 SDGs, where a low value presents an uneven status or existing trade-offs among the SDGs.

$$ES = S_i/[\pi(L_i/2\pi)^2] \times 100 = 400\pi S_i/{L_i}^2 \tag{3}$$

The sustainable development score (SDS) is the geometric mean of the evenness score and the mean SDG index score, which diminishes the potential overestimation of sustainable development performance by using the mean SDG index score only in case there are large differences across 17 SDGs. All scores range from 0 (worst performance) to 100 (best performance).

Overall, evenness scores and sustainable development scores of 169 countries were calculated annually from 2017 to 2021. All countries were further grouped by geographic location, climate condition (by the aridity index), and economic status (by UN income groups in 2019). Aridity is chosen to represent the climate condition because 1) drylands are fragile ecosystems with limited ecosystem services and sensitive to climate changes and human activities, thus are among the most challenging regions to achieve SDGs,[10] and 2) dryland expansion is a representative outcome of the global climate change and is assumed to be accelerated in the future.[11,12] To be specific, six groups were defined by geographic location, i.e., Africa, Asia, Latin America and Caribbean (LAC), Oceania, Europe, and North America (North A.); four groups were divided by UN income groups in 2019,[3] namely, high-income countries (HIC), upper-middle-income countries (UMIC), lower-middle-income countries (LMIC), and lower-income countries (LIC); four groups were defined by the proportion of drylands in the total area of the national territory area,[13] namely, none or slightly arid (<50%), moderately arid (50-75%), severely arid (75-99%), extremely arid (>99%). Then, the differences in MIS, ES, and SDS among groups were explored.

Furthermore, the current development status for all countries was divided into four categories based on the quadrantal diagram (MIS vs. ES in 2021).[7] The threshold for relatively high and low MIS/ES was calculated based on the K-mean clustering analysis. With both thresholds, all countries were divided into four quadrants: 1) the first quadrant for relatively sustainably status with high MIS and ES; 2) the second quadrant for underdeveloped status with low MIS and high ES; 3) the third quadrant for underdeveloped and uneven status with low MIS and ES; 4) the fourth quadrant for uneven status with high MIS and low ES.

Effect of SDGs progress evenness on social and environmental indicators

The economy is strongly associated with achieving SDGs, and the top ten countries approaching SDGs are all HIC.[4] Therefore, SDGs progress evenness and GDP per capita were set as independent variables in the binary regression model to distinguish their partial effects on indicators related to human health, inequality, and the environment. Furthermore, we used path analysis to explore the direct and indirect (via $PM_{2.5}$, ratio of female-to-male mean year of education received, Gini coefficient, etc.) effects of SDGs progress evenness and GDP per capita on indicators related to public health, as public health could also be affected by social and environmental factors. The data for the indicators were from multiple sources found in the attached Supplementary Data file.

Development pathway and the effective development score

The development pathway and the effective development score were adopted to evaluate the progress toward SDGs over time. Generally, we followed the method of Liu *et al.*[7] in defining and computing the development pathway and the effective development score. The development pathway of a given country from 2017 to 2021 can be visualized by creating a vector within MIS-ES coordinates, starting with the pair-wise MIS (x_1) and ES (y_1) in 2017 and ending with the paired values in 2021 (x_2, y_2). As a result, four types of pathways are expected (namely, progress in both MIS and ES, retrogression in either MIS or ES, and retrogression in both MIS and ES). The ideal pathway was the vector with a slope of one, characterizing a simultaneous improvement in all 17 SDGs. The effective development score (EDS) refers to the projected length (red line in Fig. S11) of a given pathway (red vector) on the ideal pathway. EDS provides a more comprehensive estimation of progress toward SDGs over time than only interpreting the change of MIS or SDS. It can help to diminish the overestimation if the achievement is either the increase in mean SDG index score only (improvements only shown in a few SDGs) or the increase in evenness score only (improvement in the poorly achieved SDGs while retrogression in the better-accomplished SDGs).

$$EDS = \begin{cases} \cos(\theta - 45°) \times \sqrt{(x_1 - x_2)^2 + (y_1 - y_2)^2}\ , & -180° \le \theta < -90° \\ \cos(45° - \theta) \times \sqrt{(x_1 - x_2)^2 + (y_1 - y_2)^2}\ , & -90° \le \theta < 45° \\ \cos(\theta - 45°) \times \sqrt{(x_1 - x_2)^2 + (y_1 - y_2)^2}\ , & 45° \le \theta < 180° \end{cases} \tag{4}$$

Projections of SDGs performance in 2030

Both MIS and SDS in 2030 were predicted based on curvilinear regressions and the gray forecast model using MIS or SDS in each country from 2017 to 2021. Eleven regression models, including linear, quadratic, cubic, compound, growth, logarithmic, S, exponential, inverse, power function, and Logistic models, were used in our analyses. The best model was determined based on the Akaike information criterion (AIC). All analyses were performed with SPSS v. 27 (IBM Corp., Armonk, NY, USA). The grey forecast model was performed with MATLAB R2020b software. The data matrix was constructed by the adjacent mean value and the parameters were obtained by the least square method. We compared the projections of two prediction methods in 2030 and used the average values of the two methods in Figure 4.

Urgency and potential for priority development

The present study proposed a framework for the priority selection by integrating the SDGs progress evenness:

1) All SDGs were divided into four categories generally based on Fu *et al.*,[14] namely, essential human needs (poverty, food, health, water, and energy) (SDG 1, 2, 3, 6, 7), eco-environmental protection (SDG 12, 13, 14, 15), social development (SDG 4, 5, 10, 16, 17), and economic development (SDG 8, 9, 11).

2) The performance for each category was evaluated by the average SDG score of all SDGs included in 2021. Countries with relatively poor performance on essential human needs (lower than 61.33 based on the K-mean clustering analysis) were considered urgent for priority development.

3) Among these countries, whose current status was uneven were chosen for further discussion as the priority development might amplify the uneven progress across SDGs and bring expected problems. A country with the worst performance on eco-environmental protection was considered to have a low potential for priority development.

4) Development pathways were then considered for those unevenly progressed countries with relatively better performance on eco-environmental protection. Countries with an even pathway were treated differently from those with an uneven pathway.

5) For those with an even pathway, if the average score for essential human needs of a given country increased during the study period, it was considered less urgent for priority developments, while countries with decreasing average score might need to consider an adjustment in their development by addressing the essential human needs.

6) The most urgent need for priority development was identified as the countries with an uneven pathway, which reflected in a decreasing average score for essential human needs, particularly the countries currently performing the worst on essential human needs.

Other statistical analyses

The statistical differences in MIS, ES, SDS, and EDS among groups were based on one-way ANOVA, and *p* values between groups were based on the least significant differences (LSD) test. The normal distribution test was performed first, while data that failed to pass the test was transformed by square root (MIS and SDS were moderately negatively skewed in this study). The present study also explored the synergies and trade-offs among all SDGs within different geographic/environmental/economic groups. Based on the Pearson correlation matrix calculated from scores of all SDGs across countries in 2021, synergies and trade-offs between two SDGs refer to those significant positive or negative correlations at $p < 0.05$, while those non-significant relationships are defined as uncorrelated.[15] The number of synergies, trade-offs, and uncorrelated relationships between a given

SDG and the other SDGs were recorded and compared to show how it interacts with other SDGs. All statistical analyses were performed with SPSS v. 27 (IBM Corp., Armonk, NY, USA).

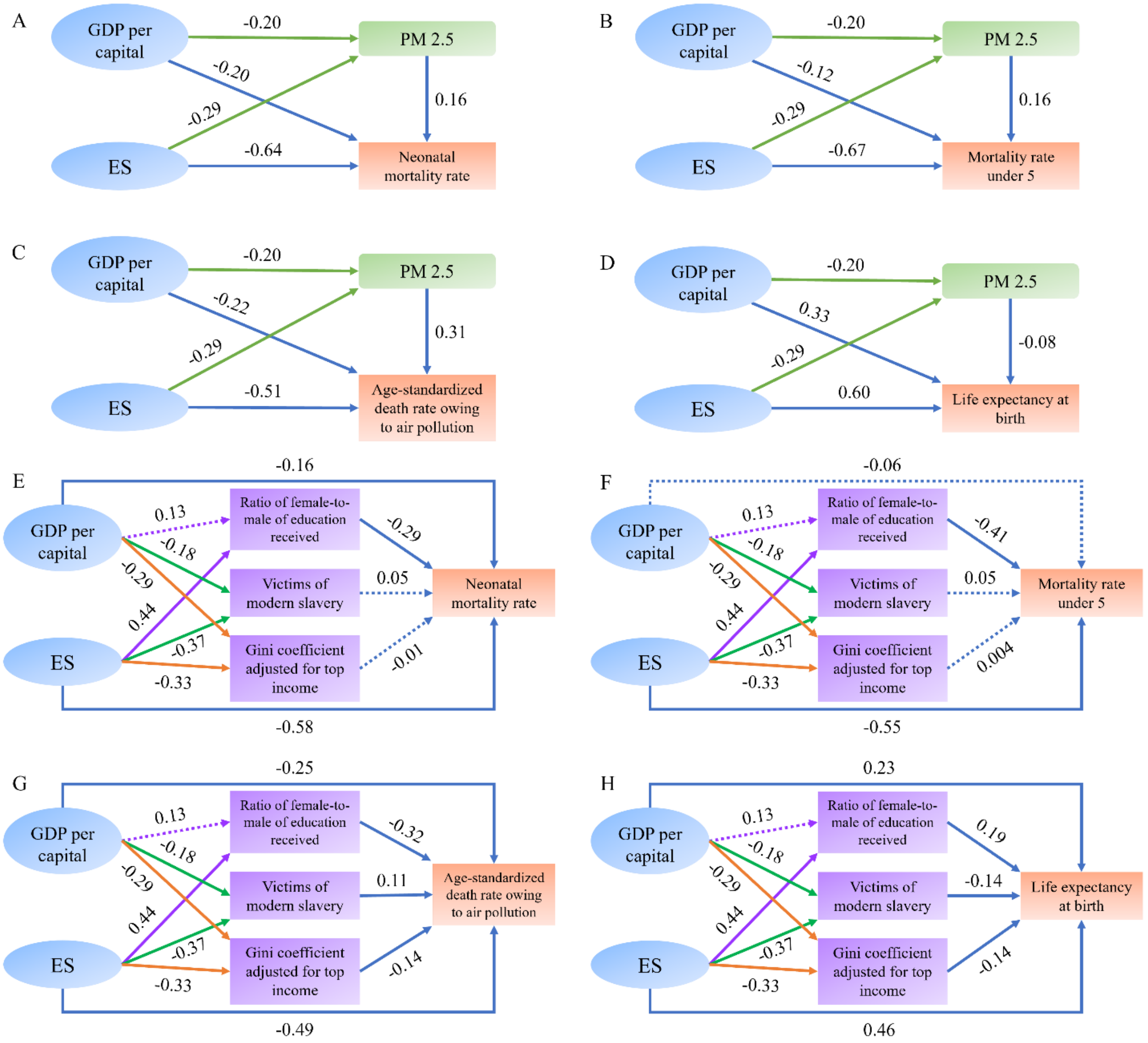

Fig. S1. Path analysis of the impact of GDP per capita and SDGs progress evenness (ES) on health-related indicators.

Solid and dashed lines represent significant and non-significant paths, respectively.

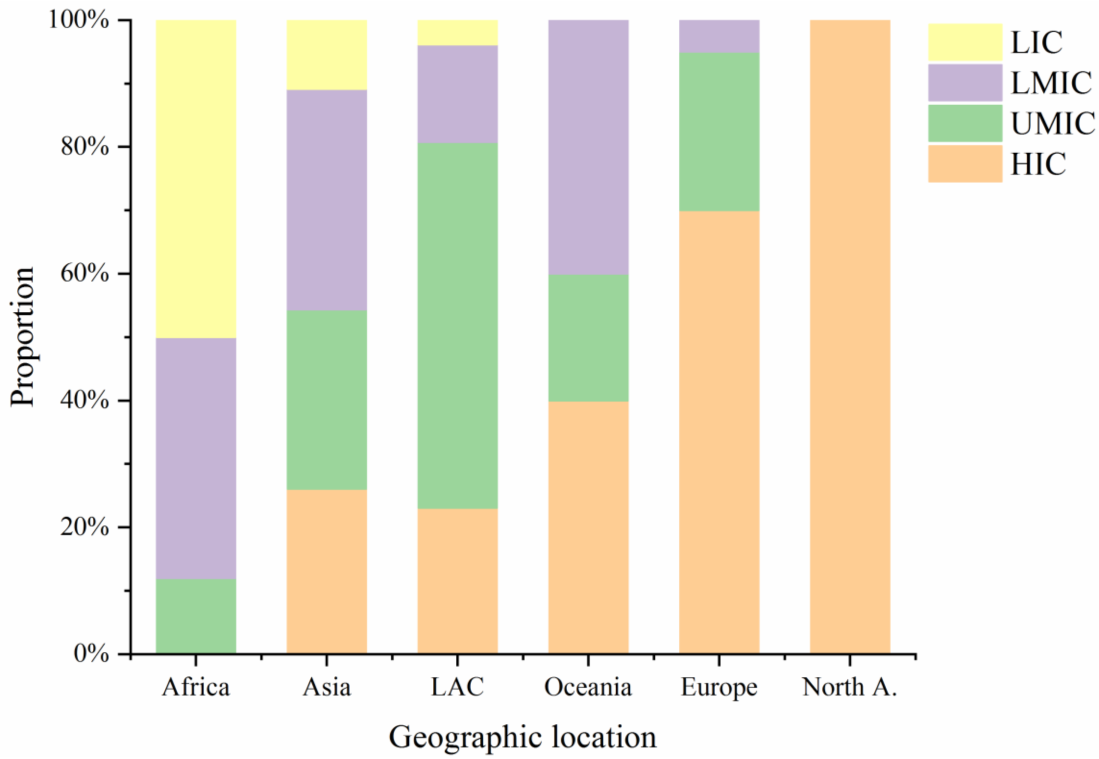

Fig. S2. The proportion of UN income groups in different regions.

HIC, UMIC, LMIC, and LIC stand for high-income, upper-middle-income, lower-middle-income, and lower-income countries, respectively. North A. is short for North America. LAC refers to Latin America and Caribbean.

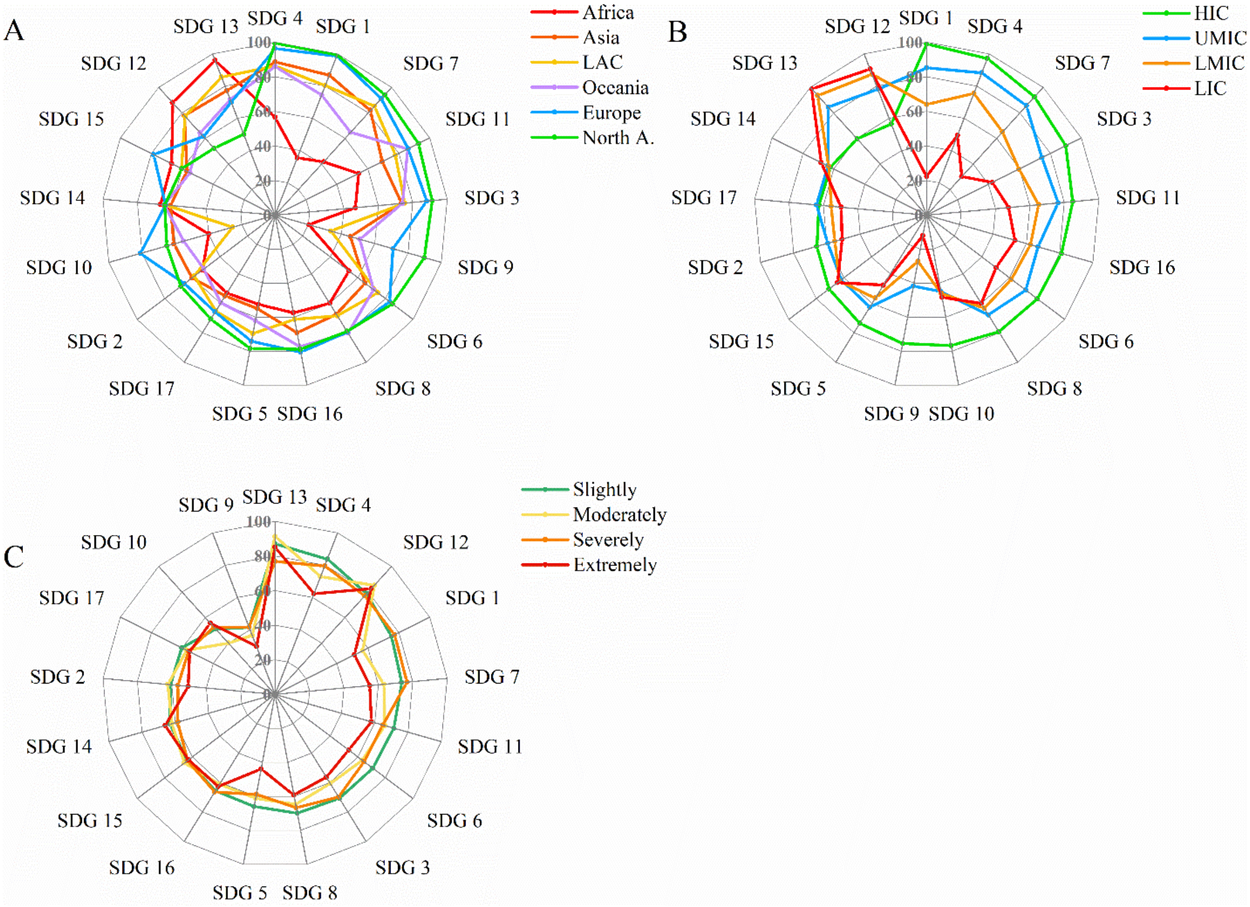

Fig. S3. Average SDG scores across different geographic locations (A), UN income groups (B), and arid levels (C) in 2021.The proportion of UN income groups in different regions.

The radar chart starts from the north with the SDG with the highest score of North America (in A), high-income countries (in B), and slightly arid countries (in C), respectively. North A. is short for North America. LAC refers to Latin America and Caribbean. HIC, UMIC, LMIC, and LIC stand for high-income, upper-middle-income, lower-middle-income, and lower-income countries, respectively.

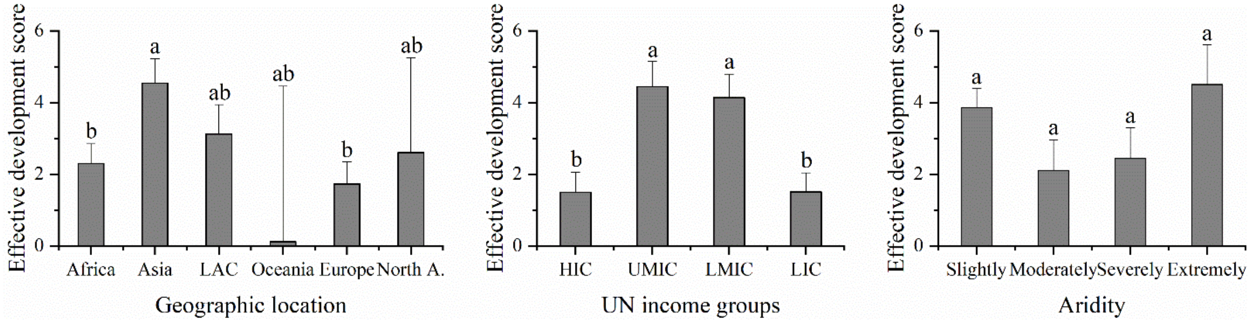

Fig. S4. Differences in effective development score across geographic locations, UN income groups, and arid levels in 2020. HIC, UMIC, LMIC, and LIC stand for high-income, upper-middle-income, lower-middle-income, and lower-income countries, respectively. North A. is short for North America. LAC refers to Latin America and Caribbean. The histogram with error bars presents the mean value ± standard error (SE). Letters a and b visualize the significant differences at $p < 0.05$. EDS is based on data from 2017 to 2020.

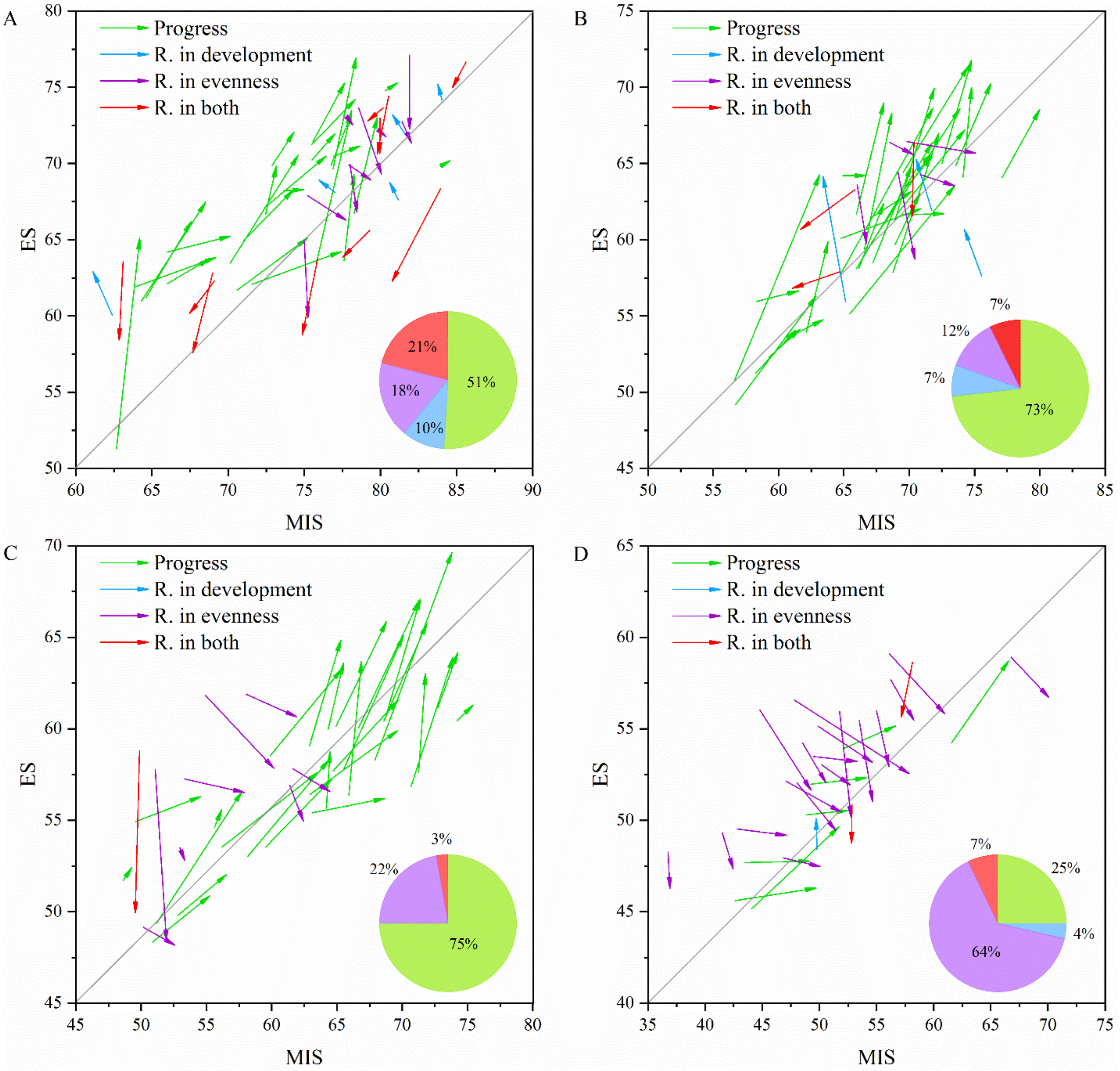

Fig. S5. Development pathways for different UN income groups from 2017 to 2020.

(A) high-income countries; (B) upper-middle-income countries; (C) lower-middle-income countries; (D) low-income countries. Different colors are used to represent the four types of pathways: progress (green), retrogression in development (R. in development, blue), retrogression in evenness (R. in evenness, purple), and retrogression in both (R. in both, red). The grey diagonal stands for the ideal pathway (slope = 1). The pie chart at the bottom right corner shows the proportion of different pathways.

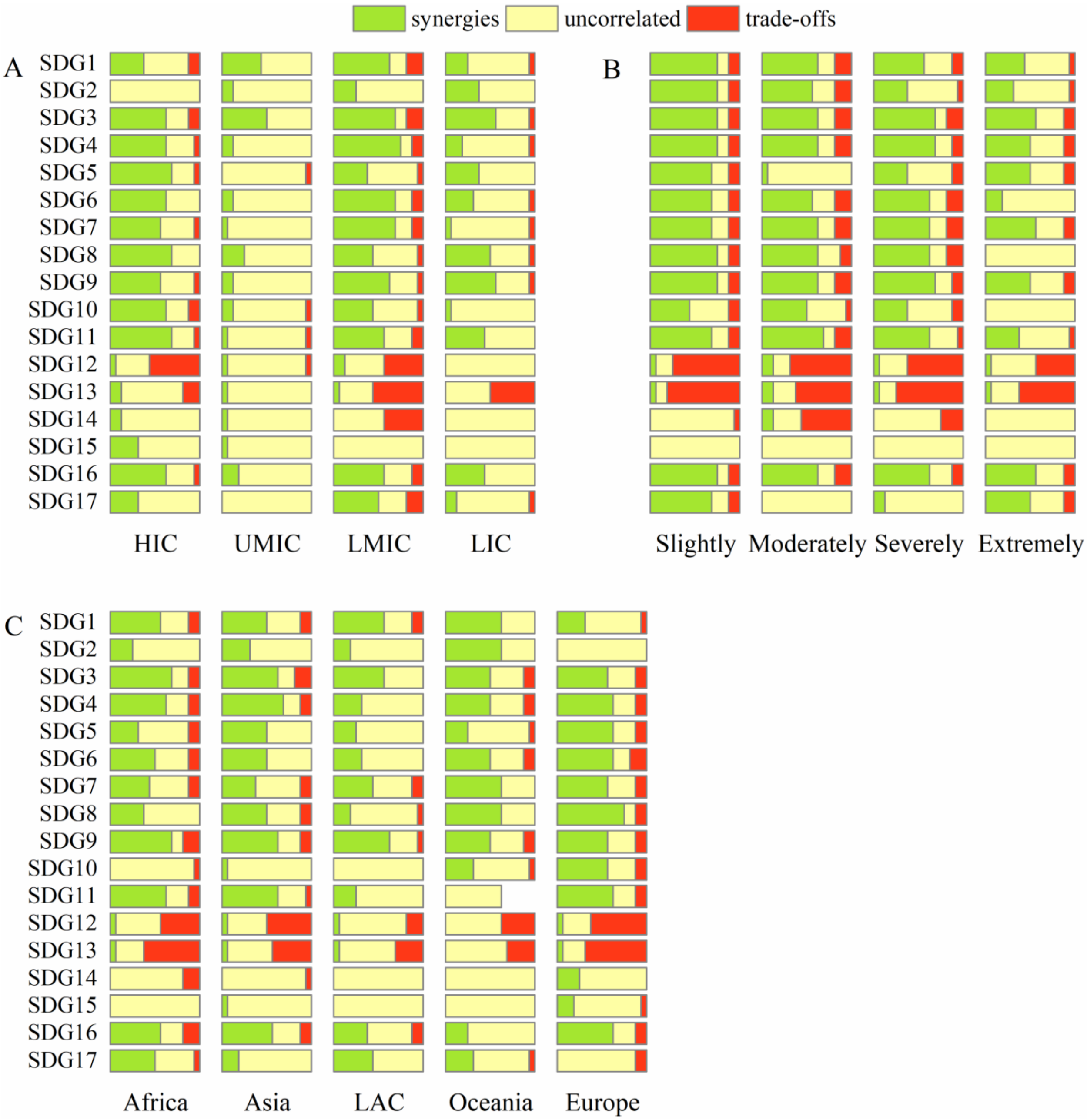

Fig. S6. Interactions among SDGs across different UN income groups (A), arid levels (B), and geographic locations (C) in 2021.

Green represents synergies with a significantly positive correlation at $p < 0.05$; red represents trade-offs with a significantly negative correlation at $p < 0.05$; and yellow represents non-significant correlations. HIC, UMIC, LMIC, and LIC stand for high-income, upper-middle-income, lower-middle-income, and lower-income countries, respectively. LAC refers to Latin America and Caribbean. North America is not involved in this analysis because only two countries exist.

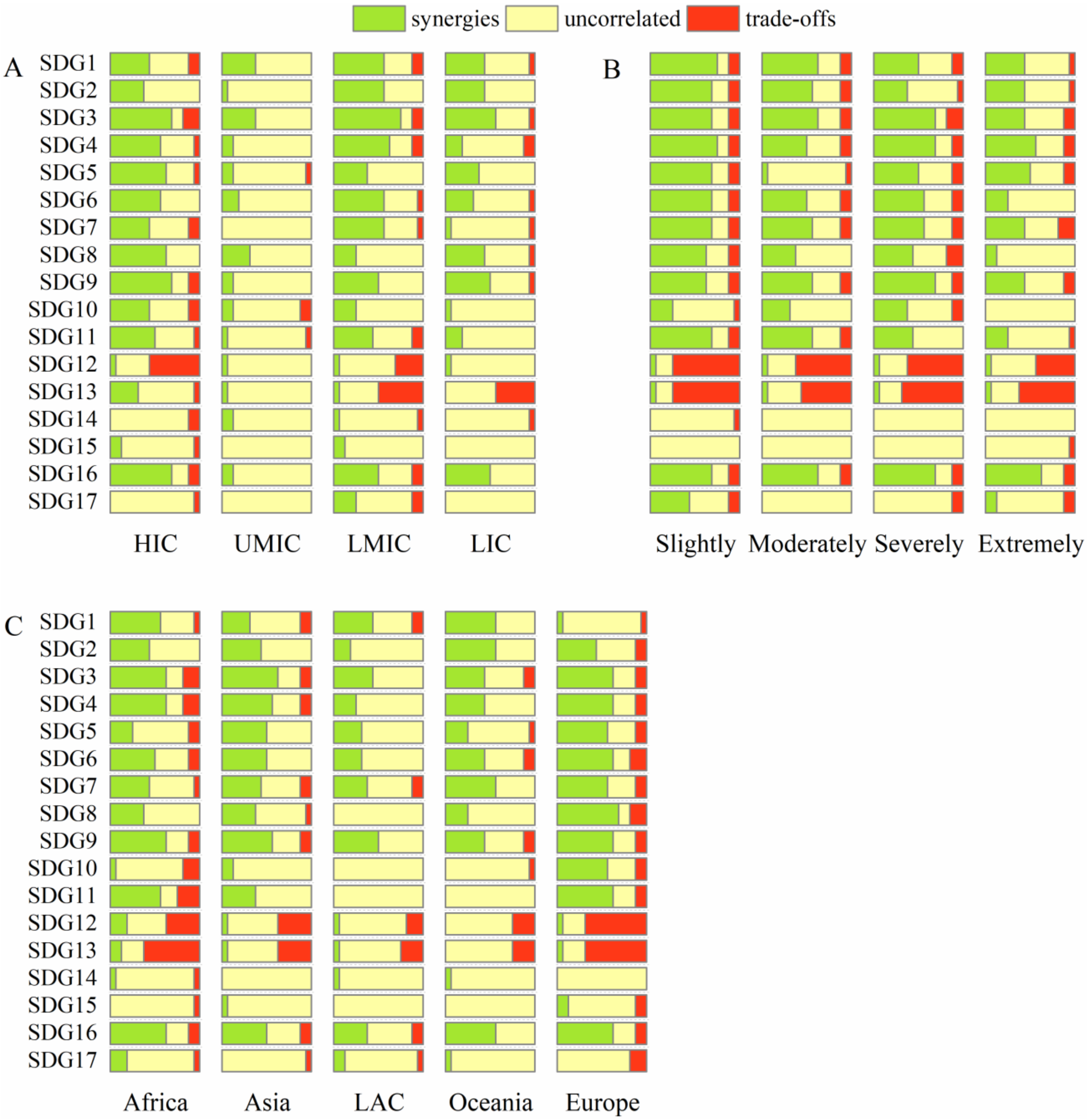

Fig. S7. Interactions among SDGs across different UN income groups (A), arid levels (B), and geographic locations (C) in 2020.

Green represents synergies with a significantly positive correlation at $p < 0.05$; red represents trade-offs with a significantly negative correlation at $p < 0.05$; and yellow represents non-significant correlations. HIC, UMIC, LMIC, and LIC stand for high-income, upper-middle-income, lower-middle-income, and lower-income countries, respectively. LAC refers to Latin America and Caribbean. North America is not involved in this analysis because only two countries exist.

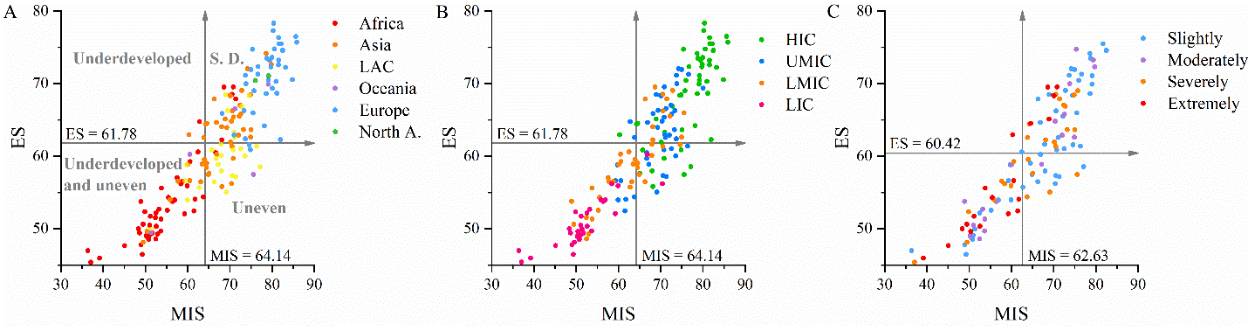

Fig. S8. Sustainable development status for countries across different geographic locations (A), UN income groups (B), and arid levels (C) in 2021.

HIC, UMIC, LMIC, and LIC stand for high-income, upper-middle-income, lower-middle-income, and lower-income countries, respectively. LAC refers to Latin America and Caribbean; North A. stands for North America. S. D. refers to the relatively sustainably developed status.

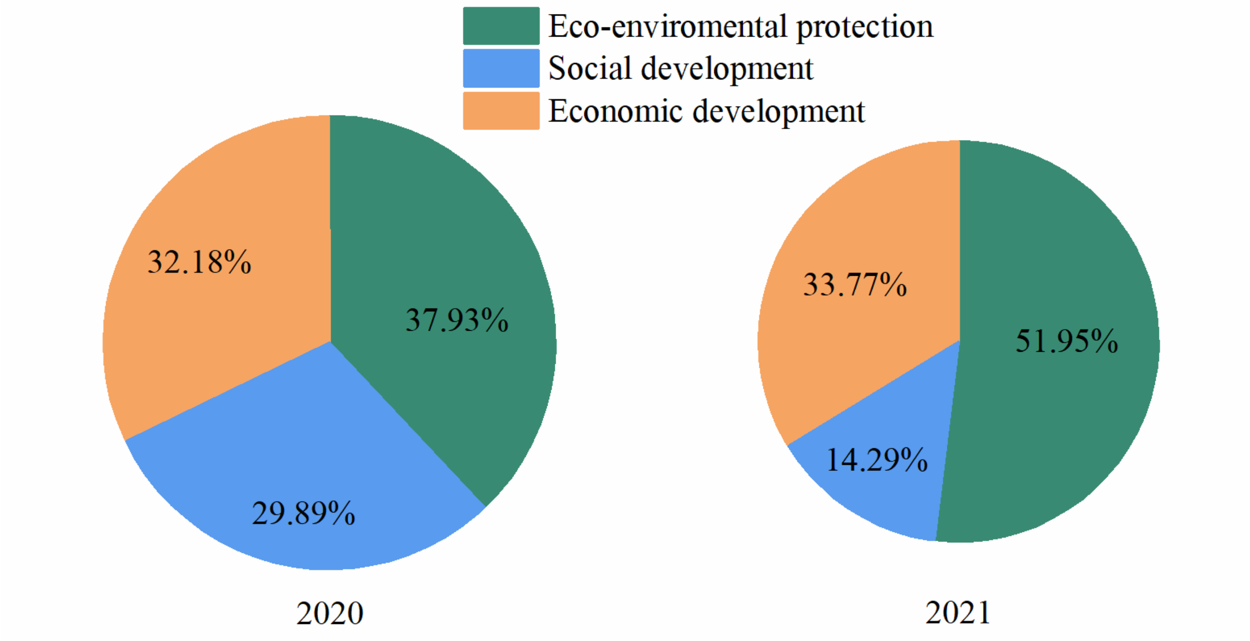

Fig. S9. Proportion of deficiencies in countries considered relatively sustainably developed in 2020 and 2021.

87 and 77 countries are considered relatively sustainably developed in 2020 and 2021, respectively.

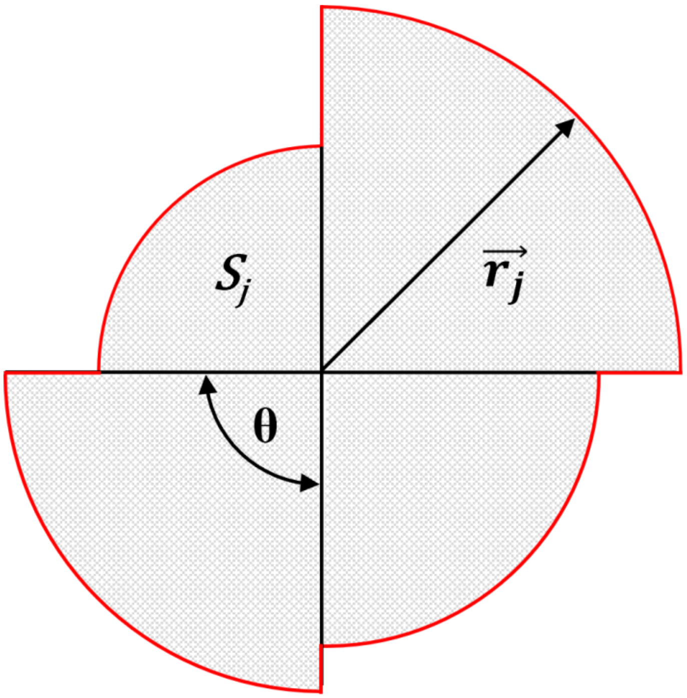

Fig. S10. Outline of the radar chart method.

r_j stands for the score of the jth SDG. θ represents the weight of each SDG, which refers to f_j in equations (1) and (2), and is set up to 1/17 for all SDGs in the present study. The total area of all sectors refers to S_i in equation (1). The red line presents the total perimeter of all sectors, referring to L_i in equation (2).

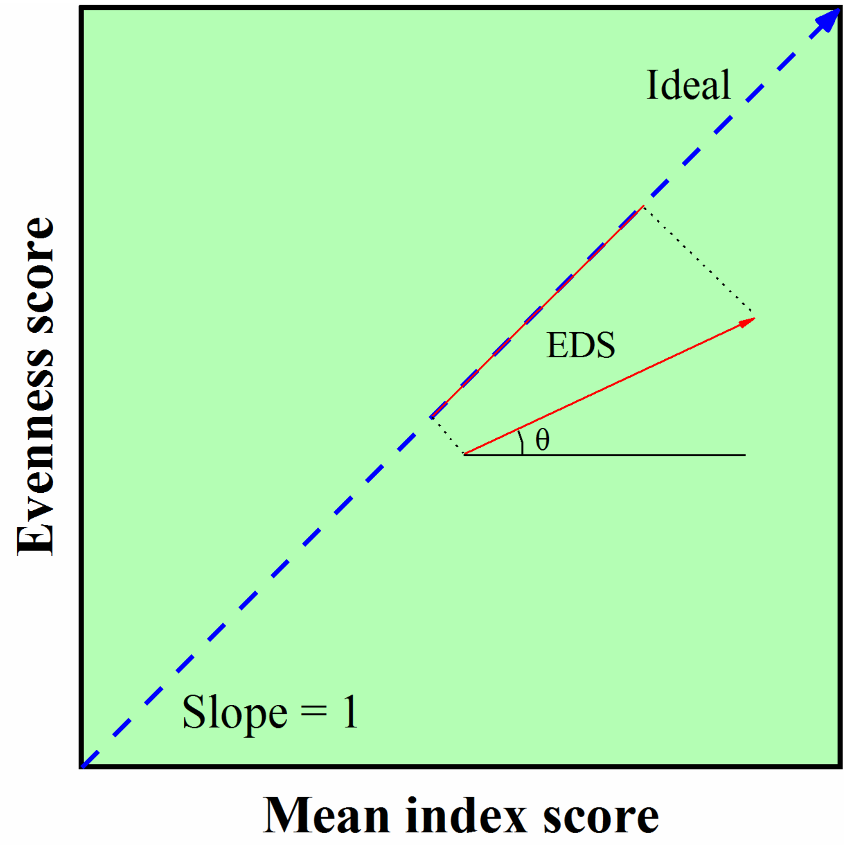

Fig. S11. Outline of the development pathway and the effective development score (EDS)[7].

θ is the angle between a given vector (marked in red) and the x-axis.

Table S1. Effects of GDP per capita and the annual mean concentration of particulate matter of less than 2.5 microns in diameter ($PM_{2.5}$) on the indicators related to health.

Factor	Neonatal mortality rate (per 1000 live births) R^2 = 0.39, $F_{2,157}$ = 51.55			Mortality rate under 5 (per 1000 live births) R^2 = 0.32, $F_{2,157}$ = 39.04			Age-standardized death rate due to air pollution (per 100,000 population) R^2 = 0.49, $F_{2,157}$ = 77.18			Life expectancy at birth (years) R^2 = 0.45, $F_{2,157}$ = 65.98			VIF
	β	t	p	β	t	p	β	t	p	β	t	p	
GDP per capita	-0.45	-6.90	<0.001	-0.39	-5.57	<0.001	-0.43	-7.15	<0.001	0.57	9.10	<0.001	1.125
$PM_{2.5}$	0.31	4.72	<0.001	0.32	4.61	<0.001	0.43	7.19	<0.001	-0.22	-3.58	<0.001	

All models are significant at $p < 0.05$. VIF: variance inflation factor.

Table S2. Raw data for the mean index score (MIS), evenness score (ES), sustainable development score (SDS), and effective development score (EDS) across geographic locations, UN income groups, and arid levels. HIC, UMIC, LMIC, and LIC stand for high-income, upper-middle-income, lower-middle-income, and lower-income countries, respectively. LAC refers to Latin America and Caribbean. N.A. is short for not available.

Countries	UN regions	Income Group in 2019	Dryland area (%)	2017			2018			2019			2020			2021			EDS	EDS
				MIS	ES	SDS	MIS	ES	SDS	MIS	ES	SDS	MIS	ES	SDS	MIS	ES	SDS	2017-2020	2017-2021
Afghanistan	Asia	LIC	88.86	46.81	47.95	47.38	46.24	49.06	47.63	45.73	47.24	46.48	50.09	47.47	48.76	49.55	48.13	48.83	1.98	2.06
Albania	Europe	UMIC	N. A.	68.90	59.66	64.12	68.91	60.11	64.36	70.27	62.46	66.25	70.82	63.06	66.83	71.02	62.91	66.85	3.76	3.80
Algeria	Africa	UMIC	99.16	68.76	57.86	63.08	67.88	60.34	64.00	71.10	62.76	66.80	72.27	66.97	69.57	70.86	69.54	70.20	8.92	9.74
Angola	Africa	LMIC	52.79	50.18	49.15	49.66	49.56	49.47	49.52	51.32	47.88	49.57	52.59	48.18	50.34	50.30	49.06	49.68	1.01	0.02
Argentina	LAC	HIC	79.83	72.45	66.68	69.50	70.28	65.78	67.99	72.35	68.10	70.19	73.17	69.82	71.47	72.80	66.88	69.78	2.73	0.39
Armenia	Asia	UMIC	62.06	71.72	61.93	66.65	69.27	61.34	65.18	70.53	65.11	67.77	70.54	65.26	67.85	72.60	65.79	69.11	1.52	3.35
Australia	Oceania	HIC	91.08	75.87	63.74	69.54	72.89	60.84	66.59	73.89	65.58	69.61	74.87	58.79	66.34	75.58	57.46	65.90	-4.21	-4.66
Austria	Europe	HIC	N. A.	81.42	72.74	76.96	79.95	72.42	76.09	82.93	70.55	76.49	82.03	71.36	76.51	83.25	72.23	77.54	-0.54	0.93
Azerbaijan	Asia	UMIC	83.13	70.81	64.28	67.47	70.80	64.14	67.38	70.46	59.10	64.53	73.46	63.54	68.32	73.25	63.64	68.28	1.36	1.28
Bahrain	Asia	HIC	84.88	64.59	61.19	62.87	65.90	61.46	63.64	66.98	68.68	67.82	67.58	66.20	66.88	64.30	64.40	64.35	5.65	2.06
Bangladesh	Asia	LMIC	N. A.	56.21	53.54	54.86	59.35	54.14	56.68	60.88	54.22	57.45	63.51	57.60	60.48	63.45	58.90	61.13	8.03	8.91
Barbados	LAC	HIC	N. A.	65.98	64.20	65.08	N. A.	N. A.	N. A.	N. A.	N. A.	N. A.	70.15	65.24	67.65	70.98	63.29	67.03	3.69	2.90
Belarus	Europe	UMIC	N. A.	77.12	64.10	70.31	75.99	63.60	69.52	79.74	67.06	73.13	80.00	68.55	74.05	80.07	69.37	74.53	5.18	5.81
Belgium	Europe	HIC	N. A.	79.96	72.97	76.38	79.00	70.28	74.51	78.89	64.22	71.18	79.96	70.65	75.16	82.19	75.48	78.76	-1.64	3.35
Belize	LAC	UMIC	N. A.	66.02	63.61	64.80	62.32	60.07	61.19	64.14	61.61	62.86	66.74	59.77	63.16	66.71	59.68	63.10	-2.21	-2.29
Benin	Africa	LIC	94.55	49.47	53.48	51.44	48.98	51.59	50.27	50.85	51.07	50.96	53.31	53.22	53.26	49.87	52.34	51.09	2.53	-0.52
Bhutan	Asia	LMIC	25.9	65.51	57.78	61.52	65.39	59.43	62.34	68.90	61.06	64.86	70.08	65.08	67.54	70.56	66.23	68.36	8.40	9.55
Bolivia	LAC	LMIC	47.71	64.69	57.69	61.09	68.08	58.71	63.22	69.31	60.52	64.76	69.70	61.56	65.50	67.90	61.64	64.69	6.28	5.06
Bosnia and Herzegovina	Europe	UMIC	N. A.	65.45	55.13	60.07	67.31	55.37	61.05	69.39	55.87	62.26	73.48	63.54	68.33	73.70	62.40	67.81	11.62	10.97
Botswana	Africa	UMIC	99.99	58.26	51.26	54.65	58.46	53.01	55.67	60.09	52.57	56.21	61.27	54.05	57.55	61.54	52.46	56.82	4.10	3.17
Brazil	LAC	UMIC	13.99	69.51	61.63	65.45	69.69	60.43	64.89	70.62	61.61	65.96	72.67	61.72	66.97	71.34	57.17	63.86	2.30	-1.86
Brunei Darussalam	Asia	HIC	N. A.	N. A.	N. A.	N. A.	N. A.	N. A.	N. A.	N. A.	N. A.	N. A.	67.84	55.19	61.19	68.16	55.80	61.67	N. A.	N. A.
Bulgaria	Europe	UMIC	57.75	72.51	65.01	68.66	73.13	65.31	69.11	74.52	67.43	70.88	74.77	71.77	73.26	73.81	69.99	71.88	6.38	4.44
Burkina Faso	Africa	LIC	99.99	49.90	55.13	52.45	50.88	52.54	51.70	52.27	53.90	53.08	54.64	53.16	53.90	52.60	50.35	51.46	1.96	-1.47
Burundi	Africa	LIC	17.54	51.77	55.94	53.81	49.82	53.56	51.66	51.36	50.25	50.80	52.77	50.17	51.45	50.79	48.54	49.65	-3.37	-5.92
Cabo Verde	Africa	LMIC	92.8	N. A.	N. A.	N. A.	64.68	63.38	64.02	65.05	61.51	63.26	67.18	60.80	63.91	68.14	62.26	65.13	N. A.	N. A.
Cambodia	Asia	LMIC	0.34	58.17	53.04	55.55	60.38	55.38	57.82	61.78	57.03	59.36	64.39	58.30	61.27	64.54	59.01	61.71	8.11	8.72
Cameroon	Africa	LMIC	14.12	52.83	49.80	51.29	55.78	50.38	53.01	56.02	51.39	53.66	56.54	52.03	54.24	55.26	52.53	53.88	4.20	3.65
Canada	North America	HIC	17.49	78.02	72.75	75.34	76.79	72.34	74.53	77.89	71.53	74.64	78.19	72.53	75.31	79.16	71.10	75.02	-0.03	-0.36
Central African Republic	Africa	LIC	29.84	36.75	48.27	42.12	37.66	49.05	42.98	38.11	50.13	43.71	36.92	46.28	41.34	36.41	46.97	41.35	-1.29	-1.16
Chad	Africa	LIC	99.83	41.50	49.30	45.23	42.81	48.62	45.62	42.05	46.89	44.41	42.46	47.34	44.83	39.21	45.93	42.44	-0.71	-4.00
Chile	LAC	HIC	48.43	71.57	62.08	66.65	72.79	61.51	66.91	75.61	62.47	68.73	77.42	64.23	70.52	77.13	58.53	67.19	5.66	1.43
China	Asia	UMIC	56.48	67.12	59.82	63.36	70.05	61.65	65.72	73.21	64.42	68.67	73.89	68.60	71.20	72.06	62.88	67.32	11.00	5.66
Colombia	LAC	UMIC	2.95	64.80	60.10	62.41	66.61	59.17	62.78	69.57	61.91	65.63	70.91	62.03	66.32	70.56	57.31	63.59	5.68	2.10
Comoros	Africa	LIC	N. A.	N. A.	N. A.	N. A.	N. A.	N. A.	N. A.	52.98	49.85	51.39	53.07	49.77	51.39	N. A.	N. A.	N. A.	N. A.	N. A.
Congo, Dem. Rep.	Africa	LIC	7.78	42.66	45.61	44.11	43.39	46.63	44.98	44.95	46.28	45.61	49.71	46.29	47.97	49.30	46.47	47.86	5.47	5.30
Congo, Rep.	Africa	LMIC	N. A.	50.88	48.34	49.60	52.38	52.41	52.40	54.22	52.35	53.28	55.25	50.87	53.02	52.95	49.25	51.07	4.88	2.10
Costa Rica	LAC	UMIC	N. A.	69.81	66.44	68.10	73.15	64.54	68.71	74.98	65.64	70.15	75.08	65.69	70.23	73.55	60.02	66.44	3.19	-1.89
Cote d'Ivoire	Africa	LMIC	N. A.	53.34	57.25	55.26	55.18	55.97	55.57	55.70	56.82	56.26	57.91	56.52	57.21	57.56	57.04	57.30	2.72	2.84
Croatia	Europe	HIC	N. A.	76.88	69.67	73.19	76.52	70.91	73.66	77.79	70.66	74.14	78.40	76.94	77.67	80.38	78.33	79.35	6.22	8.60
Cuba	LAC	UMIC	6.8	75.55	57.63	65.99	71.34	56.89	63.71	72.37	56.65	64.03	74.23	60.67	67.11	76.36	61.37	68.46	1.21	3.21
Cyprus	Asia	HIC	89.14	70.60	61.69	66.00	70.36	60.18	65.07	70.14	61.65	65.76	75.21	65.11	69.98	74.87	68.44	71.58	5.68	7.79
Czech Republic	Europe	HIC	2.56	81.90	77.10	79.46	78.72	71.25	74.89	82.57	74.96	78.67	81.90	72.27	76.93	82.51	74.59	78.45	-3.42	-1.34
Denmark	Europe	HIC	N. A.	84.16	69.96	76.73	84.61	73.37	78.79	85.22	72.83	78.78	84.56	70.19	77.04	84.86	70.54	77.37	0.44	0.90
Djibouti	Africa	LMIC	99.84	49.59	54.93	52.19	50.63	54.00	52.29	51.36	53.42	52.38	54.56	56.29	55.42	53.76	55.58	54.67	4.48	3.41
Dominican	LAC	UMIC	22.56	67.22	58.48	62.70	66.42	59.87	63.06	69.76	62.68	66.13	70.17	64.82	67.44	70.76	60.87	65.63	6.57	4.19

Republic																				
Ecuador	LAC	UMIC	18.32	69.04	61.66	65.25	70.77	62.73	66.63	72.29	63.91	67.97	74.26	67.21	70.65	72.54	61.02	66.53	7.61	2.01
Egypt, Arab Rep.	Africa	LMIC	99.8	64.92	60.13	62.48	63.47	61.24	62.35	66.21	62.08	64.11	68.79	65.86	67.31	68.65	69.50	69.07	6.79	9.26
El Salvador	LAC	LMIC	N. A.	62.92	56.41	59.58	64.09	56.06	59.94	66.73	60.48	63.53	69.62	59.89	64.58	67.93	60.08	63.89	7.20	6.14
Estonia	Europe	HIC	N. A.	78.56	73.65	76.06	78.32	71.55	74.86	80.22	74.49	77.30	80.06	69.31	74.49	81.58	73.22	77.28	-2.00	1.83
Eswatini	Africa	LMIC	N. A.	N. A.	N. A.	N. A.	N. A.	N. A.	N. A.	51.51	50.07	50.78	52.71	52.81	52.76	52.41	51.53	51.97	N. A.	N. A.
Ethiopia	Africa	LIC	74.5	53.50	55.44	54.46	53.23	53.45	53.34	53.17	50.06	51.59	54.66	51.02	52.81	53.68	49.69	51.64	-2.31	-3.94
Fiji	Oceania	UMIC	N. A.	N. A.	N. A.	N. A.	N. A.	N. A.	N. A.	70.07	62.41	66.13	69.95	63.30	66.54	71.24	66.53	68.84	N. A.	N. A.
Finland	Europe	HIC	N. A.	84.02	74.17	78.94	83.00	73.52	78.12	82.82	72.34	77.40	83.77	75.21	79.38	85.90	75.70	80.64	0.56	2.42
Former Yugoslav Republic of Macedonia (FYROM)	Europe	UMIC	N. A.	N. A.	N. A.	N. A.	68.95	63.61	66.23	N. A.	N. A.	N. A.	N. A.	N. A.	N. A.	N. A.	N. A.	N. A.	N. A.	N. A.
France	Europe	HIC	0.78	80.32	74.78	77.50	81.22	73.79	77.41	81.49	73.45	77.37	81.13	75.29	78.16	81.67	75.47	78.51	0.94	1.45
Gabon	Africa	UMIC	N. A.	65.14	55.91	60.35	62.84	57.94	60.34	64.76	61.68	63.20	63.40	64.15	63.78	62.82	64.74	63.77	4.60	4.60
Gambia, The	Africa	LIC	99.64	47.82	56.56	52.01	51.58	51.84	51.71	55.00	51.71	53.33	57.86	52.55	55.14	59.26	52.06	55.55	4.26	4.91
Georgia	Asia	LMIC	13.77	68.58	60.42	64.37	70.65	62.74	66.58	68.91	62.69	65.73	71.88	65.74	68.74	72.23	65.52	68.79	6.10	6.19
Germany	Europe	HIC	0.19	81.68	71.83	76.60	82.28	69.58	75.67	81.07	68.22	74.37	80.77	73.20	76.89	82.48	74.53	78.41	0.33	2.48
Ghana	Africa	LMIC	41.42	59.90	58.54	59.21	62.81	61.04	61.92	63.80	59.72	61.73	65.37	63.26	64.31	62.49	60.59	61.54	7.21	3.29
Greece	Europe	HIC	52.01	72.89	69.87	71.37	70.64	69.00	69.81	71.41	67.12	69.23	74.33	72.06	73.19	75.41	74.74	75.07	2.57	5.22
Guatemala	LAC	UMIC	3.84	58.32	55.96	57.13	58.24	56.38	57.30	59.65	54.64	57.09	61.54	56.65	59.04	59.91	53.95	56.85	2.76	-0.30
Guinea	Africa	LIC	10.94	48.78	50.30	49.53	52.12	51.20	51.66	52.81	50.89	51.84	52.47	50.53	51.49	50.96	49.09	50.02	2.77	0.69
Guyana	LAC	UMIC	0.17	64.66	57.90	61.19	61.90	55.05	58.37	62.93	58.27	60.56	61.06	56.81	58.90	59.77	58.81	59.28	-3.31	-2.82
Haiti	LAC	LIC	13.05	44.08	45.18	44.63	49.16	47.76	48.46	48.44	48.41	48.42	51.69	49.63	50.65	51.35	49.94	50.64	8.53	8.51
Honduras	LAC	LMIC	4	61.65	57.81	59.70	63.64	57.54	60.51	63.41	55.63	59.39	64.44	56.57	60.38	62.77	55.71	59.13	1.10	-0.70
Hungary	Europe	HIC	59.54	78.00	69.94	73.86	74.96	70.64	72.77	78.48	71.84	75.09	78.46	66.80	72.40	79.74	72.30	75.93	-1.89	2.90
Iceland	Europe	HIC	N. A.	79.29	65.60	72.12	79.75	64.97	71.98	79.20	67.21	72.96	77.52	63.85	70.35	78.17	64.16	70.82	-2.49	-1.81
India	Asia	LMIC	57.41	58.07	61.89	59.95	59.05	60.00	59.52	61.08	60.58	60.83	61.92	60.65	61.28	60.07	58.84	59.45	1.85	-0.74
Indonesia	Asia	LMIC	0.2	62.88	59.08	60.95	62.84	58.62	60.69	64.19	63.66	63.93	65.30	64.84	65.07	66.34	67.87	67.10	5.78	8.66
Iran, Islamic Rep.	Asia	UMIC	99.17	64.70	57.69	61.09	65.54	60.34	62.89	70.49	66.67	68.55	71.81	68.62	70.20	70.01	68.37	69.19	12.76	11.31
Iraq	Asia	UMIC	98.87	56.63	50.79	53.63	53.75	49.51	51.58	60.79	59.54	60.17	63.14	64.25	63.69	63.82	62.57	63.19	14.12	13.41
Ireland	Europe	HIC	N. A.	77.92	69.91	73.80	77.47	64.42	70.64	78.22	65.39	71.52	79.38	68.91	73.96	80.96	70.29	75.44	0.33	2.43
Israel	Asia	HIC	97.93	70.14	63.47	66.72	71.85	65.42	68.56	71.53	58.91	64.91	74.60	70.54	72.54	75.04	63.64	69.10	8.15	3.58
Italy	Europe	HIC	19.17	75.50	70.23	72.82	74.21	67.73	70.90	75.79	68.07	71.83	77.01	71.96	74.44	78.76	73.51	76.09	2.29	4.62
Jamaica	LAC	UMIC	1.47	66.57	59.38	62.87	65.90	59.42	62.57	68.80	62.39	65.51	68.66	68.30	68.48	68.97	68.49	68.73	7.79	8.14
Japan	Asia	HIC	N. A.	80.18	73.66	76.85	78.52	74.53	76.50	78.92	73.90	76.37	79.17	72.79	75.91	79.85	72.63	76.15	-1.33	-0.96
Jordan	Asia	UMIC	99.99	65.96	61.70	63.79	64.36	61.09	62.70	68.09	62.86	65.42	68.05	68.97	68.51	70.14	68.32	69.22	6.62	7.64
Kazakhstan	Asia	UMIC	97.84	71.09	65.13	68.05	68.13	64.51	66.30	68.71	62.66	65.61	71.82	65.62	68.65	72.44	66.99	69.66	0.86	2.27
Kenya	Africa	LMIC	89.86	54.93	61.82	58.27	56.83	64.83	60.70	57.03	55.24	56.13	60.17	57.84	58.99	60.60	59.25	59.92	0.89	2.19
Korea, Rep.	Asia	HIC	0.04	75.48	71.24	73.33	77.41	71.32	74.30	78.33	73.50	75.88	78.34	74.18	76.23	78.59	74.16	76.34	4.11	4.27
Kuwait	Asia	HIC	99.04	62.40	60.05	61.21	61.14	58.32	59.71	61.08	56.70	58.85	61.13	62.89	62.01	60.32	62.94	61.61	1.11	0.57
Kyrgyz Republic	Asia	LMIC	70.38	70.67	56.84	63.38	70.33	56.86	63.24	73.55	63.06	68.10	73.89	63.90	68.72	74.95	62.63	68.51	7.27	7.12
Lao PDR	Asia	LMIC	N. A.	61.37	56.90	59.09	60.63	56.14	58.34	63.01	54.97	58.85	62.43	54.96	58.58	63.16	56.48	59.73	-0.62	0.97
Latvia	Europe	HIC	N. A.	75.23	67.88	71.46	74.75	69.69	72.18	77.13	71.89	74.47	77.73	66.28	71.78	79.15	70.46	74.68	0.64	4.60
Lebanon	Asia	UMIC	34.6	64.93	64.20	64.56	64.79	62.34	63.55	65.67	62.44	64.03	66.68	64.20	65.43	66.84	64.64	65.73	1.24	1.66
Lesotho	Africa	LMIC	64.3	52.97	53.50	53.23	51.51	48.42	49.94	50.72	48.50	49.60	53.35	52.80	53.07	53.75	51.34	52.53	-0.23	-0.98
Liberia	Africa	LIC	N. A.	42.82	49.52	46.05	48.30	52.61	50.41	48.18	50.87	49.51	47.12	49.18	48.14	48.65	49.42	49.03	2.80	4.05
Lithuania	Europe	HIC	N. A.	73.63	68.22	70.87	72.90	69.98	71.42	75.10	69.62	72.31	74.95	68.28	71.54	76.70	72.34	74.49	0.97	5.08
Luxembourg	Europe	HIC	N. A.	75.00	64.88	69.75	76.09	65.20	70.43	76.25	61.23	68.33	75.24	59.92	67.15	74.89	60.87	67.52	-3.33	-2.91
Madagascar	Africa	LIC	28.33	43.50	47.69	45.54	45.59	49.37	47.44	46.70	47.71	47.20	49.14	47.77	48.45	49.01	47.78	48.39	4.05	3.96
Malawi	Africa	LIC	51.65	48.02	52.04	49.99	49.97	51.97	50.96	51.19	49.54	50.36	51.44	49.44	50.43	50.33	48.92	49.62	0.58	-0.58
Malaysia	Asia	UMIC	N. A.	69.68	63.37	66.45	70.01	66.98	68.48	69.56	66.95	68.24	71.76	65.46	68.54	70.88	65.36	68.07	2.95	2.26
Maldives	Asia	UMIC	N. A.	N. A.	N. A.	N. A.	N. A.	N. A.	N. A.	72.12	66.91	69.47	67.59	56.84	61.98	69.27	63.95	66.56	N. A.	N. A.
Mali	Africa	LIC	99.77	48.54	54.21	51.30	49.72	51.89	50.79	49.95	52.74	51.32	50.57	52.05	51.30	51.17	51.73	51.45	-0.09	0.11
Malta	Europe	HIC	87.33	77.02	68.09	72.42	74.20	67.74	70.89	76.11	69.27	72.61	75.97	68.91	72.35	75.75	69.00	72.29	-0.17	-0.26
Mauritania	Africa	LMIC	99.94	51.14	49.35	50.24	51.57	50.99	51.28	53.33	53.10	53.21	57.72	56.58	57.15	55.51	54.31	54.90	9.76	6.59
Mauritius	Africa	UMIC	N. A.	62.12	53.94	57.89	64.50	59.19	61.79	63.59	58.89	61.19	63.77	59.85	61.78	66.71	60.47	63.51	5.34	7.86
Mexico	LAC	UMIC	76.8	69.13	64.45	66.75	65.21	55.78	60.31	68.51	58.78	63.46	70.44	58.73	64.32	69.13	55.03	61.68	-3.12	-6.66

Moldova	Europe	LMIC	47.67	74.22	60.44	66.98	74.51	61.48	67.68	76.52	62.05	68.90	75.41	61.29	67.99	74.60	61.57	67.78	1.45	1.07
Mongolia	Asia	LMIC	97.28	64.18	55.66	59.77	63.86	56.11	59.86	65.84	58.10	61.85	64.47	58.73	61.54	63.98	59.54	61.72	2.38	2.60
Montenegro	Europe	UMIC	N. A.	67.29	61.56	64.36	67.63	61.26	64.37	67.25	61.62	64.38	70.19	63.17	66.59	68.21	65.87	67.03	3.19	3.69
Morocco	Africa	LMIC	97.98	66.66	60.06	63.28	66.27	61.09	63.63	69.07	63.75	66.36	71.29	67.02	69.12	70.53	68.51	69.51	8.19	8.70
Mozambique	Africa	LIC	67.23	49.24	51.97	50.59	50.66	53.93	52.27	53.03	51.20	52.11	54.13	52.32	53.21	51.05	50.47	50.76	3.70	0.22
Myanmar	Asia	LMIC	11.1	59.54	53.53	56.45	59.03	55.46	57.22	62.18	57.03	59.55	64.58	57.23	60.79	64.95	57.45	61.09	6.18	6.60
Namibia	Africa	UMIC	99.94	59.27	52.30	55.68	58.93	51.76	55.23	59.87	53.13	56.40	61.63	54.13	57.76	61.77	54.06	57.79	2.97	3.01
Nepal	Asia	LIC	6.07	61.57	54.24	57.79	62.75	54.83	58.66	65.03	56.77	60.76	66.54	58.70	62.50	66.88	60.24	63.48	6.67	8.01
Netherlands	Europe	HIC	N. A.	79.94	72.13	75.94	79.47	67.87	73.44	80.38	69.14	74.55	80.37	71.76	75.95	81.56	72.58	76.94	0.04	1.46
New Zealand	Oceania	HIC	1.61	77.62	63.64	70.28	77.86	65.10	71.19	79.80	71.31	75.44	78.25	69.30	73.64	79.13	69.90	74.37	4.46	5.50
Nicaragua	LAC	LMIC	3.83	63.11	55.41	59.14	66.38	56.67	61.34	67.94	56.84	62.14	68.66	56.20	62.11	66.29	56.47	61.18	4.48	2.99
Niger	Africa	LIC	99.99	44.80	56.02	50.09	48.51	54.00	51.18	49.13	53.29	51.17	49.26	51.66	50.44	48.37	50.00	49.18	0.07	-1.72
Nigeria	Africa	LMIC	59.65	48.65	51.71	50.16	47.48	50.02	48.73	46.41	50.01	48.17	49.28	52.42	50.83	48.93	53.77	51.29	0.95	1.65
North Macedonia	Europe	UMIC	N. A.	N. A.	N. A.	N. A.	N. A.	N. A.	N. A.	71.18	65.80	68.43	72.18	67.15	69.62	73.38	66.28	69.74	N. A.	N. A.
Norway	Europe	HIC	N. A.	83.94	68.33	75.73	81.17	66.85	73.66	80.66	65.21	72.52	80.76	62.27	70.92	81.98	62.27	71.45	-6.53	-5.67
Oman	Asia	HIC	99.88	64.31	60.97	62.62	63.91	59.25	61.53	66.00	64.79	65.39	68.53	67.46	67.99	68.92	65.12	66.99	7.57	6.19
Pakistan	Asia	LMIC	93.77	55.63	54.62	55.12	54.89	54.23	54.56	55.57	52.18	53.85	56.17	55.58	55.87	57.72	56.53	57.12	1.06	2.83
Panama	LAC	HIC	0.29	63.94	61.93	62.93	64.89	59.73	62.26	66.31	62.52	64.39	69.19	63.84	66.46	67.98	57.97	62.78	5.06	0.05
Papua New Guinea	Oceania	LMIC	N. A.	N. A.	N. A.	N. A.	N. A.	N. A.	N. A.	51.62	49.93	50.77	51.66	49.48	50.56	51.33	49.46	50.38	N. A.	N. A.
Paraguay	LAC	UMIC	53.32	66.14	58.12	62.00	67.21	57.86	62.36	68.38	60.88	64.52	68.04	62.38	65.15	67.20	58.86	62.89	4.36	1.28
Peru	LAC	UMIC	27.79	65.97	58.11	61.92	68.45	61.24	64.74	71.19	65.28	68.17	71.75	66.36	69.00	71.09	62.57	66.69	9.92	6.77
Philippines	Asia	LMIC	N. A.	64.33	59.99	62.12	65.03	60.35	62.65	64.94	63.55	64.24	65.50	63.59	64.54	64.51	58.40	61.38	3.37	-1.00
Poland	Europe	HIC	N. A.	75.78	63.44	69.34	73.67	68.28	70.93	75.93	68.54	72.14	78.10	73.49	75.76	80.22	77.31	78.75	8.74	12.95
Portugal	Europe	HIC	39.28	75.57	71.44	73.47	74.03	72.53	73.27	76.43	73.16	74.78	77.65	75.27	76.45	78.64	68.60	73.45	4.18	0.17
Qatar	Asia	HIC	97.36	63.10	63.59	63.34	60.85	59.04	59.94	64.21	66.59	65.39	62.84	58.44	60.60	65.06	57.63	61.23	-3.83	-2.83
Romania	Europe	UMIC	43.51	74.13	64.11	68.94	71.22	63.78	67.40	72.73	62.61	67.48	74.78	69.97	72.33	74.97	71.94	73.44	4.60	6.13
Russian Federation	Europe	UMIC	20.59	68.93	61.29	65.00	68.90	63.94	66.38	70.94	67.09	68.99	71.92	69.94	70.92	73.75	71.15	72.44	8.23	10.38
Rwanda	Africa	LIC	41.27	55.01	55.97	55.49	56.09	54.15	55.11	56.12	54.02	55.06	56.07	52.96	54.49	56.92	53.97	55.43	-1.38	-0.05
Sao Tome and Principe	Africa	LMIC	N. A.	N. A.	N. A.	N. A.	N. A.	N. A.	N. A.	65.48	57.98	61.62	62.57	56.08	59.24	58.79	56.50	57.63	N. A.	N. A.
Saudi Arabia	Asia	HIC	99.94	62.68	51.30	56.70	62.92	63.17	63.04	62.58	63.75	63.16	64.19	65.10	64.64	64.58	64.52	64.55	10.83	10.69
Senegal	Africa	LIC	98.52	56.24	57.68	56.95	57.17	56.96	57.07	57.30	53.66	55.45	58.27	55.47	56.85	58.43	56.18	57.30	-0.12	0.49
Serbia	Europe	UMIC	25.43	73.58	64.89	69.09	72.14	64.52	68.22	74.47	67.24	70.77	76.25	70.25	73.19	76.64	71.28	73.91	5.69	6.68
Sierra Leone	Africa	LIC	N. A.	47.11	52.13	49.56	49.11	50.19	49.65	49.24	49.39	49.32	51.91	50.44	51.17	51.69	49.39	50.53	2.20	1.30
Singapore	Asia	HIC	N. A.	68.97	62.80	65.82	71.31	59.20	64.97	70.07	60.31	65.01	67.69	57.60	62.44	70.83	59.66	65.01	-4.58	-0.91
Slovak Republic	Europe	HIC	N. A.	76.93	70.49	73.64	75.60	71.43	73.48	77.77	71.47	74.55	78.65	71.14	74.80	80.58	74.76	77.62	1.67	5.60
Slovenia	Europe	HIC	N. A.	80.54	74.42	77.42	79.98	72.02	75.89	79.41	65.36	72.04	79.80	70.66	75.09	81.60	76.32	78.92	-3.18	2.10
Somalia	Africa	LIC	99.93	N. A.	N. A.	N. A.	N. A.	N. A.	N. A.	N. A.	N. A.	N. A.	45.94	47.45	46.69	45.11	47.65	46.36	N. A.	N. A.
South Africa	Africa	UMIC	92.45	61.25	53.82	57.41	60.83	53.87	57.24	61.48	52.53	56.83	63.41	54.76	58.93	63.74	54.38	58.87	2.20	2.16
South Sudan	Africa	LIC	87.22	N. A.	N. A.	N. A.	N. A.	N. A.	N. A.	N. A.	N. A.	N. A.	42.36	50.14	46.09	37.08	45.38	41.02	N. A.	N. A.
Spain	Europe	HIC	72.26	76.75	69.88	73.24	75.42	70.27	72.80	77.84	74.21	76.01	78.11	73.30	75.67	79.46	73.23	76.28	3.38	4.29
Sri Lanka	Asia	LMIC	N. A.	65.91	56.38	60.96	64.56	56.65	60.48	65.84	57.20	61.37	66.88	63.68	65.26	68.10	64.05	66.04	5.84	6.97
Sudan	Africa	LMIC	99.99	49.87	58.77	54.14	49.58	56.09	52.73	51.36	51.50	51.43	49.56	49.94	49.75	49.48	50.61	50.04	-6.46	-6.05
Suriname	LAC	UMIC	N. A.	70.35	66.38	68.34	67.97	63.63	65.77	68.90	62.66	65.71	70.22	61.54	65.74	69.42	66.29	67.84	-3.51	-0.72
Swaziland	Africa	LMIC	65.47	54.98	56.57	55.77	50.67	48.50	49.57	N. A.	N. A.	N. A.	N. A.	N. A.	N. A.	N. A.	N. A.	N. A.	N. A.	N. A.
Sweden	Europe	HIC	N. A.	85.61	76.66	81.01	84.98	75.12	79.90	84.99	70.24	77.26	84.72	74.98	79.70	85.61	76.50	80.93	-1.81	-0.10
Switzerland	Europe	HIC	N. A.	81.18	67.59	74.08	80.09	67.45	73.50	80.55	64.24	71.93	80.60	68.78	74.46	81.14	68.65	74.63	0.43	0.72
Syrian Arab Republic	Asia	LIC	96.57	58.14	58.62	58.38	55.02	54.79	54.91	55.91	53.28	54.58	57.16	55.65	56.40	55.86	54.00	54.93	-2.80	-4.88
Tajikistan	Asia	LIC	48.32	66.77	58.91	62.72	67.18	56.02	61.35	71.01	56.53	63.36	70.09	56.71	63.04	70.44	56.21	62.92	0.79	0.68
Tanzania	Africa	LIC	66.76	52.08	53.91	52.98	55.15	55.06	55.11	55.82	53.29	54.54	56.64	55.14	55.89	56.43	53.77	55.08	4.10	2.98
Thailand	Asia	UMIC	12.42	69.54	64.43	66.93	69.24	65.49	67.34	73.00	66.67	69.76	74.54	71.39	72.95	74.19	72.04	73.11	8.46	8.68
Timor-Leste	Asia	LMIC	N. A.	61.51	57.96	59.71	N. A.	N. A.	N. A.	N. A.	N. A.	N. A.	N. A.	N. A.	N. A.	N. A.	N. A.	N. A.	N. A.	N. A.
Togo	Africa	LIC	53.33	50.18	53.03	51.59	52.00	52.14	52.07	51.60	53.24	52.41	52.70	51.94	52.32	53.23	52.67	52.95	1.01	1.90
Trinidad and Tobago	LAC	HIC	N. A.	69.11	62.29	65.61	67.51	61.04	64.20	69.46	61.70	65.46	67.47	60.18	63.72	65.72	60.85	63.24	-2.66	-3.41
Tunisia	Africa	LMIC	98.51	68.66	60.47	64.44	66.15	61.01	63.53	69.99	62.35	66.06	71.37	67.08	69.19	71.44	67.88	69.64	6.59	7.21
Turkey	Asia	UMIC	71.27	68.48	66.39	67.43	65.96	62.48	64.19	68.49	60.90	64.58	70.30	65.59	67.91	70.38	64.81	67.54	0.72	0.22

Turkmenistan	Asia	UMIC	99.99	56.73	49.21	52.83	59.47	48.61	53.77	64.02	54.09	58.85	62.87	56.30	59.50	60.54	56.63	58.55	9.36	7.94
Uganda	Africa	LIC	38.9	52.86	50.84	51.84	54.93	49.86	52.33	52.45	50.46	51.45	52.80	48.76	50.74	52.55	52.14	52.34	-1.51	0.70
Ukraine	Europe	LMIC	31.76	72.73	61.15	66.69	72.34	61.24	66.56	72.81	61.29	66.81	74.24	64.16	69.02	75.51	65.95	70.57	3.19	5.35
United Arab Emirates	Asia	HIC	98.73	66.01	62.13	64.04	69.22	63.07	66.07	68.22	62.61	65.35	68.80	63.76	66.23	70.17	61.92	65.92	3.13	2.80
United Kingdom	Europe	HIC	N. A.	78.28	66.74	72.28	78.67	68.68	73.50	79.38	69.26	74.15	79.79	73.03	76.34	79.97	73.60	76.72	5.52	6.05
United States	North America	HIC	45.08	72.40	67.10	69.70	73.05	65.96	69.42	74.52	65.38	69.80	76.43	70.49	73.40	76.01	70.44	73.17	5.24	4.91
Uruguay	LAC	HIC	0.11	71.05	64.99	67.95	70.42	62.49	66.34	72.55	65.74	69.06	74.28	68.24	71.20	74.55	68.37	71.39	4.58	4.86
Uzbekistan	Asia	LMIC	97.28	71.25	57.62	64.07	70.29	60.80	65.37	73.03	62.40	67.50	71.77	63.02	67.25	70.52	61.35	65.78	4.19	2.13
Vanuatu	Oceania	LMIC	N. A.	N. A.	N. A.	N. A.	N. A.	N. A.	N. A.	59.87	61.48	60.67	60.89	56.80	58.81	60.52	60.26	60.39	N. A.	N. A.
Venezuela, RB	LAC	UMIC	15.53	65.83	63.28	64.55	64.00	60.33	62.14	63.05	62.11	62.58	61.68	60.68	61.18	59.32	56.63	57.96	-4.77	-9.31
Vietnam	Asia	LMIC	0.46	67.89	58.27	62.90	69.67	59.18	64.21	71.09	62.07	66.42	73.80	69.63	71.68	72.85	69.05	70.92	12.21	11.13
Yemen, Rep.	Asia	LIC	99.9	49.80	48.41	49.10	45.66	47.58	46.61	51.21	50.99	51.10	49.71	50.09	49.90	50.40	49.62	50.01	1.12	1.28
Zambia	Africa	LMIC	65.35	51.08	57.78	54.32	53.13	55.30	54.20	52.50	52.24	52.37	51.94	48.42	50.15	52.48	48.59	50.50	-6.01	-5.51
Zimbabwe	Africa	LIC	97.53	56.11	59.10	57.59	58.76	57.43	58.09	61.82	54.32	57.95	60.98	55.82	58.34	59.93	55.91	57.89	1.12	0.44

Table S3. Statistics details for Fig. 1 and Fig. S3.

A. Statistics for the ANOVA test of the evenness scores across different regions in 2021.

ANOVA	F	df	*p*	LSD						Average
				p	Asia	LAC	Oceania	Europe	North A.	
Geographic location (N=165)	43.28	5	<0.001	Africa (N=48)	<0.001	<0.001	0.008	<0.001	<0.001	53.76
				Asia (N=45)	1	0.070	0.523	<0.001	0.036	62.38
				LAC (N=26)		1	0.764	<0.001	0.008	59.91
				Oceania (N=5)			1	<0.001	0.030	60.72
				Europe (N=39)				1	0.998	70.78
				North A. (N=2)					1	70.77
UN income groups (N=165)	71.63	3	<0.001	*p*	UMIC	LMIC	LIC			
				HIC (N=50)	<0.001	<0.001	<0.001			68.74
				UMIC (N=44)	1	<0.001	<0.001			63.12
				LMIC (N=41)		1	<0.001			58.81
				LIC (N=30)			1			50.83
Aridity (N=115)	2.03	3	0.114	*p*	Moderately	Severely	Extremely			
				Slightly (N=50)	0.183	0.308	0.020			61.95
				Moderately (N=19)	1	0.709	0.412			59.07
				Severely (N=26)		1	0.208			59.97
				Extremely (N=20)			1			56.97

B. Statistics for the ANOVA test of the mean index score across different regions in 2021.

ANOVA	F	df	*p*	LSD						Average
				p	Asia	LAC	Oceania	Europe	North A.	
Geographic location (N=165)	64.18	5	<0.001	Africa (N=48)	<0.001	<0.001	<0.001	<0.001	<0.001	55.05
				Asia (N=45)	1	0.737	0.77	<0.001	0.016	67.52
				LAC (N=26)		1	0.91	<0.001	0.024	68.16
				Oceania (N=5)			1	<0.001	0.055	67.56
				Europe (N=39)				1	0.602	78.74
				North A. (N=2)					1	77.58
UN income groups (N=165)	93.21	3	<0.001	*p*	UMIC	LMIC	LIC			
				HIC (N=50)	<0.001	<0.001	<0.001			76.70
				UMIC (N=44)	1	<0.001	<0.001			69.48
				LMIC (N=41)		1	<0.001			62.63
				LIC (N=30)			1			51.88
Aridity (N=115)	5.04	3	0.003	*p*	Moderately	Severely	Extremely			
				Slightly (N=50)	0.068	0.149	<0.001			67.89
				Moderately (N=19)	1	0.632	0.114			62.94
				Severely (N=26)		1	0.03			64.98
				Extremely (N=20)			1			58.11

C. Statistics for the ANOVA test of the sustainable development score across different regions in 2021.

ANOVA	F	df	*p*	LSD						Average
				p	Asia	LAC	Oceania	Europe	North A.	
Geographic location (N=165)	60.86	5	<0.001	Africa (N=48)	<0.001	<0.001	0.001	<0.001	<0.001	54.37
				Asia (N=45)	1	0.397	0.874	<0.001	0.013	64.87
				LAC (N=26)		1	0.784	<0.001	0.006	63.86
				Oceania (N=5)			1	<0.001	0.025	63.98
				Europe (N=39)				1	0.702	74.62
				North A. (N=2)					1	74.09
UN income groups (N=165)	86.17	3	<0.001	*p*	UMIC	LMIC	LIC			
				HIC (N=50)	<0.001	<0.001	<0.001			72.57
				UMIC (N=44)	1	<0.001	<0.001			66.19
				LMIC (N=41)		1	<0.001			60.66
				LIC (N=30)			1			51.31
Aridity (N=115)	3.62	3	0.015	*p*	Moderately	Severely	Extremely			
				Slightly (N=50)	0.116	0.153	0.002			64.80
				Moderately (N=19)	1	0.795	0.191			60.95
				Severely (N=26)		1	0.096			62.37
				Extremely (N=20)			1			57.50

D1. Statistics for the ANOVA test of the effective development score across different regions from 2017 to 2020.

ANOVA	F	df	*p*	LSD test						Average
				p	Asia	LAC	Oceania	Europe	North A.	
Geographic locations (N=163)	2.49	5	0.034	Africa (N=43)	0.011	0.413	0.454	0.520	0.917	2.30
				Asia (N=43)	1	0.156	0.130	0.002	0.505	4.54
				LAC (N=26)		1	0.310	0.174	0.861	3.12
				Oceania (N=2)			1	0.583	0.537	0.12
				Europe (N=38)				1	0.763	1.72
				North A. (N=2)					1	2.60
UN income groups (N=166)	6.65	3	<0.001	*p*	UMIC	LMIC	LIC			
				HIC (N=49)	<0.001	0.002	0.991			1.50
				UMIC (N=41)	1	0.728	0.003			4.44
				LMIC (N=36)		1	0.008			4.13
				LIC (N=28)			1			1.51
Aridity (N=115)	1.77	3	0.158	*p*	Moderately	Severely	Extremely			
				Slightly (N=50)	0.112	0.162	0.559			3.86
				Moderately (N=19)	1	0.787	0.072			2.10
				Severely (N=26)		1	0.102			2.44
				Extremely (N=20)			1			4.50

D2. Statistics for the ANOVA test of the effective development score across different regions from 2017 to 2021.

ANOVA	F	df	*p*	LSD						Average	2021 vs. 2020
				p	Asia	LAC	Oceania	Europe	North A.		
Geographic location (N=154)	2.92	5	0.015	Africa (N=43)	0.004	0.536	0.643	0.089	0.867	1.78	-0.52
				Asia (N=43)	1	0.002	0.183	0.261	0.481	4.34	-0.2
				LAC (N=26)		1	0.805	0.037	0.708	1.16	-1.96
				Oceania (N=2)			1	0.325	0.648	0.42	0.3
				Europe (N=38)				1	0.721	3.33	1.61
				North A. (N=2)					1	2.27	-0.33
UN income groups (N=154)	3.85	3	0.011	*p*	UMIC	LMIC	LIC				
				HIC (N=49)	0.12	0.083	0.129			2.30	0.8
				UMIC (N=41)	1	0.829	0.005			3.66	-0.78
				LMIC (N=36)		1	0.004			3.86	-0.27
				LIC (N=28)			1			0.83	-0.68
Aridity (N=112)	1.56	3	0.204	*p*	Moderately	Severely	Extremely				
				Slightly (N=50)	0.125	0.168	0.663			3.32	-0.54
				Moderately (N=19)	1	0.815	0.103			1.55	-0.55
				Severely (N=24)		1	0.135			1.86	-0.58
				Extremely (N=19)			1			3.82	-0.68

Table S4. Spearman correlations among SDGs across different geographic locations (A), UN income groups (B), and arid levels (C) in 2021. *, $p < 0.05$; **, $p < 0.01$.

A1. Africa

r	SDG 1	SDG 2	SDG 3	SDG 4	SDG 5	SDG 6	SDG 7	SDG 8	SDG 9	SDG 10	SDG 11	SDG 12	SDG 13	SDG 14	SDG 15	SDG 16	SDG 17
**SDG 1**	1	0.186	0.574**	0.509**	0.047	0.617**	0.704**	0.363*	0.748**	0.284	0.498**	-0.284	-0.674**	-0.402*	0.028	0.563**	0.300*
**SDG 2**		1	0.335*	0.293*	0.124	0.225	0.187	0.497**	0.430**	-0.010	0.164	0.268	-0.144	-0.262	-0.060	0.189	0.024
**SDG 3**			1	0.633**	0.348*	0.586**	0.570**	0.408**	0.713**	0.066	0.606**	-.376**	-0.531**	-0.341	-0.078	0.779**	0.386**
**SDG 4**				1	0.542**	0.497**	0.630**	0.204	0.673**	-0.173	0.622**	-.302*	-0.645**	-0.265	-0.017	0.568**	0.505**
**SDG 5**					1	0.190	0.236	0.087	0.321*	-0.465**	0.519**	-0.205	-0.332*	0.109	-0.208	0.187	0.448**
**SDG 6**						1	0.551**	0.267	0.679**	-0.073	0.609**	-.318*	-0.680**	-0.284	0.138	0.615**	0.447**
**SDG 7**							1	0.110	0.694**	-0.078	0.527**	-.305*	-0.801**	-0.340	0.040	0.497**	0.272
**SDG 8**								1	0.386**	0.199	0.301*	0.142	-0.124	-0.119	-0.056	0.451**	0.096
**SDG 9**									1	-0.089	0.552**	-.326*	-0.742**	-0.388*	0.050	0.552**	0.395**
**SDG 10**										1	-0.100	0.144	0.283	-0.305	-0.161	0.065	-0.190
**SDG 11**											1	-.382**	-0.614**	-0.244	-0.086	0.625**	0.586**
**SDG 12**												1	0.368*	0.102	-0.132	-0.352*	-0.203
**SDG 13**													1	0.248	-0.132	-0.506**	-0.542**
**SDG 14**														1	0.169	-0.395*	-0.280
**SDG 15**															1	-0.080	0.048
**SDG 16**																1	0.471**
**SDG 17**																	1

A2. Asia

r	SDG 1	SDG 2	SDG 3	SDG 4	SDG 5	SDG 6	SDG 7	SDG 8	SDG 9	SDG 10	SDG 11	SDG 12	SDG 13	SDG 14	SDG 15	SDG 16	SDG 17
**SDG 1**	1	0.061	0.772**	0.369*	0.139	0.450**	0.607**	0.359*	0.686**	0.139	0.455**	-0.740**	-0.596**	-0.138	0.032	0.447**	0.270
**SDG 2**		1	0.436**	0.325*	0.497**	0.043	0.224	0.230	0.494**	0.072	0.214	-0.101	-0.203	-0.276	-0.197	0.330*	-0.072
**SDG 3**			1	0.669**	0.308*	0.329*	0.803**	0.505**	0.836**	0.109	0.467**	-0.825**	-0.767**	-0.370*	0.008	0.659**	0.185
**SDG 4**				1	0.481**	0.376*	0.464**	0.631**	0.557**	0.117	0.539**	-0.568**	-0.508**	-0.255	0.250	0.492**	0.310*
**SDG 5**					1	0.442**	0.066	0.434**	0.362*	0.230	0.485**	-0.144	-0.237	-0.324	0.134	0.320*	0.004
**SDG 6**						1	0.267	0.497**	0.296*	0.032	0.483**	-0.187	-0.194	-0.324	0.062	0.213	0.323*
**SDG 7**							1	0.291	0.586**	0.134	0.436**	-0.684**	-0.576**	-0.311	-0.010	0.677**	0.280
**SDG 8**								1	0.496**	0.010	0.383**	-0.405**	-0.360*	-0.263	0.012	0.388**	0.024
**SDG 9**									1	0.010	0.351*	-0.678**	-0.667**	-0.228	-0.088	0.526**	0.166
**SDG 10**										1	0.057	-0.161	-0.173	-0.189	0.368*	0.190	0.002
**SDG 11**											1	-0.327*	-0.265	-0.143	0.092	0.439**	0.387**
**SDG 12**												1	0.890**	0.261	-0.104	-0.603**	-0.203
**SDG 13**													1	0.210	-0.086	-0.560**	-0.152
**SDG 14**														1	0.297	-0.093	-0.087
**SDG 15**															1	0.151	0.238
**SDG 16**																1	0.203
**SDG 17**																	1

A3. Latin America and Caribbean

r	SDG 1	SDG 2	SDG 3	SDG 4	SDG 5	SDG 6	SDG 7	SDG 8	SDG 9	SDG 10	SDG 11	SDG 12	SDG 13	SDG 14	SDG 15	SDG 16	SDG 17
**SDG 1**	1	0.410*	0.632**	0.605**	0.447*	0.355	0.408*	0.531**	0.528**	0.099	0.503*	-0.417*	-0.433*	-0.169	-0.045	0.434*	0.244
**SDG 2**		1	0.134	0.359	0.185	0.452*	0.205	0.190	0.461*	-0.009	0.136	-0.381	-0.180	0.146	-0.108	0.233	0.300
**SDG 3**			1	0.571**	0.525**	0.504**	0.490*	0.467*	0.565**	0.042	0.341	-0.235	-0.376	-0.137	0.019	0.666**	0.610**
**SDG 4**				1	0.573**	0.339	0.357	0.301	0.541**	-0.080	0.051	-0.208	-0.339	0.060	0.041	0.361	0.457*
**SDG 5**					1	0.175	0.192	0.279	0.383	0.206	0.241	0.026	-0.033	-0.123	0.322	0.351	0.536**
**SDG 6**						1	0.529**	0.184	0.763**	-0.394	0.282	-0.095	-0.142	0.067	-0.071	0.229	0.439*
**SDG 7**							1	0.261	0.652**	-0.098	0.394*	-0.602**	-0.524**	0.034	0.229	0.545**	0.515**
**SDG 8**								1	0.444*	0.116	0.168	-0.311	-0.493*	0.162	0.158	0.322	0.252
**SDG 9**									1	-0.317	0.405*	-0.346	-0.400*	0.094	0.024	0.516**	0.486*
**SDG 10**										1	-0.065	0.038	0.128	-0.112	0.195	0.059	0.008
**SDG 11**											1	-0.256	-0.276	0.096	0.186	0.520**	0.174
**SDG 12**												1	0.802**	0.297	0.128	-0.535**	-0.227
**SDG 13**													1	0.175	-0.052	-0.594**	-0.194
**SDG 14**														1	0.311	-0.029	0.043
**SDG 15**															1	0.106	0.188
**SDG 16**																1	0.581**

A4. Oceania

r	SDG 1	SDG 2	SDG 3	SDG 4	SDG 5	SDG 6	SDG 7	SDG 8	SDG 9	SDG 10	SDG 11	SDG 12	SDG 13	SDG 14	SDG 15	SDG 16	SDG 17
**SDG 1**	1	1.000**	0.900*	0.900*	0.900*	0.900*	1.000**	1.000**	0.900*	0.800	0.600	-0.800	-0.800	-0.600	-0.500	0.900*	0.900*
**SDG 2**		1	0.900*	0.900*	0.900*	0.900*	1.000**	1.000**	0.900*	0.800	0.600	-0.800	-0.800	-0.600	-0.500	0.900*	0.900*
**SDG 3**			1	1.000**	0.800	1.000**	0.900*	0.900*	1.000**	0.900*	0.700	-0.900*	-0.900*	-0.500	-0.300	0.700	0.800
**SDG 4**				1	0.800	1.000**	0.900*	0.900*	1.000**	0.900*	0.700	-0.900*	-0.900*	-0.500	-0.300	0.700	0.800
**SDG 5**					1	0.800	0.900*	0.900*	0.800	0.600	0.500	-0.600	-0.900*	-0.700	-0.300	0.800	0.700
**SDG 6**						1	0.900*	0.900*	1.000**	0.900*	0.700	-0.900*	-0.900*	-0.500	-0.300	0.700	0.800
**SDG 7**							1	1.000**	0.900*	0.800	0.600	-0.800	-0.800	-0.600	-0.500	0.900*	0.900*
**SDG 8**								1	0.900*	0.800	0.600	-0.800	-0.800	-0.600	-0.500	0.900*	0.900*
**SDG 9**									1	0.900*	0.700	-0.900*	-0.900*	-0.500	-0.300	0.700	0.800
**SDG 10**										1	0.400	-1.000**	-0.700	-0.600	-0.100	0.500	0.900*
**SDG 11**											1	-0.400	-0.600	0.200	-0.700	0.700	0.300
**SDG 12**												1	0.700	0.600	0.100	-0.500	-0.900*
**SDG 13**													1	0.600	0.100	-0.600	-0.600
**SDG 14**														1	-0.300	-0.300	-0.700
**SDG 15**															1	-0.800	-0.300
**SDG 16**																1	0.700
**SDG 17**																	1

A5. Europe

r	SDG 1	SDG 2	SDG 3	SDG 4	SDG 5	SDG 6	SDG 7	SDG 8	SDG 9	SDG 10	SDG 11	SDG 12	SDG 13	SDG 14	SDG 15	SDG 16	SDG 17
**SDG 1**	1	-0.109	0.254	0.459**	0.346*	0.185	0.284	0.353*	0.297	0.563**	0.362*	-0.241	-0.415**	0.317	-0.100	0.239	0.050
**SDG 2**		1	0.065	0.003	-0.025	0.246	0.109	0.197	0.128	-0.123	-0.032	-0.028	-0.029	0.139	0.220	-0.031	-0.312
**SDG 3**			1	0.612**	0.801**	0.524**	0.729**	0.558**	0.904**	0.471**	0.840**	-0.845**	-0.828**	0.170	0.012	0.805**	-0.024
**SDG 4**				1	0.574**	0.353*	0.540**	0.578**	0.625**	0.316*	0.559**	-0.633**	-0.680**	0.135	0.054	0.480**	-0.072
**SDG 5**					1	0.531**	0.735**	0.554**	0.820**	0.505**	0.752**	-0.612**	-0.655**	0.269	0.006	0.717**	0.157
**SDG 6**						1	0.441**	0.568**	0.607**	0.268	0.602**	-0.405*	-0.467**	0.474**	0.429**	0.561**	-0.322*
**SDG 7**							1	0.554**	0.728**	0.526**	0.583**	-0.606**	-0.575**	-0.087	-0.079	0.527**	0.011
**SDG 8**								1	0.644**	0.503**	0.616**	-0.574**	-0.654**	0.413*	0.490**	0.653**	-0.156
**SDG 9**									1	0.426**	0.870**	-0.806**	-0.839**	0.209	0.040	0.782**	0.018
**SDG 10**										1	0.386*	-0.352*	-0.455**	0.234	-0.014	0.439**	0.272
**SDG 11**											1	-0.755**	-0.835**	0.283	0.135	0.756**	-0.164
**SDG 12**												1	0.900**	-0.166	0.003	-0.779**	0.071
**SDG 13**													1	-0.338	-0.106	-0.814**	0.078
**SDG 14**														1	0.471**	0.418*	0.003
**SDG 15**															1	0.198	-0.487**
**SDG 16**																1	-0.058
**SDG 17**																	1

B1. High-income countries

HIC	SDG 1	SDG 2	SDG 3	SDG 4	SDG 5	SDG 6	SDG 7	SDG 8	SDG 9	SDG 10	SDG 11	SDG 12	SDG 13	SDG 14	SDG 15	SDG 16	SDG 17
**SDG 1**	1	-0.081	0.294	0.195	0.382^{*}	0.331^{*}	0.265	0.493^{**}	0.264	0.400^{**}	0.336^{*}	-0.409^{**}	-0.311^{*}	0.253	0.070	0.424^{**}	0.136
**SDG 2**		1	0.028	0.040	-0.096	0.095	0.061	0.113	0.197	-0.123	-0.105	0.159	0.172	-0.041	0.013	0.037	-0.102
**SDG 3**			1	0.566^{**}	0.646^{**}	0.347^{*}	0.555^{**}	0.486^{**}	0.825^{**}	0.478^{**}	0.602^{**}	-0.594^{**}	-0.292^{*}	-0.100	0.067	0.687^{**}	0.287^{*}
**SDG 4**				1	0.408^{**}	0.285^{*}	0.388^{**}	0.479^{**}	0.524^{**}	0.349^{*}	0.500^{**}	-0.488^{**}	-0.227	-0.003	0.185	0.407^{**}	0.282^{*}
**SDG 5**					1	0.569^{**}	0.591^{**}	0.539^{**}	0.617^{**}	0.363^{*}	0.651^{**}	-0.434^{**}	0.013	0.213	0.259	0.590^{**}	0.396^{**}
**SDG 6**						1	0.141	0.575^{**}	0.373^{**}	0.087	0.476^{**}	-0.057	0.239	0.352^{*}	0.542^{**}	0.430^{**}	0.237
**SDG 7**							1	0.349^{*}	0.435^{**}	0.343^{*}	0.348^{*}	-0.454^{**}	0.064	-0.248	-0.125	0.320^{*}	0.362^{**}
**SDG 8**								1	0.523^{**}	0.566^{**}	0.361^{*}	-0.142	0.049	0.230	0.536^{**}	0.576^{**}	0.194
**SDG 9**									1	0.399^{**}	0.588^{**}	-0.449^{**}	-0.271	0.046	0.083	0.616^{**}	0.266
**SDG 10**										1	0.389^{*}	-0.470^{**}	-0.562^{**}	0.184	0.373^{*}	0.583^{**}	0.131
**SDG 11**											1	-0.475^{**}	-0.181	0.125	0.268	0.457^{**}	0.299^{*}
**SDG 12**												1	0.565^{**}	0.126	0.079	-0.493^{**}	-0.230
**SDG 13**													1	0.003	0.281^{*}	-0.263	-0.026
**SDG 14**														1	0.410^{**}	0.250	0.108
**SDG 15**															1	0.267	-0.077
**SDG 16**																1	0.264
**SDG 17**																	1

B2. Upper-middle-income countries

r	SDG 1	SDG 2	SDG 3	SDG 4	SDG 5	SDG 6	SDG 7	SDG 8	SDG 9	SDG 10	SDG 11	SDG 12	SDG 13	SDG 14	SDG 15	SDG 16	SDG 17
**SDG 1**	1	0.309*	0.599**	0.431**	-0.192	0.119	0.205	0.401**	0.489**	0.611**	0.244	-0.082	-0.152	-0.167	-0.052	0.381*	-0.011
**SDG 2**		1	0.327*	0.193	0.106	0.162	0.050	0.100	0.228	0.127	0.091	-0.053	0.048	-0.003	0.086	0.097	0.020
**SDG 3**			1	0.527**	0.079	0.452**	0.319*	0.426**	0.412**	0.256	0.095	0.018	0.034	-0.206	-0.016	0.352*	0.150
**SDG 4**				1	0.097	0.266	-0.041	0.243	0.268	0.095	0.182	0.027	0.066	-0.141	-0.165	0.167	0.160
**SDG 5**					1	0.240	-0.135	0.250	0.134	-0.527**	0.206	0.027	0.020	0.109	0.189	-0.200	0.281
**SDG 6**						1	0.251	0.377*	0.142	-0.126	0.225	-0.074	0.238	0.073	0.014	-0.030	0.164
**SDG 7**							1	0.074	0.120	0.091	0.190	0.062	0.185	0.067	0.025	0.047	0.117
**SDG 8**								1	0.192	0.060	0.438**	-0.198	-0.097	0.064	0.003	0.265	-0.062
**SDG 9**									1	0.016	0.144	-0.060	-0.235	-0.176	-0.082	0.161	0.090
**SDG 10**										1	-0.216	0.058	-0.096	-0.150	0.140	0.455**	0.017
**SDG 11**											1	-0.321*	-0.193	0.102	-0.163	0.197	-0.154
**SDG 12**												1	0.565**	0.234	-0.022	-0.127	-0.060
**SDG 13**													1	0.167	-0.116	-0.128	0.082
**SDG 14**														1	0.402*	-0.046	-0.022
**SDG 15**															1	0.025	0.170
**SDG 16**																1	0.111
**SDG 17**																	1

B3. Lower-middle-income countries

r	SDG 1	SDG 2	SDG 3	SDG 4	SDG 5	SDG 6	SDG 7	SDG 8	SDG 9	SDG 10	SDG 11	SDG 12	SDG 13	SDG 14	SDG 15	SDG 16	SDG 17
**SDG 1**	1	0.239	0.786**	0.708**	0.295	0.675**	0.730**	0.401**	0.586**	0.680**	0.454**	-0.350*	-0.474**	-0.613**	-0.122	0.555**	0.570**
**SDG 2**		1	0.194	0.422**	0.211	0.262	0.352*	0.155	0.429**	0.175	0.067	0.476**	0.019	-0.258	-0.156	0.142	-0.092
**SDG 3**			1	0.720**	0.366*	0.653**	0.778**	0.432**	0.489**	0.411**	0.675**	-0.459**	-0.457**	-0.383*	-0.142	0.600**	0.587**
**SDG 4**				1	0.387*	0.574**	0.691**	0.541**	0.659**	0.396*	0.451**	-0.332*	-0.610**	-0.288	-0.114	0.624**	0.481**
**SDG 5**					1	0.505**	0.337*	0.248	-0.088	-0.066	0.472**	-0.120	-0.393*	-0.197	-0.137	0.017	0.477**
**SDG 6**						1	0.648**	0.323*	0.386*	0.333*	0.476**	-0.189	-0.489**	-0.410*	-0.093	0.339*	0.506**
**SDG 7**							1	0.214	0.549**	0.394*	0.518**	-0.267	-0.567**	-0.517**	-0.013	0.499**	0.601**
**SDG 8**								1	0.443**	0.197	0.426**	-0.336*	-0.272	-0.011	-0.260	0.353*	0.154
**SDG 9**									1	0.397*	0.185	-0.122	-0.307	-0.381*	-0.124	0.574**	0.318*
**SDG 10**										1	0.153	-0.171	-0.186	-0.435*	-0.241	0.422**	0.178
**SDG 11**											1	-0.432**	-0.578**	-0.281	-0.171	0.380*	0.515**
**SDG 12**												1	0.552**	-0.063	-0.060	-0.475**	-0.573**
**SDG 13**													1	0.316	-0.071	-0.324*	-0.660**
**SDG 14**														1	0.050	-0.234	-0.357*
**SDG 15**															1	-0.017	0.213
**SDG 16**																1	0.291
**SDG 17**																	1

B4. Low-income countries

r	SDG 1	SDG 2	SDG 3	SDG 4	SDG 5	SDG 6	SDG 7	SDG 8	SDG 9	SDG 10	SDG 11	SDG 12	SDG 13	SDG 14	SDG 15	SDG 16	SDG 17
**SDG 1**	1	0.256	0.315	0.168	-0.082	0.359	0.490*	0.607**	0.415*	0.646**	0.254	0.201	-0.547**	0.072	0.049	0.343	0.169
**SDG 2**		1	0.552**	0.208	0.463**	0.301	-0.014	0.621**	0.625**	0.043	0.432*	0.265	-0.215	-0.036	0.106	0.523**	0.213
**SDG 3**			1	0.477**	0.389*	0.509**	0.283	0.515**	0.688**	0.091	0.466**	-0.076	-0.528**	-0.121	0.049	0.459*	0.429*
**SDG 4**				1	0.427*	0.310	0.350	0.131	0.435*	0.042	0.170	0.053	-0.410*	0.095	-0.179	0.194	0.261
**SDG 5**					1	0.276	0.002	0.362*	0.427*	-0.177	0.442*	0.179	-0.042	0.207	0.038	0.306	0.345
**SDG 6**						1	0.214	0.499**	0.458*	0.168	0.436*	0.013	-0.436*	-0.250	0.007	0.644**	0.350
**SDG 7**							1	0.161	0.344	0.189	0.205	0.032	-0.684**	0.039	-0.143	-0.017	-0.022
**SDG 8**								1	0.700**	0.298	0.512**	0.233	-0.449*	-0.264	0.202	0.670**	0.358
**SDG 9**									1	-0.055	0.383*	0.098	-0.605**	-0.064	0.110	0.362*	0.351
**SDG 10**										1	0.243	0.068	-0.030	0.061	-0.062	0.251	0.023
**SDG 11**											1	0.140	-0.292	-0.354	0.091	0.589**	0.297
**SDG 12**												1	-0.210	0.050	-0.235	0.139	0.283
**SDG 13**													1	0.232	0.088	-0.289	-0.384*
**SDG 14**														1	-0.143	-0.429	-0.225
**SDG 15**															1	0.149	0.117
**SDG 16**																1	0.543**
**SDG 17**																	1

C1. Slightly arid

r	SDG 1	SDG 2	SDG 3	SDG 4	SDG 5	SDG 6	SDG 7	SDG 8	SDG 9	SDG 10	SDG 11	SDG 12	SDG 13	SDG 14	SDG 15	SDG 16	SDG 17
**SDG 1**	1	0.568**	0.799**	0.746**	0.477**	0.701**	0.759**	0.725**	0.791**	0.527**	0.748**	-0.655**	-0.777**	-0.217	-0.023	0.678**	0.596**
**SDG 2**		1	0.564**	0.565**	0.368**	0.494**	0.503**	0.506**	0.679**	0.335*	0.463**	-0.413**	-0.491**	-0.227	-0.104	0.559**	0.419**
**SDG 3**			1	0.764**	0.644**	0.873**	0.860**	0.785**	0.838**	0.323*	0.796**	-0.723**	-0.831**	-0.130	0.017	0.681**	0.682**
**SDG 4**				1	0.528**	0.729**	0.761**	0.697**	0.777**	0.428**	0.685**	-0.537**	-0.776**	-0.121	0.033	0.660**	0.609**
**SDG 5**					1	0.599**	0.558**	0.605**	0.477**	0.124	0.604**	-0.478**	-0.459**	-0.096	-0.020	0.422**	0.651**
**SDG 6**						1	0.854**	0.714**	0.814**	0.171	0.753**	-0.630**	-0.748**	-0.011	0.031	0.625**	0.698**
**SDG 7**							1	0.673**	0.808**	0.275	0.755**	-0.714**	-0.838**	-0.165	0.059	0.589**	0.645**
**SDG 8**								1	0.773**	0.409**	0.640**	-0.583**	-0.694**	-0.101	0.176	0.693**	0.557**
**SDG 9**									1	0.368*	0.709**	-0.668**	-0.826**	-0.266	0.000	0.696**	0.530**
**SDG 10**										1	0.213	-0.268	-0.360*	-0.412*	0.116	0.471**	0.195
**SDG 11**											1	-0.661**	-0.764**	-0.106	0.004	0.663**	0.557**
**SDG 12**												1	0.787**	0.236	-0.129	-0.604**	-0.527**
**SDG 13**													1	0.254	-0.017	-0.649**	-0.548**
**SDG 14**														1	0.138	-0.292	-0.049
**SDG 15**															1	0.035	-0.077
**SDG 16**																1	0.428**
**SDG 17**																	1

C2. Moderately arid

r	SDG 1	SDG 2	SDG 3	SDG 4	SDG 5	SDG 6	SDG 7	SDG 8	SDG 9	SDG 10	SDG 11	SDG 12	SDG 13	SDG 14	SDG 15	SDG 16	SDG 17
**SDG 1**	1	0.668**	0.826**	0.747**	0.139	0.846**	0.805**	0.667**	0.867**	0.619**	0.723**	-0.846**	-0.854**	-0.918**	0.098	0.642**	0.132
**SDG 2**		1	0.774**	0.537*	0.358	0.663**	0.511*	0.593**	0.584**	0.419	0.646**	-0.614**	-0.586**	-0.645*	0.044	0.488*	-0.074
**SDG 3**			1	0.784**	0.263	0.782**	0.835**	0.716**	0.811**	0.668**	0.761**	-0.811**	-0.782**	-0.782**	0.042	0.863**	0.135
**SDG 4**				1	0.218	0.670**	0.693**	0.563*	0.746**	0.539*	0.721**	-0.772**	-0.730**	-0.691*	-0.012	0.753**	0.319
**SDG 5**					1	0.228	0.137	0.312	0.233	0.056	0.609**	-0.316	-0.246	-0.218	-0.046	0.333	0.130
**SDG 6**						1	0.800**	0.551*	0.839**	0.425	0.763**	-0.923**	-0.932**	-0.873**	0.284	0.658**	0.251
**SDG 7**							1	0.640**	0.707**	0.633**	0.782**	-0.835**	-0.849**	-0.827**	0.168	0.782**	0.300
**SDG 8**								1	0.663**	0.602**	0.686**	-0.649**	-0.549*	-0.382	-0.019	0.686**	-0.079
**SDG 9**									1	0.463*	0.647**	-0.823**	-0.847**	-0.855**	0.128	0.737**	0.093
**SDG 10**										1	0.551*	-0.505*	-0.426	-0.500	0.268	0.642**	0.037
**SDG 11**											1	-0.839**	-0.770**	-0.645*	0.151	0.726**	0.389
**SDG 12**												1	0.949**	0.745**	-0.293	-0.719**	-0.333
**SDG 13**													1	0.873**	-0.291	-0.704**	-0.370
**SDG 14**														1	0.045	-0.727*	-0.345
**SDG 15**															1	0.086	0.347
**SDG 16**																1	0.325
**SDG 17**																	1

C3. Severely arid

r	SDG 1	SDG 2	SDG 3	SDG 4	SDG 5	SDG 6	SDG 7	SDG 8	SDG 9	SDG 10	SDG 11	SDG 12	SDG 13	SDG 14	SDG 15	SDG 16	SDG 17
**SDG 1**	1	0.301	0.814**	0.723**	0.301	0.647**	0.781**	0.435*	0.701**	0.755**	0.661**	-0.634**	-0.755**	-0.459	-0.004	0.717**	0.100
**SDG 2**		1	0.629**	0.454*	0.190	0.227	0.580**	0.448*	0.605**	0.222	0.336	-0.242	-0.426*	-0.177	-0.121	0.546**	-0.203
**SDG 3**			1	0.830**	0.440*	0.700**	0.883**	0.681**	0.853**	0.614**	0.618**	-0.725**	-0.796**	-0.456*	-0.150	0.816**	-0.026
**SDG 4**				1	0.608**	0.653**	0.696**	0.649**	0.784**	0.470*	0.598**	-0.672**	-0.770**	-0.405	-0.041	0.644**	0.176
**SDG 5**					1	0.427*	0.253	0.428*	0.604**	-0.106	0.615**	-0.424*	-0.480*	-0.032	-0.035	0.305	0.316
**SDG 6**						1	0.644**	0.557**	0.540**	0.297	0.685**	-0.544**	-0.606**	-0.618**	-0.160	0.456*	0.395*
**SDG 7**							1	0.458*	0.748**	0.621**	0.563**	-0.625**	-0.655**	-0.549*	-0.164	0.701**	0.065
**SDG 8**								1	0.611**	0.188	0.459*	-0.559**	-0.531**	-0.472*	0.119	0.612**	-0.012
**SDG 9**									1	0.452*	0.640**	-0.729**	-0.818**	-0.079	-0.154	0.715**	0.078
**SDG 10**										1	0.280	-0.509*	-0.635**	-0.152	0.056	0.657**	-0.153
**SDG 11**											1	-0.386	-0.403*	-0.270	0.019	0.507**	0.493*
**SDG 12**												1	0.797**	0.260	0.074	-0.596**	0.118
**SDG 13**													1	0.126	0.231	-0.670**	-0.061
**SDG 14**														1	0.058	-0.004	0.089
**SDG 15**															1	0.080	0.030
**SDG 16**																1	0.020
**SDG 17**																	1

C4. Extremely arid

r	SDG 1	SDG 2	SDG 3	SDG 4	SDG 5	SDG 6	SDG 7	SDG 8	SDG 9	SDG 10	SDG 11	SDG 12	SDG 13	SDG 14	SDG 15	SDG 16	SDG 17
**SDG 1**	1	0.479	0.788**	0.700**	0.459	0.579*	0.809**	-0.215	0.824**	0.029	0.485	-0.350	-0.671**	-0.018	-0.115	0.644**	0.512*
**SDG 2**		1	0.624**	0.384	0.338	0.326	0.577**	0.414	0.651**	0.188	0.427	-0.320	-0.526*	-0.143	0.146	0.645**	0.496*
**SDG 3**			1	0.825**	0.541*	0.322	0.866**	0.212	0.890**	-0.256	0.529*	-0.764**	-0.851**	-0.099	-0.277	0.901**	0.549*
**SDG 4**				1	0.484*	0.358	0.767**	0.191	0.763**	-0.332	0.623**	-0.763**	-0.856**	-0.077	-0.165	0.740**	0.484*
**SDG 5**					1	0.738**	0.459*	0.256	0.514*	-0.368	0.620**	-0.564**	-0.629**	-0.152	0.047	0.665**	0.498*
**SDG 6**						1	0.280	0.182	0.421	-0.247	0.714**	-0.206	-0.365	-0.002	0.226	0.394	0.417
**SDG 7**							1	-0.039	0.794**	-0.188	0.448*	-0.708**	-0.823**	-0.112	-0.346	0.820**	0.469*
**SDG 8**								1	0.179	0.206	0.370	-0.086	-0.226	-0.015	0.152	0.347	0.200
**SDG 9**									1	-0.256	0.439	-0.750**	-0.776**	-0.051	-0.117	0.785**	0.657**
**SDG 10**										1	-0.350	0.444	0.385	-0.536	-0.082	-0.197	-0.159
**SDG 11**											1	-0.403	-0.544*	0.398	0.183	0.632**	0.326
**SDG 12**												1	0.844**	0.116	0.122	-0.771**	-0.487*
**SDG 13**													1	-0.020	0.086	-0.814**	-0.555*
**SDG 14**														1	0.240	-0.165	0.059
**SDG 15**															1	-0.114	0.191
**SDG 16**																1	0.531*
**SDG 17**																	1

Table S5. Projections of the mean index score (MIS) and sustainable development score (SDS) in 2030. N.A. is short for not available, because the amount of data in these countries is insufficient for regression analysis (less than 3). The value of SD ratio test below 0.35 is good for gray forecast model, 0.35~0.5 is qualified, 0.5~0.65 is barely qualified, and above 0.65 is not qualified.

Countries	Curvilinear regressions			Gray forecast model		average MIS	Curvilinear regressions			Gray forecast model		average SDS
	Model	MIS	p-value	MIS	SD ratio test		Model	SDS	p-value	SDS	SD ratio test	
Afghanistan	Logistic	58.96	<0.001	65.47	0.548	62.22	Compound/Logistic	52.45	<0.001	54.58	0.704	53.52
Albania	Linear	76.75	0.013	77.84	0.292	77.29	Compound/Logistic	75.01	<0.001	75.00	0.358	75.01
Algeria	Logistic	80.29	<0.001	81.80	0.648	81.05	Compound/Logistic	92.46	<0.001	93.97	0.195	93.22
Angola	Logistic	54.47	<0.001	54.67	0.902	54.57	Compound/Logistic	50.69	<0.001	51.10	0.896	50.89
Argentina	Logistic	76.27	<0.001	81.42	0.546	78.84	Compound/Logistic	74.36	<0.001	77.10	0.799	75.73
Armenia	Logistic	74.33	<0.001	82.08	0.326	78.21	Compound/Logistic	76.18	<0.001	81.08	0.362	78.63
Australia	Logistic	76.18	<0.001	84.42	0.068	80.30	Compound/Logistic	59.78	<0.001	61.77	0.734	60.77
Austria	Logistic	88.48	<0.001	92.00	0.611	90.24	Compound/Logistic	78.48	<0.001	81.40	0.400	79.94
Azerbaijan	Logistic	80.52	<0.001	83.75	0.499	82.13	Compound/Logistic	70.04	<0.001	74.35	0.878	72.19
Bahrain	Logistic	67.03	<0.001	61.97	0.805	64.50	Compound/Logistic	72.36	<0.001	66.91	0.807	69.64
Bangladesh	Logistic	85.27	<0.001	79.53	0.193	82.40	Compound/Logistic	79.16	<0.001	78.87	0.198	79.01
Barbados	N. A.	N. A.	N. A.	N. A.	N. A.	N. A.	N. A.	N. A.	N. A.	N. A.	N. A.	N. A.
Belarus	Logistic	90.28	<0.001	93.06	0.527	91.67	Compound/Logistic	88.08	<0.001	91.43	0.371	89.75
Belgium	Compound/Logistic	86.11	<0.001	92.05	0.437	89.08	Compound/Logistic	81.17	<0.001	95.08	0.692	88.12
Belize	Logistic	71.88	<0.001	83.68	0.319	77.78	Compound/Logistic	61.52	<0.001	69.19	0.351	65.35
Benin	Logistic	56.40	<0.001	56.30	0.882	56.35	Compound/Logistic	53.97	<0.001	56.59	0.881	55.28
Bhutan	Compound/Logistic	86.50	<0.001	88.47	0.321	87.49	Compound/Logistic	89.33	<0.001	91.38	0.168	90.35
Bolivia	Logistic	77.53	<0.001	68.61	0.392	73.07	Compound/Logistic	75.29	<0.001	70.15	0.340	72.72
Bosnia and Herzegovina	Compound/Logistic	99.80	<0.001	99.88	0.225	99.84	Compound/Logistic	94.31	<0.001	99.03	0.352	96.67
Botswana	Linear	70.24	<0.001	72.26	0.230	71.25	Compound/Logistic	63.47	<0.001	61.80	0.410	62.64
Brazil	Compound/Logistic	78.46	<0.001	78.78	0.587	78.62	Compound/Logistic	64.17	<0.001	63.31	0.980	63.74
Brunei Darussalam	N. A.	N. A.	N. A.	N. A.	N. A.	N. A.	N. A.	N. A.	N. A.	N. A.	N. A.	N. A.
Bulgaria	Compound/Logistic	78.59	<0.001	76.51	0.622	77.55	Compound/Logistic	83.41	<0.001	83.29	0.488	83.35
Burkina Faso	Logistic	63.19	<0.001	60.99	0.583	62.09	Compound/Logistic	52.71	<0.001	52.64	0.999	52.68
Burundi	Logistic	52.39	<0.001	55.84	0.869	54.12	Compound/Logistic	42.91	<0.001	45.56	0.330	44.24
Cabo Verde	Logistic	80.79	<0.001	84.12	0.169	82.45	Compound/Logistic	68.38	<0.001	74.23	0.168	71.30
Cambodia	Linear	80.27	0.004	80.67	0.194	80.47	Linear	76.49	0.003	76.03	0.139	76.26
Cameroon	Compound/Logistic	61.93	<0.001	54.81	0.306	58.37	Compound/Logistic	60.79	<0.001	57.12	0.233	58.96
Canada	Compound/Logistic	82.14	<0.001	86.15	0.197	84.14	Compound/Logistic	75.11	<0.001	77.13	0.530	76.12
Central African Republic	Compound/Logistic	35.64	<0.001	32.47	0.513	34.05	Compound/Logistic	38.92	<0.001	35.40	0.619	37.16
Chad	Compound/Logistic	36.37	<0.001	32.13	0.585	34.25	Compound/Logistic	37.93	<0.001	35.73	0.471	36.83
Chile	Compound/Logistic	94.47	<0.001	92.85	0.306	93.66	Compound/Logistic	73.30	<0.001	71.10	0.867	72.20
China	Compound/Logistic	88.31	<0.001	79.59	0.457	83.95	Compound/Logistic	83.84	<0.001	76.19	0.621	80.02
Colombia	Compound/Logistic	88.41	<0.001	84.54	0.315	86.48	Compound/Logistic	70.99	<0.001	67.88	0.806	69.43
Comoros	N. A.	N. A.	N. A.	N. A.	N. A.	N. A.	N. A.	N. A.	N. A.	N. A.	N. A.	N. A.
Congo, Dem. Rep.	Logistic	73.31	<0.001	77.08	0.328	75.19	Compound/Logistic	59.19	<0.001	59.66	0.294	59.42

Congo, Rep.	Compound/Logistic	61.46	<0.001	56.58	0.638	59.02	S-function	53.18	0.173	48.20	0.465	50.69
Costa Rica	Logistic	84.58	<0.001	75.55	0.393	80.07	Compound/Logistic	66.66	<0.001	62.26	0.852	64.46
Cote d'Ivoire	Compound/Logistic	69.73	<0.001	67.27	0.281	68.50	Compound/Logistic	62.97	<0.001	63.40	0.214	63.18
Croatia	Compound/Logistic	88.32	<0.001	92.18	0.185	90.25	Compound/Logistic	95.68	<0.001	100.00	0.223	97.84
Cuba	Compound/Logistic	79.07	<0.001	93.69	0.125	86.38	Compound/Logistic	75.57	<0.001	86.81	0.274	81.19
Cyprus	Compound/Logistic	88.40	<0.001	94.96	0.467	91.68	Compound/Logistic	87.60	<0.001	98.20	0.245	92.90
Czech Republic	Compound/Logistic	86.56	<0.001	93.37	0.652	89.97	Compound/Logistic	77.78	<0.001	87.15	0.628	82.46
Denmark	Logistic	86.17	<0.001	84.92	0.670	85.55	Compound/Logistic	77.23	<0.001	71.94	0.443	74.59
Djibouti	Logistic	67.39	<0.001	67.51	0.392	67.45	Compound/Logistic	63.01	<0.001	65.45	0.512	64.23
Dominican Republic	Logistic	81.85	<0.001	84.73	0.407	83.29	Compound/Logistic	77.33	<0.001	75.56	0.605	76.45
Ecuador	Logistic	84.35	<0.001	80.45	0.476	82.40	Compound/Logistic	75.00	<0.001	70.44	0.799	72.72
Egypt, Arab Rep.	Logistic	81.99	<0.001	88.50	0.343	85.24	Compound/Logistic	88.19	<0.001	95.37	0.108	91.78
El Salvador	Logistic	85.83	<0.001	83.77	0.446	84.80	Compound/Logistic	78.80	<0.001	77.81	0.461	78.30
Estonia	Logistic	88.76	<0.001	90.79	0.322	89.77	Compound/Logistic	78.28	<0.001	80.84	0.924	79.56
Eswatini	Compound/Logistic	56.93	0.005	49.77	0.001	53.35	Compound/Logistic	58.16	0.01	45.31	0.001	51.74
Ethiopia	Compound/Logistic	55.64	<0.001	56.72	0.840	56.18	Compound/Logistic	46.42	<0.001	48.41	0.509	47.42
Fiji	Compound	76.51	0.004	84.05	0.002	80.28	Compound/Logistic	82.15	0.005	93.50	0.003	87.82
Finland	Compound/Logistic	88.99	<0.001	94.71	0.466	91.85	Compound/Logistic	84.14	<0.001	89.61	0.501	86.88
Former Yugoslav Republic of Macedonia (FYROM)	N. A.	N. A.	N. A.	N. A.	N. A.	N. A.	N. A.	N. A.	N. A.	N. A.	N. A.	N. A.
France	Logistic	84.11	<0.001	82.44	0.355	83.27	Compound/Logistic	80.89	<0.001	82.27	0.337	81.58
Gabon	Compound/Logistic	59.47	<0.001	61.99	0.711	60.73	Compound/Logistic	74.72	<0.001	75.15	0.420	74.94
Gambia, The	Compound/Logistic	98.50	<0.001	90.51	0.123	94.50	Compound/Logistic	66.40	<0.001	69.79	0.209	68.09
Georgia	Compound/Logistic	80.47	<0.001	79.54	0.581	80.00	Compound/Logistic	80.11	<0.001	78.42	0.412	79.26
Germany	Compound/Logistic	81.75	<0.001	81.97	0.999	81.86	Compound/Logistic	81.83	<0.001	88.57	0.589	85.20
Ghana	Compound/Logistic	72.10	<0.001	64.26	0.558	68.18	Compound/Logistic	70.03	<0.001	63.89	0.617	66.96
Greece	Compound/Logistic	83.09	<0.001	93.44	0.228	88.27	Compound/Logistic	84.42	<0.001	95.96	0.392	90.19
Guatemala	Compound/Logistic	67.11	<0.001	67.48	0.653	67.29	Compound/Logistic	58.78	<0.001	58.21	0.972	58.49
Guinea	S-function	52.99	0.109	48.26	0.338	50.63	Compound/Logistic	51.82	<0.001	46.04	0.400	48.93
Guyana	Compound/Logistic	51.40	<0.001	53.36	0.377	52.38	Compound/Logistic	56.19	<0.001	60.41	0.678	58.30
Haiti	Compound/Logistic	72.25	<0.001	61.62	0.279	66.94	Compound/Logistic	67.28	<0.001	59.68	0.205	63.48
Honduras	S-function	64.10	0.155	61.96	0.548	63.03	Compound/Logistic	58.44	<0.001	56.65	0.806	57.55
Hungary	Compound/Logistic	86.00	<0.001	94.38	0.443	90.19	Compound/Logistic	78.19	<0.001	81.55	0.860	79.87
Iceland	Compound/Logistic	74.00	<0.001	72.18	0.535	73.09	Compound/Logistic	67.12	<0.001	65.43	0.719	66.27
India	Compound/Logistic	68.14	<0.001	64.73	0.639	66.43	Compound/Logistic	61.04	<0.001	60.52	0.984	60.78
Indonesia	Compound/Logistic	75.47	<0.001	78.08	0.060	76.77	Compound/Logistic	84.71	<0.001	89.41	0.172	87.06
Iran, Islamic Rep.	Compound/Logistic	90.06	<0.001	86.44	0.536	88.25	Compound/Logistic	98.35	<0.001	92.48	0.416	95.42
Iraq	Compound/Logistic	92.37	<0.001	100	0.396	96.18	Linear	92.79	0.043	100	0.431	96.40
Ireland	Compound/Logistic	88.04	<0.001	92.25	0.143	90.14	Compound/Logistic	80.63	<0.001	92.89	0.160	86.76
Israel	Compound/Logistic	87.78	<0.001	87.81	0.331	87.79	Compound/Logistic	78.53	<0.001	79.22	0.875	78.87
Italy	Compound/Logistic	87.14	<0.001	93.72	0.059	90.43	Compound/Logistic	85.07	<0.001	95.13	0.163	90.10
Jamaica	Compound/Logistic	76.65	<0.001	78.24	0.530	77.45	Compound/Logistic	88.11	<0.001	92.80	0.262	90.46
Japan	Compound/Logistic	79.31	<0.001	83.69	0.141	81.50	S-function	75.97	0.035	74.66	0.416	75.32
Jordan	Compound/Logistic	81.90	<0.001	88.34	0.355	85.12	Compound/Logistic	86.92	<0.001	94.78	0.217	90.85
Kazakhstan	Compound/Logistic	77.75	<0.001	89.25	0.294	83.50	Compound/Logistic	74.00	<0.001	82.90	0.457	78.45
Kenya	Compound/Logistic	76.49	<0.001	75.96	0.263	76.23	Compound/Logistic	60.56	<0.001	59.50	0.985	60.03
Korea, Rep.	S-function	79.17	<0.001	82.00	0.166	80.59	Power function	78.76	0.005	82.78	0.288	80.77

Kuwait	S-function	60.39	0.022	58.45	0.291	59.42	Compound/Logistic	64.15	<0.001	70.61	0.636	67.38
Kyrgyz Republic	Compound/Logistic	87.30	<0.001	89.56	0.332	88.43	Compound/Logistic	86.27	<0.001	86.43	0.476	86.35
Lao PDR	Compound/Logistic	68.32	<0.001	70.07	0.581	69.19	Compound/Logistic	60.59	<0.001	63.10	0.550	61.84
Latvia	Linear	88.72	0.016	93.08	0.213	90.90	Compound/Logistic	79.84	<0.001	78.54	0.774	79.19
Lebanon	Compound/Logistic	72.36	<0.001	73.93	0.224	73.14	Compound/Logistic	69.44	<0.001	73.56	0.251	71.50
Lesotho	Compound/Logistic	56.28	<0.001	63.14	0.540	59.71	Compound/Logistic	53.63	<0.001	64.51	0.499	59.07
Liberia	S-function	49.70	0.037	48.05	0.236	48.87	Compound/Logistic	53.05	<0.001	43.79	0.325	48.42
Lithuania	Compound/Logistic	84.21	<0.001	87.69	0.335	85.95	Compound/Logistic	80.59	<0.001	81.90	0.558	81.24
Luxembourg	Compound/Logistic	74.33	<0.001	70.93	0.378	72.63	Compound/Logistic	60.60	<0.001	58.61	0.435	59.61
Madagascar	Logistic	65.99	<0.001	62.88	0.230	64.43	Compound/Logistic	55.42	<0.001	52.37	0.268	53.89
Malawi	S-function	51.69	0.028	52.16	0.431	51.92	Compound/Logistic	48.89	<0.001	46.37	0.364	47.63
Malaysia	Compound/Logistic	75.09	<0.001	75.81	0.698	75.45	Compound/Logistic	71.71	<0.001	67.36	0.181	69.54
Maldives	Compound/Logistic	56.92	0.016	86.41	0.001	71.66	S-function	62.47	0.578	100	0.005	81.24
Mali	Power function	52.63	0.002	55.86	0.105	54.24	Compound/Logistic	52.14	<0.001	53.30	0.498	52.72
Malta	Compound/Logistic	74.99	<0.001	80.35	0.564	77.67	Compound/Logistic	73.46	<0.001	76.28	0.732	74.87
Mauritania	Compound/Logistic	72.91	<0.001	74.11	0.525	73.51	Compound/Logistic	73.00	<0.001	71.78	0.505	72.39
Mauritius	Compound/Logistic	74.07	<0.001	72.30	0.583	73.18	Compound/Logistic	75.05	<0.001	68.47	0.281	71.76
Mexico	Compound/Logistic	74.51	<0.001	84.06	0.604	79.28	Compound/Logistic	57.07	<0.001	67.79	0.587	62.43
Moldova	S-function	75.61	0.376	74.40	0.859	75.01	Compound/Logistic	70.00	<0.001	67.42	0.690	68.71
Mongolia	Compound/Logistic	64.70	<0.001	63.50	0.970	64.10	Compound/Logistic	67.42	<0.001	66.99	0.529	67.20
Montenegro	Compound/Logistic	73.08	<0.001	73.36	0.823	73.22	Compound/Logistic	74.15	<0.001	77.21	0.339	75.68
Morocco	Compound/Logistic	84.33	<0.001	86.75	0.421	85.54	Compound/Logistic	89.34	<0.001	92.14	0.229	90.74
Mozambique	Compound/Logistic	60.09	<0.001	54.62	0.718	57.36	S-function	52.43	0.4	48.64	0.714	50.53
Myanmar	Compound/Logistic	82.89	<0.001	87.53	0.270	85.21	Compound/Logistic	75.00	<0.001	74.65	0.250	74.83
Namibia	Compound/Logistic	69.36	<0.001	72.33	0.260	70.84	Compound/Logistic	64.49	<0.001	67.06	0.288	65.77
Nepal	Compound/Exponential/Logistic	82.58	<0.001	81.53	0.215	82.05	Compound/Logistic	79.89	<0.001	80.82	0.126	80.35
Netherlands	Compound/Logistic	85.01	<0.001	87.29	0.317	86.15	Compound/Logistic	80.47	<0.001	88.77	0.064	84.62
New Zealand	Compound/Logistic	82.39	<0.001	81.17	0.781	81.78	Compound/Logistic	85.77	<0.001	82.18	0.599	83.97
Nicaragua	Compound/Logistic	76.83	<0.001	67.77	0.474	72.30	S-function	62.50	0.041	61.18	0.355	61.84
Niger	S-function	50.11	0.023	48.52	0.207	49.31	Compound/Logistic	47.64	<0.001	43.92	0.379	45.78
Nigeria	Compound/Logistic	50.78	<0.001	56.25	0.692	53.52	Compound/Logistic	54.81	<0.001	61.91	0.486	58.36
North Macedonia	Compound/Logistic	84.17	<0.001	85.19	0.001	84.68	Compound/Logistic	76.13	0.003	70.82	0.001	73.47
Norway	Compound/Logistic	77.12	<0.001	83.84	0.323	80.48	Power function	68.08	0.005	63.93	0.263	66.00
Oman	Compound/Logistic	83.37	<0.001	87.91	0.229	85.64	Compound/Logistic	83.97	<0.001	88.31	0.458	86.14
Pakistan	Compound/Logistic	62.29	<0.001	66.55	0.224	64.42	Compound/Logistic	61.39	<0.001	66.63	0.493	64.01
Panama	Compound	81.59	<0.001	81.02	0.423	81.30	Compound/Logistic	68.12	<0.001	67.81	0.933	67.97
Papua New Guinea	Compound/Logistic	50.13	0.001	48.45	0.001	49.29	Compound/Logistic	48.70	<0.001	48.86	0.001	48.78
Paraguay	S-function	68.22	0.107	67.34	0.591	67.78	Compound/Logistic	68.62	<0.001	66.08	0.808	67.35
Peru	Compound/Logistic	86.48	<0.001	80.02	0.353	83.25	S-function	69.24	0.032	74.41	0.506	71.82
Philippines	S-function	65.16	0.331	63.94	0.724	64.55	Compound/Logistic	63.36	<0.001	59.66	0.890	61.51
Poland	Compound/Logistic	92.71	<0.001	100	0.026	96.36	Compound/Logistic	100	<0.001	100	0.136	100.00
Portugal	Compound/Logistic	87.96	<0.001	94.17	0.212	91.06	Compound/Logistic	77.78	<0.001	76.82	0.924	77.30
Qatar	Compound/Logistic	70.04	<0.001	76.19	0.613	73.12	Compound/Logistic	58.31	<0.001	60.86	0.950	59.58
Romania	Compound/Logistic	79.57	<0.001	88.75	0.275	84.16	Compound/Logistic	86.81	<0.001	98.99	0.348	92.90
Russian Federation	Compound/Logistic	86.21	<0.001	89.65	0.108	87.93	Compound/Exponential/Logistic	93.78	<0.001	94.22	0.099	94.00
Rwanda	Power function	57.71	0.032	58.94	0.344	58.32	Compound/Logistic	54.31	<0.001	55.43	0.846	54.87
Sao Tome and Principe	Compound/Logistic	36.29	<0.001	33.53	0.003	34.91	Power function	53.07	0.03	45.02	0.001	49.04

Saudi Arabia	Compound/Logistic	69.20	<0.001	70.89	0.429	70.04	Power function	71.22	0.024	70.46	0.102	70.84
Senegal	Compound/Logistic	63.85	<0.001	63.01	0.205	63.43	Compound/Logistic	57.24	<0.001	58.93	0.930	58.08
Serbia	Compound/Logistic	86.73	<0.001	92.63	0.274	89.68	Compound/Logistic	88.96	<0.001	95.02	0.207	91.99
Sierra Leone	Compound/Logistic	64.89	<0.001	62.68	0.306	63.78	Compound/Logistic	53.98	<0.001	55.10	0.680	54.54
Singapore	Compound/Logistic	69.85	<0.001	66.04	0.905	67.95	Compound/Logistic	60.22	<0.001	61.81	0.834	61.02
Slovak Republic	Compound	90.08	<0.001	96.65	0.145	93.37	Compound/Logistic	85.63	<0.001	89.68	0.344	87.66
Slovenia	Compound/Logistic	82.40	<0.001	85.94	0.698	84.17	Compound/Logistic	78.18	<0.001	89.63	0.782	83.91
Somalia	N. A.	N. A.	N. A.	N. A.	N. A.	N. A.	N. A.	N. A.	N. A.	N. A.	N. A.	N. A.
South Africa	Compound/Logistic	71.01	<0.001	74.61	0.249	72.81	Compound/Logistic	63.12	<0.001	65.80	0.542	64.46
South Sudan	N. A.	N. A.	N. A.	N. A.	N. A.	N. A.	N. A.	N. A.	N. A.	N. A.	N. A.	N. A.
Spain	Compound	86.94	<0.001	91.80	0.300	89.37	Compound/Logistic	85.35	<0.001	86.50	0.501	85.92
Sri Lanka	Compound/Logistic	74.00	<0.001	79.76	0.041	76.88	Compound/Logistic	81.41	<0.001	88.99	0.262	85.20
Sudan	Compound/Logistic	49.10	<0.001	47.87	0.953	48.48	Power function	47.09	0.006	41.65	0.255	44.37
Suriname	Compound/Logistic	69.83	<0.001	75.31	0.527	72.57	Compound/Logistic	65.57	<0.001	73.20	0.447	69.39
Swaziland	N. A.	N. A.	N. A.	N. A.	N. A.	N. A.	N. A.	N. A.	N. A.	N. A.	N. A.	N. A.
Sweden	Compound/Logistic	84.91	<0.001	86.80	0.675	85.86	Compound/Logistic	79.37	<0.001	85.53	0.788	82.45
Switzerland	Compound/Logistic	81.19	<0.001	84.02	0.227	82.61	Compound/Logistic	76.01	<0.001	80.15	0.771	78.08
Syrian Arab Republic	Compound/Logistic	53.87	<0.001	60.06	0.517	56.97	Compound/Logistic	50.27	<0.001	57.21	0.424	53.74
Tajikistan	Compound/Logistic	81.40	<0.001	79.50	0.560	80.45	Compound/Logistic	65.05	<0.001	67.44	0.776	66.24
Tanzania	Compound/Logistic	67.84	<0.001	61.12	0.139	64.48	Compound/Logistic	60.52	<0.001	56.51	0.423	58.52
Thailand	Compound/Logistic	90.11	<0.001	91.88	0.412	90.99	Compound/Logistic	92.77	<0.001	95.69	0.247	94.23
Timor-Leste	N. A.	N. A.	N. A.	N. A.	N. A.	N. A.	N. A.	N. A.	N. A.	N. A.	N. A.	N. A.
Togo	Power function	54.93	0.018	57.67	0.281	56.30	Compound/Logistic	55.63	<0.001	55.17	0.299	55.40
Trinidad and Tobago	Compound/Logistic	60.69	<0.001	60.30	0.694	60.50	Compound/Logistic	58.94	<0.001	59.51	0.616	59.23
Tunisia	Compound/Logistic	82.45	<0.001	90.14	0.440	86.30	Compound/Logistic	86.70	<0.001	93.63	0.242	90.16
Turkey	Compound/Logistic	78.26	<0.001	86.41	0.355	82.33	Compound/Logistic	70.79	<0.001	81.61	0.430	76.20
Turkmenistan	Compound/Logistic	74.43	<0.001	63.89	0.624	69.16	Power function	64.73	0.036	75.32	0.496	70.03
Uganda	Compound/Logistic	50.20	<0.001	46.46	0.654	48.33	Compound/Logistic	51.09	<0.001	51.00	0.990	51.04
Ukraine	Compound/Logistic	82.16	<0.001	86.18	0.172	84.17	Compound/Logistic	80.03	<0.001	85.00	0.238	82.52
United Arab Emirates	Compound/Logistic	77.82	<0.001	72.84	0.384	75.33	Compound/Logistic	70.01	<0.001	66.33	0.367	68.17
United Kingdom	Compound/Exponential/Logistic	84.34	<0.001	84.12	0.182	84.23	Compound/Logistic	88.65	<0.001	88.68	0.202	88.67
United States	Compound/Exponential/Logistic	87.10	<0.001	87.15	0.323	87.12	Compound/Logistic	84.10	<0.001	88.83	0.401	86.46
Uruguay	Linear	84.51	0.024	89.27	0.266	86.89	Compound/Logistic	83.33	<0.001	90.05	0.311	86.69
Uzbekistan	Compound/Logistic	71.39	<0.001	70.82	0.996	71.10	Compound/Logistic	72.12	<0.001	67.48	0.645	69.80
Vanuatu	Compound/Logistic	63.78	<0.001	57.37	0.001	60.57	Compound/Logistic	58.59	0.011	76.74	0.002	67.67
Venezuela, RB	Linear	45.91	0.001	47.82	0.154	46.87	Compound/Logistic	47.82	<0.001	48.07	0.386	47.95
Vietnam	Compound/Logistic	88.36	<0.001	85.82	0.344	87.09	Compound/Logistic	98.70	<0.001	100.00	0.327	99.35
Yemen, Rep.	Compound/Logistic	55.58	<0.001	64.21	0.747	59.90	Compound/Logistic	55.35	<0.001	59.60	0.802	57.47
Zambia	Compound/Logistic	54.06	<0.001	49.93	0.409	51.99	Compound/Logistic	40.87	<0.001	39.42	0.316	40.15
Zimbabwe	Compound/Logistic	71.61	<0.001	63.20	0.498	67.40	Compound/Logistic	58.91	<0.001	57.83	0.626	58.37

Table S6. Details for priority selections. EHN, EP, SD, and ED refer to SDGs related to essential human needs, eco-environmental protection, social development, and economic development, respectively. UD, UE, and SS stand for relatively underdeveloped, uneven, and sustainable status. N.A. is short for not available.

Countries	2017				2021				Development status in 2021	Development pathway	Best performance in 2021	Worst performance in 2021	Change in EHN
	EHN	EP	SD	ED	EHN	EP	SD	ED					
Afghanistan	45.52	69.10	48.27	24.25	49.59	80.96	44.10	25.35	UD & UE	Progression	EP	ED	4.08
Albania	77.58	65.83	70.53	55.82	79.62	74.05	70.11	54.17	SS	Progression	EHN	ED	2.04
Algeria	74.51	69.37	72.68	53.16	75.37	70.42	76.14	55.11	SS	Progression	SD	ED	0.86
Angola	44.50	68.25	49.60	36.54	42.43	80.11	43.14	35.57	UD & UE	Retrogression in ES	EP	ED	-2.07
Argentina	86.16	65.85	66.09	69.01	83.14	70.15	68.87	65.65	SS	Progression	EHN	ED	-3.02
Armenia	79.85	75.24	71.72	54.64	79.17	81.18	69.02	59.04	SS	Progression	EP	ED	-0.68
Australia	87.15	48.93	78.68	88.33	88.05	44.22	81.62	86.52	UE	Retrogression in both	EHN	EP	0.90
Austria	90.98	65.62	78.65	85.88	89.93	58.45	87.02	90.63	SS	Retrogression in ES	ED	EP	-1.05
Azerbaijan	78.31	73.66	68.55	59.22	78.80	79.56	70.56	62.20	SS	Retrogression in ES	EP	ED	0.48
Bahrain	78.15	48.80	62.20	66.25	81.43	52.93	63.56	57.60	SS	Retrogression in MIS	EHN	EP	3.28
Bangladesh	63.97	63.62	58.51	29.54	62.33	77.65	61.52	49.64	UD & UE	Progression	EP	ED	-1.65
Barbados	80.85	47.64	61.69	72.81	77.04	60.66	80.85	61.49	SS	Retrogression in ES	SD	EP	-3.81
Belarus	82.11	75.14	77.09	70.86	83.79	84.62	83.81	63.08	SS	Progression	EP	ED	1.68
Belgium	89.01	62.85	82.56	83.34	86.96	66.01	86.11	89.29	SS	Progression	ED	EP	-2.05
Belize	81.01	54.00	63.40	60.59	71.05	71.36	66.60	53.43	UE	Retrogression in ES	EP	ED	-9.96
Benin	43.31	66.15	48.19	39.60	37.85	80.18	41.53	43.39	UD & UE	Retrogression in ES	EP	EHN	-5.46
Bhutan	75.16	76.31	59.98	47.82	71.58	78.84	69.41	62.52	SS	Progression	EP	ED	-3.58
Bolivia	72.04	78.49	57.21	51.13	71.05	81.75	63.81	55.62	UE	Progression	EP	ED	-0.99
Bosnia and Herzegovina	79.25	57.42	68.17	48.65	80.17	77.34	73.41	58.55	SS	Progression	EHN	ED	0.93
Botswana	58.58	59.87	58.04	56.47	56.01	75.40	59.05	61.07	UD & UE	Progression	EP	EHN	-2.58
Brazil	84.31	69.09	56.07	67.77	83.15	72.12	61.53	66.96	UE	Retrogression in ES	EHN	SD	-1.16
Brunei Darussalam	N. A.	N. A.	N. A.	N. A.	77.03	46.21	73.86	77.97	UE	N. A.	ED	EP	N. A.
Bulgaria	80.43	73.14	70.17	62.37	81.05	76.32	66.86	70.00	SS	Progression	EHN	SD	0.62
Burkina Faso	43.98	82.81	48.54	29.13	35.76	90.58	50.74	45.80	UD & UE	Retrogression in ES	EP	EHN	-8.22
Burundi	33.60	78.17	65.44	32.83	31.10	86.61	58.36	35.14	UD & UE	Retrogression in both	EP	EHN	-2.50
Cabo Verde	N. A.	N. A.	N. A.	N. A.	69.86	76.48	64.69	59.93	SS	N. A.	EP	ED	N. A.
Cambodia	63.25	54.56	61.92	48.28	66.54	73.02	63.45	51.72	UE	Progression	EP	ED	3.28
Cameroon	56.97	70.87	53.01	21.55	51.33	83.00	46.45	39.51	UD & UE	Progression	EP	ED	-5.64
Canada	87.50	56.21	81.10	86.14	88.33	55.53	84.43	86.58	SS	Retrogression in ES	EHN	EP	0.83
Central African Republic	24.68	78.62	32.28	22.44	23.60	87.44	26.36	23.49	UD & UE	Retrogression in both	EP	ED	-1.08
Chad	30.16	80.48	39.00	25.58	23.30	87.42	31.57	30.26	UD & UE	Retrogression in both	EP	EHN	-6.86
Chile	87.19	66.07	62.29	68.31	88.16	75.24	68.69	75.35	UE	Retrogression in ES	EHN	SD	0.97
China	80.34	55.78	64.99	63.75	81.64	69.47	63.28	74.19	SS	Progression	EHN	SD	1.29
Colombia	80.09	62.69	55.36	57.87	80.02	77.30	61.22	61.34	UE	Retrogression in ES	EHN	SD	-0.07
Congo, Dem. Rep.	34.67	61.54	45.89	25.39	33.04	85.49	45.15	35.05	UD & UE	Progression	EP	EHN	-1.63
Congo, Rep.	43.86	73.05	53.32	28.97	41.04	86.71	46.54	38.45	UD & UE	Progression	EP	ED	-2.82
Costa Rica	81.14	67.97	64.74	65.63	81.53	71.10	68.64	71.71	UE	Retrogression in ES	EHN	SD	0.39
Cote d'Ivoire	56.32	67.49	46.22	41.36	51.00	86.00	46.27	49.40	UD & UE	Retrogression in ES	EP	SD	-5.32
Croatia	87.30	76.04	71.73	69.23	87.82	78.83	78.16	73.72	SS	Progression	EHN	ED	0.52

Cuba	84.45	65.24	84.64	62.32	75.96	80.07	85.31	60.00	UE	Progression	SD	ED	-8.50
Cyprus	84.71	58.25	67.48	68.73	83.98	62.81	75.29	75.05	SS	Progression	EHN	EP	-0.73
Czech Republic	87.89	79.84	78.34	79.90	85.81	76.01	81.04	85.98	SS	Retrogression in ES	ED	EP	-2.08
Denmark	90.22	65.11	90.06	89.65	87.49	67.03	91.92	92.49	SS	Progression	ED	EP	-2.73
Djibouti	59.10	46.65	49.24	38.24	49.19	72.87	46.12	48.66	UD & UE	Progression	EP	SD	-9.91
Dominican Republic	76.24	72.82	60.15	56.53	76.23	84.11	59.12	63.24	UE	Progression	EP	SD	0.00
Ecuador	78.02	67.83	65.87	60.98	77.63	79.51	67.69	62.84	UE	Retrogression in ES	EP	ED	-0.39
Egypt, Arab Rep.	76.63	66.51	64.81	43.44	73.74	75.46	65.32	56.63	SS	Progression	EP	ED	-2.89
El Salvador	78.00	60.77	58.05	48.78	77.36	68.71	65.00	56.02	UE	Progression	EHN	ED	-0.64
Estonia	85.57	72.28	75.97	79.57	85.76	75.95	80.30	84.24	SS	Retrogression in ES	EHN	EP	0.19
Eswatini	N. A.	N. A.	N. A.	N. A.	45.55	73.50	50.39	46.14	UD & UE	N. A.	EP	EHN	N. A.
Ethiopia	51.50	69.57	54.91	38.39	43.45	84.53	52.11	42.48	UD & UE	Retrogression in ES	EP	ED	-8.05
Fiji	N. A.	N. A.	N. A.	N. A.	73.14	71.89	68.86	71.20	SS	N. A.	EHN	SD	N. A.
Finland	90.37	66.45	89.20	88.22	89.94	71.21	90.44	91.19	SS	Progression	ED	EP	-0.43
France	90.88	66.92	78.00	84.43	89.93	65.58	83.47	86.38	SS	Progression	EHN	EP	-0.96
Gabon	70.77	73.87	64.41	45.32	66.69	80.99	52.48	49.34	UD	Retrogression in MIS	EP	ED	-4.07
Gambia, The	44.91	66.32	47.91	27.84	51.13	86.74	53.14	46.39	UD & UE	Retrogression in ES	EP	ED	6.22
Georgia	77.33	64.99	70.95	54.83	76.28	75.99	71.57	61.59	SS	Progression	EHN	ED	-1.05
Germany	90.13	66.48	82.07	87.20	88.81	64.49	85.71	90.55	SS	Progression	ED	EP	-1.32
Ghana	62.22	70.73	58.65	43.67	60.25	79.10	57.24	52.85	UD & UE	Progression	EP	ED	-1.97
Greece	86.83	62.12	69.69	69.34	86.06	67.38	73.40	71.71	SS	Progression	EHN	EP	-0.77
Guatemala	67.61	59.70	51.81	51.87	65.19	72.54	48.11	53.96	UD & UE	Retrogression in ES	EP	SD	-2.42
Guinea	39.60	74.32	47.08	32.87	36.29	81.94	47.02	40.69	UD & UE	Retrogression in ES	EP	EHN	-3.31
Guyana	79.07	56.74	63.72	52.78	71.29	42.68	70.53	49.00	UD & UE	Retrogression in MIS	EHN	EP	-7.78
Haiti	38.25	60.83	46.13	28.06	39.37	78.46	51.60	34.75	UD & UE	Progression	EP	ED	1.12
Honduras	70.91	66.32	57.26	47.33	66.68	77.75	51.52	55.04	UD & UE	Retrogression in ES	EP	SD	-4.22
Hungary	86.48	80.36	71.94	71.60	87.13	79.76	72.89	78.82	SS	Progression	EHN	SD	0.65
Iceland	92.65	51.08	87.75	80.54	85.63	49.28	89.45	85.47	SS	Retrogression in both	SD	EP	-7.03
India	62.63	61.52	58.46	45.21	59.21	75.18	51.61	55.49	UD & UE	Retrogression in ES	EP	SD	-3.42
Indonesia	69.74	64.12	62.42	50.59	69.11	71.90	63.27	59.45	SS	Progression	EP	ED	-0.63
Iran, Islamic Rep.	75.02	62.71	60.21	57.64	77.23	76.69	62.48	61.65	SS	Progression	EHN	ED	2.21
Iraq	67.16	53.55	58.66	39.81	70.83	68.96	63.41	45.94	UD	Progression	EHN	ED	3.67
Ireland	88.78	63.83	75.13	83.26	89.93	66.09	80.86	86.03	SS	Progression	EHN	EP	1.15
Israel	84.02	49.15	66.44	81.14	84.81	54.01	74.12	88.33	SS	Progression	ED	EP	0.79
Italy	87.51	64.54	71.25	77.15	87.82	68.55	76.60	80.84	SS	Progression	EHN	EP	0.31
Jamaica	78.61	59.22	65.42	58.22	75.68	68.78	66.55	62.07	SS	Progression	EHN	ED	-2.93
Japan	90.06	64.85	75.78	91.50	89.40	62.15	79.93	87.39	SS	Retrogression in both	EHN	EP	-0.66
Jordan	75.34	58.85	68.81	55.05	76.21	77.76	63.36	61.16	SS	Progression	EP	ED	0.86
Kazakhstan	77.92	58.75	74.28	66.74	80.64	64.32	72.99	65.98	SS	Progression	EHN	EP	2.71
Kenya	51.78	61.92	55.91	49.22	52.03	78.50	57.65	55.93	UD & UE	Retrogression in ES	EP	EHN	0.25
Korea, Rep.	87.86	60.63	69.06	83.21	88.29	61.23	76.84	88.51	SS	Progression	ED	EP	0.43
Kuwait	77.00	39.92	72.93	53.99	73.58	40.78	71.48	53.80	UD	Retrogression in MIS	EHN	EP	-3.42
Kyrgyz Republic	80.60	71.97	74.50	46.43	75.30	83.36	77.80	61.19	SS	Progression	EP	ED	-5.30
Lao PDR	62.15	70.63	62.61	48.76	60.99	79.79	59.35	56.50	UD & UE	Retrogression in ES	EP	ED	-1.17
Latvia	84.31	71.55	70.61	72.69	86.57	77.02	73.70	78.71	SS	Progression	EHN	SD	2.27
Lebanon	77.71	59.59	58.78	60.99	77.74	71.52	61.42	51.44	SS	Progression	EHN	ED	0.03
Lesotho	43.34	73.11	58.32	39.96	40.31	85.95	54.21	43.16	UD & UE	Retrogression in ES	EP	EHN	-3.03
Liberia	28.80	64.49	44.11	35.16	32.79	82.49	48.19	30.71	UD & UE	Retrogression in ES	EP	ED	3.99

Lithuania	81.44	67.28	70.55	74.18	81.93	76.65	72.31	75.37	SS	Progression	EHN	SD	0.49
Luxembourg	83.04	52.66	75.16	83.65	80.22	42.90	82.64	85.06	UE	Retrogression in both	ED	EP	-2.82
Madagascar	23.76	67.19	51.06	32.22	30.76	76.16	50.07	41.44	UD & UE	Progression	EP	EHN	7.00
Malawi	37.10	71.46	53.72	33.28	34.23	82.81	52.01	41.90	UD & UE	Retrogression in ES	EP	EHN	-2.87
Malaysia	81.92	57.40	63.78	75.49	79.02	63.85	64.40	77.52	SS	Progression	EHN	EP	-2.90
Maldives	N. A.	N. A.	N. A.	N. A.	79.64	66.96	63.01	65.51	SS	N. A.	EHN	SD	N. A.
Mali	42.64	75.94	46.76	33.95	39.92	82.63	46.57	46.13	UD & UE	Retrogression in ES	EP	EHN	-2.72
Malta	83.81	70.78	75.67	76.28	85.84	59.86	76.01	79.66	SS	Retrogression in MIS	EHN	EP	2.03
Mauritania	57.60	64.94	49.15	25.30	51.26	81.70	51.52	34.29	UD & UE	Progression	EP	ED	-6.34
Mauritius	74.99	37.49	67.76	64.13	80.39	53.49	63.35	67.13	UE	Progression	EHN	EP	5.40
Mexico	80.42	65.34	62.70	66.11	78.86	69.62	61.46	65.05	UE	Retrogression in both	EHN	SD	-1.56
Moldova	80.00	79.06	77.25	54.67	79.42	78.64	78.82	55.51	UE	Progression	EHN	ED	-0.58
Mongolia	65.27	66.57	70.49	49.46	65.77	58.95	73.72	49.79	UD & UE	Retrogression in MIS	SD	ED	0.50
Montenegro	79.90	46.30	78.17	56.16	75.27	58.78	71.98	62.71	SS	Progression	EHN	EP	-4.62
Morocco	75.49	68.33	61.44	58.42	76.94	80.22	62.53	60.25	SS	Progression	EP	ED	1.45
Mozambique	35.15	72.65	55.70	30.74	35.91	79.02	49.14	42.21	UD & UE	Retrogression in ES	EP	EHN	0.76
Myanmar	63.55	62.24	73.32	30.87	64.96	77.05	65.03	48.65	UE	Progression	EP	ED	1.41
Namibia	54.87	69.36	63.23	46.56	50.64	81.28	62.92	52.40	UD & UE	Progression	EP	EHN	-4.23
Nepal	66.58	80.19	64.82	29.17	61.68	86.88	70.83	48.97	UE	Progression	EP	ED	-4.89
Netherlands	89.23	60.53	80.99	88.59	88.68	61.10	84.66	91.79	SS	Progression	ED	EP	-0.55
New Zealand	88.99	54.75	82.12	83.16	89.58	54.78	83.24	87.34	SS	Progression	EHN	EP	0.59
Nicaragua	72.31	67.73	59.52	47.59	70.88	79.28	59.58	52.50	UE	Progression	EP	ED	-1.43
Niger	34.57	70.19	49.66	28.36	32.94	86.95	46.49	38.65	UD & UE	Retrogression in ES	EP	EHN	-1.63
Nigeria	44.89	69.39	45.89	31.84	40.20	82.73	36.79	38.61	UD & UE	Progression	EP	SD	-4.69
North Macedonia	N. A.	N. A.	N. A.	N. A.	78.44	81.70	71.90	59.11	SS	N. A.	EP	ED	N. A.
Norway	89.63	62.63	91.30	90.59	87.21	53.01	94.64	90.78	SS	Retrogression in both	SD	EP	-2.42
Oman	73.26	57.12	62.96	60.75	70.88	65.77	70.99	67.75	SS	Progression	SD	EP	-2.38
Pakistan	63.34	67.77	49.91	36.13	56.62	79.20	50.74	42.56	UD & UE	Progression	EP	ED	-6.72
Panama	77.61	61.29	51.84	64.85	78.69	71.89	54.99	66.58	UE	Retrogression in ES	EHN	SD	1.08
Papua New Guinea	N. A.	N. A.	N. A.	N. A.	35.69	77.61	46.18	50.95	UD & UE	N. A.	EP	EHN	N. A.
Paraguay	83.55	66.81	57.24	51.27	81.15	71.45	54.12	61.52	UE	Progression	EHN	SD	-2.40
Peru	78.74	66.35	61.40	51.79	80.54	78.78	63.14	58.33	SS	Progression	EHN	ED	1.80
Philippines	70.81	68.21	62.70	51.08	65.95	78.86	58.54	52.90	UE	Retrogression in ES	EP	ED	-4.85
Poland	85.13	67.64	74.10	73.88	85.41	77.10	77.95	79.53	SS	Progression	EHN	EP	0.29
Portugal	87.62	60.21	74.57	77.62	86.72	63.65	80.54	82.00	SS	Retrogression in ES	EHN	EP	-0.90
Qatar	74.94	48.65	65.16	59.21	75.97	54.14	67.70	61.57	UE	Retrogression in ES	EHN	EP	1.03
Romania	80.74	75.33	71.75	65.48	83.23	78.22	65.34	72.95	SS	Progression	EHN	SD	2.49
Russian Federation	75.80	69.94	61.01	69.34	79.80	67.15	71.42	76.34	SS	Progression	EHN	EP	4.00
Rwanda	47.42	76.25	60.80	36.76	42.40	80.83	61.10	50.27	UD & UE	Retrogression in ES	EP	EHN	-5.02
Sao Tome and Principe	N. A.	N. A.	N. A.	N. A.	48.83	83.55	56.21	46.68	UD & UE	N. A.	EP	ED	N. A.
Saudi Arabia	72.61	53.18	75.24	42.03	69.06	57.84	66.85	64.56	SS	Progression	EHN	EP	-3.56
Senegal	57.64	65.67	53.40	46.06	53.26	81.64	48.59	52.51	UD & UE	Retrogression in ES	EP	SD	-4.38
Serbia	83.14	68.97	76.02	58.18	82.33	74.00	77.12	68.98	SS	Progression	EHN	ED	-0.80
Sierra Leone	29.33	69.49	57.42	29.73	31.28	79.07	60.35	34.79	UD & UE	Retrogression in ES	EP	EHN	1.95
Singapore	88.66	34.69	63.35	91.24	85.64	36.71	75.79	85.04	UE	Retrogression in ES	EHN	EP	-3.03
Slovak Republic	88.04	69.80	74.37	69.83	86.95	76.33	78.75	77.30	SS	Progression	EHN	EP	-1.09
Slovenia	88.35	71.27	83.82	74.44	87.41	68.25	85.09	83.90	SS	Progression	EHN	EP	-0.93

Somalia	N. A.	N. A.	N. A.	N. A.	25.53	75.75	44.80	37.31	UD & UE	N. A.	EP	EHN	N. A.
South Africa	64.25	63.45	60.40	54.71	60.00	71.11	59.64	66.99	UD & UE	Progression	EP	SD	-4.25
South Sudan	N. A.	N. A.	N. A.	N. A.	23.30	82.97	32.35	22.04	UD & UE	N. A.	EP	ED	N. A.
Spain	87.19	62.32	74.76	81.90	87.64	64.68	79.87	84.86	SS	Progression	EHN	EP	0.45
Sri Lanka	74.24	66.46	62.44	57.08	71.01	77.04	60.22	64.45	SS	Progression	EP	SD	-3.22
Sudan	47.44	59.51	53.24	35.46	40.55	75.32	49.57	29.78	UD & UE	Retrogression in both	EP	ED	-6.88
Suriname	75.46	71.36	71.13	59.45	69.48	76.69	65.65	64.66	SS	Retrogression in both	EP	ED	-5.98
Sweden	91.96	65.19	90.76	93.65	90.21	67.02	92.06	92.00	SS	Retrogression in ES	SD	EP	-1.75
Switzerland	91.54	61.71	76.10	91.86	90.73	54.97	80.46	92.46	SS	Retrogression in MIS	ED	EP	-0.80
Syrian Arab Republic	63.64	59.99	58.17	48.32	63.29	71.50	46.88	40.10	UD & UE	Retrogression in both	EP	ED	-0.36
Tajikistan	70.81	72.44	74.21	41.98	73.76	82.98	70.13	52.87	UE	Retrogression in ES	EP	ED	2.95
Tanzania	43.79	66.77	59.76	33.49	41.52	81.75	54.47	50.80	UD & UE	Retrogression in ES	EP	EHN	-2.27
Thailand	80.65	62.88	65.45	66.70	79.59	74.70	70.44	70.76	SS	Progression	EHN	SD	-1.06
Togo	44.32	66.45	55.30	29.71	39.81	84.29	50.22	39.21	UD & UE	Retrogression in ES	EP	ED	-4.51
Trinidad and Tobago	77.05	54.31	77.44	64.50	79.48	50.04	68.22	60.38	UE	Retrogression in both	EHN	EP	2.42
Tunisia	78.09	69.03	68.61	52.54	76.80	77.38	69.84	57.28	SS	Progression	EP	ED	-1.29
Turkey	83.18	61.67	61.19	65.25	82.38	68.01	63.83	64.45	SS	Retrogression in ES	EHN	SD	-0.80
Turkmenistan	67.33	65.05	51.94	38.71	58.41	64.95	65.80	52.68	UD & UE	Progression	SD	ED	-8.92
Uganda	45.92	78.72	55.49	34.17	39.88	83.77	51.12	44.82	UD & UE	Retrogression in MIS	EP	EHN	-6.05
Ukraine	81.95	67.51	74.84	60.82	81.98	72.50	79.52	62.06	SS	Progression	EHN	ED	0.03
United Arab Emirates	76.58	42.50	81.54	59.01	80.07	53.88	73.37	70.07	SS	Retrogression in ES	EHN	EP	3.48
United Kingdom	89.21	60.94	75.86	87.20	89.17	65.24	77.81	87.88	SS	Progression	EHN	EP	-0.04
United States	88.62	45.70	67.36	89.39	86.97	57.87	72.54	87.71	SS	Progression	ED	EP	-1.65
Uruguay	87.47	55.03	69.33	67.90	85.10	67.08	72.60	70.17	SS	Progression	EHN	EP	-2.37
Uzbekistan	74.51	73.70	81.38	46.47	72.96	81.74	70.47	55.33	UE	Retrogression in MIS	EP	ED	-1.56
Vanuatu	N. A.	N. A.	N. A.	N. A.	49.83	70.86	60.02	65.39	UD & UE	N. A.	EP	EHN	N. A.
Venezuela, RB	77.12	65.94	57.22	61.22	58.40	75.20	53.01	50.22	UD & UE	Retrogression in both	EP	ED	-18.72
Vietnam	79.76	60.75	72.04	50.72	79.00	70.44	73.09	65.41	SS	Progression	EHN	ED	-0.77
Yemen, Rep.	40.95	66.52	54.86	33.84	46.18	78.57	41.25	33.71	UD & UE	Progression	EP	ED	5.23
Zambia	43.80	75.08	53.77	34.71	43.40	86.84	46.48	43.24	UD & UE	Retrogression in ES	EP	ED	-0.40
Zimbabwe	47.49	73.89	60.26	45.78	46.90	89.78	60.80	46.00	UD & UE	Retrogression in ES	EP	ED	-0.60

Table S7. Major existing literatures related to SDGs assessment at multiple scales

References	Scales	Major contributions	Innovations of our study
Schmidt-Traub et al., 2017[9]	global	Build up the indicator system and assessment methods for global SDGs assessment (the mean SDG index score, MIS).	Our study develops a new dimension for global SDGs assessment – the SDGs progress evenness, which expands the implications of sustainable development assessment and could reduce the potential overestimation of SDGs performance when large progress differences exists.
Sachs et al., 2016-2022[1-5,16,17]	global	Present the average SDGs performance of each country.	Hidden challenges are revealed by integrating SDGs progress evenness. For instance, the progress of many high-income countries becomes stagnant, despite they generally show the best average performance toward SDGs.
Xu et al., 2020[6]	provincial (China)	First national SDGs assessment over time and space.	Not related to our study.
Fu et al., 2019 and 2020[14,18]	N.A.[*]	Clarify the logic and categorization of the SDGs to facilitate management.	Based on these categories, our study proposes a framework for the priority selection by integrating the SDGs progress evenness, which could help with future SDGs policymaking.
Liu et al., 2021[7]	provincial (China)	First adopt SDGs progress evenness in national SDGs assessment, with hidden challenges disclosed for China.	Our study improves the methodology and first applies it in global SDGs assessment.
Zhu et al., 2023[19]	municipal (Inner Mongolia, China)	Reveal the challenges for the achievement of SDGs in drylands.	Hidden challenges and future opportunities are identified for different categories of countries. For instances, low-, middle-, and high-income countries; slightly, moderately, severely, and extremely arid countries.

REFERENCES

1. Sachs, J., Schmidt-Traub, G., Kroll, C., et al. (2017). SDG Index and Dashboards Report 2017. New York: Bertelsmann Stiftung and Sustainable Development Solutions Network (SDSN).
2. Sachs, J., Schmidt-Traub, G., Kroll, C., et al. (2019). Sustainable Development Report 2019. New York: Bertelsmann Stiftung and Sustainable Development Solutions Network (SDSN).
3. Sachs, J., Schmidt-Traub, G., Kroll, C., et al. (2020). The Sustainable Development Goals and COVID-19. Sustainable Development Report 2020. Cambridge: Cambridge University Press.
4. Sachs, J., Kroll, C., Lafortune, G., et al. (2021). Sustainable Development Report 2021. Cambridge: Cambridge University Press.
5. Sachs, J., Schmidt-Traub, G., Kroll, C., et al. (2018). SDG Index and Dashboards Report 2018. New York: Bertelsmann Stiftung and Sustainable Development Solutions Network (SDSN).
6. Xu, Z., Chau, S.N., Chen, X., et al. (2020). Assessing progress towards sustainable development over space and time. Nature ***577***, 74-78. DOI: 10.1038/s41586-019-1846-3.
7. Liu, Y., Du, J., Wang, Y., et al. (2021). Evenness is important in assessing progress towards sustainable development goals. Natl. Sci. Rev. ***8***, nwaa238. DOI: 10.1093/nsr/nwaa238.
8. Liu, Y., Du, J., Xu, X., et al. (2020). Microtopography-induced ecohydrological effects alter plant community structure. Geoderma ***362***, 114119. DOI: 10.1016/j.geoderma.2019.114119.
9. Schmidt-Traub, G., Kroll, C., Teksoz, K., et al. (2017). National baselines for the Sustainable Development Goals assessed in the SDG Index and Dashboards. Nat. Geosci. ***10***, 547-555. DOI: 10.1038/ngeo2985.
10. Fu, B., Stafford-Smith, M., Wang, Y., et al. (2021). The Global-DEP conceptual framework - research on dryland ecosystems to promote sustainability. Curr. Opin. Env. Sust. ***48***, 17-28. DOI: 10.1016/j.cosust.2020.08.009.
11. Yao, J., Liu, H., Huang, J., et al. (2020). Accelerated dryland expansion regulates future variability in dryland gross primary production. Nat. Commun. ***11***, 1665. DOI: 10.1038/s41467-020-15515-2.
12. Huang, J.P., Yu, H.P., Guan, X.D., et al. (2016). Accelerated dryland expansion under climate change. Nat. Clim. Change ***6***, 166-171. DOI: 10.1038/nclimate2837.
13. Pravalie, R. (2016). Drylands extent and environmental issues. A global approach. Earth-Sci. Rev. ***161***, 259-278. DOI: 10.1016/j.earscirev.2016.08.003.
14. Fu, B., Wang, S., Zhang, J., et al. (2019). Unravelling the complexity in achieving the 17 sustainable-development goals. Natl. Sci. Rev. ***6***, 386-388. DOI: 10.1093/nsr/nwz038.
15. Pradhan, P., Costa, L., Rybski, D., et al. (2017). A Systematic Study of Sustainable Development Goal (SDG) Interactions. Earth's Future ***5***, 1169-1179. DOI: 10.1002/2017EF000632.
16. Sachs, J., Schmidt-Traub, G., Kroll, C., et al. (2016). An SDG Index and Dashboards - Global Report. New York: Bertelsmann Stiftung and Sustainable Development Solutions Network (SDSN).
17. Sachs, J., Lafortune, G., Kroll, C., et al. (2022). From Crisis to Sustainable Development: the SDGs as Roadmap to 2030 and Beyond. Sustainable Development Report 2022. Cambridge: Cambridge University Press.
18. Fu, B., Zhang, J., Wang, S., and Zhao, W. (2020). Classification–coordination–collaboration: a systems approach for advancing Sustainable Development Goals. Natl. Sci. Rev. ***7***, 838-840. DOI: 10.1093/nsr/nwaa048.
19. Zhu, J., Yang, Y., Liu, Y., et al. (2023). Progress and water stress of sustainable development in Chinese northern drylands. J. Clean. Prod. ***399***, 136611. DOI: 10.1016/j.jclepro.2023.136611.
