# Supplementary material for: Overlooked uneven progress across sustainable development goals at the global scale: Challenges and opportunities
Source: Innovation (Camb). 2024 Jan 8;5(2):100573. doi: 10.1016/j.xinn.2024.100573 (PMC10876912; doi:10.1016/j.xinn.2024.100573)
Supplement: Document S2. Article plus supplemental information [file mmc3.pdf]

Overlooked uneven progress across sustainable development goals at the global scale: Challenges and opportunities

Yali Liu,[1] Jianqing Du,[2,3] Yanfen Wang,[2,3,4,*] Xiaoyong Cui,[2,5] Jichang Dong,[6] Pan Gu,[7] Yanbin Hao,[2,5] Kai Xue,[2,3,8] Hongbo Duan,[6] Anquan Xia,[5] Yi Hu,[6] Zhi Dong,[9] Bingfang Wu,[3,10] Jürgen P. Kropp,[11,12] and Bojie Fu[3,13]

*Correspondence: yfwang@ucas.ac.cn

Received: June 1, 2023; Accepted: January 2, 2024; Published Online: January 8, 2024; https://doi.org/10.1016/j.xinn.2024.100573

© 2024 The Author(s). This is an open access article under the CC BY license (http://creativecommons.org/licenses/by/4.0/).

GRAPHICAL ABSTRACT

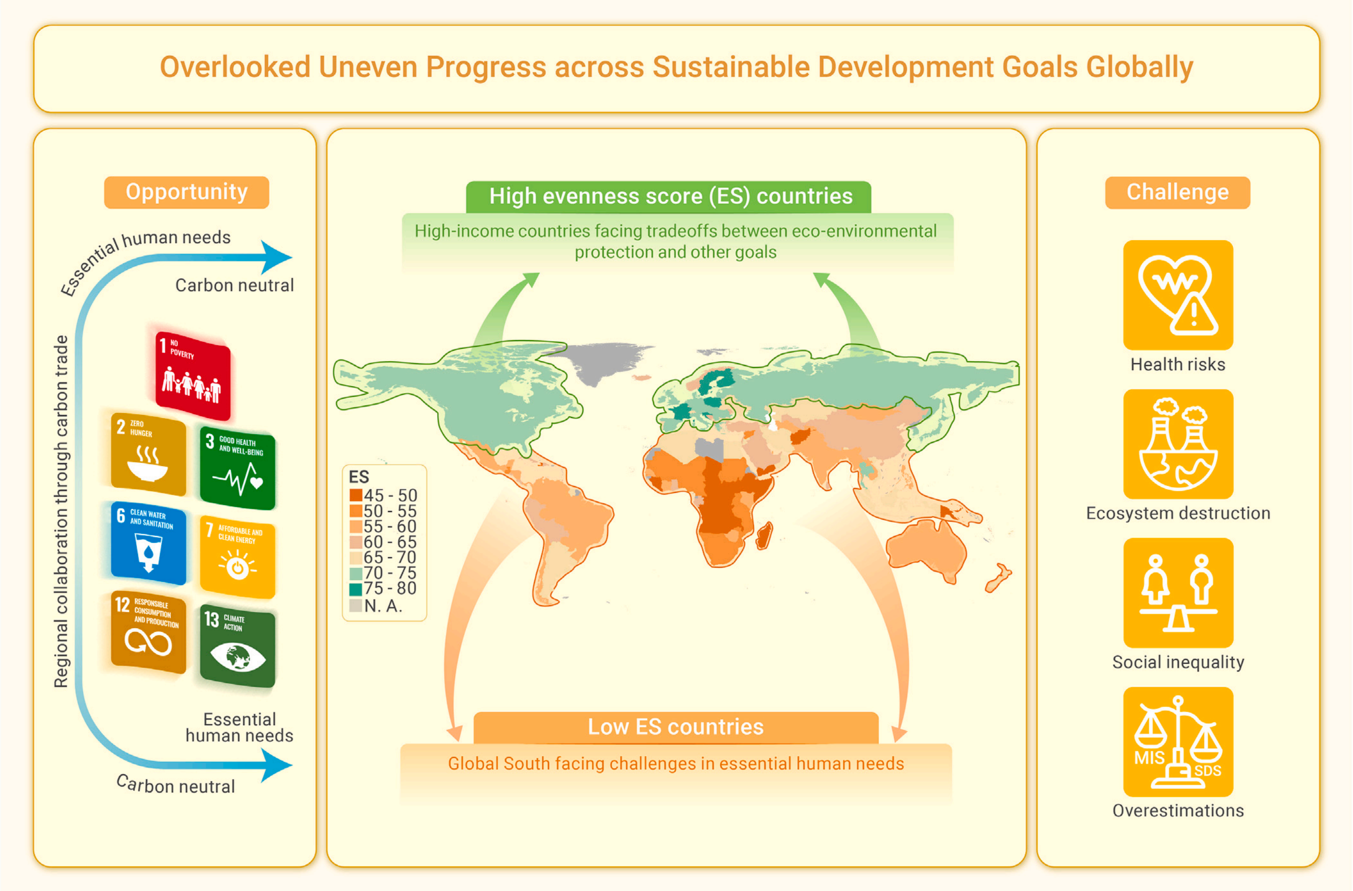

PUBLIC SUMMARY

- Integrating progress evenness reveals hidden challenges and opportunities toward sustainable development goals (SDGs).
- Uneven SDGs progress is related to health risks, ecosystem damage, and social inequality.
- Uneven SDGs progress is common in the low-income and extremely arid countries.
- Many high-income countries face antagonism between climate actions and other goals.
- Complementary collaborations are crucial to achieve SDGs.

ARTICLE

Overlooked uneven progress across sustainable development goals at the global scale: Challenges and opportunities

Yali Liu,[1] Jianqing Du,[2,3] Yanfen Wang,[2,3,4,*] Xiaoyong Cui,[2,5] Jichang Dong,[6] Pan Gu,[7] Yanbin Hao,[2,5] Kai Xue,[2,3,8] Hongbo Duan,[6] Anquan Xia,[5] Yi Hu,[6] Zhi Dong,[9] Bingfang Wu,[3,10] Jürgen P. Kropp,[11,12] and Bojie Fu[3,13]

[1]School of Grassland Science, Beijing Forestry University, Beijing 100083, China
[2]Beijing Yanshan Earth Critical Zone National Research Station, University of Chinese Academy of Sciences, Beijing 101408, China
[3]College of Resources and Environment, University of Chinese Academy of Sciences, Beijing 100049, China
[4]State Key Laboratory of Tibetan Plateau Earth System, Environment and Resources, Chinese Academy of Sciences, Beijing 100101, China
[5]College of Life Sciences, University of Chinese Academy of Sciences, Beijing 100049, China
[6]School of Economics and Management, University of Chinese Academy of Sciences, Beijing 100190, China
[7]School of Humanities, University of Chinese Academy of Sciences, Beijing 100049, China
[8]Key Laboratory of Adaptation and Evolution of Plateau Biota, Northwest Institute of Plateau Biology, Chinese Academy of Sciences, Xining 810001, China
[9]School of Innovation and Entrepreneurship, University of Chinese Academy of Sciences, Beijing 100190, China
[10]State Key Laboratory of Remote Sensing Science, Aerospace Information Research Institute, Chinese Academy of Sciences, Beijing 100101, China
[11]Potsdam Institute for Climate Impact Research, 14412 Potsdam, Germany
[12]Institute for Environmental Science and Geography, University of Potsdam, 14412 Potsdam, Germany
[13]State Key Laboratory of Urban and Regional Ecology, Research Center for Eco-Environmental Sciences, Chinese Academy of Sciences, Beijing 100085, China
*Correspondence: yfwang@ucas.ac.cn

Received: June 1, 2023; Accepted: January 2, 2024; Published Online: January 8, 2024; https://doi.org/10.1016/j.xinn.2024.100573

© 2024 The Author(s). This is an open access article under the CC BY license (http://creativecommons.org/licenses/by/4.0/).

Citation: Liu Y., Du J., Wang Y., et al., (2024). Overlooked uneven progress across sustainable development goals at the global scale: Challenges and opportunities. The Innovation **5(2)**, 100573.

ABSTRACT

Differences in progress across sustainable development goals (SDGs) are widespread globally; meanwhile, the rising call for prioritizing specific SDGs may exacerbate such gaps. Nevertheless, how these progress differences would influence global sustainable development has been long neglected. Here, we present the first quantitative assessment of SDGs' progress differences globally by adopting the SDGs progress evenness index. Our results highlight that the uneven progress across SDGs has been a hindrance to sustainable development because (1) it is strongly associated with many public health risks (e.g., air pollution), social inequalities (e.g., gender inequality, modern slavery, wealth gap), and a reduction in life expectancy; (2) it is also associated with deforestation and habitat loss in terrestrial and marine ecosystems, increasing the challenges related to biodiversity conservation; (3) most countries with low average SDGs performance show lower progress evenness, which further hinders their fulfillment of SDGs; and (4) many countries with high average SDGs performance also showcase stagnation or even retrogression in progress evenness, which is partly ascribed to the antagonism between climate actions and other goals. These findings highlight that while setting SDGs priorities may be more realistic under the constraints of multiple global stressors, caution must be exercised to avoid new problems from intensifying uneven progress across goals. Moreover, our study reveals that the urgent needs regarding SDGs of different regions seem complementary, emphasizing that regional collaborations (e.g., demand-oriented carbon trading between SDGs poorly performed and well-performed countries) may promote sustainable development achievements at the global scale.

INTRODUCTION

The 17 sustainable development goals (SDGs) were put forward at the 2015 United Nations Summit, and aim to provide a shared blueprint for peace and prosperity for all people and our planet.[1] As we move toward 2030, multiple global stressors such as the coronavirus disease 2019 (COVID-19) pandemic and the Russo-Ukrainian conflict cast a shadow over the holistic achievements proposed by the SDGs.[2,3] These multiple and simultaneous crises have directly threatened the SDGs regarding the goals no poverty, zero hunger, good health and well-being, and affordable and clean energy, and indirectly through its hindering of globalization.[2–4] Moreover, they have diverted policy and public attention away from long-term planning and investment,[3] leading to the rising call for a focus on more immediate and clearer SDG priorities instead of on the holistic achievement of the SDGs.[2,5]

These global stressors have also exposed, if not amplified, the conflicts over targets.[2] Given that potential priorities such as food security, biodiversity conservation, and climate actions widely interact with other goals,[6–10] the overemphasis on one or a few priorities could impede the achievement of the others. For instance, increasing food production could intensify emerging human infectious diseases globally.[6] In addition, although the reduction in human activities during the COVID-19 pandemic could benefit environment-related targets,[4,11–14] the strict regulations that emerged, such as travel restrictions, have largely slowed down globalization[15] and may expose the global economy and food security to further threats.[2] The Russo-Ukrainian conflict has also plunged Europe into a deep energy crisis, likely intensifying the tradeoffs between sustainable energy consumption and other SDGs.[3] In general, these progress differences across SDGs, which already exist because of the socioeconomic conditions and/or the interactions among SDGs,[16–21] may worsen because of these external disturbances.

It remains, nonetheless, that we currently lack a comprehensive understanding of the progress differences across SDGs at the global scale, whether and how they may influence SDGs achievement, and whether they may affect priority decision making in this context. The most recent assessment systems use the arithmetic mean of all 17 SDGs to quantify a given country's or region's SDG performance[13,22,23] without considering the differences in performance across goals. An example of such methods is the widely used SDG index score proposed by the SDG Index and Dashboards 2016.[23] Given their limitations, these methods could yield overoptimistic results; for example, they may yield a high average SDG performance because of a few well-achieved goals that have tradeoffs with others—such as a rapid economic development at the cost of environmental protection.[24] Moreover, considering only this average score is very likely to lead to the neglect of the challenges caused by an uneven progress across goals. For instance, Liu et al. suggested that many top-income regions in China, whose performance toward SDGs was previously thought to be relatively good, were reaching a bottleneck because of their uneven development pathway.[24] Such uneven progress could lead to challenges such as water depletion and air pollution.[25–28] As such, it limits the policy-making capacity toward SDGs, particularly in choosing priorities without bringing up new problems.

Here, we innovatively adopted the concept of "progress evenness" from biodiversity measurements in ecology[29] to quantify the global pattern of progress differences toward SDGs by country from 2017 to 2021 (hereafter referred to as SDGs progress evenness), and then investigated the associated challenges (explained further in the materials and methods section). A low SDGs progress evenness reflects a significant progress difference—that is, an uneven progress across SDGs.[24] This score is supposed to supplement the existing major SDGs assessment indicator (i.e., the mean SDG index score [MIS]), which may then be combined to form a novel two-dimensional assessment system by integrating the average performance across all 17 SDGs and their progress differences (explained further in the materials and methods section).

Table 1. Effects of GDP per capita and ES on the indicators

	Indicators related to health											
	Neonatal mortality rate, per 1,000 live births $R2 = 0.67$, $F_{2,155} = 161.23$			Mortality rate under 5, per 1,000 live births $R2 = 0.63$, $F_{2,155} = 135.75$			Age-standardized death rate owing to air pollution, per 100,000 population $R2 = 0.60$, $F_{2,155} = 118.88$			Life expectancy at birth, years $R2 = 0.70$, $F_{2,155} = 186.30$		
Factor	β	t	p	β	t	p	β	t	p	β	t	p
GDP per capita	−0.23	−4.38	<0.001	−0.15	−2.69	0.008	−0.29	−4.99	<0.001	0.35	7.01	<0.001
ES	−0.69	−13.24	<0.001	−0.72	−13.03	<0.001	−0.60	−10.47	<0.001	0.62	12.50	<0.001

	Indicators related to the environment											
	Annual mean concentration of PM 2.5, μg/m^3 $R^2 = 0.17$, $F_{2,155} = 16.77$			Mean area that is protected in marine sites important to biodiversity, % $R^2 = 0.16$, $F_{2,109} = 11.68$			Mean area that is protected in terrestrial sites important to biodiversity, % $R^2 = 0.13$, $F_{2,153} = 12.23$			Permanent deforestation, % of forest area, 5-year average $R^2 = 0.11$, $F_{2,141} = 9.80$		
Factor	β	t	P	β	t	p	β	t	p	β	t	p
GDP per capita	−0.20	−2.43	0.02	0.13	1.25	0.20	0.21	2.50	0.01	−0.12	−1.26	0.18
ES	−0.29	−3.47	0.001	0.34	3.39	0.001	0.22	2.56	0.01	−0.27	−3.01	0.003

	Indicators related to equality											
	Ratio of female-to-male mean year of education received, % $R2 = 0.25$, $F_{2,151} = 26.74$			Seats held by women in national parliament, % $R^2 = 0.06$, $F_{2,155} = 6.31$			Victims of modern slavery, per 1,000 population $R2 = 0.22$, $F_{2,137} = 20.96$			Gini coefficient adjusted for top income $R^2 = 0.27$, $F_{2,140} = 27.36$		
Factor	β	t	p	β	t	P	β	t	p	β	t	p
GDP per capita	0.13	1.64	0.10	0.22	2.5	0.01	−0.18	−2.07	0.04	−0.29	−3.45	0.001
ES	0.44	5.52	<0.001	0.09	1.06	0.30	−0.37	−4.37	<0.001	−0.33	−3.95	<0.001

All models are significant at $p < 0.05$. β, standardized coefficient; $PM_{2.5}$, particulate matter of <2.5 μm in diameter.

Our results highlight that an uneven progress, rather than poor economic performance, is associated more with problems such as health risks, ecosystem destruction, and social inequality. Low-income countries and drylands exhibited the lowest SDGs progress evenness, which further broadens their gaps in approaching the SDGs. Meanwhile, many high-income countries (HICs) had setbacks in the holistic achievement of SDGs because of the stagnation or even retrogression in SDGs progress evenness. While considering SDGs progress evenness, we estimated that only approximately 30% of countries would be able to achieve SDGs by 2030—an underwhelming achievement that is far below the projections made based on previous SDGs assessment methods. These findings underscore that future policy making should consider SDGs progress evenness, especially in choosing priorities. Nevertheless, the uneven progress poses not only global challenges but also opportunities for international cooperation to restore and accelerate SDGs progress toward 2030 and beyond.

RESULTS

Methods summary

In general, the MIS, the SDGs progress evenness score (ES), and their integration (i.e., the geometric mean of MIS and ES, hereafter referred to as sustainable development score [SDS]) all were considered to present a comprehensive understanding of global sustainable development. A radar chart method[24] was adopted to quantify ES in each country, and we used a binary regression to study how ES relates to human health, social equality, and the environment, in addition to the economic condition as the major influencing factor. Then, to tap into the hidden challenges left behind by past assessments using exclusively the MIS, we reassessed the spatial and temporal progress toward SDGs by examining the ES global pattern from 2017 to 2021 and integrated it with the widely adopted MIS.[4,13,30–32] This enabled us to investigate whether and how uneven progress across goals has impeded the holistic achievement of SDGs globally. Moreover, in response to the rising calls for reprioritizing SDGs under global stressors, we conducted analyses to explore the SDGs that require urgent prioritization by integrating each country's poorly performed SDGs and development pathway.

Social and environmental issues associated with uneven progress across SDGs

By controlling for economic development level across countries using gross domestic product (GDP) per capita, we revealed that uneven progress across SDGs was related to global challenges such as public health issues, environmental damage, and social inequality. Firstly, ES was positively related to life expectancy, strongly related to a high mortality rate for neonates and young children younger than 5 years, and elevated the age-standardized death rate owing to air pollution (Table 1). The binary regression further showed that these public health issues were related more to ES than to GDP per capita, because the former had much higher standardized coefficients (β) than the latter (from approximately 1.8 to 4.8 times; Table 1). To summarize, ES and GDP per capita could explain 70% of the variance in life expectancy. Some of these public health issues may be ascribed partly to environmental pollution and social inequality, which were also related to an uneven progress across SDGs. For instance, we observed an increase in the annual mean concentration of particulate matter of less than 2.5 μm in diameter ($PM_{2.5}$), with a decrease in ES (Table 1). Further analyses suggested that an elevated $PM_{2.5}$ could increase the aforementioned mortality rates and reduce life expectancy (Figure S1; Table S1).

In the path analysis, ES still exhibited a strong direct influence on all four indicators related to public health (Figure S1), indirectly affecting public health through $PM_{2.5}$ and social inequality factors (i.e., ratio of female-to-male of education received, modern slavery, and the Gini coefficient). Unsurprisingly, although $PM_{2.5}$ was an essential mediator affecting the age-standardized death rate owing to air pollution (Figure S1C), it did not contribute much to the mortality rate for neonates and young children younger than 5 years (Figures S1A and S1B) or to life expectancy (Figure S1D). Regarding social inequality factors, an elevated ES was associated with an increase in female rights, which could then strongly decrease the mortality rate for neonates and young children younger than 5 years (Figures S1E and S1F) while increasing life expectancy (Figure S1H).

ES was also crucial for biodiversity conservation, because a high ES was related to a larger protected area important for terrestrial and marine biodiversity, whereas a low ES was strongly related to deforestation (Table 1). Similar to the findings for public health issues, ES had a greater influence on biodiversity conservation indicators than did GDP per capita (Table 1). Moreover, both low ES and GDP per capita were related to social inequality (e.g., gender inequality, modern slavery, the rich–poor gap; Table 1). ES was also much more influential on female education level and modern slavery than GDP per capita (β = 0.44 versus 0.13, respectively, for female education level; β = −0.37 versus −0.18, respectively, for modern slavery).

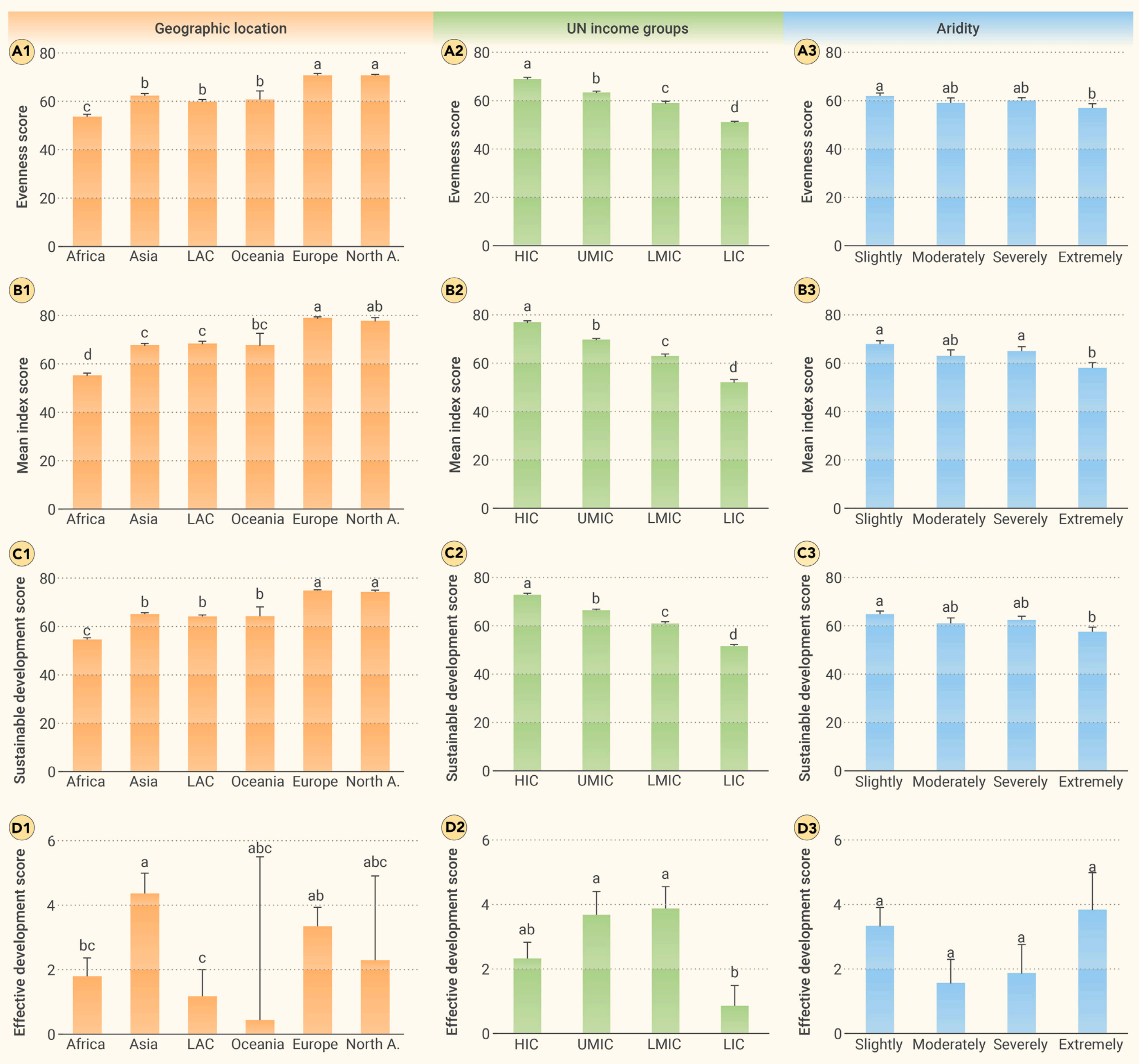

Figure 1. **Differences in the ES (A1–A3), MIS (B1–B3), SDS (C1–C3), and EDS (D1–D3) across geographic locations, United Nations income groups, and arid levels in 2021** The histogram with error bars presents the mean value ±SE. Different lowercase letters visualize the significant differences at $p < 0.05$. EDS is based on data from 2017 to 2021. HIC, high-income countries; LAC, Latin America and the Caribbean; LIC, low-income countries; LMIC, lower-middle-income countries; North A., North America; UMIC, upper-middle-income countries.

Spatial pattern of global sustainable development when incorporating SDGs progress evenness

While considering SDGs progress evenness (explained further in the materials and methods section), we reassessed the progress toward SDGs in 169 countries. Our results highlight that regions with a low MIS also had a low ES, whereas high-MIS regions had a high ES (Figures 1A and 1B); for instance, in 2021, HIC (ie, most countries in Europe and North America; Figure S2; Table S2) had the highest MIS and ES (76.70 for MIS, 68.74 for ES), whereas low-income countries (LICs; in general, in Africa; Figure S2) had the lowest MIS and ES (51.88 for MIS, 50.83 for ES; Table S3). In addition, slightly arid countries also had higher MIS and ES than extremely arid countries (67.89 versus 58.11, respectively, for MIS; 61.95 versus 56.97, respectively, for ES; Table S3). Consequently, countries facing the greatest challenges were LICs in drylands, namely Chad, Somalia, Niger, Yemen, Mali, Burkina Faso, and Gambia. These countries had a lower SDS (see the materials and methods section for the definition of SDS) than nonextremely arid LICs (49.49 versus 52.06, respectively).

The progress toward SDGs has varied mainly by regions of the globe, regardless of the index under consideration (ie, MIS, ES, and SDS; Figures 1A–1C and 2). Based on the SDS in 2021, Sub-Saharan Africa, the Caribbean, and West and South Asia were considered poorly performing regions, whereas high-income regions such as North America and Europe generally were considered well-performing regions (Figure 2C; Table S2). In general, the deficiencies in the poorly performing regions were related to SDGs 1 (no poverty), 2 (zero hunger), and 9 (industry, innovation and infrastructure), whereas the deficiencies in the well-performing regions were related to SDGs 12 (responsible consumption and production), 14 (life below water), and 17 (partnerships; Figure S3).

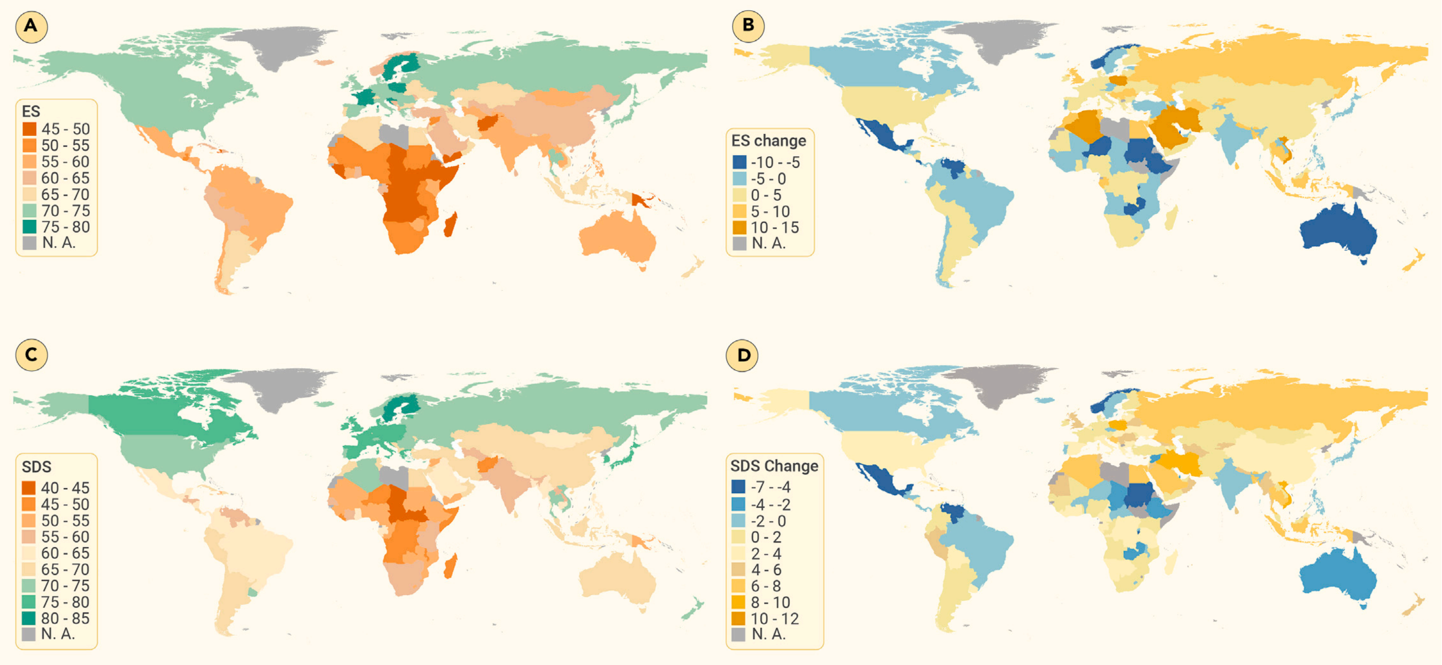

Figure 2. The spatial pattern of the ES and the sustainable development score (SDS) in 2021 and corresponding changes from 2017 to 2021 (A) ES; (B) change in ES; (C) SDS; (D) change in SDS. ES and SDS increase from red to green; notably, the change in ES and SDS increases from blue (negative) to orange (positive). N.A., not available.

Progress toward SDGs over time

The development pathway toward SDGs and the effective development score (EDS; i.e., the vector defined by a given country's pairwise MIS and ES between 2017 and 2021) were used to evaluate the progress toward SDGs from 2017 to 2021 (explained further in the materials and methods section). The findings show that well-performing countries (mostly HICs) had their own challenges, because they generally made little or even negative SDS progress from 2017 to 2021. The number of countries experiencing a retrogression increased from 10 to 22 (44.9%) when considering ES (Figure 3A; Table S2), because some countries had setbacks only in ES. For example, the MIS of the Czech Republic increased from 63.10 to 65.06 from 2017 to 2021, whereas the ES decreased from 63.59 to 57.63; this can be ascribed largely to solid progress in SDG 9 but a setback in SDG 13 (climate action). As a result, the EDS of HIC was lower than that of middle-income countries (Figure 1D2). Nevertheless, the most undesirable pathway was still found in LICs, generally showing an uneven development pathway toward SDGs (decrease in ES; Figure 3D).

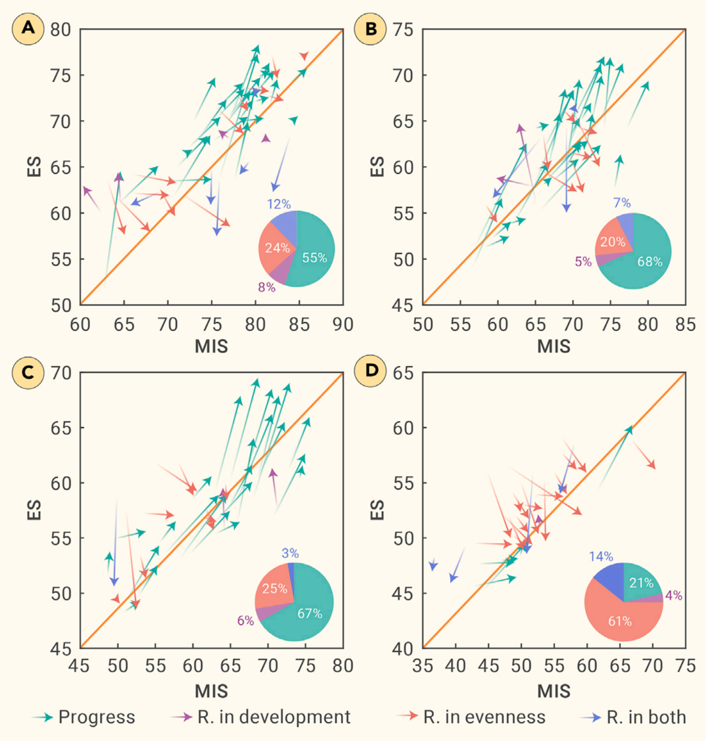

Figure 3. Development pathways for different United Nations income groups from 2017 to 2021 (A) HICs; (B) UMICs; (C) LMICs; (D) LICs. Different colors are used to represent the 4 types of development pathways: progress (green; progressing pathway), R. in development (retrogression in development, purple; underdeveloped pathway), R. in evenness (retrogression in evenness, red; uneven development pathway), and R. in both (retrogression in both, blue; retrogressive pathway). The orange diagonal stands for the ideal pathway (slope = 1). The pie charts at the bottom right corners show the proportion of different pathways.

In 2020, HICs had the lowest EDS, even less than LICs (1.50 versus 1.51; Figure S4), albeit HICs also showed more resistance to the effects of the COVID-19 pandemic than LICs, because the latter had the lowest EDS in 2021 (decreased to 0.83; Figure 1D2). Moreover, more countries stepped into an uneven development pathway (ie, retrogression in ES plus retrogression in both ES and MIS) in 2021, with the proportion of uneven development pathways increasing from 19% to 27%, 25% to 28%, and 71% to 75% for upper-middle-income countries, lower-middle-income countries, and LICs, respectively (Figures 3 and S5). The only exception was found in HICs, which rarely showed changes in the proportion of uneven development pathways (39% in 2020 versus 36% in 2021).

The relationships among SDGs were also analyzed across income and aridity levels to study the potential causes of an uneven development pathway. Our results highlighted that the significant interactions among SDGs tended to be weaker in LICs (versus HICs) and extremely arid countries (versus slightly arid; Figures S6A and S6B). For example, a strong negative relationship between SDG 12 (responsible consumption and production) and other goals only existed in HICs (Figure S6A). The goals that generally correlated negatively with most other goals were SDGs 12 and 13 (Figure S6; Table S4). Compared with the pre-pandemic situation in 2020, the tradeoffs intensified in lower-middle-income countries and moderately arid countries, especially those between SDG 14 and other SDGs (Figures S6 and S7).

Projection of SDG performance across countries in 2030

We projected the MIS and SDS in 2030 using curvilinear regression and a gray forecast model based on data from 2017 to 2021. Upon considering ES in the analyses, only 26.7% (by curvilinear regression) and 34.1% (by gray forecast model) of countries would be able to approach the SDGs (i.e., projected score higher than 80 based on data from 2017 to 2021) in 2030, which is much less

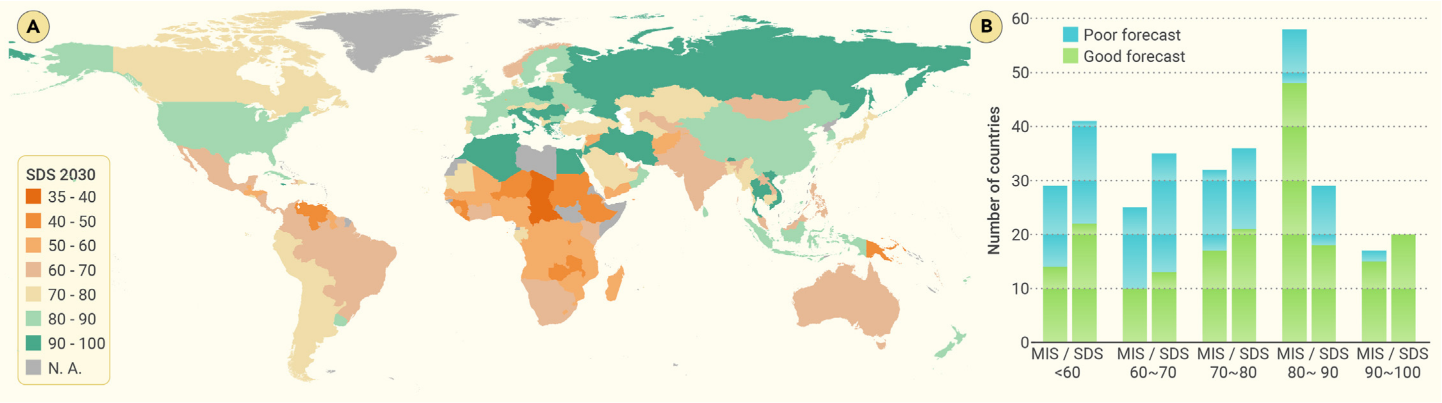

Figure 4. Projections of SDG performance across countries in 2030 based on trends from 2017 to 2021 (A) Global variation of the SDS; (B) the number of countries with different scores projected by the MIS and SDS. SDS increases from red (poorly performing) to green (well performing).

than the projection using only MIS (41% and 47.2% based on curvilinear regression and gray forecast model, respectively; Figure 4B; Table S5). In addition, the number of countries with a projected SDS lower than 60 was much higher when considering ES than when using only MIS (27–28 using MIS versus 40–41 using SDS; Figure 4B).

These results align with the challenges mentioned above for the relatively well-performing and poorly performing countries. In addition, the situation could be even worse, because most countries with SDS projections above 80 in 2030 were middle-income countries (56% and 49% in curvilinear regression and gray forecast model, respectively; Figure 4A; Table S5). Furthermore, from 2017 to 2021, middle-income countries exhibited the most desirable development pathway toward SDGs (Figures 3B and 3C).

Urgency and potential for priority development

In response to the rising calls to reprioritize SDGs under global stressors, we integrated each country's poorly performing SDGs and development pathway toward the SDGs to identify goals that should be urgently prioritized (explained further in the materials and methods section).

In 2021, most countries were considered to have either a relatively sustainable performance (relatively high MIS and ES) or an underdeveloped and uneven performance (relatively low MIS and ES). Meanwhile, approximately 51.5% of countries (85 countries) showed an uneven performance across SDGs (relatively low ES; Table S6). There were 42 African countries (all LICs; out of 48 African countries) and 13 extremely arid countries (out of 20 extremely arid countries) that were classified as having an uneven performance, and only 36 countries with an uneven performance did not belong to any of these 3 categories (Figure S8). Fifty-one countries were classified as poorly performing on essential human needs and considered to have uneven performance (Table S6)—41 were in Africa, 46 were LICs or lower-middle-income countries, and 21 were extremely or severely arid countries.

As for the development pathway toward the SDGs from 2017 to 2021, 28 countries with an uneven performance (out of 51) also showed an uneven development pathway (decrease in ES), and the other 17 showed an even pathway (increase in ES; data not available for 6 countries; Figure 5). Among the 16 countries with a progressing pathway (increase in both MIS and ES), 4 countries showed improved performance in essential human needs; the other 12 (10 from Africa and 2 from Asia) showed an average decline of 4.97 points in essential human needs and a general considerable progress in eco-environmental protection and economic development (average increase of 13.14 and 9.64, respectively). Therefore, these 12 countries may need to focus more on supporting essential human needs in the future. Five countries (out of the 28 countries with an uneven development pathway) with an uneven development pathway showed an increasing average score for essential human needs, albeit not as high as the increase in eco-environmental protection and economic development (Table S6). The other 23 countries (out of the 28 countries with an uneven development pathway) generally showed the worst performance in essential human needs (11/23) and had an average decrease of 4.37 points from 2017 to 2021 (Figure 5; Table S6); they were thus considered to be the countries that need to most urgently prioritize the development of essential human needs. These countries also had a distinct geographic feature, because most were in Africa (except Bolivarian Republic of Venezuela, India, and Lao People's Democratic Republic [Laos]), suggesting that countries in Africa, particularly LICs and lower-middle-income African countries, need to reprioritize their development pathways regarding SDGs.

Meanwhile, 49 countries, generally HICs (43/49 countries; 24 in Europe, 12 in Asia, and 0 in Africa) with good performance on essential human needs and economic development (Table S6), had poor performance regarding eco-environmental protection. The challenge for these countries may be striking a balance between human development and eco-environmental protection. Furthermore, the number of countries with a relatively sustainable performance decreased by 10 from 2020 to 2021 (87 in 2020 versus 77 in 2021), and the proportion of countries with poor performance in eco-environmental protection among countries with a relatively sustainable performance increased from 37.93% to 51.95% during the same period (Figure S9).

DISCUSSION

Major challenges associated with an uneven progress across SDGs

This study exposes various global issues related to public health, social inequality, and the environment by considering SDGs progress evenness. Our results suggest that SDGs progress evenness has a greater influence (versus GDP per capita) on public health issues (e.g., the mortality rate of neonates and young children younger than 5 years and the age-standardized death rate owing to air pollution) and a stronger association with life expectancy. These findings regarding SDGs progress evenness are partly ascribed to the environmental pollution (e.g., $PM_{2.5}$, ozone, and nitrogen pollution) associated with an uneven progress across SDGs. For instance, elevated $PM_{2.5}$ concentration—which was shown to be associated with an uneven progress across SDGs in this study—is a crucial risk factor for pulmonary diseases, cancers, diabetes, and cardiovascular health,[33–35] and could increase the premature mortality rate.[12] These findings of past studies are generally concordant with our results. Moreover, our results indicate that the uneven progress across SDGs could result in social inequalities such as gender inequality, which further reduce life expectancy.[36,37] This study also demonstrates that that biodiversity conservation becomes more challenging when under the constraints of an uneven progress across SDGs, which was mainly associated with deforestation and habitat loss in terrestrial and marine ecosystems. Furthermore, the regions that we identified to have the lowest SDGs progress evenness—Sub-Saharan Africa, Latin America and the Caribbean, and South and Southeast Asia—were also identified in a past study to be global priority areas for ecosystem restoration to ensure the efficient promotion of biodiversity conservation.[38] Growing evidence suggests that ecosystem destruction and fragmentation increases the risk of disease transmission to humans[5,39–41]; thus, these aforementioned regions may face greater challenges regarding public health in the near future. To summarize, the global issues associated with an uneven progress across SDGs could impede SDGs achievement, particularly if this uneven status intensifies because of multiple global stressors.

In the present study, low-income and extremely arid countries showed a low SDGs progress evenness and the worst average SDG performance. Given the aforementioned problems associated with an uneven progress across SDGs, we can infer that the situation of these regions is even worse than previously

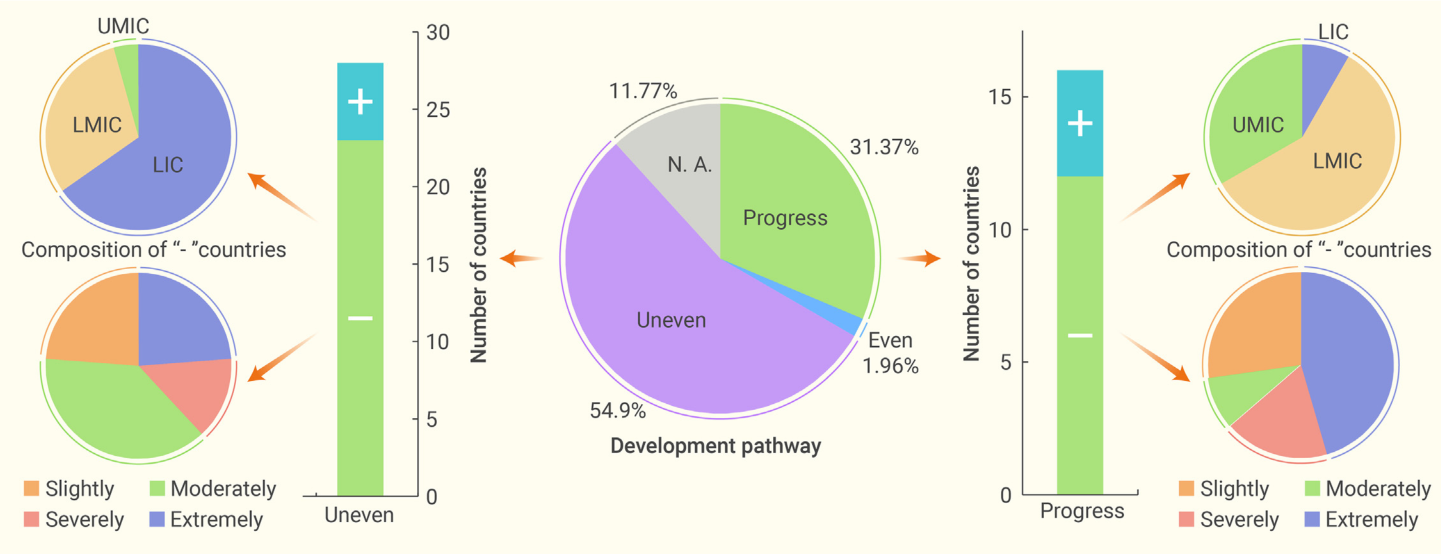

Figure 5. Composition of the 51 countries with a relatively poor performance on essential human needs All 51 countries were classified to have an uneven development pathway toward SDGs. The pie chart in the center represents the ratio for each development pathway. Progress stands for progress in both MIS and ES; Even refers to progress only in ES; Uneven refers to retrogression in ES. "+" and "-" stand for countries with increasing or decreasing scores in essential human needs, respectively. The composition of countries marked with "-" is shown in the small pie chart.

reported.[13] A case study in China recently reported a similar conclusion, demonstrating that the country's north and northwest arid regions are low-income regions with poor SDGs performance.[24] These results imply that extreme droughts could be a crucial factor restricting sustainable development in the future. Such findings are of particular significance under the accelerated expansion of drylands under climate change.[42] At the same time, our findings show that the progress of many HICs reached a bottleneck, because many of these countries (44.9%) showed a retrogression development pathway from 2017 to 2021, although they also generally had the best average performance toward SDGs. Such stagnant progress for economically developed regions was also reported in the aforementioned study in China,[24] and is probably due to the tradeoffs between some SDGs.[16,17,19] In our evidence, the SDGs that negatively correlated with most other SDGs were SDGs 12 and 13, and this is corroborated by previous studies.[16,19,20] Our results also indicate that HICs and countries under economic or environmental stresses face divergent problems; although HICs need to achieve development in a responsible and sustainable way, the latter are struggling to fulfill the essential needs for human survival.[13] Although the dilemmas vary by region, the challenges that seem to generally hinder the holistic achievement of SDGs remain to be securing responsible consumption and production and dealing with the associated climate issues,[43] such as carbon emissions reduction, which is becoming more and more challenging.[44]

Among one of the most alarming and problematic findings of the present study, we observed that the incorporation of SDGs progress evenness into the analysis led to a projection of only approximately 30% of the countries analyzed being able to achieve the SDGs by 2030. This indicates that the ostensibly good average SDGs performance of many countries is primarily related to improvements in only a few of the goals. Moreover, many countries that were observed in this study to have the potential to achieve SDGs by 2030 are middle-income countries; considering their income characteristics, it may be hard for these countries to be able to sustain their noteworthy trends toward SDGs. Some of the reasons for this are as follows: (1) they may be caught in the middle-income trap, as many countries are[45,46]; (2) even if they manage to avoid the middle-income trap (e.g., by ramping domestic demand and/or finding new markets) and become HICs, they are very likely to step into the same bottlenecks as HICs; and (3) their progress could be affected by global stressors such as military clashes, energy crises, and global health risks.[47] Therefore, prioritizing development in essential human needs rather than overoptimistically pursuing a holistic achievement of all SDGs seems more realistic under the multiple current global stressors.[2]

This study also identifies that LICs in Africa are the countries that most urgently need to reprioritize their development pathways toward SDGs, and that they should focus particularly on SDGs 1, 3 (good health and well-being), 7 (affordable and clean energy), and 9. Among these SDGs that may need to be prioritized, SDG 7 exhibited strong associations with the others. A recent study suggested that replacing traditional fuel with clean energy in rural pasture areas could improve the local livelihood by providing electricity, lowering health risks, and reducing indoor air pollution (e.g., from burning coal or cow dung), and lead to improvements regarding performance on SDGs 1 and 3.[48] Moreover, there is an expected decrease in the cost of clean energy for the near future. For instance, solar power is predicted to become the cheapest energy source in most countries in 2027.[49] With a low technical threshold and cost, distributed solar power could effectively accelerate the progress of SDG 7, which should be considered an effective pathway to promote sustainable development in these countries.

Future opportunities enlighten by integrating SDGs progress evenness

Although the uneven progress across SDGs observed in this study brings many challenges for global sustainable development, it also presents opportunities to enhance regional cooperation. Our findings demonstrate that many HICs are facing a stagnant development of SDGs 12 and 13, which may be related to tradeoffs between climate action and other goals. To solve these problems, a sharp acceleration in clean energy innovation is essential, although this may be a very time-consuming endeavor.[50] There is also the possibility of engaging in other natural-based actions such as natural climate solutions[51]; although crucial, these efforts are not sufficient to tackle all of the related problems.[52] In this case, regional collaborations could effectively complement existing actions.[52] Considering that LICs and middle-income countries generally performed well on SDGs 12 (responsible consumption and production) and 13 (climate action) but poorly on SDGs related to essential human needs or economic development, we argue that regional collaborations between LICs, middle-income countries, and HICs related to climate action (e.g., collaborations through carbon trade between Europe and Africa or North America and Latin America) could be mutually beneficial. Notably, LICs should aim at acquiring the infrastructure and technology conducive to development and sustainable livelihoods, rather than simply fulfilling basic needs (e.g., food, water, power).[1,53] For example, LICs could focus on acquiring clean energy technology, as well as agricultural infrastructure and technology. If LICs can successfully cooperate with other countries to acquire such technologies, they may be able to more autonomously improve their performance regarding essential human needs and then sustain these betterments. These actions are particularly important for LICs because their progress toward SDGs has been primarily uneven.

Meanwhile, middle-income countries may need to focus on technical and industrial innovation, because this may enable them to undertake a more effective development pathway without incurring environmental costs, as well as help them step over the middle-income trap. In addition, regional collaborations could also promote the stability of suitable development across countries. For instance, Germany exported medical supplies and received critically ill patients from

neighboring European countries, relieving the burden on the healthcare system of many of these countries and reducing their own pandemic-induced economic losses. On the contrary, the setbacks in SDGs performances in Latin America and the Caribbean may be related to the lack of or reduced regional collaborations caused by COVID-19. Therefore, revitalizing global partnerships is an urgent matter for supporting SDGs performance worldwide, and future research should focus on identifying various solutions for such revitalization.

CONCLUSION

By incorporating SDGs progress evenness into the analyses, this study delivers a detailed evaluation of the development pathways and progress toward SDGs across regions worldwide (see Table S7 for comparisons with other assessments), making this the most comprehensive assessment of global sustainable development to date and to the best of our knowledge. In our findings, an uneven progress across SDGs was associated with problems such as public health risks, ecosystem destruction, and social inequality, which are also identified as major global challenges by the United Nations Environment Programme.[54] These findings point to a possible dilemma related to priority development, because environmental pollution and other problems associated with an uneven progress across SDGs may, in turn, compromise human health and well-being. Nevertheless, the uneven development pathways may sometimes be unavoidable or even efficient for many countries because of their specific resource endowments or social backgrounds. Therefore, to guide future policy making toward SDGs, future studies should focus on exploring the tipping points, mechanisms, and pathways linking uneven progress across SDGs with economic development, environmental pollution, public health, and social equality. Overall, we argue that SDGs progress evenness represents a novel and far-reaching equality issue in human development. Its relationship with the widely used mean SDG index score seems to be similar to that between average income and the wealth gap—although a high average income value is good, a high average income value accompanied by low inequality is better. The integration of SDGs progress evenness and MIS into a single index holds potential to reduce the overestimation of SDGs performance when large progress differences exist across goals. Therefore, future studies and assessments of global sustainable development are suggested to consider both indices.

Our findings also emphasize that the major challenges of LICs and countries with extremely harsh climate conditions is to acquire and secure essential needs for human survival, such as food, water, and medicine. However, HICs should instead strive toward achieving a more even development pathway toward SDGs by paying more attention to biodiversity conservation and climate action. Given that LICs and countries with extremely harsh climate conditions generally performed better on SDGs 12 and 13 than did HICs, we argue that global collaborations over climate action may provide opportunities for solving the aforementioned problems in a mutually beneficial way. The integration of the suggestions provided in this study is expected to help narrow down the uneven progress across SDGs worldwide and support the holistic achievement of sustainable development. Thus, they should be implemented into the global roadmap toward SDGs.

REFERENCES

1. United Nations. (2015). Sustainable Development Goals: 17 Goals to Transform Our World (United Nations).
2. Naidoo, R., and Fisher, B. (2020). Sustainable Development Goals: Pandemic reset. Nature **583**: 198–201. https://doi.org/10.1038/d41586-020-01999-x.
3. Sachs, J., Lafortune, G., Kroll, C., et al. (2022). From Crisis to Sustainable Development: The SDGs as Roadmap to 2030 and beyond. Sustainable Development Report 2022 (Cambridge University Press).
4. Sachs, J., Schmidt-Traub, G., Kroll, C., et al. (2020). The Sustainable Development Goals and COVID-19. Sustainable Development Report 2020 (Cambridge University Press).
5. Di Marco, M., Baker, M.L., Daszak, P., et al. (2020). Sustainable development must account for pandemic risk. P. Natl. Acad. Sci. USA **117**: 3888–3892. https://doi.org/10.1073/pnas.2001655117.
6. Rohr, J.R., Barrett, C.B., Civitello, D.J., et al. (2019). Emerging human infectious diseases and the links to global food production. Nat. Sustain. **2**: 445–456. https://doi.org/10.1038/s41893-019-0293-3.
7. Springmann, M., Clark, M., Mason-D'Croz, D., et al. (2018). Options for keeping the food system within environmental limits. Nature **562**: 519–525. https://doi.org/10.1038/s41586-018-0594-0.
8. Di Marco, M., Watson, J.E.M., Currie, D.J., et al. (2018). The extent and predictability of the biodiversity–carbon correlation. Ecol. Lett. **21**: 365–375. https://doi.org/10.1111/ele.12903.
9. Hanspach, J., Abson, D.J., French Collier, N., et al. (2017). From trade-offs to synergies in food security and biodiversity conservation. Front. Ecol. Environ. **15**: 489–494. https://doi.org/10.1002/fee.1632.
10. Wu, X., Fu, B., Wang, S., et al. (2023). Three main dimensions reflected by national SDG performance. Innovation **4**: 100507. https://doi.org/10.1016/j.xinn.2023.100507.
11. Le Quéré, C., Jackson, R.B., Jones, M.W., et al. (2020). Temporary reduction in daily global CO2 emissions during the COVID-19 forced confinement. Nat. Clim. Change **10**: 647–653. https://doi.org/10.1038/s41558-020-0797-x.
12. Giani, P., Castruccio, S., Anav, A., et al. (2020). Short-term and long-term health impacts of air pollution reductions from COVID-19 lockdowns in China and Europe: a modelling study. Lancet Planet. Health **4**: e474–e482. https://doi.org/10.1016/S2542-5196(20)30224-2.
13. Sachs, J., Kroll, C., Lafortune, G., et al. (2021). The Decade of Action for the Sustainable Development Goals: Sustainable Development Report 2021 (Cambridge University Press).
14. Jiang, Q., Xu, Z., and Zhang, H. (2022). Global impacts of COVID-19 on sustainable ocean development. Innovation **3**: 100250. https://doi.org/10.1016/j.xinn.2022.100250.
15. Chinazzi, M., Davis, J.T., Ajelli, M., et al. (2020). The effect of travel restrictions on the spread of the 2019 novel coronavirus (COVID-19) outbreak. Science **368**: 395–400. https://doi.org/10.1126/science.aba9757.
16. Pradhan, P., Costa, L., Rybski, D., et al. (2017). A systematic study of Sustainable Development Goal (SDG) interactions. Earth's Future **5**: 1169–1179. https://doi.org/10.1002/2017EF000632.
17. Nilsson, M., Griggs, D., and Visbeck, M. (2016). Policy: Map the interactions between Sustainable Development Goals. Nature **534**: 320–322. https://doi.org/10.1038/534320a.
18. Fu, B., Wang, S., Zhang, J., et al. (2019). Unravelling the complexity in achieving the 17 sustainable-development goals. Natl. Sci. Rev. **6**: 386–388. https://doi.org/10.1093/nsr/nwz038.
19. Pradhan, P. (2019). Antagonists to meeting the 2030 Agenda. Nat. Sustain. **2**: 171–172. https://doi.org/10.1038/s41893-019-0248-8.
20. Warchold, A., Pradhan, P., and Kropp, J.P. (2020). Variations in sustainable development goal interactions: Population, regional, and income disaggregation. Sustain. Dev. **29**: 285–299. https://doi.org/10.1002/sd.2145.
21. Pedercini, M., Arquitt, S., Collste, D., et al. (2019). Harvesting synergy from sustainable development goal interactions. P. Natl. Acad. Sci. USA **116**: 23021–23028. https://doi.org/10.1073/pnas.1817276116.
22. Xu, Z., Chau, S.N., Chen, X., et al. (2020). Assessing progress towards sustainable development over space and time. Nature **577**: 74–78. https://doi.org/10.1038/s41586-019-1846-3.
23. Sachs, J., Schmidt-Traub, G., Kroll, C., et al. (2016). An SDG Index and Dashboards - Global Report (Bertelsmann Stiftung and Sustainable Development Solutions Network (SDSN)).
24. Liu, Y., Du, J., Wang, Y., et al. (2021). Evenness is important in assessing progress towards sustainable development goals. Natl. Sci. Rev. **8**: nwaa238. https://doi.org/10.1093/nsr/nwaa238.
25. Liu, J., and Raven, P.H. (2010). China's Environmental Challenges and Implications for the World. Crit. Rev. Environ. Sci. Technol. **40**: 823–851. https://doi.org/10.1080/10643389.2010.502645.
26. Rodell, M., Famiglietti, J.S., Wiese, D.N., et al. (2018). Emerging trends in global freshwater availability. Nature **557**: 651–659. https://doi.org/10.1038/s41586-018-0123-1.
27. Chan, C.K., and Yao, X. (2008). Air pollution in mega cities in China. Atmos. Environ. X. **42**: 1–42. https://doi.org/10.1016/j.atmosenv.2007.09.003.
28. Zhu, J., Yang, Y., Liu, Y., et al. (2023). Progress and water stress of sustainable development in Chinese northern drylands. J. Clean. Prod. **399**: 136611. https://doi.org/10.1016/j.jclepro.2023.136611.
29. Smith, B., and Wilson, J.B. (1996). A Consumer's Guide to Evenness Indices. Oikos **76**: 70–82. https://doi.org/10.2307/3545749.
30. Sachs, J., Schmidt-Traub, G., Kroll, C., et al. (2017). SDG Index and Dashboards Report 2017 (Bertelsmann Stiftung and Sustainable Development Solutions Network (SDSN)).
31. Sachs, J., Schmidt-Traub, G., Kroll, C., et al. (2018). SDG Index and Dashboards Report 2018 (Bertelsmann Stiftung and Sustainable Development Solutions Network (SDSN)).
32. Sachs, J., Schmidt-Traub, G., Kroll, C., et al. (2019). Sustainable Development Report 2019 (Bertelsmann Stiftung and Sustainable Development Solutions Network (SDSN)).
33. Zhao, B., Johnston, F.H., Salimi, F., et al. (2020). Short-term exposure to ambient fine particulate matter and out-of-hospital cardiac arrest: a nationwide case-crossover study in Japan. Lancet Planet. Health **4**: e15–e23. https://doi.org/10.1016/S2542-5196(19)30262-1.
34. Burnett, R., Chen, H., Szyszkowicz, M., et al. (2018). Global estimates of mortality associated with long-term exposure to outdoor fine particulate matter. P. Natl. Acad. Sci. USA **115**: 9592–9597. https://doi.org/10.1073/pnas.1803222115.
35. Bowe, B., Xie, Y., Li, T., et al. (2018). The 2016 global and national burden of diabetes mellitus attributable to PM2.5 air pollution. Lancet Planet. Health **2**: e301–e312. https://doi.org/10.1016/S2542-5196(18)30140-2.
36. Brønnum-Hansen, H., and Baadsgaard, M. (2007). Increasing social inequality in life expectancy in Denmark. Eur. J. Publ. Health **17**: 585–586. https://doi.org/10.1093/eurpub/ckm045.
37. Singh, G.K., and Siahpush, M. (2006). Widening socioeconomic inequalities in US life expectancy, 1980-2000. Int. J. Epidemiol. **35**: 969–979. https://doi.org/10.1093/ije/dyl083.
38. Strassburg, B.B.N., Iribarrem, A., Beyer, H.L., et al. (2020). Global priority areas for ecosystem restoration. Nature **586**: 724–729. https://doi.org/10.1038/s41586-020-2784-9.

39. Keesing, F., Belden, L.K., Daszak, P., et al. (2010). Impacts of biodiversity on the emergence and transmission of infectious diseases. Nature ***468***: 647–652. https://doi.org/10.1038/nature09575.
40. Allen, T., Murray, K.A., Zambrana-Torrelio, C., et al. (2017). Global hotspots and correlates of emerging zoonotic diseases. Nat. Commun. ***8***: 1124. https://doi.org/10.1038/s41467-017-00923-8.
41. Jones, K.E., Patel, N.G., Levy, M.A., et al. (2008). Global trends in emerging infectious diseases. Nature ***451***: 990–993. https://doi.org/10.1038/nature06536.
42. Huang, J., Yu, H., Guan, X., et al. (2016). Accelerated dryland expansion under climate change. Nat. Clim. Change ***6***: 166–171. https://doi.org/10.1038/nclimate2837.
43. Cheng, H. (2020). Future Earth and Sustainable Developments. Innovation ***1***: 100055. https://doi.org/10.1016/j.xinn.2020.100055.
44. Tian, H., Xu, R., Canadell, J.G., et al. (2020). A comprehensive quantification of global nitrous oxide sources and sinks. Nature ***586***: 248–256. https://doi.org/10.1038/s41586-020-2780-0.
45. Felipe, J., Abdon, A., and Kumar, U. (2012). Tracking the Middle-Income Trap: What Is it, Who Is in it, and Why? (Levy Economics Institute), p. 715.
46. Kharas, H., and Kohli, H. (2011). What is the middle income trap, why do countries fall into it, and how can it be avoided? Global Journal of Emerging Market Economies ***3***: 281–289. https://doi.org/10.1177/097491011100300302.
47. Egger, D., Miguel, E., Warren, S.S., et al. (2021). Falling living standards during the COVID-19 crisis: Quantitative evidence from nine developing countries. Sci. Adv. ***7***: eabe0997. https://doi.org/10.1126/sciadv.abe0997.
48. Zhuang, M., Lu, X., Peng, W., et al. (2021). Opportunities for household energy on the Qinghai-Tibet Plateau in line with United Nations' Sustainable Development Goals. Renew. Sustain. Energy Rev. ***144***: 110982. https://doi.org/10.1016/j.rser.2021.110982.
49. Nijsse, F.J.M.M., Mercure, J.F., Ameli, N., et al. (2023). The momentum of the solar energy transition. Nat. Commun. ***14***: 6542. https://doi.org/10.1038/s41467-023-41971-7.
50. International Energy Agency (2020). Clean Energy Innovation (IEA).
51. Griscom, B.W., Adams, J., Ellis, P.W., et al. (2017). Natural climate solutions. P. Natl. Acad. Sci. USA ***114***: 11645–11650. https://doi.org/10.1073/pnas.1710465114.
52. Anderson, C.M., DeFries, R.S., Litterman, R., et al. (2019). Natural climate solutions are not enough. Science ***363***: 933–934. https://doi.org/10.1126/science.aaw2741.
53. Zimmerer, K.S., Duvall, C.S., Jaenicke, E.C., et al. (2021). Urbanization and agrobiodiversity: Leveraging a key nexus for sustainable development. One Earth ***4***: 1557–1568. https://doi.org/10.1016/j.oneear.2021.10.012.
54. United Nations Environment Programme. (2021). UNEP Medium-Term Strategy 2022-2025 (UNEP).

ACKNOWLEDGMENTS

Part of this work was generated based on the Sustainable Development Reports (https://www.sdgindex.org/). We are grateful to all of the participants who contributed to the assessment. This work was supported by the National Natural Science Foundation of China (42001267, 42041005, and 42041007), the International Partnership Program of Chinese Academy of Sciences (121311KYSB20170004-04), and the Chinese Academy of Sciences Strategic Priority Research Program (A) (grant no. XDA20050103).

AUTHOR CONTRIBUTIONS

Y.W. and J. Du designed the research. Y.L. and J. Du analyzed the data and wrote the first draft of the manuscript. A.X. helped to analyze the data related to the spatial pattern. All of the other authors provided comments on the manuscript. All of the authors reviewed the manuscript.

DECLARATION OF INTERESTS

All of the authors declare no competing interests.

SUPPLEMENTAL INFORMATION

It can be found online at https://doi.org/10.1016/j.xinn.2024.100573.

LEAD CONTACT WEBSITE

Yanfen Wang https://people.ucas.edu.cn/~Wang.Yanfen.

The Innovation, Volume *5*

Supplemental Information

Overlooked uneven progress across sustainable development goals at the global scale: Challenges and opportunities

Yali Liu, Jianqing Du, Yanfen Wang, Xiaoyong Cui, Jichang Dong, Pan Gu, Yanbin Hao, Kai Xue, Hongbo Duan, Anquan Xia, Yi Hu, Zhi Dong, Bingfang Wu, Jürgen P. Kropp, and Bojie Fu

MATERIALS AND METHODS

Data sources

All SDG scores (the score for each SDG) and SDG index scores (the average of all 17 SDG scores) over time and space were based on SDG Index and Dashboards Reports from 2017 to 2021.[1-5] Briefly, SDG Index and Dashboards Reports adopt around 100 indicators from a mix of official and non-official data sources, generally following the official SDG indicators endorsed by the UN Statistical Commission. The value for each indicator is normalized to 0-100 by range transformation with carefully established upper and lower bounds for each indicator. The score for each SDG is calculated using the arithmetic mean of all corresponding SDG indicators' normalized values. These scores across all 17 SDGs are later averaged to calculate the SDG Index score (the mean SDG index score in the present study, MIS). More details about indicator selection, data source, normalization, calculations, and sensitivity tests can be found in those reports (https://www.sdgindex.org/reports/).

In SDG Index and Dashboards Reports and other well-established SDGs assessments,[4,6] the quantification of progress toward SDGs across countries and regions was mainly based on the SDG index score, which might be biased and over-optimistic when a large difference exists across 17 SDGs.[7] Therefore, the present study further quantified the progress evenness across all 17 SDG scores by the SDGs progress evenness score (ES) to diminish the potential overestimation of sustainable development performance by adopting the SDG index score only, then computed the sustainable development score (SDS) integrating both ES and MIS to quantify the progress toward SDGs at the global scale comprehensively. We further compared the SDGs progress evenness before and after the outbreak of COVID-19 based on the SDG Reports 2020 and 2021, respectively. The raw data used in the SDG report was mainly before the year the report was published (mostly in June) due to the lagging update of statistical data. Therefore, the SDG Report 2021, published in June 2021, reflected the first evaluation of global sustainable development post-COVID-19.

SDGs progress evenness score and sustainable development score

The computation of the evenness score and sustainable development score generally follows Liu *et al.*[7] with an improved radar chart method.[8] More details are quoted as follows:

The score of each SDG for a given country or region in a specific year is used as the radius of each sector, forming a radar chart with 17 sectors corresponding to the 17 SDGs. The area (S) and perimeter (L) of the radar chart are expressed as follows (Fig. S10):

$$S_i = \sum_{j=1}^{n} S_j = \sum_{j=1}^{n} \pi f_j {r_j}^2 \quad j = 1,2,\cdots,n \tag{1}$$

$$L_i = \sum_{j=1}^{n} L_j = 2|r_{max} - r_{min}| + \sum_{j=1}^{n} 2\pi f_j r_j \quad j = 1,2,\cdots,n \tag{2}$$

where n represents the number of SDGs, which is 17; r_{max} and r_{min} represent the maximum and minimum among 17 SDG scores, respectively; r_j refers to the score of the jth SDG; and f_j stands for the weight of the jth SDG and is set as 1/17 for all SDGs because there is no reason to give one SDG greater weight than another in the SDGs assessment, which focuses on the current SDGs progress status rather than future policy-making.[4,6,9] The evenness score refers to the ratio between the total area of the radar chart formed by the 17 SDGs and the area of a circle with the same perimeter (the evenest distribution with the same perimeter), which is calculated by S_i and L_i following equation (3). It is re-scaled to 0-100 by multiplying 100 to be comparable with the SDG index score. As the area of the radar chart with a given perimeter reaches the largest (100) when it is a circle and decreases with increasing uneven progress across all 17 SDGs, the evenness score (ES) is the highest when each SDG shares the same score. ES depicts the progress differences across the 17 SDGs, where a low value presents an uneven status or existing trade-offs among the SDGs.

$$ES = S_i/[\pi(L_i/2\pi)^2] \times 100 = 400\pi S_i/{L_i}^2 \tag{3}$$

The sustainable development score (SDS) is the geometric mean of the evenness score and the mean SDG index score, which diminishes the potential overestimation of sustainable development performance by using the mean SDG index score only in case there are large differences across 17 SDGs. All scores range from 0 (worst performance) to 100 (best performance).

Overall, evenness scores and sustainable development scores of 169 countries were calculated annually from 2017 to 2021. All countries were further grouped by geographic location, climate condition (by the aridity index), and economic status (by UN income groups in 2019). Aridity is chosen to represent the climate condition because 1) drylands are fragile ecosystems with limited ecosystem services and sensitive to climate changes and human activities, thus are among the most challenging regions to achieve SDGs,[10] and 2) dryland expansion is a representative outcome of the global climate change and is assumed to be accelerated in the future.[11,12] To be specific, six groups were defined by geographic location, i.e., Africa, Asia, Latin America and Caribbean (LAC), Oceania, Europe, and North America (North A.); four groups were divided by UN income groups in 2019,[3] namely, high-income countries (HIC), upper-middle-income countries (UMIC), lower-middle-income countries (LMIC), and lower-income countries (LIC); four groups were defined by the proportion of drylands in the total area of the national territory area,[13] namely, none or slightly arid (<50%), moderately arid (50-75%), severely arid (75-99%), extremely arid (>99%). Then, the differences in MIS, ES, and SDS among groups were explored.

Furthermore, the current development status for all countries was divided into four categories based on the quadrantal diagram (MIS vs. ES in 2021).[7] The threshold for relatively high and low MIS/ES was calculated based on the K-mean clustering analysis. With both thresholds, all countries were divided into four quadrants: 1) the first quadrant for relatively sustainably status with high MIS and ES; 2) the second quadrant for underdeveloped status with low MIS and high ES; 3) the third quadrant for underdeveloped and uneven status with low MIS and ES; 4) the fourth quadrant for uneven status with high MIS and low ES.

Effect of SDGs progress evenness on social and environmental indicators

The economy is strongly associated with achieving SDGs, and the top ten countries approaching SDGs are all HIC.[4] Therefore, SDGs progress evenness and GDP per capita were set as independent variables in the binary regression model to distinguish their partial effects on indicators related to human health, inequality, and the environment. Furthermore, we used path analysis to explore the direct and indirect (via $PM_{2.5}$, ratio of female-to-male mean year of education received, Gini coefficient, etc.) effects of SDGs progress evenness and GDP per capita on indicators related to public health, as public health could also be affected by social and environmental factors. The data for the indicators were from multiple sources found in the attached Supplementary Data file.

Development pathway and the effective development score

The development pathway and the effective development score were adopted to evaluate the progress toward SDGs over time. Generally, we followed the method of Liu *et al.*[7] in defining and computing the development pathway and the effective development score. The development pathway of a given country from 2017 to 2021 can be visualized by creating a vector within MIS-ES coordinates, starting with the pair-wise MIS (x_1) and ES (y_1) in 2017 and ending with the paired values in 2021 (x_2, y_2). As a result, four types of pathways are expected (namely, progress in both MIS and ES, retrogression in either MIS or ES, and retrogression in both MIS and ES). The ideal pathway was the vector with a slope of one, characterizing a simultaneous improvement in all 17 SDGs. The effective development score (EDS) refers to the projected length (red line in Fig. S11) of a given pathway (red vector) on the ideal pathway. EDS provides a more comprehensive estimation of progress toward SDGs over time than only interpreting the change of MIS or SDS. It can help to diminish the overestimation if the achievement is either the increase in mean SDG index score only (improvements only shown in a few SDGs) or the increase in evenness score only (improvement in the poorly achieved SDGs while retrogression in the better-accomplished SDGs).

$$EDS = \begin{cases} \cos(\theta - 45°) \times \sqrt{(x_1 - x_2)^2 + (y_1 - y_2)^2}, & -180° \leq \theta < -90° \\ \cos(45° - \theta) \times \sqrt{(x_1 - x_2)^2 + (y_1 - y_2)^2}, & -90° \leq \theta < 45° \\ \cos(\theta - 45°) \times \sqrt{(x_1 - x_2)^2 + (y_1 - y_2)^2}, & 45° \leq \theta < 180° \end{cases} \quad (4)$$

Projections of SDGs performance in 2030

Both MIS and SDS in 2030 were predicted based on curvilinear regressions and the gray forecast model using MIS or SDS in each country from 2017 to 2021. Eleven regression models, including linear, quadratic, cubic, compound, growth, logarithmic, S, exponential, inverse, power function, and Logistic models, were used in our analyses. The best model was determined based on the Akaike information criterion (AIC). All analyses were performed with SPSS v. 27 (IBM Corp., Armonk, NY, USA). The grey forecast model was performed with MATLAB R2020b software. The data matrix was constructed by the adjacent mean value and the parameters were obtained by the least square method. We compared the projections of two prediction methods in 2030 and used the average values of the two methods in Figure 4.

Urgency and potential for priority development

The present study proposed a framework for the priority selection by integrating the SDGs progress evenness:

1) All SDGs were divided into four categories generally based on Fu *et al.*,[14] namely, essential human needs (poverty, food, health, water, and energy) (SDG 1, 2, 3, 6, 7), eco-environmental protection (SDG 12, 13, 14, 15), social development (SDG 4, 5, 10, 16, 17), and economic development (SDG 8, 9, 11).

2) The performance for each category was evaluated by the average SDG score of all SDGs included in 2021. Countries with relatively poor performance on essential human needs (lower than 61.33 based on the K-mean clustering analysis) were considered urgent for priority development.

3) Among these countries, whose current status was uneven were chosen for further discussion as the priority development might amplify the uneven progress across SDGs and bring expected problems. A country with the worst performance on eco-environmental protection was considered to have a low potential for priority development.

4) Development pathways were then considered for those unevenly progressed countries with relatively better performance on eco-environmental protection. Countries with an even pathway were treated differently from those with an uneven pathway.

5) For those with an even pathway, if the average score for essential human needs of a given country increased during the study period, it was considered less urgent for priority developments, while countries with decreasing average score might need to consider an adjustment in their development by addressing the essential human needs.

6) The most urgent need for priority development was identified as the countries with an uneven pathway, which reflected in a decreasing average score for essential human needs, particularly the countries currently performing the worst on essential human needs.

Other statistical analyses

The statistical differences in MIS, ES, SDS, and EDS among groups were based on one-way ANOVA, and *p* values between groups were based on the least significant differences (LSD) test. The normal distribution test was performed first, while data that failed to pass the test was transformed by square root (MIS and SDS were moderately negatively skewed in this study). The present study also explored the synergies and trade-offs among all SDGs within different geographic/environmental/economic groups. Based on the Pearson correlation matrix calculated from scores of all SDGs across countries in 2021, synergies and trade-offs between two SDGs refer to those significant positive or negative correlations at $p < 0.05$, while those non-significant relationships are defined as uncorrelated.[15] The number of synergies, trade-offs, and uncorrelated relationships between a given

SDG and the other SDGs were recorded and compared to show how it interacts with other SDGs. All statistical analyses were performed with SPSS v. 27 (IBM Corp., Armonk, NY, USA).

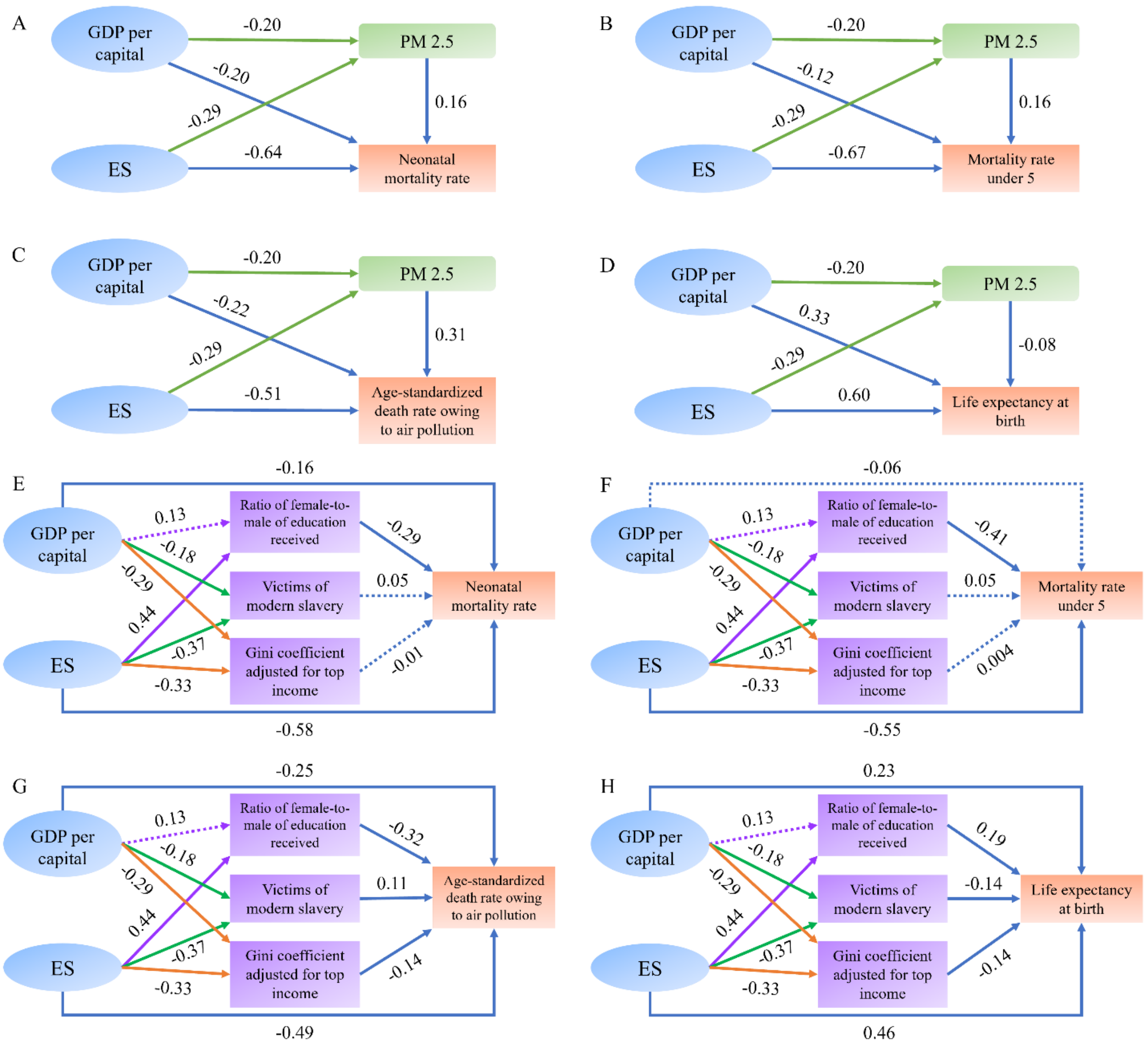

Fig. S1. Path analysis of the impact of GDP per capita and SDGs progress evenness (ES) on health-related indicators.

Solid and dashed lines represent significant and non-significant paths, respectively.

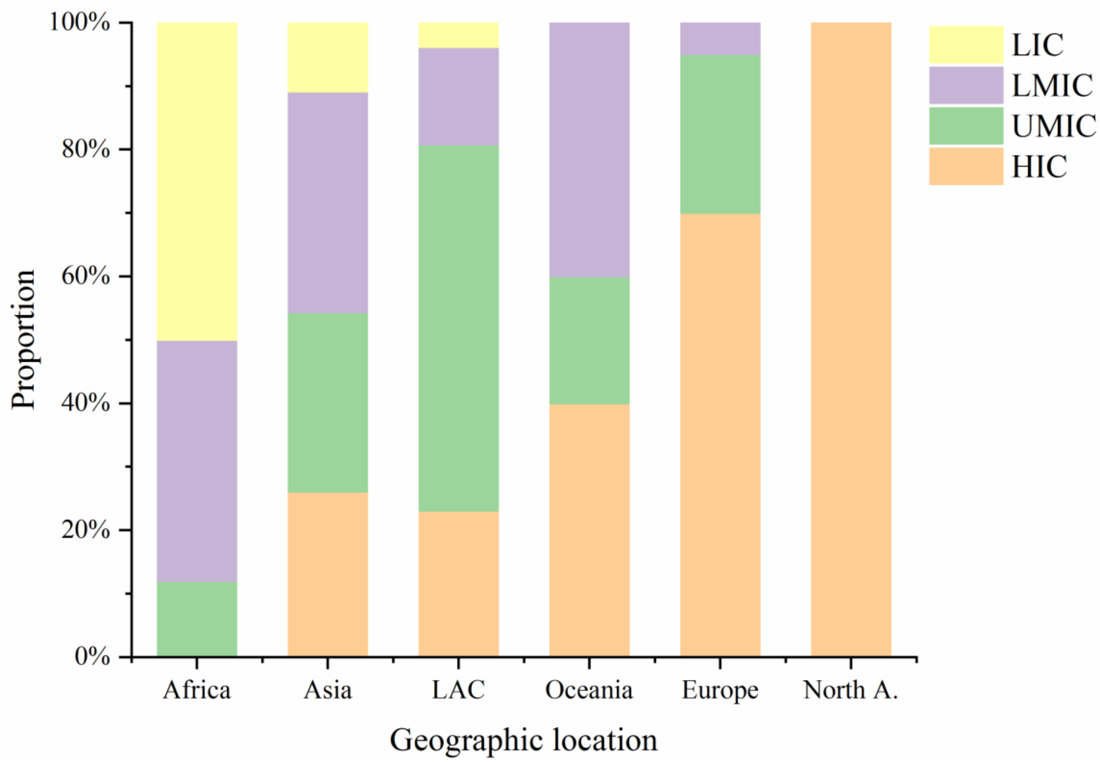

Fig. S2. The proportion of UN income groups in different regions.

HIC, UMIC, LMIC, and LIC stand for high-income, upper-middle-income, lower-middle-income, and lower-income countries, respectively. North A. is short for North America. LAC refers to Latin America and Caribbean.

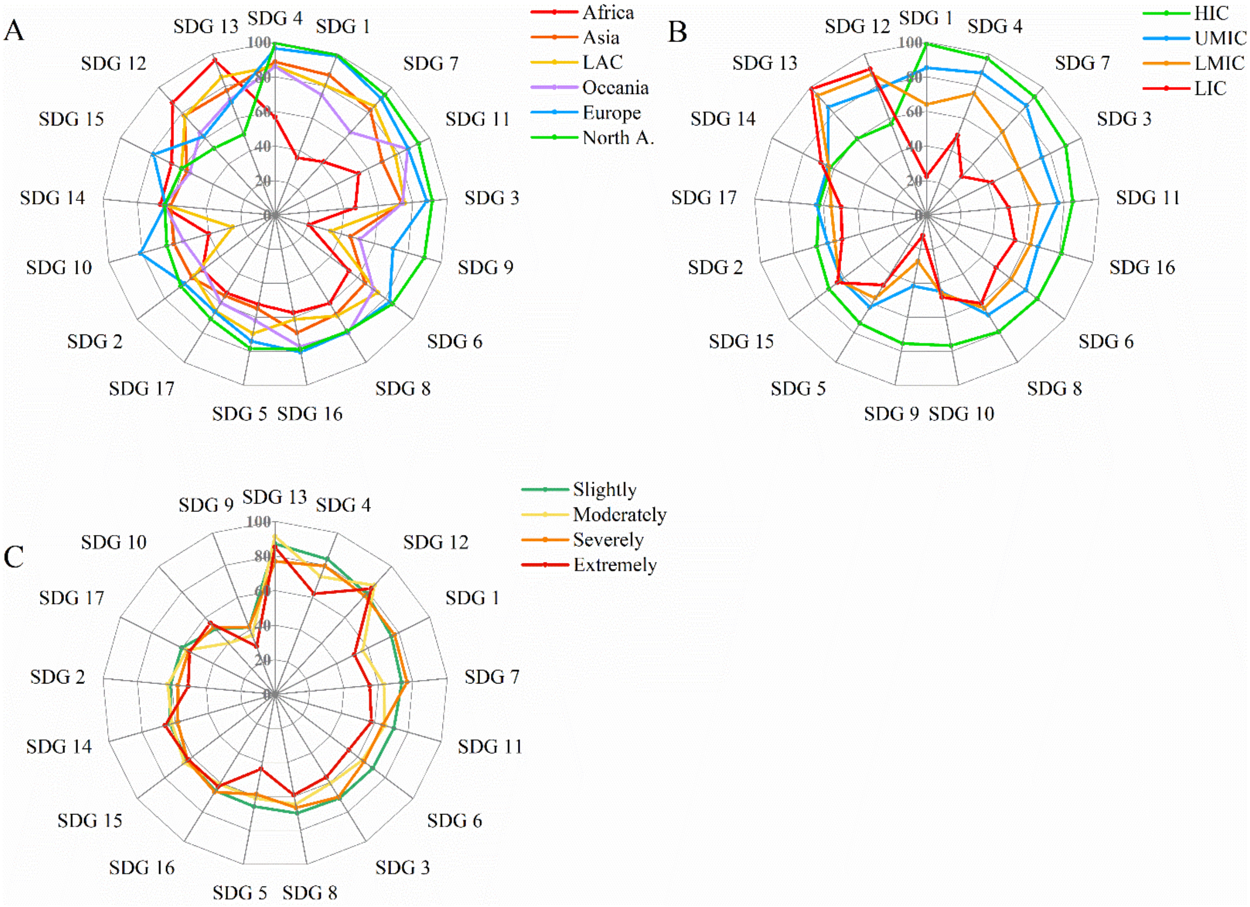

Fig. S3. Average SDG scores across different geographic locations (A), UN income groups (B), and arid levels (C) in 2021.The proportion of UN income groups in different regions.

The radar chart starts from the north with the SDG with the highest score of North America (in A), high-income countries (in B), and slightly arid countries (in C), respectively. North A. is short for North America. LAC refers to Latin America and Caribbean. HIC, UMIC, LMIC, and LIC stand for high-income, upper-middle-income, lower-middle-income, and lower-income countries, respectively.

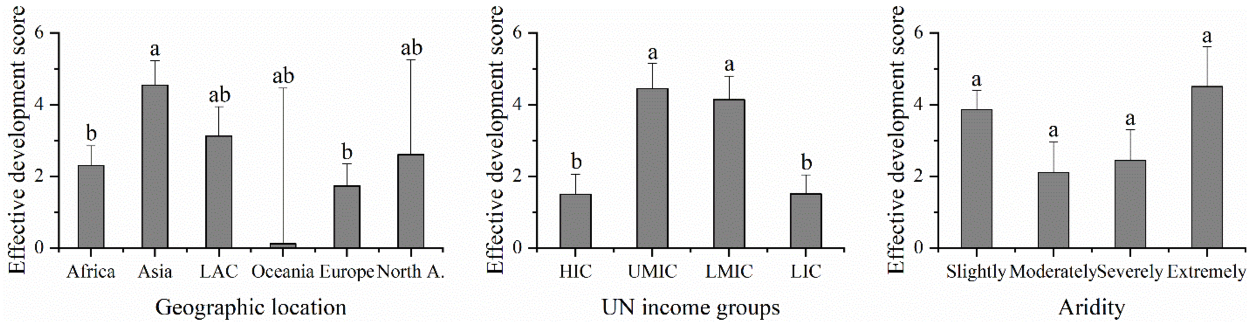

Fig. S4. Differences in effective development score across geographic locations, UN income groups, and arid levels in 2020.

HIC, UMIC, LMIC, and LIC stand for high-income, upper-middle-income, lower-middle-income, and lower-income countries, respectively. North A. is short for North America. LAC refers to Latin America and Caribbean. The histogram with error bars presents the mean value ± standard error (SE). Letters a and b visualize the significant differences at $p < 0.05$. EDS is based on data from 2017 to 2020.

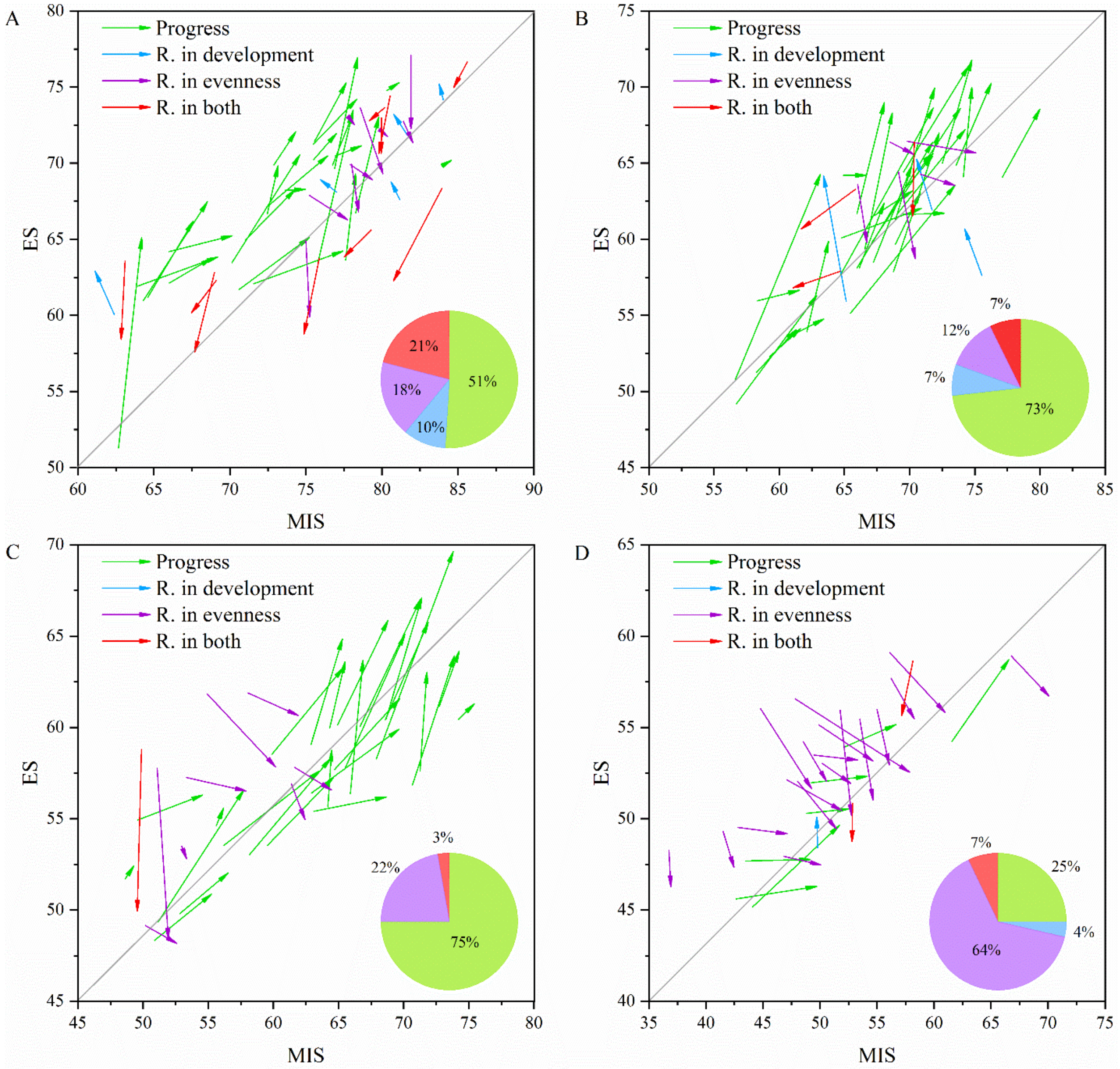

Fig. S5. Development pathways for different UN income groups from 2017 to 2020.

(A) high-income countries; (B) upper-middle-income countries; (C) lower-middle-income countries; (D) low-income countries. Different colors are used to represent the four types of pathways: progress (green), retrogression in development (R. in development, blue), retrogression in evenness (R. in evenness, purple), and retrogression in both (R. in both, red). The grey diagonal stands for the ideal pathway (slope = 1). The pie chart at the bottom right corner shows the proportion of different pathways.

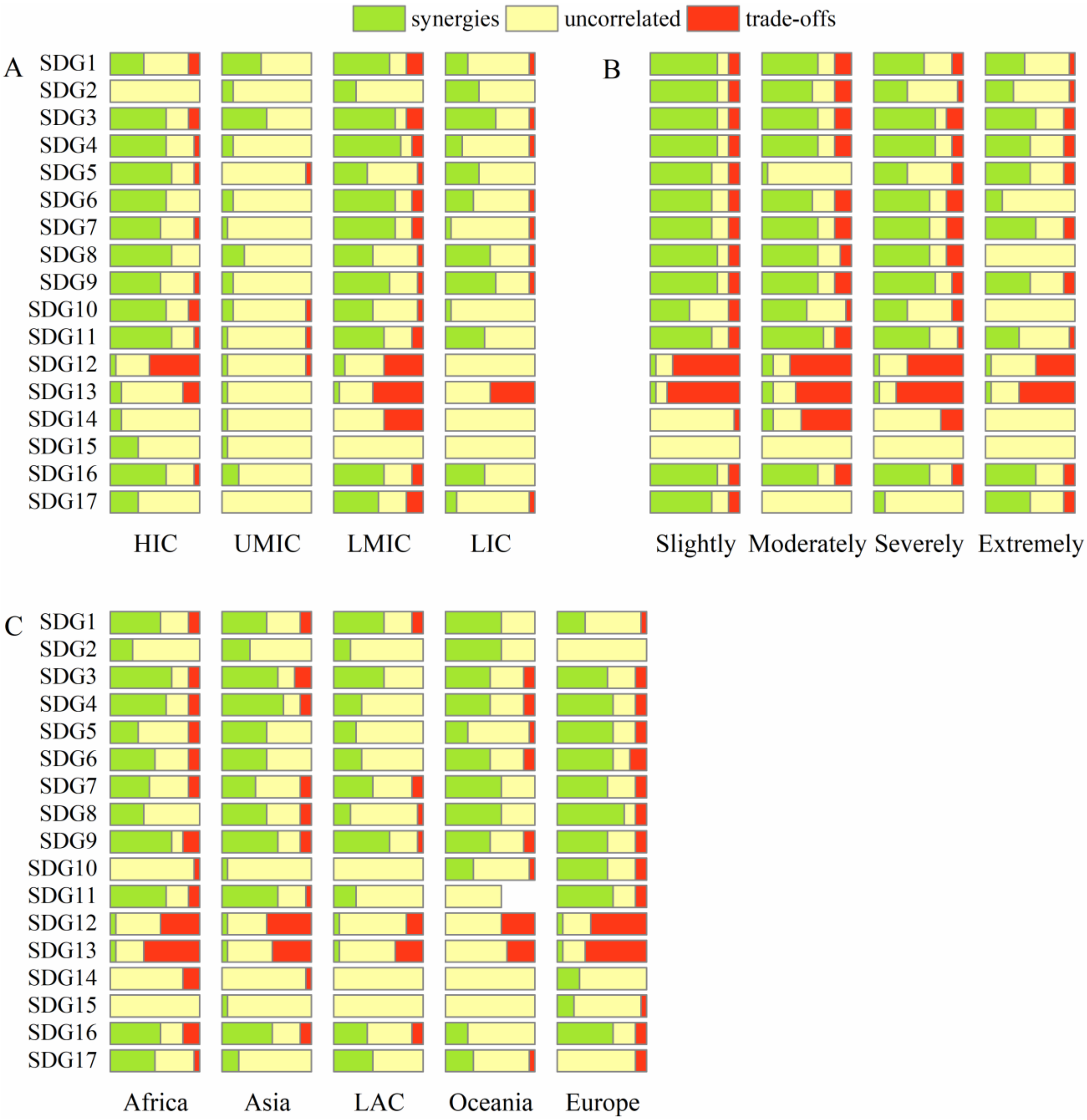

Fig. S6. Interactions among SDGs across different UN income groups (A), arid levels (B), and geographic locations (C) in 2021.

Green represents synergies with a significantly positive correlation at $p < 0.05$; red represents trade-offs with a significantly negative correlation at $p < 0.05$; and yellow represents non-significant correlations. HIC, UMIC, LMIC, and LIC stand for high-income, upper-middle-income, lower-middle-income, and lower-income countries, respectively. LAC refers to Latin America and Caribbean. North America is not involved in this analysis because only two countries exist.

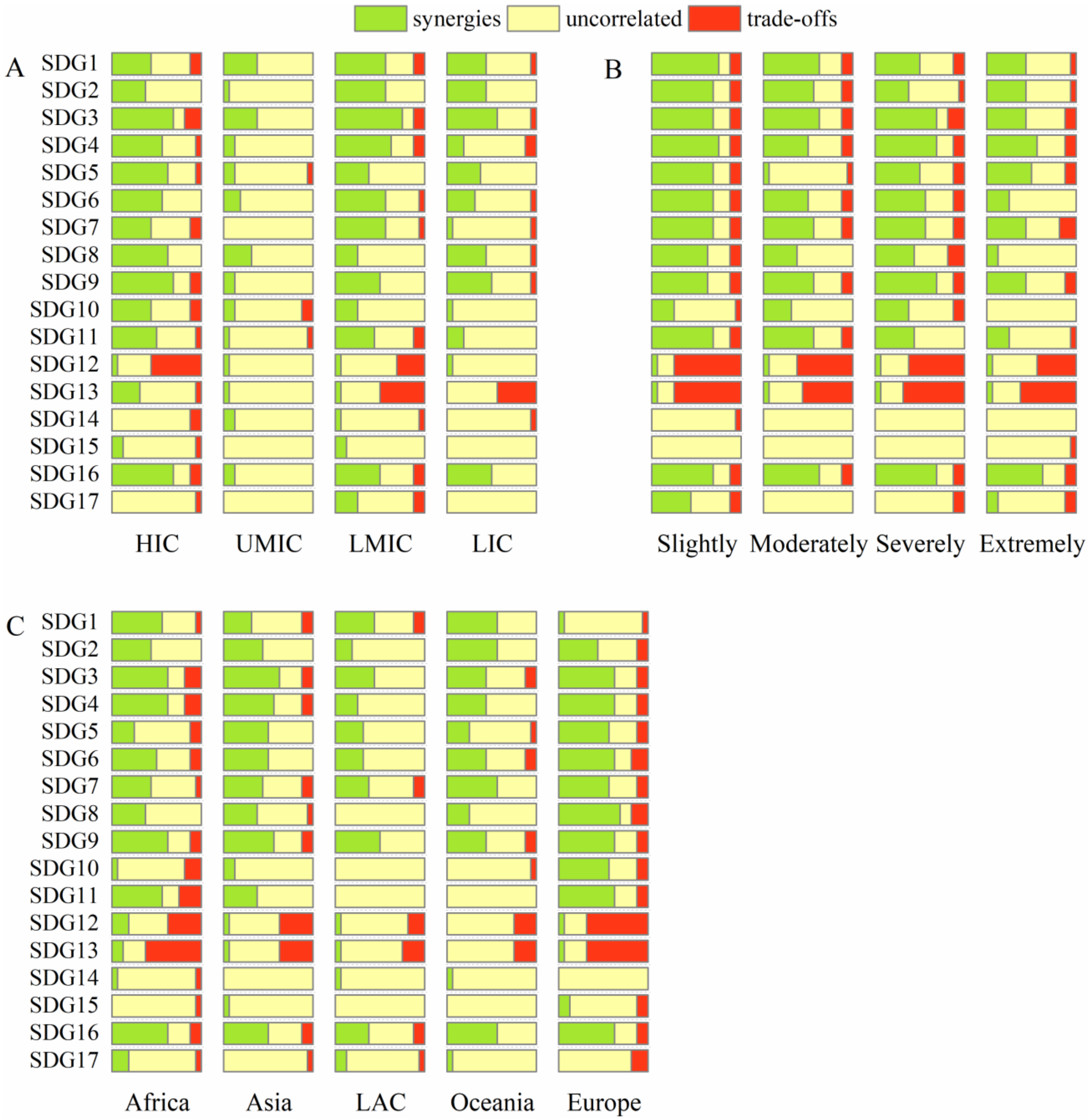

Fig. S7. Interactions among SDGs across different UN income groups (A), arid levels (B), and geographic locations (C) in 2020.

Green represents synergies with a significantly positive correlation at $p < 0.05$; red represents trade-offs with a significantly negative correlation at $p < 0.05$; and yellow represents non-significant correlations. HIC, UMIC, LMIC, and LIC stand for high-income, upper-middle-income, lower-middle-income, and lower-income countries, respectively. LAC refers to Latin America and Caribbean. North America is not involved in this analysis because only two countries exist.

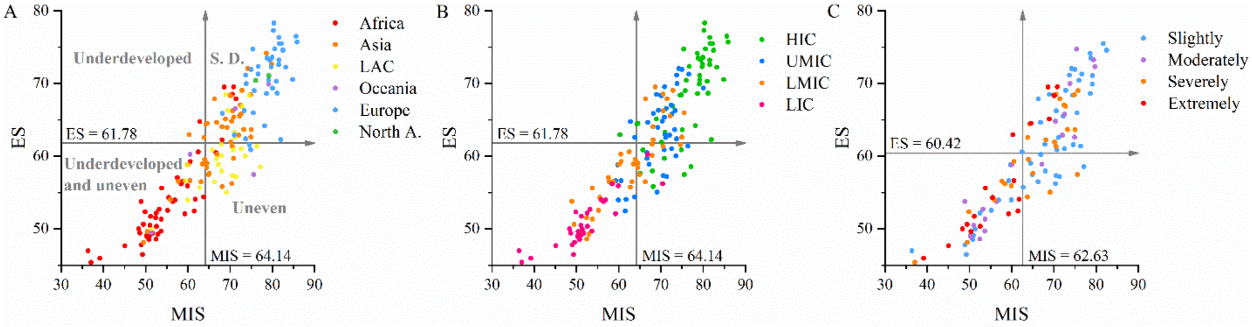

Fig. S8. Sustainable development status for countries across different geographic locations (A), UN income groups (B), and arid levels (C) in 2021.

HIC, UMIC, LMIC, and LIC stand for high-income, upper-middle-income, lower-middle-income, and lower-income countries, respectively. LAC refers to Latin America and Caribbean; North A. stands for North America. S. D. refers to the relatively sustainably developed status.

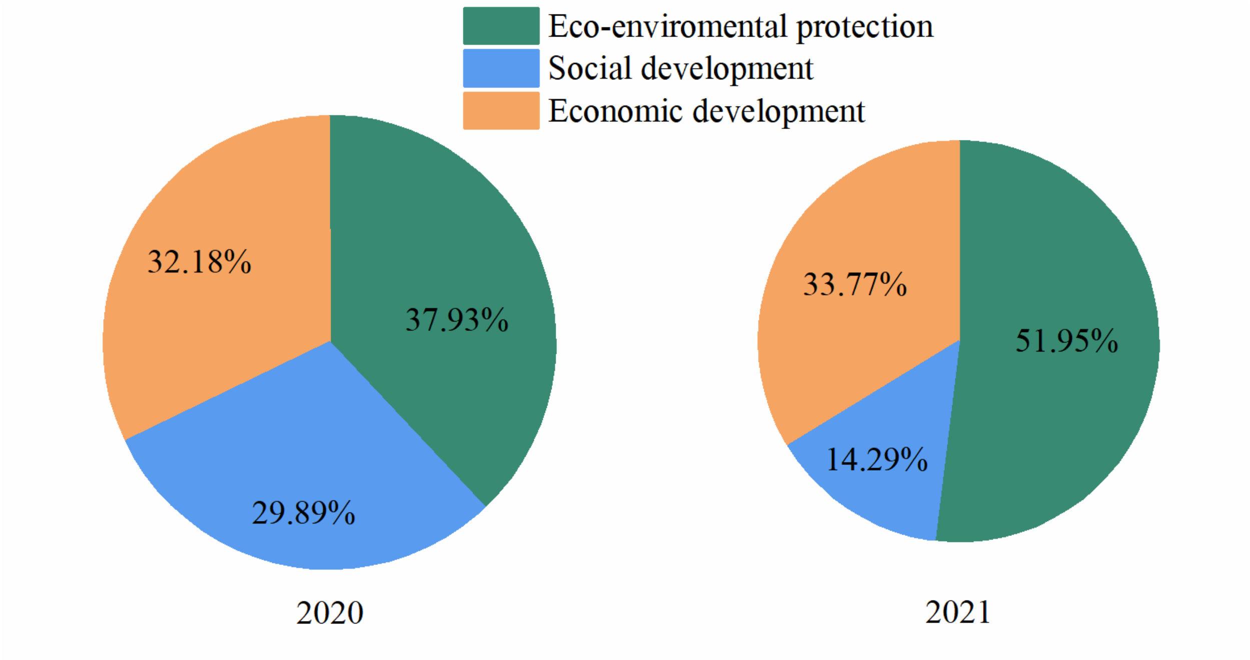

Fig. S9. Proportion of deficiencies in countries considered relatively sustainably developed in 2020 and 2021.

87 and 77 countries are considered relatively sustainably developed in 2020 and 2021, respectively.

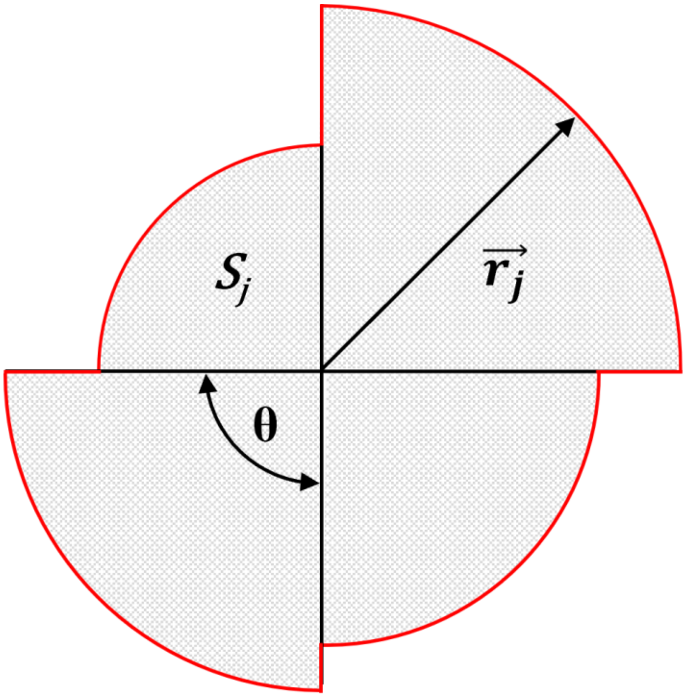

Fig. S10. Outline of the radar chart method.

r_j stands for the score of the jth SDG. θ represents the weight of each SDG, which refers to f_j in equations (1) and (2), and is set up to 1/17 for all SDGs in the present study. The total area of all sectors refers to S_i in equation (1). The red line presents the total perimeter of all sectors, referring to L_i in equation (2).

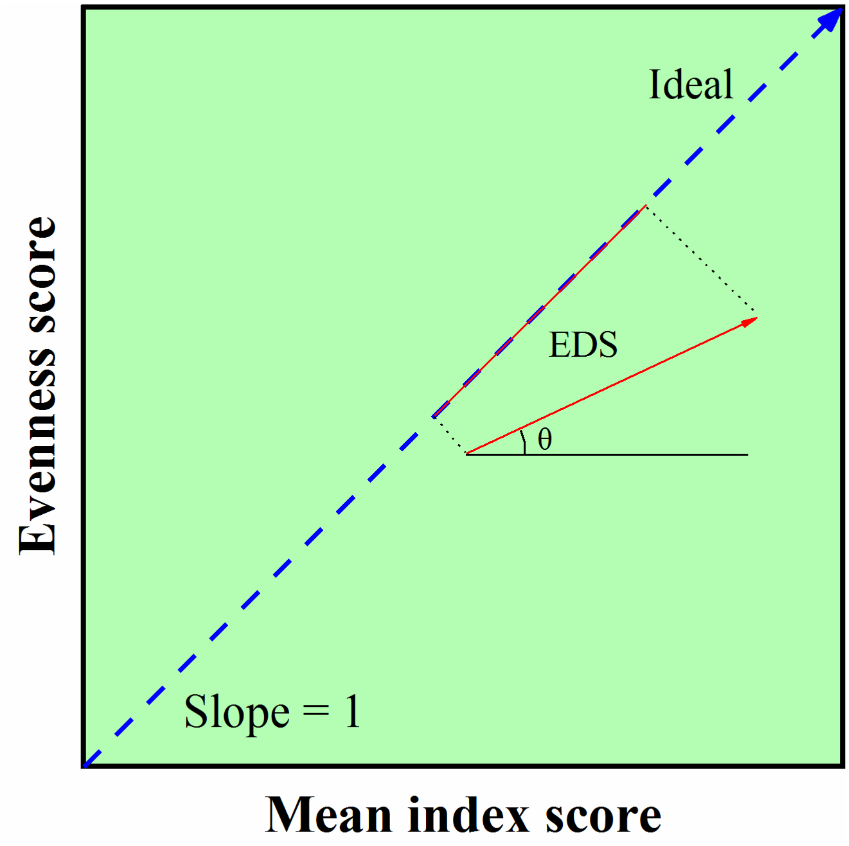

Fig. S11. Outline of the development pathway and the effective development score (EDS)[7].

θ is the angle between a given vector (marked in red) and the x-axis.

Table S1. Effects of GDP per capita and the annual mean concentration of particulate matter of less than 2.5 microns in diameter ($PM_{2.5}$) on the indicators related to health.

Factor	Neonatal mortality rate (per 1000 live births) $R^2 = 0.39$, $F_{2,157} = 51.55$			Mortality rate under 5 (per 1000 live births) $R^2 = 0.32$, $F_{2,157} = 39.04$			Age-standardized death rate due to air pollution (per 100,000 population) $R^2 = 0.49$, $F_{2,157} = 77.18$			Life expectancy at birth (years) $R^2 = 0.45$, $F_{2,157} = 65.98$			VIF
	β	t	p	β	t	p	β	t	p	β	t	p	
GDP per capita	-0.45	-6.90	<0.001	-0.39	-5.57	<0.001	-0.43	-7.15	<0.001	0.57	9.10	<0.001	1.125
$PM_{2.5}$	0.31	4.72	<0.001	0.32	4.61	<0.001	0.43	7.19	<0.001	-0.22	-3.58	<0.001	

All models are significant at $p < 0.05$. VIF: variance inflation factor.

Table S2. Raw data for the mean index score (MIS), evenness score (ES), sustainable development score (SDS), and effective development score (EDS) across geographic locations, UN income groups, and arid levels. HIC, UMIC, LMIC, and LIC stand for high-income, upper-middle-income, lower-middle-income, and lower-income countries, respectively. LAC refers to Latin America and Caribbean. N.A. is short for not available.

Countries	UN regions	Income Group in 2019	Dryland area (%)	2017			2018			2019			2020			2021			EDS	EDS
				MIS	ES	SDS	MIS	ES	SDS	MIS	ES	SDS	MIS	ES	SDS	MIS	ES	SDS	2017-2020	2017-2021
Afghanistan	Asia	LIC	88.86	46.81	47.95	47.38	46.24	49.06	47.63	45.73	47.24	46.48	50.09	47.47	48.76	49.55	48.13	48.83	1.98	2.06
Albania	Europe	UMIC	N. A.	68.90	59.66	64.12	68.91	60.11	64.36	70.27	62.46	66.25	70.82	63.06	66.83	71.02	62.91	66.85	3.76	3.80
Algeria	Africa	UMIC	99.16	68.76	57.86	63.08	67.88	60.34	64.00	71.10	62.76	66.80	72.27	66.97	69.57	70.86	69.54	70.20	8.92	9.74
Angola	Africa	LMIC	52.79	50.18	49.15	49.66	49.56	49.47	49.52	51.32	47.88	49.57	52.59	48.18	50.34	50.30	49.06	49.68	1.01	0.02
Argentina	LAC	HIC	79.83	72.45	66.68	69.50	70.28	65.78	67.99	72.35	68.10	70.19	73.17	69.82	71.47	72.80	66.88	69.78	2.73	0.39
Armenia	Asia	UMIC	62.06	71.72	61.93	66.65	69.27	61.34	65.18	70.53	65.11	67.77	70.54	65.26	67.85	72.60	65.79	69.11	1.52	3.35
Australia	Oceania	HIC	91.08	75.87	63.74	69.54	72.89	60.84	66.59	73.89	65.58	69.61	74.87	58.79	66.34	75.58	57.46	65.90	-4.21	-4.66
Austria	Europe	HIC	N. A.	81.42	72.74	76.96	79.95	72.42	76.09	82.93	70.55	76.49	82.03	71.36	76.51	83.25	72.23	77.54	-0.54	0.93
Azerbaijan	Asia	UMIC	83.13	70.81	64.28	67.47	70.80	64.14	67.38	70.46	59.10	64.53	73.46	63.54	68.32	73.25	63.64	68.28	1.36	1.28
Bahrain	Asia	HIC	84.88	64.59	61.19	62.87	65.90	61.46	63.64	66.98	68.68	67.82	67.58	66.20	66.88	64.30	64.40	64.35	5.65	2.06
Bangladesh	Asia	LMIC	N. A.	56.21	53.54	54.86	59.35	54.14	56.68	60.88	54.22	57.45	63.51	57.60	60.48	63.45	58.90	61.13	8.03	8.91
Barbados	LAC	HIC	N. A.	65.98	64.20	65.08	N. A.	N. A.	N. A.	N. A.	N. A.	N. A.	70.15	65.24	67.65	70.98	63.29	67.03	3.69	2.90
Belarus	Europe	UMIC	N. A.	77.12	64.10	70.31	75.99	63.60	69.52	79.74	67.06	73.13	80.00	68.55	74.05	80.07	69.37	74.53	5.18	5.81
Belgium	Europe	HIC	N. A.	79.96	72.97	76.38	79.00	70.28	74.51	78.89	64.22	71.18	79.96	70.65	75.16	82.19	75.48	78.76	-1.64	3.35
Belize	LAC	UMIC	N. A.	66.02	63.61	64.80	62.32	60.07	61.19	64.14	61.61	62.86	66.74	59.77	63.16	66.71	59.68	63.10	-2.21	-2.29
Benin	Africa	LIC	94.55	49.47	53.48	51.44	48.98	51.59	50.27	50.85	51.07	50.96	53.31	53.22	53.26	49.87	52.34	51.09	2.53	-0.52
Bhutan	Asia	LMIC	25.9	65.51	57.78	61.52	65.39	59.43	62.34	68.90	61.06	64.86	70.08	65.08	67.54	70.56	66.23	68.36	8.40	9.55
Bolivia	LAC	LMIC	47.71	64.69	57.69	61.09	68.08	58.71	63.22	69.31	60.52	64.76	69.70	61.56	65.50	67.90	61.64	64.69	6.28	5.06
Bosnia and Herzegovina	Europe	UMIC	N. A.	65.45	55.13	60.07	67.31	55.37	61.05	69.39	55.87	62.26	73.48	63.54	68.33	73.70	62.40	67.81	11.62	10.97
Botswana	Africa	UMIC	99.99	58.26	51.26	54.65	58.46	53.01	55.67	60.09	52.57	56.21	61.27	54.05	57.55	61.54	52.46	56.82	4.10	3.17
Brazil	LAC	UMIC	13.99	69.51	61.63	65.45	69.69	60.43	64.89	70.62	61.61	65.96	72.67	61.72	66.97	71.34	57.17	63.86	2.30	-1.86
Brunei Darussalam	Asia	HIC	N. A.	N. A.	N. A.	N. A.	N. A.	N. A.	N. A.	N. A.	N. A.	N. A.	67.84	55.19	61.19	68.16	55.80	61.67	N. A.	N. A.
Bulgaria	Europe	UMIC	57.75	72.51	65.01	68.66	73.13	65.31	69.11	74.52	67.43	70.88	74.77	71.77	73.26	73.81	69.99	71.88	6.38	4.44
Burkina Faso	Africa	LIC	99.99	49.90	55.13	52.45	50.88	52.54	51.70	52.27	53.90	53.08	54.64	53.16	53.90	52.60	50.35	51.46	1.96	-1.47
Burundi	Africa	LIC	17.54	51.77	55.94	53.81	49.82	53.56	51.66	51.36	50.25	50.80	52.77	50.17	51.45	50.79	48.54	49.65	-3.37	-5.92
Cabo Verde	Africa	LMIC	92.8	N. A.	N. A.	N. A.	64.68	63.38	64.02	65.05	61.51	63.26	67.18	60.80	63.91	68.14	62.26	65.13	N. A.	N. A.
Cambodia	Asia	LMIC	0.34	58.17	53.04	55.55	60.38	55.38	57.82	61.78	57.03	59.36	64.39	58.30	61.27	64.54	59.01	61.71	8.11	8.72
Cameroon	Africa	LMIC	14.12	52.83	49.80	51.29	55.78	50.38	53.01	56.02	51.39	53.66	56.54	52.03	54.24	55.26	52.53	53.88	4.20	3.65
Canada	North America	HIC	17.49	78.02	72.75	75.34	76.79	72.34	74.53	77.89	71.53	74.64	78.19	72.53	75.31	79.16	71.10	75.02	-0.03	-0.36
Central African Republic	Africa	LIC	29.84	36.75	48.27	42.12	37.66	49.05	42.98	38.11	50.13	43.71	36.92	46.28	41.34	36.41	46.97	41.35	-1.29	-1.16
Chad	Africa	LIC	99.83	41.50	49.30	45.23	42.81	48.62	45.62	42.05	46.89	44.41	42.46	47.34	44.83	39.21	45.93	42.44	-0.71	-4.00
Chile	LAC	HIC	48.43	71.57	62.08	66.65	72.79	61.51	66.91	75.61	62.47	68.73	77.42	64.23	70.52	77.13	58.53	67.19	5.66	1.43
China	Asia	UMIC	56.48	67.12	59.82	63.36	70.05	61.65	65.72	73.21	64.42	68.67	73.89	68.60	71.20	72.06	62.88	67.32	11.00	5.66
Colombia	LAC	UMIC	2.95	64.80	60.10	62.41	66.61	59.17	62.78	69.57	61.91	65.63	70.91	62.03	66.32	70.56	57.31	63.59	5.68	2.10
Comoros	Africa	LIC	N. A.	N. A.	N. A.	N. A.	N. A.	N. A.	N. A.	52.98	49.85	51.39	53.07	49.77	51.39	N. A.	N. A.	N. A.	N. A.	N. A.
Congo, Dem. Rep.	Africa	LIC	7.78	42.66	45.61	44.11	43.39	46.63	44.98	44.95	46.28	45.61	49.71	46.29	47.97	49.30	46.47	47.86	5.47	5.30
Congo, Rep.	Africa	LMIC	N. A.	50.88	48.34	49.60	52.38	52.41	52.40	54.22	52.35	53.28	55.25	50.87	53.02	52.95	49.25	51.07	4.88	2.10
Costa Rica	LAC	UMIC	N. A.	69.81	66.44	68.10	73.15	64.54	68.71	74.98	65.64	70.15	75.08	65.69	70.23	73.55	60.02	66.44	3.19	-1.89
Cote d'Ivoire	Africa	LMIC	N. A.	53.34	57.25	55.26	55.18	55.97	55.57	55.70	56.82	56.26	57.91	56.52	57.21	57.56	57.04	57.30	2.72	2.84
Croatia	Europe	HIC	N. A.	76.88	69.67	73.19	76.52	70.91	73.66	77.79	70.66	74.14	78.40	76.94	77.67	80.38	78.33	79.35	6.22	8.60
Cuba	LAC	UMIC	6.8	75.55	57.63	65.99	71.34	56.89	63.71	72.37	56.65	64.03	74.23	60.67	67.11	76.36	61.37	68.46	1.21	3.21
Cyprus	Asia	HIC	89.14	70.60	61.69	66.00	70.36	60.18	65.07	70.14	61.65	65.76	75.21	65.11	69.98	74.87	68.44	71.58	5.68	7.79
Czech Republic	Europe	HIC	2.56	81.90	77.10	79.46	78.72	71.25	74.89	82.57	74.96	78.67	81.90	72.27	76.93	82.51	74.59	78.45	-3.42	-1.34
Denmark	Europe	HIC	N. A.	84.16	69.96	76.73	84.61	73.37	78.79	85.22	72.83	78.78	84.56	70.19	77.04	84.86	70.54	77.37	0.44	0.90
Djibouti	Africa	LMIC	99.84	49.59	54.93	52.19	50.63	54.00	52.29	51.36	53.42	52.38	54.56	56.29	55.42	53.76	55.58	54.67	4.48	3.41
Dominican	LAC	UMIC	22.56	67.22	58.48	62.70	66.42	59.87	63.06	69.76	62.68	66.13	70.17	64.82	67.44	70.76	60.87	65.63	6.57	4.19

Republic																				
Ecuador	LAC	UMIC	18.32	69.04	61.66	65.25	70.77	62.73	66.63	72.29	63.91	67.97	74.26	67.21	70.65	72.54	61.02	66.53	7.61	2.01
Egypt, Arab Rep.	Africa	LMIC	99.8	64.92	60.13	62.48	63.47	61.24	62.35	66.21	62.08	64.11	68.79	65.86	67.31	68.65	69.50	69.07	6.79	9.26
El Salvador	LAC	LMIC	N. A.	62.92	56.41	59.58	64.09	56.06	59.94	66.73	60.48	63.53	69.62	59.89	64.58	67.93	60.08	63.89	7.20	6.14
Estonia	Europe	HIC	N. A.	78.56	73.65	76.06	78.32	71.55	74.86	80.22	74.49	77.30	80.06	69.31	74.49	81.58	73.22	77.28	-2.00	1.83
Eswatini	Africa	LMIC	N. A.	N. A.	N. A.	N. A.	N. A.	N. A.	N. A.	51.51	50.07	50.78	52.71	52.81	52.76	52.41	51.53	51.97	N. A.	N. A.
Ethiopia	Africa	LIC	74.5	53.50	55.44	54.46	53.23	53.45	53.34	53.17	50.06	51.59	54.66	51.02	52.81	53.68	49.69	51.64	-2.31	-3.94
Fiji	Oceania	UMIC	N. A.	N. A.	N. A.	N. A.	N. A.	N. A.	N. A.	70.07	62.41	66.13	69.95	63.30	66.54	71.24	66.53	68.84	N. A.	N. A.
Finland	Europe	HIC	N. A.	84.02	74.17	78.94	83.00	73.52	78.12	82.82	72.34	77.40	83.77	75.21	79.38	85.90	75.70	80.64	0.56	2.42
Former Yugoslav Republic of Macedonia (FYROM)	Europe	UMIC	N. A.	N. A.	N. A.	N. A.	68.95	63.61	66.23	N. A.	N. A.	N. A.	N. A.	N. A.	N. A.	N. A.	N. A.	N. A.	N. A.	N. A.
France	Europe	HIC	0.78	80.32	74.78	77.50	81.22	73.79	77.41	81.49	73.45	77.37	81.13	75.29	78.16	81.67	75.47	78.51	0.94	1.45
Gabon	Africa	UMIC	N. A.	65.14	55.91	60.35	62.84	57.94	60.34	64.76	61.68	63.20	63.40	64.15	63.78	62.82	64.74	63.77	4.60	4.60
Gambia, The	Africa	LIC	99.64	47.82	56.56	52.01	51.58	51.84	51.71	55.00	51.71	53.33	57.86	52.55	55.14	59.26	52.06	55.55	4.26	4.91
Georgia	Asia	LMIC	13.77	68.58	60.42	64.37	70.65	62.74	66.58	68.91	62.69	65.73	71.88	65.74	68.74	72.23	65.52	68.79	6.10	6.19
Germany	Europe	HIC	0.19	81.68	71.83	76.60	82.28	69.58	75.67	81.07	68.22	74.37	80.77	73.20	76.89	82.48	74.53	78.41	0.33	2.48
Ghana	Africa	LMIC	41.42	59.90	58.54	59.21	62.81	61.04	61.92	63.80	59.72	61.73	65.37	63.26	64.31	62.49	60.59	61.54	7.21	3.29
Greece	Europe	HIC	52.01	72.89	69.87	71.37	70.64	69.00	69.81	71.41	67.12	69.23	74.33	72.06	73.19	75.41	74.74	75.07	2.57	5.22
Guatemala	LAC	UMIC	3.84	58.32	55.96	57.13	58.24	56.38	57.30	59.65	54.64	57.09	61.54	56.65	59.04	59.91	53.95	56.85	2.76	-0.30
Guinea	Africa	LIC	10.94	48.78	50.30	49.53	52.12	51.20	51.66	52.81	50.89	51.84	52.47	50.53	51.49	50.96	49.09	50.02	2.77	0.69
Guyana	LAC	UMIC	0.17	64.66	57.90	61.19	61.90	55.05	58.37	62.93	58.27	60.56	61.06	56.81	58.90	59.77	58.81	59.28	-3.31	-2.82
Haiti	LAC	LIC	13.05	44.08	45.18	44.63	49.16	47.76	48.46	48.44	48.41	48.42	51.69	49.63	50.65	51.35	49.94	50.64	8.53	8.51
Honduras	LAC	LMIC	4	61.65	57.81	59.70	63.64	57.54	60.51	63.41	55.63	59.39	64.44	56.57	60.38	62.77	55.71	59.13	1.10	-0.70
Hungary	Europe	HIC	59.54	78.00	69.94	73.86	74.96	70.64	72.77	78.48	71.84	75.09	78.46	66.80	72.40	79.74	72.30	75.93	-1.89	2.90
Iceland	Europe	HIC	N. A.	79.29	65.60	72.12	79.75	64.97	71.98	79.20	67.21	72.96	77.52	63.85	70.35	78.17	64.16	70.82	-2.49	-1.81
India	Asia	LMIC	57.41	58.07	61.89	59.95	59.05	60.00	59.52	61.08	60.58	60.83	61.92	60.65	61.28	60.07	58.84	59.45	1.85	-0.74
Indonesia	Asia	LMIC	0.2	62.88	59.08	60.95	62.84	58.62	60.69	64.19	63.66	63.93	65.30	64.84	65.07	66.34	67.87	67.10	5.78	8.66
Iran, Islamic Rep.	Asia	UMIC	99.17	64.70	57.69	61.09	65.54	60.34	62.89	70.49	66.67	68.55	71.81	68.62	70.20	70.01	68.37	69.19	12.76	11.31
Iraq	Asia	UMIC	98.87	56.63	50.79	53.63	53.75	49.51	51.58	60.79	59.54	60.17	63.14	64.25	63.69	63.82	62.57	63.19	14.12	13.41
Ireland	Europe	HIC	N. A.	77.92	69.91	73.80	77.47	64.42	70.64	78.22	65.39	71.52	79.38	68.91	73.96	80.96	70.29	75.44	0.33	2.43
Israel	Asia	HIC	97.93	70.14	63.47	66.72	71.85	65.42	68.56	71.53	58.91	64.91	74.60	70.54	72.54	75.04	63.64	69.10	8.15	3.58
Italy	Europe	HIC	19.17	75.50	70.23	72.82	74.21	67.73	70.90	75.79	68.07	71.83	77.01	71.96	74.44	78.76	73.51	76.09	2.29	4.62
Jamaica	LAC	UMIC	1.47	66.57	59.38	62.87	65.90	59.42	62.57	68.80	62.39	65.51	68.66	68.30	68.48	68.97	68.49	68.73	7.79	8.14
Japan	Asia	HIC	N. A.	80.18	73.66	76.85	78.52	74.53	76.50	78.92	73.90	76.37	79.17	72.79	75.91	79.85	72.63	76.15	-1.33	-0.96
Jordan	Asia	UMIC	99.99	65.96	61.70	63.79	64.36	61.09	62.70	68.09	62.86	65.42	68.05	68.97	68.51	70.14	68.32	69.22	6.62	7.64
Kazakhstan	Asia	UMIC	97.84	71.09	65.13	68.05	68.13	64.51	66.30	68.71	62.66	65.61	71.82	65.62	68.65	72.44	66.99	69.66	0.86	2.27
Kenya	Africa	LMIC	89.86	54.93	61.82	58.27	56.83	64.83	60.70	57.03	55.24	56.13	60.17	57.84	58.99	60.60	59.25	59.92	0.89	2.19
Korea, Rep.	Asia	HIC	0.04	75.48	71.24	73.33	77.41	71.32	74.30	78.33	73.50	75.88	78.34	74.18	76.23	78.59	74.16	76.34	4.11	4.27
Kuwait	Asia	HIC	99.04	62.40	60.05	61.21	61.14	58.32	59.71	61.08	56.70	58.85	61.13	62.89	62.01	60.32	62.94	61.61	1.11	0.57
Kyrgyz Republic	Asia	LMIC	70.38	70.67	56.84	63.38	70.33	56.86	63.24	73.55	63.06	68.10	73.89	63.90	68.72	74.95	62.63	68.51	7.27	7.12
Lao PDR	Asia	LMIC	N. A.	61.37	56.90	59.09	60.63	56.14	58.34	63.01	54.97	58.85	62.43	54.96	58.58	63.16	56.48	59.73	-0.62	0.97
Latvia	Europe	HIC	N. A.	75.23	67.88	71.46	74.75	69.69	72.18	77.13	71.89	74.47	77.73	66.28	71.78	79.15	70.46	74.68	0.64	4.60
Lebanon	Asia	UMIC	34.6	64.93	64.20	64.56	64.79	62.34	63.55	65.67	62.44	64.03	66.68	64.20	65.43	66.84	64.64	65.73	1.24	1.66
Lesotho	Africa	LMIC	64.3	52.97	53.50	53.23	51.51	48.42	49.94	50.72	48.50	49.60	53.35	52.80	53.07	53.75	51.34	52.53	-0.23	-0.98
Liberia	Africa	LIC	N. A.	42.82	49.52	46.05	48.30	52.61	50.41	48.18	50.87	49.51	47.12	49.18	48.14	48.65	49.42	49.03	2.80	4.05
Lithuania	Europe	HIC	N. A.	73.63	68.22	70.87	72.90	69.98	71.42	75.10	69.62	72.31	74.95	68.28	71.54	76.70	72.34	74.49	0.97	5.08
Luxembourg	Europe	HIC	N. A.	75.00	64.88	69.75	76.09	65.20	70.43	76.25	61.23	68.33	75.24	59.92	67.15	74.89	60.87	67.52	-3.33	-2.91
Madagascar	Africa	LIC	28.33	43.50	47.69	45.54	45.59	49.37	47.44	46.70	47.71	47.20	49.14	47.77	48.45	49.01	47.78	48.39	4.05	3.96
Malawi	Africa	LIC	51.65	48.02	52.04	49.99	49.97	51.97	50.96	51.19	49.54	50.36	51.44	49.44	50.43	50.33	48.92	49.62	0.58	-0.58
Malaysia	Asia	UMIC	N. A.	69.68	63.37	66.45	70.01	66.98	68.48	69.56	66.95	68.24	71.76	65.46	68.54	70.88	65.36	68.07	2.95	2.26
Maldives	Asia	UMIC	N. A.	N. A.	N. A.	N. A.	N. A.	N. A.	N. A.	72.12	66.91	69.47	67.59	56.84	61.98	69.27	63.95	66.56	N. A.	N. A.
Mali	Africa	LIC	99.77	48.54	54.21	51.30	49.72	51.89	50.79	49.95	52.74	51.32	50.57	52.05	51.30	51.17	51.73	51.45	-0.09	0.11
Malta	Europe	HIC	87.33	77.02	68.09	72.42	74.20	67.74	70.89	76.11	69.27	72.61	75.97	68.91	72.35	75.75	69.00	72.29	-0.17	-0.26
Mauritania	Africa	LMIC	99.94	51.14	49.35	50.24	51.57	50.99	51.28	53.33	53.10	53.21	57.72	56.58	57.15	55.51	54.31	54.90	9.76	6.59
Mauritius	Africa	UMIC	N. A.	62.12	53.94	57.89	64.50	59.19	61.79	63.59	58.89	61.19	63.77	59.85	61.78	66.71	60.47	63.51	5.34	7.86
Mexico	LAC	UMIC	76.8	69.13	64.45	66.75	65.21	55.78	60.31	68.51	58.78	63.46	70.44	58.73	64.32	69.13	55.03	61.68	-3.12	-6.66

Moldova	Europe	LMIC	47.67	74.22	60.44	66.98	74.51	61.48	67.68	76.52	62.05	68.90	75.41	61.29	67.99	74.60	61.57	67.78	1.45	1.07
Mongolia	Asia	LMIC	97.28	64.18	55.66	59.77	63.86	56.11	59.86	65.84	58.10	61.85	64.47	58.73	61.54	63.98	59.54	61.72	2.38	2.60
Montenegro	Europe	UMIC	N. A.	67.29	61.56	64.36	67.63	61.26	64.37	67.25	61.62	64.38	70.19	63.17	66.59	68.21	65.87	67.03	3.19	3.69
Morocco	Africa	LMIC	97.98	66.66	60.06	63.28	66.27	61.09	63.63	69.07	63.75	66.36	71.29	67.02	69.12	70.53	68.51	69.51	8.19	8.70
Mozambique	Africa	LIC	67.23	49.24	51.97	50.59	50.66	53.93	52.27	53.03	51.20	52.11	54.13	52.32	53.21	51.05	50.47	50.76	3.70	0.22
Myanmar	Asia	LMIC	11.1	59.54	53.53	56.45	59.03	55.46	57.22	62.18	57.03	59.55	64.58	57.23	60.79	64.95	57.45	61.09	6.18	6.60
Namibia	Africa	UMIC	99.94	59.27	52.30	55.68	58.93	51.76	55.23	59.87	53.13	56.40	61.63	54.13	57.76	61.77	54.06	57.79	2.97	3.01
Nepal	Asia	LIC	6.07	61.57	54.24	57.79	62.75	54.83	58.66	65.03	56.77	60.76	66.54	58.70	62.50	66.88	60.24	63.48	6.67	8.01
Netherlands	Europe	HIC	N. A.	79.94	72.13	75.94	79.47	67.87	73.44	80.38	69.14	74.55	80.37	71.76	75.95	81.56	72.58	76.94	0.04	1.46
New Zealand	Oceania	HIC	1.61	77.62	63.64	70.28	77.86	65.10	71.19	79.80	71.31	75.44	78.25	69.30	73.64	79.13	69.90	74.37	4.46	5.50
Nicaragua	LAC	LMIC	3.83	63.11	55.41	59.14	66.38	56.67	61.34	67.94	56.84	62.14	68.66	56.20	62.11	66.29	56.47	61.18	4.48	2.99
Niger	Africa	LIC	99.99	44.80	56.02	50.09	48.51	54.00	51.18	49.13	53.29	51.17	49.26	51.66	50.44	48.37	50.00	49.18	0.07	-1.72
Nigeria	Africa	LMIC	59.65	48.65	51.71	50.16	47.48	50.02	48.73	46.41	50.01	48.17	49.28	52.42	50.83	48.93	53.77	51.29	0.95	1.65
North Macedonia	Europe	UMIC	N. A.	N. A.	N. A.	N. A.	N. A.	N. A.	N. A.	71.18	65.80	68.43	72.18	67.15	69.62	73.38	66.28	69.74	N. A.	N. A.
Norway	Europe	HIC	N. A.	83.94	68.33	75.73	81.17	66.85	73.66	80.66	65.21	72.52	80.76	62.27	70.92	81.98	62.27	71.45	-6.53	-5.67
Oman	Asia	HIC	99.88	64.31	60.97	62.62	63.91	59.25	61.53	66.00	64.79	65.39	68.53	67.46	67.99	68.92	65.12	66.99	7.57	6.19
Pakistan	Asia	LMIC	93.77	55.63	54.62	55.12	54.89	54.23	54.56	55.57	52.18	53.85	56.17	55.58	55.87	57.72	56.53	57.12	1.06	2.83
Panama	LAC	HIC	0.29	63.94	61.93	62.93	64.89	59.73	62.26	66.31	62.52	64.39	69.19	63.84	66.46	67.98	57.97	62.78	5.06	0.05
Papua New Guinea	Oceania	LMIC	N. A.	N. A.	N. A.	N. A.	N. A.	N. A.	N. A.	51.62	49.93	50.77	51.66	49.48	50.56	51.33	49.46	50.38	N. A.	N. A.
Paraguay	LAC	UMIC	53.32	66.14	58.12	62.00	67.21	57.86	62.36	68.38	60.88	64.52	68.04	62.38	65.15	67.20	58.86	62.89	4.36	1.28
Peru	LAC	UMIC	27.79	65.97	58.11	61.92	68.45	61.24	64.74	71.19	65.28	68.17	71.75	66.36	69.00	71.09	62.57	66.69	9.92	6.77
Philippines	Asia	LMIC	N. A.	64.33	59.99	62.12	65.03	60.35	62.65	64.94	63.55	64.24	65.50	63.59	64.54	64.51	58.40	61.38	3.37	-1.00
Poland	Europe	HIC	N. A.	75.78	63.44	69.34	73.67	68.28	70.93	75.93	68.54	72.14	78.10	73.49	75.76	80.22	77.31	78.75	8.74	12.95
Portugal	Europe	HIC	39.28	75.57	71.44	73.47	74.03	72.53	73.27	76.43	73.16	74.78	77.65	75.27	76.45	78.64	68.60	73.45	4.18	0.17
Qatar	Asia	HIC	97.36	63.10	63.59	63.34	60.85	59.04	59.94	64.21	66.59	65.39	62.84	58.44	60.60	65.06	57.63	61.23	-3.83	-2.83
Romania	Europe	UMIC	43.51	74.13	64.11	68.94	71.22	63.78	67.40	72.73	62.61	67.48	74.78	69.97	72.33	74.97	71.94	73.44	4.60	6.13
Russian Federation	Europe	UMIC	20.59	68.93	61.29	65.00	68.90	63.94	66.38	70.94	67.09	68.99	71.92	69.94	70.92	73.75	71.15	72.44	8.23	10.38
Rwanda	Africa	LIC	41.27	55.01	55.97	55.49	56.09	54.15	55.11	56.12	54.02	55.06	56.07	52.96	54.49	56.92	53.97	55.43	-1.38	-0.05
Sao Tome and Principe	Africa	LMIC	N. A.	N. A.	N. A.	N. A.	N. A.	N. A.	N. A.	65.48	57.98	61.62	62.57	56.08	59.24	58.79	56.50	57.63	N. A.	N. A.
Saudi Arabia	Asia	HIC	99.94	62.68	51.30	56.70	62.92	63.17	63.04	62.58	63.75	63.16	64.19	65.10	64.64	64.58	64.52	64.55	10.83	10.69
Senegal	Africa	LIC	98.52	56.24	57.68	56.95	57.17	56.96	57.07	57.30	53.66	55.45	58.27	55.47	56.85	58.43	56.18	57.30	-0.12	0.49
Serbia	Europe	UMIC	25.43	73.58	64.89	69.09	72.14	64.52	68.22	74.47	67.24	70.77	76.25	70.25	73.19	76.64	71.28	73.91	5.69	6.68
Sierra Leone	Africa	LIC	N. A.	47.11	52.13	49.56	49.11	50.19	49.65	49.24	49.39	49.32	51.91	50.44	51.17	51.69	49.39	50.53	2.20	1.30
Singapore	Asia	HIC	N. A.	68.97	62.80	65.82	71.31	59.20	64.97	70.07	60.31	65.01	67.69	57.60	62.44	70.83	59.66	65.01	-4.58	-0.91
Slovak Republic	Europe	HIC	N. A.	76.93	70.49	73.64	75.60	71.43	73.48	77.77	71.47	74.55	78.65	71.14	74.80	80.58	74.76	77.62	1.67	5.60
Slovenia	Europe	HIC	N. A.	80.54	74.42	77.42	79.98	72.02	75.89	79.41	65.36	72.04	79.80	70.66	75.09	81.60	76.32	78.92	-3.18	2.10
Somalia	Africa	LIC	99.93	N. A.	N. A.	N. A.	N. A.	N. A.	N. A.	N. A.	N. A.	N. A.	45.94	47.45	46.69	45.11	47.65	46.36	N. A.	N. A.
South Africa	Africa	UMIC	92.45	61.25	53.82	57.41	60.83	53.87	57.24	61.48	52.53	56.83	63.41	54.76	58.93	63.74	54.38	58.87	2.20	2.16
South Sudan	Africa	LIC	87.22	N. A.	N. A.	N. A.	N. A.	N. A.	N. A.	N. A.	N. A.	N. A.	42.36	50.14	46.09	37.08	45.38	41.02	N. A.	N. A.
Spain	Europe	HIC	72.26	76.75	69.88	73.24	75.42	70.27	72.80	77.84	74.21	76.01	78.11	73.30	75.67	79.46	73.23	76.28	3.38	4.29
Sri Lanka	Asia	LMIC	N. A.	65.91	56.38	60.96	64.56	56.65	60.48	65.84	57.20	61.37	66.88	63.68	65.26	68.10	64.05	66.04	5.84	6.97
Sudan	Africa	LMIC	99.99	49.87	58.77	54.14	49.58	56.09	52.73	51.36	51.50	51.43	49.56	49.94	49.75	49.48	50.61	50.04	-6.46	-6.05
Suriname	LAC	UMIC	N. A.	70.35	66.38	68.34	67.97	63.63	65.77	68.90	62.66	65.71	70.22	61.54	65.74	69.42	66.29	67.84	-3.51	-0.72
Swaziland	Africa	LMIC	65.47	54.98	56.57	55.77	50.67	48.50	49.57	N. A.	N. A.	N. A.	N. A.	N. A.	N. A.	N. A.	N. A.	N. A.	N. A.	N. A.
Sweden	Europe	HIC	N. A.	85.61	76.66	81.01	84.98	75.12	79.90	84.99	70.24	77.26	84.72	74.98	79.70	85.61	76.50	80.93	-1.81	-0.10
Switzerland	Europe	HIC	N. A.	81.18	67.59	74.08	80.09	67.45	73.50	80.55	64.24	71.93	80.60	68.78	74.46	81.14	68.65	74.63	0.43	0.72
Syrian Arab Republic	Asia	LIC	96.57	58.14	58.62	58.38	55.02	54.79	54.91	55.91	53.28	54.58	57.16	55.65	56.40	55.86	54.00	54.93	-2.80	-4.88
Tajikistan	Asia	LIC	48.32	66.77	58.91	62.72	67.18	56.02	61.35	71.01	56.53	63.36	70.09	56.71	63.04	70.44	56.21	62.92	0.79	0.68
Tanzania	Africa	LIC	66.76	52.08	53.91	52.98	55.15	55.06	55.11	55.82	53.29	54.54	56.64	55.14	55.89	56.43	53.77	55.08	4.10	2.98
Thailand	Asia	UMIC	12.42	69.54	64.43	66.93	69.24	65.49	67.34	73.00	66.67	69.76	74.54	71.39	72.95	74.19	72.04	73.11	8.46	8.68
Timor-Leste	Asia	LMIC	N. A.	61.51	57.96	59.71	N. A.	N. A.	N. A.	N. A.	N. A.	N. A.	N. A.	N. A.	N. A.	N. A.	N. A.	N. A.	N. A.	N. A.
Togo	Africa	LIC	53.33	50.18	53.03	51.59	52.00	52.14	52.07	51.60	53.24	52.41	52.70	51.94	52.32	53.23	52.67	52.95	1.01	1.90
Trinidad and Tobago	LAC	HIC	N. A.	69.11	62.29	65.61	67.51	61.04	64.20	69.46	61.70	65.46	67.47	60.18	63.72	65.72	60.85	63.24	-2.66	-3.41
Tunisia	Africa	LMIC	98.51	68.66	60.47	64.44	66.15	61.01	63.53	69.99	62.35	66.06	71.37	67.08	69.19	71.44	67.88	69.64	6.59	7.21
Turkey	Asia	UMIC	71.27	68.48	66.39	67.43	65.96	62.48	64.19	68.49	60.90	64.58	70.30	65.59	67.91	70.38	64.81	67.54	0.72	0.22

Turkmenistan	Asia	UMIC	99.99	56.73	49.21	52.83	59.47	48.61	53.77	64.02	54.09	58.85	62.87	56.30	59.50	60.54	56.63	58.55	9.36	7.94
Uganda	Africa	LIC	38.9	52.86	50.84	51.84	54.93	49.86	52.33	52.45	50.46	51.45	52.80	48.76	50.74	52.55	52.14	52.34	-1.51	0.70
Ukraine	Europe	LMIC	31.76	72.73	61.15	66.69	72.34	61.24	66.56	72.81	61.29	66.81	74.24	64.16	69.02	75.51	65.95	70.57	3.19	5.35
United Arab Emirates	Asia	HIC	98.73	66.01	62.13	64.04	69.22	63.07	66.07	68.22	62.61	65.35	68.80	63.76	66.23	70.17	61.92	65.92	3.13	2.80
United Kingdom	Europe	HIC	N. A.	78.28	66.74	72.28	78.67	68.68	73.50	79.38	69.26	74.15	79.79	73.03	76.34	79.97	73.60	76.72	5.52	6.05
United States	North America	HIC	45.08	72.40	67.10	69.70	73.05	65.96	69.42	74.52	65.38	69.80	76.43	70.49	73.40	76.01	70.44	73.17	5.24	4.91
Uruguay	LAC	HIC	0.11	71.05	64.99	67.95	70.42	62.49	66.34	72.55	65.74	69.06	74.28	68.24	71.20	74.55	68.37	71.39	4.58	4.86
Uzbekistan	Asia	LMIC	97.28	71.25	57.62	64.07	70.29	60.80	65.37	73.03	62.40	67.50	71.77	63.02	67.25	70.52	61.35	65.78	4.19	2.13
Vanuatu	Oceania	LMIC	N. A.	N. A.	N. A.	N. A.	N. A.	N. A.	N. A.	59.87	61.48	60.67	60.89	56.80	58.81	60.52	60.26	60.39	N. A.	N. A.
Venezuela, RB	LAC	UMIC	15.53	65.83	63.28	64.55	64.00	60.33	62.14	63.05	62.11	62.58	61.68	60.68	61.18	59.32	56.63	57.96	-4.77	-9.31
Vietnam	Asia	LMIC	0.46	67.89	58.27	62.90	69.67	59.18	64.21	71.09	62.07	66.42	73.80	69.63	71.68	72.85	69.05	70.92	12.21	11.13
Yemen, Rep.	Asia	LIC	99.9	49.80	48.41	49.10	45.66	47.58	46.61	51.21	50.99	51.10	49.71	50.09	49.90	50.40	49.62	50.01	1.12	1.28
Zambia	Africa	LMIC	65.35	51.08	57.78	54.32	53.13	55.30	54.20	52.50	52.24	52.37	51.94	48.42	50.15	52.48	48.59	50.50	-6.01	-5.51
Zimbabwe	Africa	LIC	97.53	56.11	59.10	57.59	58.76	57.43	58.09	61.82	54.32	57.95	60.98	55.82	58.34	59.93	55.91	57.89	1.12	0.44

Table S3. Statistics details for Fig. 1 and Fig. S3.

A. Statistics for the ANOVA test of the evenness scores across different regions in 2021.

ANOVA	F	df	*p*	LSD						Average
				p	Asia	LAC	Oceania	Europe	North A.	
Geographic location (N=165)	43.28	5	<0.001	Africa (N=48)	<0.001	<0.001	0.008	<0.001	<0.001	53.76
				Asia (N=45)	1	0.070	0.523	<0.001	0.036	62.38
				LAC (N=26)		1	0.764	<0.001	0.008	59.91
				Oceania (N=5)			1	<0.001	0.030	60.72
				Europe (N=39)				1	0.998	70.78
				North A. (N=2)					1	70.77
UN income groups (N=165)	71.63	3	<0.001	*p*	UMIC	LMIC	LIC			
				HIC (N=50)	<0.001	<0.001	<0.001			68.74
				UMIC (N=44)	1	<0.001	<0.001			63.12
				LMIC (N=41)		1	<0.001			58.81
				LIC (N=30)			1			50.83
Aridity (N=115)	2.03	3	0.114	*p*	Moderately	Severely	Extremely			
				Slightly (N=50)	0.183	0.308	0.020			61.95
				Moderately (N=19)	1	0.709	0.412			59.07
				Severely (N=26)		1	0.208			59.97
				Extremely (N=20)			1			56.97

B. Statistics for the ANOVA test of the mean index score across different regions in 2021.

ANOVA	F	df	*p*	LSD						Average
				p	Asia	LAC	Oceania	Europe	North A.	
Geographic location (N=165)	64.18	5	<0.001	Africa (N=48)	<0.001	<0.001	<0.001	<0.001	<0.001	55.05
				Asia (N=45)	1	0.737	0.77	<0.001	0.016	67.52
				LAC (N=26)		1	0.91	<0.001	0.024	68.16
				Oceania (N=5)			1	<0.001	0.055	67.56
				Europe (N=39)				1	0.602	78.74
				North A. (N=2)					1	77.58
UN income groups (N=165)	93.21	3	<0.001	*p*	UMIC	LMIC	LIC			
				HIC (N=50)	<0.001	<0.001	<0.001			76.70
				UMIC (N=44)	1	<0.001	<0.001			69.48
				LMIC (N=41)		1	<0.001			62.63
				LIC (N=30)			1			51.88
Aridity (N=115)	5.04	3	0.003	*p*	Moderately	Severely	Extremely			
				Slightly (N=50)	0.068	0.149	<0.001			67.89
				Moderately (N=19)	1	0.632	0.114			62.94
				Severely (N=26)		1	0.03			64.98
				Extremely (N=20)			1			58.11

C. Statistics for the ANOVA test of the sustainable development score across different regions in 2021.

ANOVA	F	df	*p*	LSD						Average
				p	Asia	LAC	Oceania	Europe	North A.	
Geographic location (N=165)	60.86	5	<0.001	Africa (N=48)	<0.001	<0.001	0.001	<0.001	<0.001	54.37
				Asia (N=45)	1	0.397	0.874	<0.001	0.013	64.87
				LAC (N=26)		1	0.784	<0.001	0.006	63.86
				Oceania (N=5)			1	<0.001	0.025	63.98
				Europe (N=39)				1	0.702	74.62
				North A. (N=2)					1	74.09
UN income groups (N=165)	86.17	3	<0.001	*p*	UMIC	LMIC	LIC			
				HIC (N=50)	<0.001	<0.001	<0.001			72.57
				UMIC (N=44)	1	<0.001	<0.001			66.19
				LMIC (N=41)		1	<0.001			60.66
				LIC (N=30)			1			51.31
Aridity (N=115)	3.62	3	0.015	*p*	Moderately	Severely	Extremely			
				Slightly (N=50)	0.116	0.153	0.002			64.80
				Moderately (N=19)	1	0.795	0.191			60.95
				Severely (N=26)		1	0.096			62.37
				Extremely (N=20)			1			57.50

D1. Statistics for the ANOVA test of the effective development score across different regions from 2017 to 2020.

ANOVA	F	df	*p*	LSD test						Average
				p	Asia	LAC	Oceania	Europe	North A.	
Geographic locations (N=163)	2.49	5	0.034	Africa (N=43)	0.011	0.413	0.454	0.520	0.917	2.30
				Asia (N=43)	1	0.156	0.130	0.002	0.505	4.54
				LAC (N=26)		1	0.310	0.174	0.861	3.12
				Oceania (N=2)			1	0.583	0.537	0.12
				Europe (N=38)				1	0.763	1.72
				North A. (N=2)					1	2.60
UN income groups (N=166)	6.65	3	<0.001	*p*	UMIC	LMIC	LIC			
				HIC (N=49)	<0.001	0.002	0.991			1.50
				UMIC (N=41)	1	0.728	0.003			4.44
				LMIC (N=36)		1	0.008			4.13
				LIC (N=28)			1			1.51
Aridity (N=115)	1.77	3	0.158	*p*	Moderately	Severely	Extremely			
				Slightly (N=50)	0.112	0.162	0.559			3.86
				Moderately (N=19)	1	0.787	0.072			2.10
				Severely (N=26)		1	0.102			2.44
				Extremely (N=20)			1			4.50

D2. Statistics for the ANOVA test of the effective development score across different regions from 2017 to 2021.

ANOVA	F	df	*p*	LSD						Average	2021 vs. 2020
				p	Asia	LAC	Oceania	Europe	North A.		
Geographic location (N=154)	2.92	5	0.015	Africa (N=43)	0.004	0.536	0.643	0.089	0.867	1.78	-0.52
				Asia (N=43)	1	0.002	0.183	0.261	0.481	4.34	-0.2
				LAC (N=26)		1	0.805	0.037	0.708	1.16	-1.96
				Oceania (N=2)			1	0.325	0.648	0.42	0.3
				Europe (N=38)				1	0.721	3.33	1.61
				North A. (N=2)					1	2.27	-0.33
UN income groups (N=154)	3.85	3	0.011	*p*	UMIC	LMIC	LIC				
				HIC (N=49)	0.12	0.083	0.129			2.30	0.8
				UMIC (N=41)	1	0.829	0.005			3.66	-0.78
				LMIC (N=36)		1	0.004			3.86	-0.27
				LIC (N=28)			1			0.83	-0.68
Aridity (N=112)	1.56	3	0.204	*p*	Moderately	Severely	Extremely				
				Slightly (N=50)	0.125	0.168	0.663			3.32	-0.54
				Moderately (N=19)	1	0.815	0.103			1.55	-0.55
				Severely (N=24)		1	0.135			1.86	-0.58
				Extremely (N=19)			1			3.82	-0.68

Table S4. Spearman correlations among SDGs across different geographic locations (A), UN income groups (B), and arid levels (C) in 2021. *, p < 0.05; **, p < 0.01.

A1. Africa

r	SDG 1	SDG 2	SDG 3	SDG 4	SDG 5	SDG 6	SDG 7	SDG 8	SDG 9	SDG 10	SDG 11	SDG 12	SDG 13	SDG 14	SDG 15	SDG 16	SDG 17
**SDG 1**	1	0.186	0.574**	0.509**	0.047	0.617**	0.704**	0.363*	0.748**	0.284	0.498**	-0.284	-0.674**	-0.402*	0.028	0.563**	0.300*
**SDG 2**		1	0.335*	0.293*	0.124	0.225	0.187	0.497**	0.430**	-0.010	0.164	0.268	-0.144	-0.262	-0.060	0.189	0.024
**SDG 3**			1	0.633**	0.348*	0.586**	0.570**	0.408**	0.713**	0.066	0.606**	-.376**	-0.531**	-0.341	-0.078	0.779**	0.386**
**SDG 4**				1	0.542**	0.497**	0.630**	0.204	0.673**	-0.173	0.622**	-.302*	-0.645**	-0.265	-0.017	0.568**	0.505**
**SDG 5**					1	0.190	0.236	0.087	0.321*	-0.465**	0.519**	-0.205	-0.332*	0.109	-0.208	0.187	0.448**
**SDG 6**						1	0.551**	0.267	0.679**	-0.073	0.609**	-.318*	-0.680**	-0.284	0.138	0.615**	0.447**
**SDG 7**							1	0.110	0.694**	-0.078	0.527**	-.305*	-0.801**	-0.340	0.040	0.497**	0.272
**SDG 8**								1	0.386**	0.199	0.301*	0.142	-0.124	-0.119	-0.056	0.451**	0.096
**SDG 9**									1	-0.089	0.552**	-.326*	-0.742**	-0.388*	0.050	0.552**	0.395**
**SDG 10**										1	-0.100	0.144	0.283	-0.305	-0.161	0.065	-0.190
**SDG 11**											1	-.382**	-0.614**	-0.244	-0.086	0.625**	0.586**
**SDG 12**												1	0.368*	0.102	-0.132	-0.352*	-0.203
**SDG 13**													1	0.248	-0.132	-0.506**	-0.542**
**SDG 14**														1	0.169	-0.395*	-0.280
**SDG 15**															1	-0.080	0.048
**SDG 16**																1	0.471**
**SDG 17**																	1

A2. Asia

r	SDG 1	SDG 2	SDG 3	SDG 4	SDG 5	SDG 6	SDG 7	SDG 8	SDG 9	SDG 10	SDG 11	SDG 12	SDG 13	SDG 14	SDG 15	SDG 16	SDG 17
**SDG 1**	1	0.061	0.772**	0.369*	0.139	0.450**	0.607**	0.359*	0.686**	0.139	0.455**	-0.740**	-0.596**	-0.138	0.032	0.447**	0.270
**SDG 2**		1	0.436**	0.325*	0.497**	0.043	0.224	0.230	0.494**	0.072	0.214	-0.101	-0.203	-0.276	-0.197	0.330*	-0.072
**SDG 3**			1	0.669**	0.308*	0.329*	0.803**	0.505**	0.836**	0.109	0.467**	-0.825**	-0.767**	-0.370*	0.008	0.659**	0.185
**SDG 4**				1	0.481**	0.376*	0.464**	0.631**	0.557**	0.117	0.539**	-0.568**	-0.508**	-0.255	0.250	0.492**	0.310*
**SDG 5**					1	0.442**	0.066	0.434**	0.362*	0.230	0.485**	-0.144	-0.237	-0.324	0.134	0.320*	0.004
**SDG 6**						1	0.267	0.497**	0.296*	0.032	0.483**	-0.187	-0.194	-0.324	0.062	0.213	0.323*
**SDG 7**							1	0.291	0.586**	0.134	0.436**	-0.684**	-0.576**	-0.311	-0.010	0.677**	0.280
**SDG 8**								1	0.496**	0.010	0.383**	-0.405**	-0.360*	-0.263	0.012	0.388**	0.024
**SDG 9**									1	0.010	0.351*	-0.678**	-0.667**	-0.228	-0.088	0.526**	0.166
**SDG 10**										1	0.057	-0.161	-0.173	-0.189	0.368*	0.190	0.002
**SDG 11**											1	-0.327*	-0.265	-0.143	0.092	0.439**	0.387**
**SDG 12**												1	0.890**	0.261	-0.104	-0.603**	-0.203
**SDG 13**													1	0.210	-0.086	-0.560**	-0.152
**SDG 14**														1	0.297	-0.093	-0.087
**SDG 15**															1	0.151	0.238
**SDG 16**																1	0.203
**SDG 17**																	1

A3. Latin America and Caribbean

r	SDG 1	SDG 2	SDG 3	SDG 4	SDG 5	SDG 6	SDG 7	SDG 8	SDG 9	SDG 10	SDG 11	SDG 12	SDG 13	SDG 14	SDG 15	SDG 16	SDG 17
**SDG 1**	1	0.410*	0.632**	0.605**	0.447*	0.355	0.408*	0.531**	0.528**	0.099	0.503*	-0.417*	-0.433*	-0.169	-0.045	0.434*	0.244
**SDG 2**		1	0.134	0.359	0.185	0.452*	0.205	0.190	0.461*	-0.009	0.136	-0.381	-0.180	0.146	-0.108	0.233	0.300
**SDG 3**			1	0.571**	0.525**	0.504**	0.490*	0.467*	0.565**	0.042	0.341	-0.235	-0.376	-0.137	0.019	0.666**	0.610**
**SDG 4**				1	0.573**	0.339	0.357	0.301	0.541**	-0.080	0.051	-0.208	-0.339	0.060	0.041	0.361	0.457*
**SDG 5**					1	0.175	0.192	0.279	0.383	0.206	0.241	0.026	-0.033	-0.123	0.322	0.351	0.536**
**SDG 6**						1	0.529**	0.184	0.763**	-0.394	0.282	-0.095	-0.142	0.067	-0.071	0.229	0.439*
**SDG 7**							1	0.261	0.652**	-0.098	0.394*	-0.602**	-0.524**	0.034	0.229	0.545**	0.515**
**SDG 8**								1	0.444*	0.116	0.168	-0.311	-0.493*	0.162	0.158	0.322	0.252
**SDG 9**									1	-0.317	0.405*	-0.346	-0.400*	0.094	0.024	0.516**	0.486*
**SDG 10**										1	-0.065	0.038	0.128	-0.112	0.195	0.059	0.008
**SDG 11**											1	-0.256	-0.276	0.096	0.186	0.520**	0.174
**SDG 12**												1	0.802**	0.297	0.128	-0.535**	-0.227
**SDG 13**													1	0.175	-0.052	-0.594**	-0.194
**SDG 14**														1	0.311	-0.029	0.043
**SDG 15**															1	0.106	0.188
**SDG 16**																1	0.581**

A4. Oceania

r	SDG 1	SDG 2	SDG 3	SDG 4	SDG 5	SDG 6	SDG 7	SDG 8	SDG 9	SDG 10	SDG 11	SDG 12	SDG 13	SDG 14	SDG 15	SDG 16	SDG 17
**SDG 1**	1	1.000**	0.900*	0.900*	0.900*	0.900*	1.000**	1.000**	0.900*	0.800	0.600	-0.800	-0.800	-0.600	-0.500	0.900*	0.900*
**SDG 2**		1	0.900*	0.900*	0.900*	0.900*	1.000**	1.000**	0.900*	0.800	0.600	-0.800	-0.800	-0.600	-0.500	0.900*	0.900*
**SDG 3**			1	1.000**	0.800	1.000**	0.900*	0.900*	1.000**	0.900*	0.700	-0.900*	-0.900*	-0.500	-0.300	0.700	0.800
**SDG 4**				1	0.800	1.000**	0.900*	0.900*	1.000**	0.900*	0.700	-0.900*	-0.900*	-0.500	-0.300	0.700	0.800
**SDG 5**					1	0.800	0.900*	0.900*	0.800	0.600	0.500	-0.600	-0.900*	-0.700	-0.300	0.800	0.700
**SDG 6**						1	0.900*	0.900*	1.000**	0.900*	0.700	-0.900*	-0.900*	-0.500	-0.300	0.700	0.800
**SDG 7**							1	1.000**	0.900*	0.800	0.600	-0.800	-0.800	-0.600	-0.500	0.900*	0.900*
**SDG 8**								1	0.900*	0.800	0.600	-0.800	-0.800	-0.600	-0.500	0.900*	0.900*
**SDG 9**									1	0.900*	0.700	-0.900*	-0.900*	-0.500	-0.300	0.700	0.800
**SDG 10**										1	0.400	-1.000**	-0.700	-0.600	-0.100	0.500	0.900*
**SDG 11**											1	-0.400	-0.600	0.200	-0.700	0.700	0.300
**SDG 12**												1	0.700	0.600	0.100	-0.500	-0.900*
**SDG 13**													1	0.600	0.100	-0.600	-0.600
**SDG 14**														1	-0.300	-0.300	-0.700
**SDG 15**															1	-0.800	-0.300
**SDG 16**																1	0.700
**SDG 17**																	1

A5. Europe

r	SDG 1	SDG 2	SDG 3	SDG 4	SDG 5	SDG 6	SDG 7	SDG 8	SDG 9	SDG 10	SDG 11	SDG 12	SDG 13	SDG 14	SDG 15	SDG 16	SDG 17
**SDG 1**	1	-0.109	0.254	0.459**	0.346*	0.185	0.284	0.353*	0.297	0.563**	0.362*	-0.241	-0.415**	0.317	-0.100	0.239	0.050
**SDG 2**		1	0.065	0.003	-0.025	0.246	0.109	0.197	0.128	-0.123	-0.032	-0.028	-0.029	0.139	0.220	-0.031	-0.312
**SDG 3**			1	0.612**	0.801**	0.524**	0.729**	0.558**	0.904**	0.471**	0.840**	-0.845**	-0.828**	0.170	0.012	0.805**	-0.024
**SDG 4**				1	0.574**	0.353*	0.540**	0.578**	0.625**	0.316*	0.559**	-0.633**	-0.680**	0.135	0.054	0.480**	-0.072
**SDG 5**					1	0.531**	0.735**	0.554**	0.820**	0.505**	0.752**	-0.612**	-0.655**	0.269	0.006	0.717**	0.157
**SDG 6**						1	0.441**	0.568**	0.607**	0.268	0.602**	-0.405*	-0.467**	0.474**	0.429**	0.561**	-0.322*
**SDG 7**							1	0.554**	0.728**	0.526**	0.583**	-0.606**	-0.575**	-0.087	-0.079	0.527**	0.011
**SDG 8**								1	0.644**	0.503**	0.616**	-0.574**	-0.654**	0.413*	0.490**	0.653**	-0.156
**SDG 9**									1	0.426**	0.870**	-0.806**	-0.839**	0.209	0.040	0.782**	0.018
**SDG 10**										1	0.386*	-0.352*	-0.455**	0.234	-0.014	0.439**	0.272
**SDG 11**											1	-0.755**	-0.835**	0.283	0.135	0.756**	-0.164
**SDG 12**												1	0.900**	-0.166	0.003	-0.779**	0.071
**SDG 13**													1	-0.338	-0.106	-0.814**	0.078
**SDG 14**														1	0.471**	0.418*	0.003
**SDG 15**															1	0.198	-0.487**
**SDG 16**																1	-0.058
**SDG 17**																	1

B1. High-income countries

HIC	SDG 1	SDG 2	SDG 3	SDG 4	SDG 5	SDG 6	SDG 7	SDG 8	SDG 9	SDG 10	SDG 11	SDG 12	SDG 13	SDG 14	SDG 15	SDG 16	SDG 17
**SDG 1**	1	-0.081	0.294	0.195	0.382*	0.331*	0.265	0.493**	0.264	0.400**	0.336*	-0.409**	-0.311*	0.253	0.070	0.424**	0.136
**SDG 2**		1	0.028	0.040	-0.096	0.095	0.061	0.113	0.197	-0.123	-0.105	0.159	0.172	-0.041	0.013	0.037	-0.102
**SDG 3**			1	0.566**	0.646**	0.347*	0.555**	0.486**	0.825**	0.478**	0.602**	-0.594**	-0.292*	-0.100	0.067	0.687**	0.287*
**SDG 4**				1	0.408**	0.285*	0.388**	0.479**	0.524**	0.349*	0.500**	-0.488**	-0.227	-0.003	0.185	0.407**	0.282*
**SDG 5**					1	0.569**	0.591**	0.539**	0.617**	0.363*	0.651**	-0.434**	0.013	0.213	0.259	0.590**	0.396**
**SDG 6**						1	0.141	0.575**	0.373**	0.087	0.476**	-0.057	0.239	0.352*	0.542**	0.430**	0.237
**SDG 7**							1	0.349*	0.435**	0.343*	0.348*	-0.454**	0.064	-0.248	-0.125	0.320*	0.362**
**SDG 8**								1	0.523**	0.566**	0.361*	-0.142	0.049	0.230	0.536**	0.576**	0.194
**SDG 9**									1	0.399**	0.588**	-0.449**	-0.271	0.046	0.083	0.616**	0.266
**SDG 10**										1	0.389*	-0.470**	-0.562**	0.184	0.373*	0.583**	0.131
**SDG 11**											1	-0.475**	-0.181	0.125	0.268	0.457**	0.299*
**SDG 12**												1	0.565**	0.126	0.079	-0.493**	-0.230
**SDG 13**													1	0.003	0.281*	-0.263	-0.026
**SDG 14**														1	0.410**	0.250	0.108
**SDG 15**															1	0.267	-0.077
**SDG 16**																1	0.264
**SDG 17**																	1

B2. Upper-middle-income countries

r	SDG 1	SDG 2	SDG 3	SDG 4	SDG 5	SDG 6	SDG 7	SDG 8	SDG 9	SDG 10	SDG 11	SDG 12	SDG 13	SDG 14	SDG 15	SDG 16	SDG 17
**SDG 1**	1	0.309*	0.599**	0.431**	-0.192	0.119	0.205	0.401**	0.489**	0.611**	0.244	-0.082	-0.152	-0.167	-0.052	0.381*	-0.011
**SDG 2**		1	0.327*	0.193	0.106	0.162	0.050	0.100	0.228	0.127	0.091	-0.053	0.048	-0.003	0.086	0.097	0.020
**SDG 3**			1	0.527**	0.079	0.452**	0.319*	0.426**	0.412**	0.256	0.095	0.018	0.034	-0.206	-0.016	0.352*	0.150
**SDG 4**				1	0.097	0.266	-0.041	0.243	0.268	0.095	0.182	0.027	0.066	-0.141	-0.165	0.167	0.160
**SDG 5**					1	0.240	-0.135	0.250	0.134	-0.527**	0.206	0.027	0.020	0.109	0.189	-0.200	0.281
**SDG 6**						1	0.251	0.377*	0.142	-0.126	0.225	-0.074	0.238	0.073	0.014	-0.030	0.164
**SDG 7**							1	0.074	0.120	0.091	0.190	0.062	0.185	0.067	0.025	0.047	0.117
**SDG 8**								1	0.192	0.060	0.438**	-0.198	-0.097	0.064	0.003	0.265	-0.062
**SDG 9**									1	0.016	0.144	-0.060	-0.235	-0.176	-0.082	0.161	0.090
**SDG 10**										1	-0.216	0.058	-0.096	-0.150	0.140	0.455**	0.017
**SDG 11**											1	-0.321*	-0.193	0.102	-0.163	0.197	-0.154
**SDG 12**												1	0.565**	0.234	-0.022	-0.127	-0.060
**SDG 13**													1	0.167	-0.116	-0.128	0.082
**SDG 14**														1	0.402*	-0.046	-0.022
**SDG 15**															1	0.025	0.170
**SDG 16**																1	0.111
**SDG 17**																	1

B3. Lower-middle-income countries

r	SDG 1	SDG 2	SDG 3	SDG 4	SDG 5	SDG 6	SDG 7	SDG 8	SDG 9	SDG 10	SDG 11	SDG 12	SDG 13	SDG 14	SDG 15	SDG 16	SDG 17
**SDG 1**	1	0.239	0.786^{**}	0.708^{**}	0.295	0.675^{**}	0.730^{**}	0.401^{**}	0.586^{**}	0.680^{**}	0.454^{**}	-0.350^{*}	-0.474^{**}	-0.613^{**}	-0.122	0.555^{**}	0.570^{**}
**SDG 2**		1	0.194	0.422^{**}	0.211	0.262	0.352^{*}	0.155	0.429^{**}	0.175	0.067	0.476^{**}	0.019	-0.258	-0.156	0.142	-0.092
**SDG 3**			1	0.720^{**}	0.366^{*}	0.653^{**}	0.778^{**}	0.432^{**}	0.489^{**}	0.411^{**}	0.675^{**}	-0.459^{**}	-0.457^{**}	-0.383^{*}	-0.142	0.600^{**}	0.587^{**}
**SDG 4**				1	0.387^{*}	0.574^{**}	0.691^{**}	0.541^{**}	0.659^{**}	0.396^{*}	0.451^{**}	-0.332^{*}	-0.610^{**}	-0.288	-0.114	0.624^{**}	0.481^{**}
**SDG 5**					1	0.505^{**}	0.337^{*}	0.248	-0.088	-0.066	0.472^{**}	-0.120	-0.393^{*}	-0.197	-0.137	0.017	0.477^{**}
**SDG 6**						1	0.648^{**}	0.323^{*}	0.386^{*}	0.333^{*}	0.476^{**}	-0.189	-0.489^{**}	-0.410^{*}	-0.093	0.339^{*}	0.506^{**}
**SDG 7**							1	0.214	0.549^{**}	0.394^{*}	0.518^{**}	-0.267	-0.567^{**}	-0.517^{**}	-0.013	0.499^{**}	0.601^{**}
**SDG 8**								1	0.443^{**}	0.197	0.426^{**}	-0.336^{*}	-0.272	-0.011	-0.260	0.353^{*}	0.154
**SDG 9**									1	0.397^{*}	0.185	-0.122	-0.307	-0.381^{*}	-0.124	0.574^{**}	0.318^{*}
**SDG 10**										1	0.153	-0.171	-0.186	-0.435^{*}	-0.241	0.422^{**}	0.178
**SDG 11**											1	-0.432^{**}	-0.578^{**}	-0.281	-0.171	0.380^{*}	0.515^{**}
**SDG 12**												1	0.552^{**}	-0.063	-0.060	-0.475^{**}	-0.573^{**}
**SDG 13**													1	0.316	-0.071	-0.324^{*}	-0.660^{**}
**SDG 14**														1	0.050	-0.234	-0.357^{*}
**SDG 15**															1	-0.017	0.213
**SDG 16**																1	0.291
**SDG 17**																	1

B4. Low-income countries

r	SDG 1	SDG 2	SDG 3	SDG 4	SDG 5	SDG 6	SDG 7	SDG 8	SDG 9	SDG 10	SDG 11	SDG 12	SDG 13	SDG 14	SDG 15	SDG 16	SDG 17
**SDG 1**	1	0.256	0.315	0.168	-0.082	0.359	0.490*	0.607**	0.415*	0.646**	0.254	0.201	-0.547**	0.072	0.049	0.343	0.169
**SDG 2**		1	0.552**	0.208	0.463**	0.301	-0.014	0.621**	0.625**	0.043	0.432*	0.265	-0.215	-0.036	0.106	0.523**	0.213
**SDG 3**			1	0.477**	0.389*	0.509**	0.283	0.515**	0.688**	0.091	0.466**	-0.076	-0.528**	-0.121	0.049	0.459*	0.429*
**SDG 4**				1	0.427*	0.310	0.350	0.131	0.435*	0.042	0.170	0.053	-0.410*	0.095	-0.179	0.194	0.261
**SDG 5**					1	0.276	0.002	0.362*	0.427*	-0.177	0.442*	0.179	-0.042	0.207	0.038	0.306	0.345
**SDG 6**						1	0.214	0.499**	0.458*	0.168	0.436*	0.013	-0.436*	-0.250	0.007	0.644**	0.350
**SDG 7**							1	0.161	0.344	0.189	0.205	0.032	-0.684**	0.039	-0.143	-0.017	-0.022
**SDG 8**								1	0.700**	0.298	0.512**	0.233	-0.449*	-0.264	0.202	0.670**	0.358
**SDG 9**									1	-0.055	0.383*	0.098	-0.605**	-0.064	0.110	0.362*	0.351
**SDG 10**										1	0.243	0.068	-0.030	0.061	-0.062	0.251	0.023
**SDG 11**											1	0.140	-0.292	-0.354	0.091	0.589**	0.297
**SDG 12**												1	-0.210	0.050	-0.235	0.139	0.283
**SDG 13**													1	0.232	0.088	-0.289	-0.384*
**SDG 14**														1	-0.143	-0.429	-0.225
**SDG 15**															1	0.149	0.117
**SDG 16**																1	0.543**
**SDG 17**																	1

C1. Slightly arid

r	SDG 1	SDG 2	SDG 3	SDG 4	SDG 5	SDG 6	SDG 7	SDG 8	SDG 9	SDG 10	SDG 11	SDG 12	SDG 13	SDG 14	SDG 15	SDG 16	SDG 17
**SDG 1**	1	0.568**	0.799**	0.746**	0.477**	0.701**	0.759**	0.725**	0.791**	0.527**	0.748**	-0.655**	-0.777**	-0.217	-0.023	0.678**	0.596**
**SDG 2**		1	0.564**	0.565**	0.368**	0.494**	0.503**	0.506**	0.679**	0.335*	0.463**	-0.413**	-0.491**	-0.227	-0.104	0.559**	0.419**
**SDG 3**			1	0.764**	0.644**	0.873**	0.860**	0.785**	0.838**	0.323*	0.796**	-0.723**	-0.831**	-0.130	0.017	0.681**	0.682**
**SDG 4**				1	0.528**	0.729**	0.761**	0.697**	0.777**	0.428**	0.685**	-0.537**	-0.776**	-0.121	0.033	0.660**	0.609**
**SDG 5**					1	0.599**	0.558**	0.605**	0.477**	0.124	0.604**	-0.478**	-0.459**	-0.096	-0.020	0.422**	0.651**
**SDG 6**						1	0.854**	0.714**	0.814**	0.171	0.753**	-0.630**	-0.748**	-0.011	0.031	0.625**	0.698**
**SDG 7**							1	0.673**	0.808**	0.275	0.755**	-0.714**	-0.838**	-0.165	0.059	0.589**	0.645**
**SDG 8**								1	0.773**	0.409**	0.640**	-0.583**	-0.694**	-0.101	0.176	0.693**	0.557**
**SDG 9**									1	0.368*	0.709**	-0.668**	-0.826**	-0.266	0.000	0.696**	0.530**
**SDG 10**										1	0.213	-0.268	-0.360*	-0.412*	0.116	0.471**	0.195
**SDG 11**											1	-0.661**	-0.764**	-0.106	0.004	0.663**	0.557**
**SDG 12**												1	0.787**	0.236	-0.129	-0.604**	-0.527**
**SDG 13**													1	0.254	-0.017	-0.649**	-0.548**
**SDG 14**														1	0.138	-0.292	-0.049
**SDG 15**															1	0.035	-0.077
**SDG 16**																1	0.428**
**SDG 17**																	1

C2. Moderately arid

r	SDG 1	SDG 2	SDG 3	SDG 4	SDG 5	SDG 6	SDG 7	SDG 8	SDG 9	SDG 10	SDG 11	SDG 12	SDG 13	SDG 14	SDG 15	SDG 16	SDG 17
**SDG 1**	1	0.668**	0.826**	0.747**	0.139	0.846**	0.805**	0.667**	0.867**	0.619**	0.723**	-0.846**	-0.854**	-0.918**	0.098	0.642**	0.132
**SDG 2**		1	0.774**	0.537*	0.358	0.663**	0.511*	0.593**	0.584**	0.419	0.646**	-0.614**	-0.586**	-0.645*	0.044	0.488*	-0.074
**SDG 3**			1	0.784**	0.263	0.782**	0.835**	0.716**	0.811**	0.668**	0.761**	-0.811**	-0.782**	-0.782**	0.042	0.863**	0.135
**SDG 4**				1	0.218	0.670**	0.693**	0.563*	0.746**	0.539*	0.721**	-0.772**	-0.730**	-0.691*	-0.012	0.753**	0.319
**SDG 5**					1	0.228	0.137	0.312	0.233	0.056	0.609**	-0.316	-0.246	-0.218	-0.046	0.333	0.130
**SDG 6**						1	0.800**	0.551*	0.839**	0.425	0.763**	-0.923**	-0.932**	-0.873**	0.284	0.658**	0.251
**SDG 7**							1	0.640**	0.707**	0.633**	0.782**	-0.835**	-0.849**	-0.827**	0.168	0.782**	0.300
**SDG 8**								1	0.663**	0.602**	0.686**	-0.649**	-0.549*	-0.382	-0.019	0.686**	-0.079
**SDG 9**									1	0.463*	0.647**	-0.823**	-0.847**	-0.855**	0.128	0.737**	0.093
**SDG 10**										1	0.551*	-0.505*	-0.426	-0.500	0.268	0.642**	0.037
**SDG 11**											1	-0.839**	-0.770**	-0.645*	0.151	0.726**	0.389
**SDG 12**												1	0.949**	0.745**	-0.293	-0.719**	-0.333
**SDG 13**													1	0.873**	-0.291	-0.704**	-0.370
**SDG 14**														1	0.045	-0.727*	-0.345
**SDG 15**															1	0.086	0.347
**SDG 16**																1	0.325
**SDG 17**																	1

C3. Severely arid

r	SDG 1	SDG 2	SDG 3	SDG 4	SDG 5	SDG 6	SDG 7	SDG 8	SDG 9	SDG 10	SDG 11	SDG 12	SDG 13	SDG 14	SDG 15	SDG 16	SDG 17
**SDG 1**	1	0.301	0.814**	0.723**	0.301	0.647**	0.781**	0.435*	0.701**	0.755**	0.661**	-0.634**	-0.755**	-0.459	-0.004	0.717**	0.100
**SDG 2**		1	0.629**	0.454*	0.190	0.227	0.580**	0.448*	0.605**	0.222	0.336	-0.242	-0.426*	-0.177	-0.121	0.546**	-0.203
**SDG 3**			1	0.830**	0.440*	0.700**	0.883**	0.681**	0.853**	0.614**	0.618**	-0.725**	-0.796**	-0.456*	-0.150	0.816**	-0.026
**SDG 4**				1	0.608**	0.653**	0.696**	0.649**	0.784**	0.470*	0.598**	-0.672**	-0.770**	-0.405	-0.041	0.644**	0.176
**SDG 5**					1	0.427*	0.253	0.428*	0.604**	-0.106	0.615**	-0.424*	-0.480*	-0.032	-0.035	0.305	0.316
**SDG 6**						1	0.644**	0.557**	0.540**	0.297	0.685**	-0.544**	-0.606**	-0.618**	-0.160	0.456*	0.395*
**SDG 7**							1	0.458*	0.748**	0.621**	0.563**	-0.625**	-0.655**	-0.549*	-0.164	0.701**	0.065
**SDG 8**								1	0.611**	0.188	0.459*	-0.559**	-0.531**	-0.472*	0.119	0.612**	-0.012
**SDG 9**									1	0.452*	0.640**	-0.729**	-0.818**	-0.079	-0.154	0.715**	0.078
**SDG 10**										1	0.280	-0.509*	-0.635**	-0.152	0.056	0.657**	-0.153
**SDG 11**											1	-0.386	-0.403*	-0.270	0.019	0.507**	0.493*
**SDG 12**												1	0.797**	0.260	0.074	-0.596**	0.118
**SDG 13**													1	0.126	0.231	-0.670**	-0.061
**SDG 14**														1	0.058	-0.004	0.089
**SDG 15**															1	0.080	0.030
**SDG 16**																1	0.020
**SDG 17**																	1

C4. Extremely arid

r	SDG 1	SDG 2	SDG 3	SDG 4	SDG 5	SDG 6	SDG 7	SDG 8	SDG 9	SDG 10	SDG 11	SDG 12	SDG 13	SDG 14	SDG 15	SDG 16	SDG 17
**SDG 1**	1	0.479	0.788**	0.700**	0.459	0.579*	0.809**	-0.215	0.824**	0.029	0.485	-0.350	-0.671**	-0.018	-0.115	0.644**	0.512*
**SDG 2**		1	0.624**	0.384	0.338	0.326	0.577**	0.414	0.651**	0.188	0.427	-0.320	-0.526*	-0.143	0.146	0.645**	0.496*
**SDG 3**			1	0.825**	0.541*	0.322	0.866**	0.212	0.890**	-0.256	0.529*	-0.764**	-0.851**	-0.099	-0.277	0.901**	0.549*
**SDG 4**				1	0.484*	0.358	0.767**	0.191	0.763**	-0.332	0.623**	-0.763**	-0.856**	-0.077	-0.165	0.740**	0.484*
**SDG 5**					1	0.738**	0.459*	0.256	0.514*	-0.368	0.620**	-0.564**	-0.629**	-0.152	0.047	0.665**	0.498*
**SDG 6**						1	0.280	0.182	0.421	-0.247	0.714**	-0.206	-0.365	-0.002	0.226	0.394	0.417
**SDG 7**							1	-0.039	0.794**	-0.188	0.448*	-0.708**	-0.823**	-0.112	-0.346	0.820**	0.469*
**SDG 8**								1	0.179	0.206	0.370	-0.086	-0.226	-0.015	0.152	0.347	0.200
**SDG 9**									1	-0.256	0.439	-0.750**	-0.776**	-0.051	-0.117	0.785**	0.657**
**SDG 10**										1	-0.350	0.444	0.385	-0.536	-0.082	-0.197	-0.159
**SDG 11**											1	-0.403	-0.544*	0.398	0.183	0.632**	0.326
**SDG 12**												1	0.844**	0.116	0.122	-0.771**	-0.487*
**SDG 13**													1	-0.020	0.086	-0.814**	-0.555*
**SDG 14**														1	0.240	-0.165	0.059
**SDG 15**															1	-0.114	0.191
**SDG 16**																1	0.531*
**SDG 17**																	1

Table S5. Projections of the mean index score (MIS) and sustainable development score (SDS) in 2030. N.A. is short for not available, because the amount of data in these countries is insufficient for regression analysis (less than 3). The value of SD ratio test below 0.35 is good for gray forecast model, 0.35~0.5 is qualified, 0.5~0.65 is barely qualified, and above 0.65 is not qualified.

Countries	Curvilinear regressions			Gray forecast model		average MIS	Curvilinear regressions			Gray forecast model		average SDS
	Model	MIS	p-value	MIS	SD ratio test		Model	SDS	p-value	SDS	SD ratio test	
Afghanistan	Logistic	58.96	<0.001	65.47	0.548	62.22	Compound/Logistic	52.45	<0.001	54.58	0.704	53.52
Albania	Linear	76.75	0.013	77.84	0.292	77.29	Compound/Logistic	75.01	<0.001	75.00	0.358	75.01
Algeria	Logistic	80.29	<0.001	81.80	0.648	81.05	Compound/Logistic	92.46	<0.001	93.97	0.195	93.22
Angola	Logistic	54.47	<0.001	54.67	0.902	54.57	Compound/Logistic	50.69	<0.001	51.10	0.896	50.89
Argentina	Logistic	76.27	<0.001	81.42	0.546	78.84	Compound/Logistic	74.36	<0.001	77.10	0.799	75.73
Armenia	Logistic	74.33	<0.001	82.08	0.326	78.21	Compound/Logistic	76.18	<0.001	81.08	0.362	78.63
Australia	Logistic	76.18	<0.001	84.42	0.068	80.30	Compound/Logistic	59.78	<0.001	61.77	0.734	60.77
Austria	Logistic	88.48	<0.001	92.00	0.611	90.24	Compound/Logistic	78.48	<0.001	81.40	0.400	79.94
Azerbaijan	Logistic	80.52	<0.001	83.75	0.499	82.13	Compound/Logistic	70.04	<0.001	74.35	0.878	72.19
Bahrain	Logistic	67.03	<0.001	61.97	0.805	64.50	Compound/Logistic	72.36	<0.001	66.91	0.807	69.64
Bangladesh	Logistic	85.27	<0.001	79.53	0.193	82.40	Compound/Logistic	79.16	<0.001	78.87	0.198	79.01
Barbados	N. A.	N. A.	N. A.	N. A.	N. A.	N. A.	N. A.	N. A.	N. A.	N. A.	N. A.	N. A.
Belarus	Logistic	90.28	<0.001	93.06	0.527	91.67	Compound/Logistic	88.08	<0.001	91.43	0.371	89.75
Belgium	Compound/Logistic	86.11	<0.001	92.05	0.437	89.08	Compound/Logistic	81.17	<0.001	95.08	0.692	88.12
Belize	Logistic	71.88	<0.001	83.68	0.319	77.78	Compound/Logistic	61.52	<0.001	69.19	0.351	65.35
Benin	Logistic	56.40	<0.001	56.30	0.882	56.35	Compound/Logistic	53.97	<0.001	56.59	0.881	55.28
Bhutan	Compound/Logistic	86.50	<0.001	88.47	0.321	87.49	Compound/Logistic	89.33	<0.001	91.38	0.168	90.35
Bolivia	Logistic	77.53	<0.001	68.61	0.392	73.07	Compound/Logistic	75.29	<0.001	70.15	0.340	72.72
Bosnia and Herzegovina	Compound/Logistic	99.80	<0.001	99.88	0.225	99.84	Compound/Logistic	94.31	<0.001	99.03	0.352	96.67
Botswana	Linear	70.24	<0.001	72.26	0.230	71.25	Compound/Logistic	63.47	<0.001	61.80	0.410	62.64
Brazil	Compound/Logistic	78.46	<0.001	78.78	0.587	78.62	Compound/Logistic	64.17	<0.001	63.31	0.980	63.74
Brunei Darussalam	N. A.	N. A.	N. A.	N. A.	N. A.	N. A.	N. A.	N. A.	N. A.	N. A.	N. A.	N. A.
Bulgaria	Compound/Logistic	78.59	<0.001	76.51	0.622	77.55	Compound/Logistic	83.41	<0.001	83.29	0.488	83.35
Burkina Faso	Logistic	63.19	<0.001	60.99	0.583	62.09	Compound/Logistic	52.71	<0.001	52.64	0.999	52.68
Burundi	Logistic	52.39	<0.001	55.84	0.869	54.12	Compound/Logistic	42.91	<0.001	45.56	0.330	44.24
Cabo Verde	Logistic	80.79	<0.001	84.12	0.169	82.45	Compound/Logistic	68.38	<0.001	74.23	0.168	71.30
Cambodia	Linear	80.27	0.004	80.67	0.194	80.47	Linear	76.49	0.003	76.03	0.139	76.26
Cameroon	Compound/Logistic	61.93	<0.001	54.81	0.306	58.37	Compound/Logistic	60.79	<0.001	57.12	0.233	58.96
Canada	Compound/Logistic	82.14	<0.001	86.15	0.197	84.14	Compound/Logistic	75.11	<0.001	77.13	0.530	76.12
Central African Republic	Compound/Logistic	35.64	<0.001	32.47	0.513	34.05	Compound/Logistic	38.92	<0.001	35.40	0.619	37.16
Chad	Compound/Logistic	36.37	<0.001	32.13	0.585	34.25	Compound/Logistic	37.93	<0.001	35.73	0.471	36.83
Chile	Compound/Logistic	94.47	<0.001	92.85	0.306	93.66	Compound/Logistic	73.30	<0.001	71.10	0.867	72.20
China	Compound/Logistic	88.31	<0.001	79.59	0.457	83.95	Compound/Logistic	83.84	<0.001	76.19	0.621	80.02
Colombia	Compound/Logistic	88.41	<0.001	84.54	0.315	86.48	Compound/Logistic	70.99	<0.001	67.88	0.806	69.43
Comoros	N. A.	N. A.	N. A.	N. A.	N. A.	N. A.	N. A.	N. A.	N. A.	N. A.	N. A.	N. A.
Congo, Dem. Rep.	Logistic	73.31	<0.001	77.08	0.328	75.19	Compound/Logistic	59.19	<0.001	59.66	0.294	59.42

Congo, Rep.	Compound/Logistic	61.46	<0.001	56.58	0.638	59.02	S-function	53.18	0.173	48.20	0.465	50.69
Costa Rica	Logistic	84.58	<0.001	75.55	0.393	80.07	Compound/Logistic	66.66	<0.001	62.26	0.852	64.46
Cote d'Ivoire	Compound/Logistic	69.73	<0.001	67.27	0.281	68.50	Compound/Logistic	62.97	<0.001	63.40	0.214	63.18
Croatia	Compound/Logistic	88.32	<0.001	92.18	0.185	90.25	Compound/Logistic	95.68	<0.001	100.00	0.223	97.84
Cuba	Compound/Logistic	79.07	<0.001	93.69	0.125	86.38	Compound/Logistic	75.57	<0.001	86.81	0.274	81.19
Cyprus	Compound/Logistic	88.40	<0.001	94.96	0.467	91.68	Compound/Logistic	87.60	<0.001	98.20	0.245	92.90
Czech Republic	Compound/Logistic	86.56	<0.001	93.37	0.652	89.97	Compound/Logistic	77.78	<0.001	87.15	0.628	82.46
Denmark	Logistic	86.17	<0.001	84.92	0.670	85.55	Compound/Logistic	77.23	<0.001	71.94	0.443	74.59
Djibouti	Logistic	67.39	<0.001	67.51	0.392	67.45	Compound/Logistic	63.01	<0.001	65.45	0.512	64.23
Dominican Republic	Logistic	81.85	<0.001	84.73	0.407	83.29	Compound/Logistic	77.33	<0.001	75.56	0.605	76.45
Ecuador	Logistic	84.35	<0.001	80.45	0.476	82.40	Compound/Logistic	75.00	<0.001	70.44	0.799	72.72
Egypt, Arab Rep.	Logistic	81.99	<0.001	88.50	0.343	85.24	Compound/Logistic	88.19	<0.001	95.37	0.108	91.78
El Salvador	Logistic	85.83	<0.001	83.77	0.446	84.80	Compound/Logistic	78.80	<0.001	77.81	0.461	78.30
Estonia	Logistic	88.76	<0.001	90.79	0.322	89.77	Compound/Logistic	78.28	<0.001	80.84	0.924	79.56
Eswatini	Compound/Logistic	56.93	0.005	49.77	0.001	53.35	Compound/Logistic	58.16	0.01	45.31	0.001	51.74
Ethiopia	Compound/Logistic	55.64	<0.001	56.72	0.840	56.18	Compound/Logistic	46.42	<0.001	48.41	0.509	47.42
Fiji	Compound	76.51	0.004	84.05	0.002	80.28	Compound/Logistic	82.15	0.005	93.50	0.003	87.82
Finland	Compound/Logistic	88.99	<0.001	94.71	0.466	91.85	Compound/Logistic	84.14	<0.001	89.61	0.501	86.88
Former Yugoslav Republic of Macedonia (FYROM)	N. A.	N. A.	N. A.	N. A.	N. A.	N. A.	N. A.	N. A.	N. A.	N. A.	N. A.	N. A.
France	Logistic	84.11	<0.001	82.44	0.355	83.27	Compound/Logistic	80.89	<0.001	82.27	0.337	81.58
Gabon	Compound/Logistic	59.47	<0.001	61.99	0.711	60.73	Compound/Logistic	74.72	<0.001	75.15	0.420	74.94
Gambia, The	Compound/Logistic	98.50	<0.001	90.51	0.123	94.50	Compound/Logistic	66.40	<0.001	69.79	0.209	68.09
Georgia	Compound/Logistic	80.47	<0.001	79.54	0.581	80.00	Compound/Logistic	80.11	<0.001	78.42	0.412	79.26
Germany	Compound/Logistic	81.75	<0.001	81.97	0.999	81.86	Compound/Logistic	81.83	<0.001	88.57	0.589	85.20
Ghana	Compound/Logistic	72.10	<0.001	64.26	0.558	68.18	Compound/Logistic	70.03	<0.001	63.89	0.617	66.96
Greece	Compound/Logistic	83.09	<0.001	93.44	0.228	88.27	Compound/Logistic	84.42	<0.001	95.96	0.392	90.19
Guatemala	Compound/Logistic	67.11	<0.001	67.48	0.653	67.29	Compound/Logistic	58.78	<0.001	58.21	0.972	58.49
Guinea	S-function	52.99	0.109	48.26	0.338	50.63	Compound/Logistic	51.82	<0.001	46.04	0.400	48.93
Guyana	Compound/Logistic	51.40	<0.001	53.36	0.377	52.38	Compound/Logistic	56.19	<0.001	60.41	0.678	58.30
Haiti	Compound/Logistic	72.25	<0.001	61.62	0.279	66.94	Compound/Logistic	67.28	<0.001	59.68	0.205	63.48
Honduras	S-function	64.10	0.155	61.96	0.548	63.03	Compound/Logistic	58.44	<0.001	56.65	0.806	57.55
Hungary	Compound/Logistic	86.00	<0.001	94.38	0.443	90.19	Compound/Logistic	78.19	<0.001	81.55	0.860	79.87
Iceland	Compound/Logistic	74.00	<0.001	72.18	0.535	73.09	Compound/Logistic	67.12	<0.001	65.43	0.719	66.27
India	Compound/Logistic	68.14	<0.001	64.73	0.639	66.43	Compound/Logistic	61.04	<0.001	60.52	0.984	60.78
Indonesia	Compound/Logistic	75.47	<0.001	78.08	0.060	76.77	Compound/Logistic	84.71	<0.001	89.41	0.172	87.06
Iran, Islamic Rep.	Compound/Logistic	90.06	<0.001	86.44	0.536	88.25	Compound/Logistic	98.35	<0.001	92.48	0.416	95.42
Iraq	Compound/Logistic	92.37	<0.001	100	0.396	96.18	Linear	92.79	0.043	100	0.431	96.40
Ireland	Compound/Logistic	88.04	<0.001	92.25	0.143	90.14	Compound/Logistic	80.63	<0.001	92.89	0.160	86.76
Israel	Compound/Logistic	87.78	<0.001	87.81	0.331	87.79	Compound/Logistic	78.53	<0.001	79.22	0.875	78.87
Italy	Compound/Logistic	87.14	<0.001	93.72	0.059	90.43	Compound/Logistic	85.07	<0.001	95.13	0.163	90.10
Jamaica	Compound/Logistic	76.65	<0.001	78.24	0.530	77.45	Compound/Logistic	88.11	<0.001	92.80	0.262	90.46
Japan	Compound/Logistic	79.31	<0.001	83.69	0.141	81.50	S-function	75.97	0.035	74.66	0.416	75.32
Jordan	Compound/Logistic	81.90	<0.001	88.34	0.355	85.12	Compound/Logistic	86.92	<0.001	94.78	0.217	90.85
Kazakhstan	Compound/Logistic	77.75	<0.001	89.25	0.294	83.50	Compound/Logistic	74.00	<0.001	82.90	0.457	78.45
Kenya	Compound/Logistic	76.49	<0.001	75.96	0.263	76.23	Compound/Logistic	60.56	<0.001	59.50	0.985	60.03
Korea, Rep.	S-function	79.17	<0.001	82.00	0.166	80.59	Power function	78.76	0.005	82.78	0.288	80.77

Kuwait	S-function	60.39	0.022	58.45	0.291	59.42	Compound/Logistic	64.15	<0.001	70.61	0.636	67.38
Kyrgyz Republic	Compound/Logistic	87.30	<0.001	89.56	0.332	88.43	Compound/Logistic	86.27	<0.001	86.43	0.476	86.35
Lao PDR	Compound/Logistic	68.32	<0.001	70.07	0.581	69.19	Compound/Logistic	60.59	<0.001	63.10	0.550	61.84
Latvia	Linear	88.72	0.016	93.08	0.213	90.90	Compound/Logistic	79.84	<0.001	78.54	0.774	79.19
Lebanon	Compound/Logistic	72.36	<0.001	73.93	0.224	73.14	Compound/Logistic	69.44	<0.001	73.56	0.251	71.50
Lesotho	Compound/Logistic	56.28	<0.001	63.14	0.540	59.71	Compound/Logistic	53.63	<0.001	64.51	0.499	59.07
Liberia	S-function	49.70	0.037	48.05	0.236	48.87	Compound/Logistic	53.05	<0.001	43.79	0.325	48.42
Lithuania	Compound/Logistic	84.21	<0.001	87.69	0.335	85.95	Compound/Logistic	80.59	<0.001	81.90	0.558	81.24
Luxembourg	Compound/Logistic	74.33	<0.001	70.93	0.378	72.63	Compound/Logistic	60.60	<0.001	58.61	0.435	59.61
Madagascar	Logistic	65.99	<0.001	62.88	0.230	64.43	Compound/Logistic	55.42	<0.001	52.37	0.268	53.89
Malawi	S-function	51.69	0.028	52.16	0.431	51.92	Compound/Logistic	48.89	<0.001	46.37	0.364	47.63
Malaysia	Compound/Logistic	75.09	<0.001	75.81	0.698	75.45	Compound/Logistic	71.71	<0.001	67.36	0.181	69.54
Maldives	Compound/Logistic	56.92	0.016	86.41	0.001	71.66	S-function	62.47	0.578	100	0.005	81.24
Mali	Power function	52.63	0.002	55.86	0.105	54.24	Compound/Logistic	52.14	<0.001	53.30	0.498	52.72
Malta	Compound/Logistic	74.99	<0.001	80.35	0.564	77.67	Compound/Logistic	73.46	<0.001	76.28	0.732	74.87
Mauritania	Compound/Logistic	72.91	<0.001	74.11	0.525	73.51	Compound/Logistic	73.00	<0.001	71.78	0.505	72.39
Mauritius	Compound/Logistic	74.07	<0.001	72.30	0.583	73.18	Compound/Logistic	75.05	<0.001	68.47	0.281	71.76
Mexico	Compound/Logistic	74.51	<0.001	84.06	0.604	79.28	Compound/Logistic	57.07	<0.001	67.79	0.587	62.43
Moldova	S-function	75.61	0.376	74.40	0.859	75.01	Compound/Logistic	70.00	<0.001	67.42	0.690	68.71
Mongolia	Compound/Logistic	64.70	<0.001	63.50	0.970	64.10	Compound/Logistic	67.42	<0.001	66.99	0.529	67.20
Montenegro	Compound/Logistic	73.08	<0.001	73.36	0.823	73.22	Compound/Logistic	74.15	<0.001	77.21	0.339	75.68
Morocco	Compound/Logistic	84.33	<0.001	86.75	0.421	85.54	Compound/Logistic	89.34	<0.001	92.14	0.229	90.74
Mozambique	Compound/Logistic	60.09	<0.001	54.62	0.718	57.36	S-function	52.43	0.4	48.64	0.714	50.53
Myanmar	Compound/Logistic	82.89	<0.001	87.53	0.270	85.21	Compound/Logistic	75.00	<0.001	74.65	0.250	74.83
Namibia	Compound/Logistic	69.36	<0.001	72.33	0.260	70.84	Compound/Logistic	64.49	<0.001	67.06	0.288	65.77
Nepal	Compound/Exponential/Logistic	82.58	<0.001	81.53	0.215	82.05	Compound/Logistic	79.89	<0.001	80.82	0.126	80.35
Netherlands	Compound/Logistic	85.01	<0.001	87.29	0.317	86.15	Compound/Logistic	80.47	<0.001	88.77	0.064	84.62
New Zealand	Compound/Logistic	82.39	<0.001	81.17	0.781	81.78	Compound/Logistic	85.77	<0.001	82.18	0.599	83.97
Nicaragua	Compound/Logistic	76.83	<0.001	67.77	0.474	72.30	S-function	62.50	0.041	61.18	0.355	61.84
Niger	S-function	50.11	0.023	48.52	0.207	49.31	Compound/Logistic	47.64	<0.001	43.92	0.379	45.78
Nigeria	Compound/Logistic	50.78	<0.001	56.25	0.692	53.52	Compound/Logistic	54.81	<0.001	61.91	0.486	58.36
North Macedonia	Compound/Logistic	84.17	<0.001	85.19	0.001	84.68	Compound/Logistic	76.13	0.003	70.82	0.001	73.47
Norway	Compound/Logistic	77.12	<0.001	83.84	0.323	80.48	Power function	68.08	0.005	63.93	0.263	66.00
Oman	Compound/Logistic	83.37	<0.001	87.91	0.229	85.64	Compound/Logistic	83.97	<0.001	88.31	0.458	86.14
Pakistan	Compound/Logistic	62.29	<0.001	66.55	0.224	64.42	Compound/Logistic	61.39	<0.001	66.63	0.493	64.01
Panama	Compound	81.59	<0.001	81.02	0.423	81.30	Compound/Logistic	68.12	<0.001	67.81	0.933	67.97
Papua New Guinea	Compound/Logistic	50.13	0.001	48.45	0.001	49.29	Compound/Logistic	48.70	<0.001	48.86	0.001	48.78
Paraguay	S-function	68.22	0.107	67.34	0.591	67.78	Compound/Logistic	68.62	<0.001	66.08	0.808	67.35
Peru	Compound/Logistic	86.48	<0.001	80.02	0.353	83.25	S-function	69.24	0.032	74.41	0.506	71.82
Philippines	S-function	65.16	0.331	63.94	0.724	64.55	Compound/Logistic	63.36	<0.001	59.66	0.890	61.51
Poland	Compound/Logistic	92.71	<0.001	100	0.026	96.36	Compound/Logistic	100	<0.001	100	0.136	100.00
Portugal	Compound/Logistic	87.96	<0.001	94.17	0.212	91.06	Compound/Logistic	77.78	<0.001	76.82	0.924	77.30
Qatar	Compound/Logistic	70.04	<0.001	76.19	0.613	73.12	Compound/Logistic	58.31	<0.001	60.86	0.950	59.58
Romania	Compound/Logistic	79.57	<0.001	88.75	0.275	84.16	Compound/Logistic	86.81	<0.001	98.99	0.348	92.90
Russian Federation	Compound/Logistic	86.21	<0.001	89.65	0.108	87.93	Compound/Exponential/Logistic	93.78	<0.001	94.22	0.099	94.00
Rwanda	Power function	57.71	0.032	58.94	0.344	58.32	Compound/Logistic	54.31	<0.001	55.43	0.846	54.87
Sao Tome and Principe	Compound/Logistic	36.29	<0.001	33.53	0.003	34.91	Power function	53.07	0.03	45.02	0.001	49.04

Saudi Arabia	Compound/Logistic	69.20	<0.001	70.89	0.429	70.04	Power function	71.22	0.024	70.46	0.102	70.84
Senegal	Compound/Logistic	63.85	<0.001	63.01	0.205	63.43	Compound/Logistic	57.24	<0.001	58.93	0.930	58.08
Serbia	Compound/Logistic	86.73	<0.001	92.63	0.274	89.68	Compound/Logistic	88.96	<0.001	95.02	0.207	91.99
Sierra Leone	Compound/Logistic	64.89	<0.001	62.68	0.306	63.78	Compound/Logistic	53.98	<0.001	55.10	0.680	54.54
Singapore	Compound/Logistic	69.85	<0.001	66.04	0.905	67.95	Compound/Logistic	60.22	<0.001	61.81	0.834	61.02
Slovak Republic	Compound	90.08	<0.001	96.65	0.145	93.37	Compound/Logistic	85.63	<0.001	89.68	0.344	87.66
Slovenia	Compound/Logistic	82.40	<0.001	85.94	0.698	84.17	Compound/Logistic	78.18	<0.001	89.63	0.782	83.91
Somalia	N. A.	N. A.	N. A.	N. A.	N. A.	N. A.	N. A.	N. A.	N. A.	N. A.	N. A.	N. A.
South Africa	Compound/Logistic	71.01	<0.001	74.61	0.249	72.81	Compound/Logistic	63.12	<0.001	65.80	0.542	64.46
South Sudan	N. A.	N. A.	N. A.	N. A.	N. A.	N. A.	N. A.	N. A.	N. A.	N. A.	N. A.	N. A.
Spain	Compound	86.94	<0.001	91.80	0.300	89.37	Compound/Logistic	85.35	<0.001	86.50	0.501	85.92
Sri Lanka	Compound/Logistic	74.00	<0.001	79.76	0.041	76.88	Compound/Logistic	81.41	<0.001	88.99	0.262	85.20
Sudan	Compound/Logistic	49.10	<0.001	47.87	0.953	48.48	Power function	47.09	0.006	41.65	0.255	44.37
Suriname	Compound/Logistic	69.83	<0.001	75.31	0.527	72.57	Compound/Logistic	65.57	<0.001	73.20	0.447	69.39
Swaziland	N. A.	N. A.	N. A.	N. A.	N. A.	N. A.	N. A.	N. A.	N. A.	N. A.	N. A.	N. A.
Sweden	Compound/Logistic	84.91	<0.001	86.80	0.675	85.86	Compound/Logistic	79.37	<0.001	85.53	0.788	82.45
Switzerland	Compound/Logistic	81.19	<0.001	84.02	0.227	82.61	Compound/Logistic	76.01	<0.001	80.15	0.771	78.08
Syrian Arab Republic	Compound/Logistic	53.87	<0.001	60.06	0.517	56.97	Compound/Logistic	50.27	<0.001	57.21	0.424	53.74
Tajikistan	Compound/Logistic	81.40	<0.001	79.50	0.560	80.45	Compound/Logistic	65.05	<0.001	67.44	0.776	66.24
Tanzania	Compound/Logistic	67.84	<0.001	61.12	0.139	64.48	Compound/Logistic	60.52	<0.001	56.51	0.423	58.52
Thailand	Compound/Logistic	90.11	<0.001	91.88	0.412	90.99	Compound/Logistic	92.77	<0.001	95.69	0.247	94.23
Timor-Leste	N. A.	N. A.	N. A.	N. A.	N. A.	N. A.	N. A.	N. A.	N. A.	N. A.	N. A.	N. A.
Togo	Power function	54.93	0.018	57.67	0.281	56.30	Compound/Logistic	55.63	<0.001	55.17	0.299	55.40
Trinidad and Tobago	Compound/Logistic	60.69	<0.001	60.30	0.694	60.50	Compound/Logistic	58.94	<0.001	59.51	0.616	59.23
Tunisia	Compound/Logistic	82.45	<0.001	90.14	0.440	86.30	Compound/Logistic	86.70	<0.001	93.63	0.242	90.16
Turkey	Compound/Logistic	78.26	<0.001	86.41	0.355	82.33	Compound/Logistic	70.79	<0.001	81.61	0.430	76.20
Turkmenistan	Compound/Logistic	74.43	<0.001	63.89	0.624	69.16	Power function	64.73	0.036	75.32	0.496	70.03
Uganda	Compound/Logistic	50.20	<0.001	46.46	0.654	48.33	Compound/Logistic	51.09	<0.001	51.00	0.990	51.04
Ukraine	Compound/Logistic	82.16	<0.001	86.18	0.172	84.17	Compound/Logistic	80.03	<0.001	85.00	0.238	82.52
United Arab Emirates	Compound/Logistic	77.82	<0.001	72.84	0.384	75.33	Compound/Logistic	70.01	<0.001	66.33	0.367	68.17
United Kingdom	Compound/Exponential/Logistic	84.34	<0.001	84.12	0.182	84.23	Compound/Logistic	88.65	<0.001	88.68	0.202	88.67
United States	Compound/Exponential/Logistic	87.10	<0.001	87.15	0.323	87.12	Compound/Logistic	84.10	<0.001	88.83	0.401	86.46
Uruguay	Linear	84.51	0.024	89.27	0.266	86.89	Compound/Logistic	83.33	<0.001	90.05	0.311	86.69
Uzbekistan	Compound/Logistic	71.39	<0.001	70.82	0.996	71.10	Compound/Logistic	72.12	<0.001	67.48	0.645	69.80
Vanuatu	Compound/Logistic	63.78	<0.001	57.37	0.001	60.57	Compound/Logistic	58.59	0.011	76.74	0.002	67.67
Venezuela, RB	Linear	45.91	0.001	47.82	0.154	46.87	Compound/Logistic	47.82	<0.001	48.07	0.386	47.95
Vietnam	Compound/Logistic	88.36	<0.001	85.82	0.344	87.09	Compound/Logistic	98.70	<0.001	100.00	0.327	99.35
Yemen, Rep.	Compound/Logistic	55.58	<0.001	64.21	0.747	59.90	Compound/Logistic	55.35	<0.001	59.60	0.802	57.47
Zambia	Compound/Logistic	54.06	<0.001	49.93	0.409	51.99	Compound/Logistic	40.87	<0.001	39.42	0.316	40.15
Zimbabwe	Compound/Logistic	71.61	<0.001	63.20	0.498	67.40	Compound/Logistic	58.91	<0.001	57.83	0.626	58.37

Table S6. Details for priority selections. EHN, EP, SD, and ED refer to SDGs related to essential human needs, eco-environmental protection, social development, and economic development, respectively. UD, UE, and SS stand for relatively underdeveloped, uneven, and sustainable status. N.A. is short for not available.

Countries	2017				2021				Development status in 2021	Development pathway	Best performance in 2021	Worst performance in 2021	Change in EHN
	EHN	EP	SD	ED	EHN	EP	SD	ED					
Afghanistan	45.52	69.10	48.27	24.25	49.59	80.96	44.10	25.35	UD & UE	Progression	EP	ED	4.08
Albania	77.58	65.83	70.53	55.82	79.62	74.05	70.11	54.17	SS	Progression	EHN	ED	2.04
Algeria	74.51	69.37	72.68	53.16	75.37	70.42	76.14	55.11	SS	Progression	SD	ED	0.86
Angola	44.50	68.25	49.60	36.54	42.43	80.11	43.14	35.57	UD & UE	Retrogression in ES	EP	ED	-2.07
Argentina	86.16	65.85	66.09	69.01	83.14	70.15	68.87	65.65	SS	Progression	EHN	ED	-3.02
Armenia	79.85	75.24	71.72	54.64	79.17	81.18	69.02	59.04	SS	Progression	EP	ED	-0.68
Australia	87.15	48.93	78.68	88.33	88.05	44.22	81.62	86.52	UE	Retrogression in both	EHN	EP	0.90
Austria	90.98	65.62	78.65	85.88	89.93	58.45	87.02	90.63	SS	Retrogression in ES	ED	EP	-1.05
Azerbaijan	78.31	73.66	68.55	59.22	78.80	79.56	70.56	62.20	SS	Retrogression in ES	EP	ED	0.48
Bahrain	78.15	48.80	62.20	66.25	81.43	52.93	63.56	57.60	SS	Retrogression in MIS	EHN	EP	3.28
Bangladesh	63.97	63.62	58.51	29.54	62.33	77.65	61.52	49.64	UD & UE	Progression	EP	ED	-1.65
Barbados	80.85	47.64	61.69	72.81	77.04	60.66	80.85	61.49	SS	Retrogression in ES	SD	EP	-3.81
Belarus	82.11	75.14	77.09	70.86	83.79	84.62	83.81	63.08	SS	Progression	EP	ED	1.68
Belgium	89.01	62.85	82.56	83.34	86.96	66.01	86.11	89.29	SS	Progression	ED	EP	-2.05
Belize	81.01	54.00	63.40	60.59	71.05	71.36	66.60	53.43	UE	Retrogression in ES	EP	ED	-9.96
Benin	43.31	66.15	48.19	39.60	37.85	80.18	41.53	43.39	UD & UE	Retrogression in ES	EP	EHN	-5.46
Bhutan	75.16	76.31	59.98	47.82	71.58	78.84	69.41	62.52	SS	Progression	EP	ED	-3.58
Bolivia	72.04	78.49	57.21	51.13	71.05	81.75	63.81	55.62	UE	Progression	EP	ED	-0.99
Bosnia and Herzegovina	79.25	57.42	68.17	48.65	80.17	77.34	73.41	58.55	SS	Progression	EHN	ED	0.93
Botswana	58.58	59.87	58.04	56.47	56.01	75.40	59.05	61.07	UD & UE	Progression	EP	EHN	-2.58
Brazil	84.31	69.09	56.07	67.77	83.15	72.12	61.53	66.96	UE	Retrogression in ES	EHN	SD	-1.16
Brunei Darussalam	N. A.	N. A.	N. A.	N. A.	77.03	46.21	73.86	77.97	UE	N. A.	ED	EP	N. A.
Bulgaria	80.43	73.14	70.17	62.37	81.05	76.32	66.86	70.00	SS	Progression	EHN	SD	0.62
Burkina Faso	43.98	82.81	48.54	29.13	35.76	90.58	50.74	45.80	UD & UE	Retrogression in ES	EP	EHN	-8.22
Burundi	33.60	78.17	65.44	32.83	31.10	86.61	58.36	35.14	UD & UE	Retrogression in both	EP	EHN	-2.50
Cabo Verde	N. A.	N. A.	N. A.	N. A.	69.86	76.48	64.69	59.93	SS	N. A.	EP	ED	N. A.
Cambodia	63.25	54.56	61.92	48.28	66.54	73.02	63.45	51.72	UE	Progression	EP	ED	3.28
Cameroon	56.97	70.87	53.01	21.55	51.33	83.00	46.45	39.51	UD & UE	Progression	EP	ED	-5.64
Canada	87.50	56.21	81.10	86.14	88.33	55.53	84.43	86.58	SS	Retrogression in ES	EHN	EP	0.83
Central African Republic	24.68	78.62	32.28	22.44	23.60	87.44	26.36	23.49	UD & UE	Retrogression in both	EP	ED	-1.08
Chad	30.16	80.48	39.00	25.58	23.30	87.42	31.57	30.26	UD & UE	Retrogression in both	EP	EHN	-6.86
Chile	87.19	66.07	62.29	68.31	88.16	75.24	68.69	75.35	UE	Retrogression in ES	EHN	SD	0.97
China	80.34	55.78	64.99	63.75	81.64	69.47	63.28	74.19	SS	Progression	EHN	SD	1.29
Colombia	80.09	62.69	55.36	57.87	80.02	77.30	61.22	61.34	UE	Retrogression in ES	EHN	SD	-0.07
Congo, Dem. Rep.	34.67	61.54	45.89	25.39	33.04	85.49	45.15	35.05	UD & UE	Progression	EP	EHN	-1.63
Congo, Rep.	43.86	73.05	53.32	28.97	41.04	86.71	46.54	38.45	UD & UE	Progression	EP	ED	-2.82
Costa Rica	81.14	67.97	64.74	65.63	81.53	71.10	68.64	71.71	UE	Retrogression in ES	EHN	SD	0.39
Cote d'Ivoire	56.32	67.49	46.22	41.36	51.00	86.00	46.27	49.40	UD & UE	Retrogression in ES	EP	SD	-5.32
Croatia	87.30	76.04	71.73	69.23	87.82	78.83	78.16	73.72	SS	Progression	EHN	ED	0.52

Cuba	84.45	65.24	84.64	62.32	75.96	80.07	85.31	60.00	UE	Progression	SD	ED	-8.50
Cyprus	84.71	58.25	67.48	68.73	83.98	62.81	75.29	75.05	SS	Progression	EHN	EP	-0.73
Czech Republic	87.89	79.84	78.34	79.90	85.81	76.01	81.04	85.98	SS	Retrogression in ES	ED	EP	-2.08
Denmark	90.22	65.11	90.06	89.65	87.49	67.03	91.92	92.49	SS	Progression	ED	EP	-2.73
Djibouti	59.10	46.65	49.24	38.24	49.19	72.87	46.12	48.66	UD & UE	Progression	EP	SD	-9.91
Dominican Republic	76.24	72.82	60.15	56.53	76.23	84.11	59.12	63.24	UE	Progression	EP	SD	0.00
Ecuador	78.02	67.83	65.87	60.98	77.63	79.51	67.69	62.84	UE	Retrogression in ES	EP	ED	-0.39
Egypt, Arab Rep.	76.63	66.51	64.81	43.44	73.74	75.46	65.32	56.63	SS	Progression	EP	ED	-2.89
El Salvador	78.00	60.77	58.05	48.78	77.36	68.71	65.00	56.02	UE	Progression	EHN	ED	-0.64
Estonia	85.57	72.28	75.97	79.57	85.76	75.95	80.30	84.24	SS	Retrogression in ES	EHN	EP	0.19
Eswatini	N. A.	N. A.	N. A.	N. A.	45.55	73.50	50.39	46.14	UD & UE	N. A.	EP	EHN	N. A.
Ethiopia	51.50	69.57	54.91	38.39	43.45	84.53	52.11	42.48	UD & UE	Retrogression in ES	EP	ED	-8.05
Fiji	N. A.	N. A.	N. A.	N. A.	73.14	71.89	68.86	71.20	SS	N. A.	EHN	SD	N. A.
Finland	90.37	66.45	89.20	88.22	89.94	71.21	90.44	91.19	SS	Progression	ED	EP	-0.43
France	90.88	66.92	78.00	84.43	89.93	65.58	83.47	86.38	SS	Progression	EHN	EP	-0.96
Gabon	70.77	73.87	64.41	45.32	66.69	80.99	52.48	49.34	UD	Retrogression in MIS	EP	ED	-4.07
Gambia, The	44.91	66.32	47.91	27.84	51.13	86.74	53.14	46.39	UD & UE	Retrogression in ES	EP	ED	6.22
Georgia	77.33	64.99	70.95	54.83	76.28	75.99	71.57	61.59	SS	Progression	EHN	ED	-1.05
Germany	90.13	66.48	82.07	87.20	88.81	64.49	85.71	90.55	SS	Progression	ED	EP	-1.32
Ghana	62.22	70.73	58.65	43.67	60.25	79.10	57.24	52.85	UD & UE	Progression	EP	ED	-1.97
Greece	86.83	62.12	69.69	69.34	86.06	67.38	73.40	71.71	SS	Progression	EHN	EP	-0.77
Guatemala	67.61	59.70	51.81	51.87	65.19	72.54	48.11	53.96	UD & UE	Retrogression in ES	EP	SD	-2.42
Guinea	39.60	74.32	47.08	32.87	36.29	81.94	47.02	40.69	UD & UE	Retrogression in ES	EP	EHN	-3.31
Guyana	79.07	56.74	63.72	52.78	71.29	42.68	70.53	49.00	UD & UE	Retrogression in MIS	EHN	EP	-7.78
Haiti	38.25	60.83	46.13	28.06	39.37	78.46	51.60	34.75	UD & UE	Progression	EP	ED	1.12
Honduras	70.91	66.32	57.26	47.33	66.68	77.75	51.52	55.04	UD & UE	Retrogression in ES	EP	SD	-4.22
Hungary	86.48	80.36	71.94	71.60	87.13	79.76	72.89	78.82	SS	Progression	EHN	SD	0.65
Iceland	92.65	51.08	87.75	80.54	85.63	49.28	89.45	85.47	SS	Retrogression in both	SD	EP	-7.03
India	62.63	61.52	58.46	45.21	59.21	75.18	51.61	55.49	UD & UE	Retrogression in ES	EP	SD	-3.42
Indonesia	69.74	64.12	62.42	50.59	69.11	71.90	63.27	59.45	SS	Progression	EP	ED	-0.63
Iran, Islamic Rep.	75.02	62.71	60.21	57.64	77.23	76.69	62.48	61.65	SS	Progression	EHN	ED	2.21
Iraq	67.16	53.55	58.66	39.81	70.83	68.96	63.41	45.94	UD	Progression	EHN	ED	3.67
Ireland	88.78	63.83	75.13	83.26	89.93	66.09	80.86	86.03	SS	Progression	EHN	EP	1.15
Israel	84.02	49.15	66.44	81.14	84.81	54.01	74.12	88.33	SS	Progression	ED	EP	0.79
Italy	87.51	64.54	71.25	77.15	87.82	68.55	76.60	80.84	SS	Progression	EHN	EP	0.31
Jamaica	78.61	59.22	65.42	58.22	75.68	68.78	66.55	62.07	SS	Progression	EHN	ED	-2.93
Japan	90.06	64.85	75.78	91.50	89.40	62.15	79.93	87.39	SS	Retrogression in both	EHN	EP	-0.66
Jordan	75.34	58.85	68.81	55.05	76.21	77.76	63.36	61.16	SS	Progression	EP	ED	0.86
Kazakhstan	77.92	58.75	74.28	66.74	80.64	64.32	72.99	65.98	SS	Progression	EHN	EP	2.71
Kenya	51.78	61.92	55.91	49.22	52.03	78.50	57.65	55.93	UD & UE	Retrogression in ES	EP	EHN	0.25
Korea, Rep.	87.86	60.63	69.06	83.21	88.29	61.23	76.84	88.51	SS	Progression	ED	EP	0.43
Kuwait	77.00	39.92	72.93	53.99	73.58	40.78	71.48	53.80	UD	Retrogression in MIS	EHN	EP	-3.42
Kyrgyz Republic	80.60	71.97	74.50	46.43	75.30	83.36	77.80	61.19	SS	Progression	EP	ED	-5.30
Lao PDR	62.15	70.63	62.61	48.76	60.99	79.79	59.35	56.50	UD & UE	Retrogression in ES	EP	ED	-1.17
Latvia	84.31	71.55	70.61	72.69	86.57	77.02	73.70	78.71	SS	Progression	EHN	SD	2.27
Lebanon	77.71	59.59	58.78	60.99	77.74	71.52	61.42	51.44	SS	Progression	EHN	ED	0.03
Lesotho	43.34	73.11	58.32	39.96	40.31	85.95	54.21	43.16	UD & UE	Retrogression in ES	EP	EHN	-3.03
Liberia	28.80	64.49	44.11	35.16	32.79	82.49	48.19	30.71	UD & UE	Retrogression in ES	EP	ED	3.99

Lithuania	81.44	67.28	70.55	74.18	81.93	76.65	72.31	75.37	SS	Progression	EHN	SD	0.49
Luxembourg	83.04	52.66	75.16	83.65	80.22	42.90	82.64	85.06	UE	Retrogression in both	ED	EP	-2.82
Madagascar	23.76	67.19	51.06	32.22	30.76	76.16	50.07	41.44	UD & UE	Progression	EP	EHN	7.00
Malawi	37.10	71.46	53.72	33.28	34.23	82.81	52.01	41.90	UD & UE	Retrogression in ES	EP	EHN	-2.87
Malaysia	81.92	57.40	63.78	75.49	79.02	63.85	64.40	77.52	SS	Progression	EHN	EP	-2.90
Maldives	N. A.	N. A.	N. A.	N. A.	79.64	66.96	63.01	65.51	SS	N. A.	EHN	SD	N. A.
Mali	42.64	75.94	46.76	33.95	39.92	82.63	46.57	46.13	UD & UE	Retrogression in ES	EP	EHN	-2.72
Malta	83.81	70.78	75.67	76.28	85.84	59.86	76.01	79.66	SS	Retrogression in MIS	EHN	EP	2.03
Mauritania	57.60	64.94	49.15	25.30	51.26	81.70	51.52	34.29	UD & UE	Progression	EP	ED	-6.34
Mauritius	74.99	37.49	67.76	64.13	80.39	53.49	63.35	67.13	UE	Progression	EHN	EP	5.40
Mexico	80.42	65.34	62.70	66.11	78.86	69.62	61.46	65.05	UE	Retrogression in both	EHN	SD	-1.56
Moldova	80.00	79.06	77.25	54.67	79.42	78.64	78.82	55.51	UE	Progression	EHN	ED	-0.58
Mongolia	65.27	66.57	70.49	49.46	65.77	58.95	73.72	49.79	UD & UE	Retrogression in MIS	SD	ED	0.50
Montenegro	79.90	46.30	78.17	56.16	75.27	58.78	71.98	62.71	SS	Progression	EHN	EP	-4.62
Morocco	75.49	68.33	61.44	58.42	76.94	80.22	62.53	60.25	SS	Progression	EP	ED	1.45
Mozambique	35.15	72.65	55.70	30.74	35.91	79.02	49.14	42.21	UD & UE	Retrogression in ES	EP	EHN	0.76
Myanmar	63.55	62.24	73.32	30.87	64.96	77.05	65.03	48.65	UE	Progression	EP	ED	1.41
Namibia	54.87	69.36	63.23	46.56	50.64	81.28	62.92	52.40	UD & UE	Progression	EP	EHN	-4.23
Nepal	66.58	80.19	64.82	29.17	61.68	86.88	70.83	48.97	UE	Progression	EP	ED	-4.89
Netherlands	89.23	60.53	80.99	88.59	88.68	61.10	84.66	91.79	SS	Progression	ED	EP	-0.55
New Zealand	88.99	54.75	82.12	83.16	89.58	54.78	83.24	87.34	SS	Progression	EHN	EP	0.59
Nicaragua	72.31	67.73	59.52	47.59	70.88	79.28	59.58	52.50	UE	Progression	EP	ED	-1.43
Niger	34.57	70.19	49.66	28.36	32.94	86.95	46.49	38.65	UD & UE	Retrogression in ES	EP	EHN	-1.63
Nigeria	44.89	69.39	45.89	31.84	40.20	82.73	36.79	38.61	UD & UE	Progression	EP	SD	-4.69
North Macedonia	N. A.	N. A.	N. A.	N. A.	78.44	81.70	71.90	59.11	SS	N. A.	EP	ED	N. A.
Norway	89.63	62.63	91.30	90.59	87.21	53.01	94.64	90.78	SS	Retrogression in both	SD	EP	-2.42
Oman	73.26	57.12	62.96	60.75	70.88	65.77	70.99	67.75	SS	Progression	SD	EP	-2.38
Pakistan	63.34	67.77	49.91	36.13	56.62	79.20	50.74	42.56	UD & UE	Progression	EP	ED	-6.72
Panama	77.61	61.29	51.84	64.85	78.69	71.89	54.99	66.58	UE	Retrogression in ES	EHN	SD	1.08
Papua New Guinea	N. A.	N. A.	N. A.	N. A.	35.69	77.61	46.18	50.95	UD & UE	N. A.	EP	EHN	N. A.
Paraguay	83.55	66.81	57.24	51.27	81.15	71.45	54.12	61.52	UE	Progression	EHN	SD	-2.40
Peru	78.74	66.35	61.40	51.79	80.54	78.78	63.14	58.33	SS	Progression	EHN	ED	1.80
Philippines	70.81	68.21	62.70	51.08	65.95	78.86	58.54	52.90	UE	Retrogression in ES	EP	ED	-4.85
Poland	85.13	67.64	74.10	73.88	85.41	77.10	77.95	79.53	SS	Progression	EHN	EP	0.29
Portugal	87.62	60.21	74.57	77.62	86.72	63.65	80.54	82.00	SS	Retrogression in ES	EHN	EP	-0.90
Qatar	74.94	48.65	65.16	59.21	75.97	54.14	67.70	61.57	UE	Retrogression in ES	EHN	EP	1.03
Romania	80.74	75.33	71.75	65.48	83.23	78.22	65.34	72.95	SS	Progression	EHN	SD	2.49
Russian Federation	75.80	69.94	61.01	69.34	79.80	67.15	71.42	76.34	SS	Progression	EHN	EP	4.00
Rwanda	47.42	76.25	60.80	36.76	42.40	80.83	61.10	50.27	UD & UE	Retrogression in ES	EP	EHN	-5.02
Sao Tome and Principe	N. A.	N. A.	N. A.	N. A.	48.83	83.55	56.21	46.68	UD & UE	N. A.	EP	ED	N. A.
Saudi Arabia	72.61	53.18	75.24	42.03	69.06	57.84	66.85	64.56	SS	Progression	EHN	EP	-3.56
Senegal	57.64	65.67	53.40	46.06	53.26	81.64	48.59	52.51	UD & UE	Retrogression in ES	EP	SD	-4.38
Serbia	83.14	68.97	76.02	58.18	82.33	74.00	77.12	68.98	SS	Progression	EHN	ED	-0.80
Sierra Leone	29.33	69.49	57.42	29.73	31.28	79.07	60.35	34.79	UD & UE	Retrogression in ES	EP	EHN	1.95
Singapore	88.66	34.69	63.35	91.24	85.64	36.71	75.79	85.04	UE	Retrogression in ES	EHN	EP	-3.03
Slovak Republic	88.04	69.80	74.37	69.83	86.95	76.33	78.75	77.30	SS	Progression	EHN	EP	-1.09
Slovenia	88.35	71.27	83.82	74.44	87.41	68.25	85.09	83.90	SS	Progression	EHN	EP	-0.93

Somalia	N. A.	N. A.	N. A.	N. A.	25.53	75.75	44.80	37.31	UD & UE	N. A.	EP	EHN	N. A.
South Africa	64.25	63.45	60.40	54.71	60.00	71.11	59.64	66.99	UD & UE	Progression	EP	SD	-4.25
South Sudan	N. A.	N. A.	N. A.	N. A.	23.30	82.97	32.35	22.04	UD & UE	N. A.	EP	ED	N. A.
Spain	87.19	62.32	74.76	81.90	87.64	64.68	79.87	84.86	SS	Progression	EHN	EP	0.45
Sri Lanka	74.24	66.46	62.44	57.08	71.01	77.04	60.22	64.45	SS	Progression	EP	SD	-3.22
Sudan	47.44	59.51	53.24	35.46	40.55	75.32	49.57	29.78	UD & UE	Retrogression in both	EP	ED	-6.88
Suriname	75.46	71.36	71.13	59.45	69.48	76.69	65.65	64.66	SS	Retrogression in both	EP	ED	-5.98
Sweden	91.96	65.19	90.76	93.65	90.21	67.02	92.06	92.00	SS	Retrogression in ES	SD	EP	-1.75
Switzerland	91.54	61.71	76.10	91.86	90.73	54.97	80.46	92.46	SS	Retrogression in MIS	ED	EP	-0.80
Syrian Arab Republic	63.64	59.99	58.17	48.32	63.29	71.50	46.88	40.10	UD & UE	Retrogression in both	EP	ED	-0.36
Tajikistan	70.81	72.44	74.21	41.98	73.76	82.98	70.13	52.87	UE	Retrogression in ES	EP	ED	2.95
Tanzania	43.79	66.77	59.76	33.49	41.52	81.75	54.47	50.80	UD & UE	Retrogression in ES	EP	EHN	-2.27
Thailand	80.65	62.88	65.45	66.70	79.59	74.70	70.44	70.76	SS	Progression	EHN	SD	-1.06
Togo	44.32	66.45	55.30	29.71	39.81	84.29	50.22	39.21	UD & UE	Retrogression in ES	EP	ED	-4.51
Trinidad and Tobago	77.05	54.31	77.44	64.50	79.48	50.04	68.22	60.38	UE	Retrogression in both	EHN	EP	2.42
Tunisia	78.09	69.03	68.61	52.54	76.80	77.38	69.84	57.28	SS	Progression	EP	ED	-1.29
Turkey	83.18	61.67	61.19	65.25	82.38	68.01	63.83	64.45	SS	Retrogression in ES	EHN	SD	-0.80
Turkmenistan	67.33	65.05	51.94	38.71	58.41	64.95	65.80	52.68	UD & UE	Progression	SD	ED	-8.92
Uganda	45.92	78.72	55.49	34.17	39.88	83.77	51.12	44.82	UD & UE	Retrogression in MIS	EP	EHN	-6.05
Ukraine	81.95	67.51	74.84	60.82	81.98	72.50	79.52	62.06	SS	Progression	EHN	ED	0.03
United Arab Emirates	76.58	42.50	81.54	59.01	80.07	53.88	73.37	70.07	SS	Retrogression in ES	EHN	EP	3.48
United Kingdom	89.21	60.94	75.86	87.20	89.17	65.24	77.81	87.88	SS	Progression	EHN	EP	-0.04
United States	88.62	45.70	67.36	89.39	86.97	57.87	72.54	87.71	SS	Progression	ED	EP	-1.65
Uruguay	87.47	55.03	69.33	67.90	85.10	67.08	72.60	70.17	SS	Progression	EHN	EP	-2.37
Uzbekistan	74.51	73.70	81.38	46.47	72.96	81.74	70.47	55.33	UE	Retrogression in MIS	EP	ED	-1.56
Vanuatu	N. A.	N. A.	N. A.	N. A.	49.83	70.86	60.02	65.39	UD & UE	N. A.	EP	EHN	N. A.
Venezuela, RB	77.12	65.94	57.22	61.22	58.40	75.20	53.01	50.22	UD & UE	Retrogression in both	EP	ED	-18.72
Vietnam	79.76	60.75	72.04	50.72	79.00	70.44	73.09	65.41	SS	Progression	EHN	ED	-0.77
Yemen, Rep.	40.95	66.52	54.86	33.84	46.18	78.57	41.25	33.71	UD & UE	Progression	EP	ED	5.23
Zambia	43.80	75.08	53.77	34.71	43.40	86.84	46.48	43.24	UD & UE	Retrogression in ES	EP	ED	-0.40
Zimbabwe	47.49	73.89	60.26	45.78	46.90	89.78	60.80	46.00	UD & UE	Retrogression in ES	EP	ED	-0.60

Table S7. Major existing literatures related to SDGs assessment at multiple scales

References	Scales	Major contributions	Innovations of our study
Schmidt-Traub et al., 2017[9]	global	Build up the indicator system and assessment methods for global SDGs assessment (the mean SDG index score, MIS).	Our study develops a new dimension for global SDGs assessment – the SDGs progress evenness, which expands the implications of sustainable development assessment and could reduce the potential overestimation of SDGs performance when large progress differences exists.
Sachs et al., 2016-2022[1-5,16,17]	global	Present the average SDGs performance of each country.	Hidden challenges are revealed by integrating SDGs progress evenness. For instance, the progress of many high-income countries becomes stagnant, despite they generally show the best average performance toward SDGs.
Xu et al., 2020[6]	provincial (China)	First national SDGs assessment over time and space.	Not related to our study.
Fu et al., 2019 and 2020[14,18]	N.A.[*]	Clarify the logic and categorization of the SDGs to facilitate management.	Based on these categories, our study proposes a framework for the priority selection by integrating the SDGs progress evenness, which could help with future SDGs policymaking.
Liu et al., 2021[7]	provincial (China)	First adopt SDGs progress evenness in national SDGs assessment, with hidden challenges disclosed for China.	Our study improves the methodology and first applies it in global SDGs assessment.
Zhu et al., 2023[19]	municipal (Inner Mongolia, China)	Reveal the challenges for the achievement of SDGs in drylands.	Hidden challenges and future opportunities are identified for different categories of countries. For instances, low-, middle-, and high-income countries; slightly, moderately, severely, and extremely arid countries.

REFERENCES

1. Sachs, J., Schmidt-Traub, G., Kroll, C., et al. (2017). SDG Index and Dashboards Report 2017. New York: Bertelsmann Stiftung and Sustainable Development Solutions Network (SDSN).
2. Sachs, J., Schmidt-Traub, G., Kroll, C., et al. (2019). Sustainable Development Report 2019. New York: Bertelsmann Stiftung and Sustainable Development Solutions Network (SDSN).
3. Sachs, J., Schmidt-Traub, G., Kroll, C., et al. (2020). The Sustainable Development Goals and COVID-19. Sustainable Development Report 2020. Cambridge: Cambridge University Press.
4. Sachs, J., Kroll, C., Lafortune, G., et al. (2021). Sustainable Development Report 2021. Cambridge: Cambridge University Press.
5. Sachs, J., Schmidt-Traub, G., Kroll, C., et al. (2018). SDG Index and Dashboards Report 2018. New York: Bertelsmann Stiftung and Sustainable Development Solutions Network (SDSN).
6. Xu, Z., Chau, S.N., Chen, X., et al. (2020). Assessing progress towards sustainable development over space and time. Nature ***577***, 74-78. DOI: 10.1038/s41586-019-1846-3.
7. Liu, Y., Du, J., Wang, Y., et al. (2021). Evenness is important in assessing progress towards sustainable development goals. Natl. Sci. Rev. ***8***, nwaa238. DOI: 10.1093/nsr/nwaa238.
8. Liu, Y., Du, J., Xu, X., et al. (2020). Microtopography-induced ecohydrological effects alter plant community structure. Geoderma ***362***, 114119. DOI: 10.1016/j.geoderma.2019.114119.
9. Schmidt-Traub, G., Kroll, C., Teksoz, K., et al. (2017). National baselines for the Sustainable Development Goals assessed in the SDG Index and Dashboards. Nat. Geosci. ***10***, 547-555. DOI: 10.1038/ngeo2985.
10. Fu, B., Stafford-Smith, M., Wang, Y., et al. (2021). The Global-DEP conceptual framework - research on dryland ecosystems to promote sustainability. Curr. Opin. Env. Sust. ***48***, 17-28. DOI: 10.1016/j.cosust.2020.08.009.
11. Yao, J., Liu, H., Huang, J., et al. (2020). Accelerated dryland expansion regulates future variability in dryland gross primary production. Nat. Commun. ***11***, 1665. DOI: 10.1038/s41467-020-15515-2.
12. Huang, J.P., Yu, H.P., Guan, X.D., et al. (2016). Accelerated dryland expansion under climate change. Nat. Clim. Change ***6***, 166-171. DOI: 10.1038/nclimate2837.
13. Pravalie, R. (2016). Drylands extent and environmental issues. A global approach. Earth-Sci. Rev. ***161***, 259-278. DOI: 10.1016/j.earscirev.2016.08.003.
14. Fu, B., Wang, S., Zhang, J., et al. (2019). Unravelling the complexity in achieving the 17 sustainable-development goals. Natl. Sci. Rev. ***6***, 386-388. DOI: 10.1093/nsr/nwz038.
15. Pradhan, P., Costa, L., Rybski, D., et al. (2017). A Systematic Study of Sustainable Development Goal (SDG) Interactions. Earth's Future ***5***, 1169-1179. DOI: 10.1002/2017EF000632.
16. Sachs, J., Schmidt-Traub, G., Kroll, C., et al. (2016). An SDG Index and Dashboards - Global Report. New York: Bertelsmann Stiftung and Sustainable Development Solutions Network (SDSN).
17. Sachs, J., Lafortune, G., Kroll, C., et al. (2022). From Crisis to Sustainable Development: the SDGs as Roadmap to 2030 and Beyond. Sustainable Development Report 2022. Cambridge: Cambridge University Press.
18. Fu, B., Zhang, J., Wang, S., and Zhao, W. (2020). Classification–coordination–collaboration: a systems approach for advancing Sustainable Development Goals. Natl. Sci. Rev. ***7***, 838-840. DOI: 10.1093/nsr/nwaa048.
19. Zhu, J., Yang, Y., Liu, Y., et al. (2023). Progress and water stress of sustainable development in Chinese northern drylands. J. Clean. Prod. ***399***, 136611. DOI: 10.1016/j.jclepro.2023.136611.
